# Supplementary material for: Modelling the demographic history of human North African genomes points to a recent soft split divergence between populations
Source: Genome Biol. 2024 Jul 30;25:201. doi: 10.1186/s13059-024-03341-4 (PMC11290046; doi:10.1186/s13059-024-03341-4)
Supplement: Supplementary file 1 — Additional file 1: Supplementary figures S1 to S11 and Supplementary Tables S1 to S10. [file 13059_2024_3341_MOESM1_ESM.pdf]

Figures & Tables (Supplementary):

ABC-DL Figures:

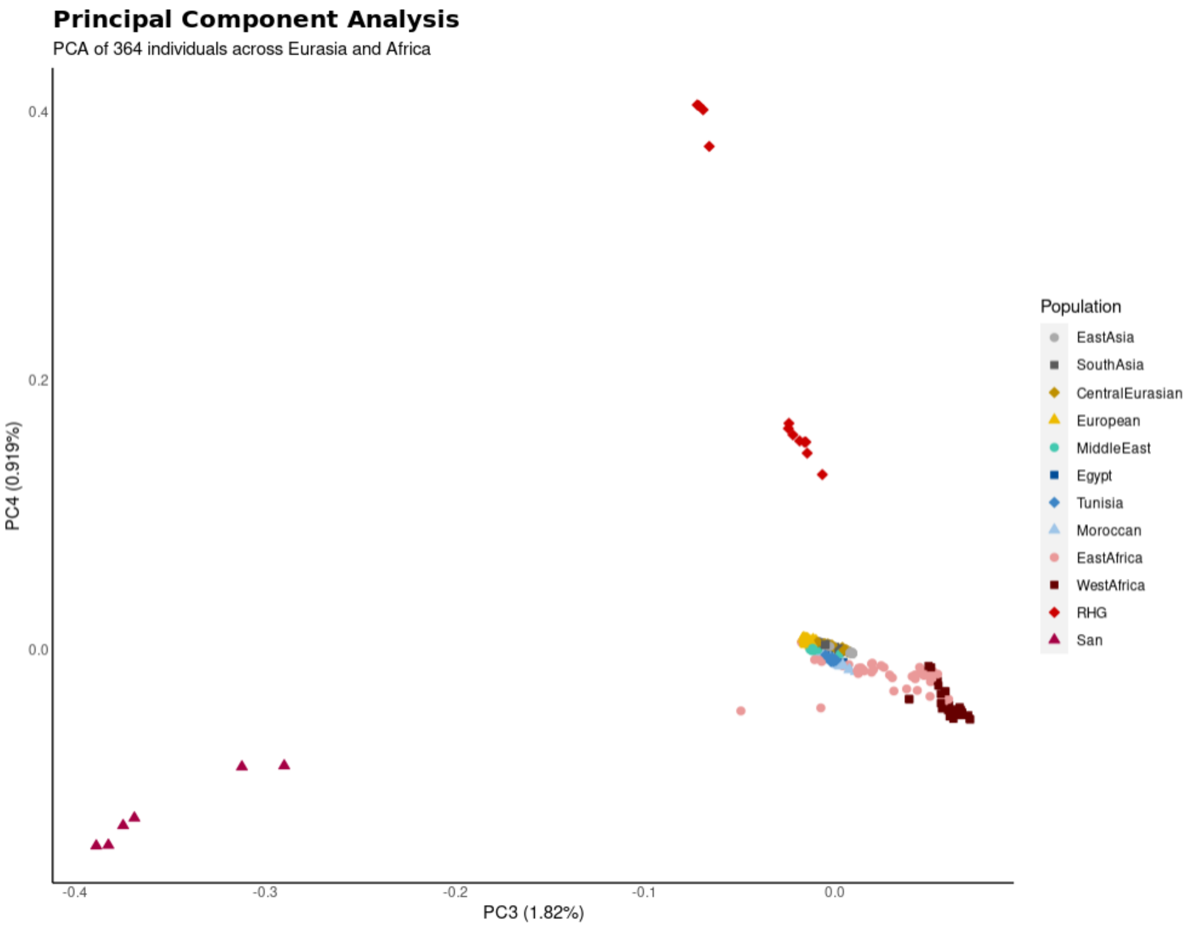

Fig. S 1: Principal Component Analysis on genomic dataset of North Africa. Visualization of PC3 & PC4

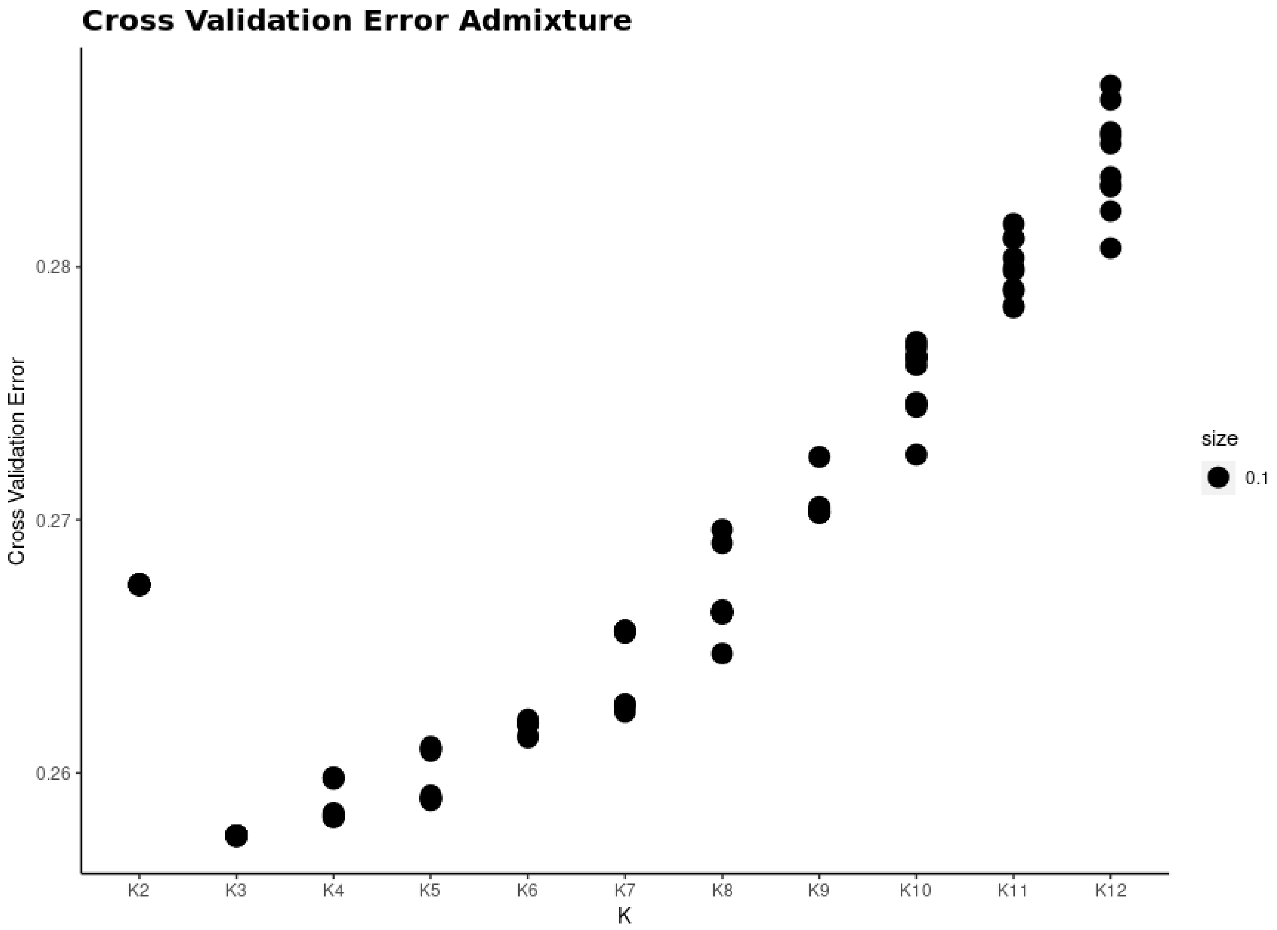

Fig. S 2: **CrossValidation error of ADMIXTURE analysis with K=2 to K=12. Best K is K=3**

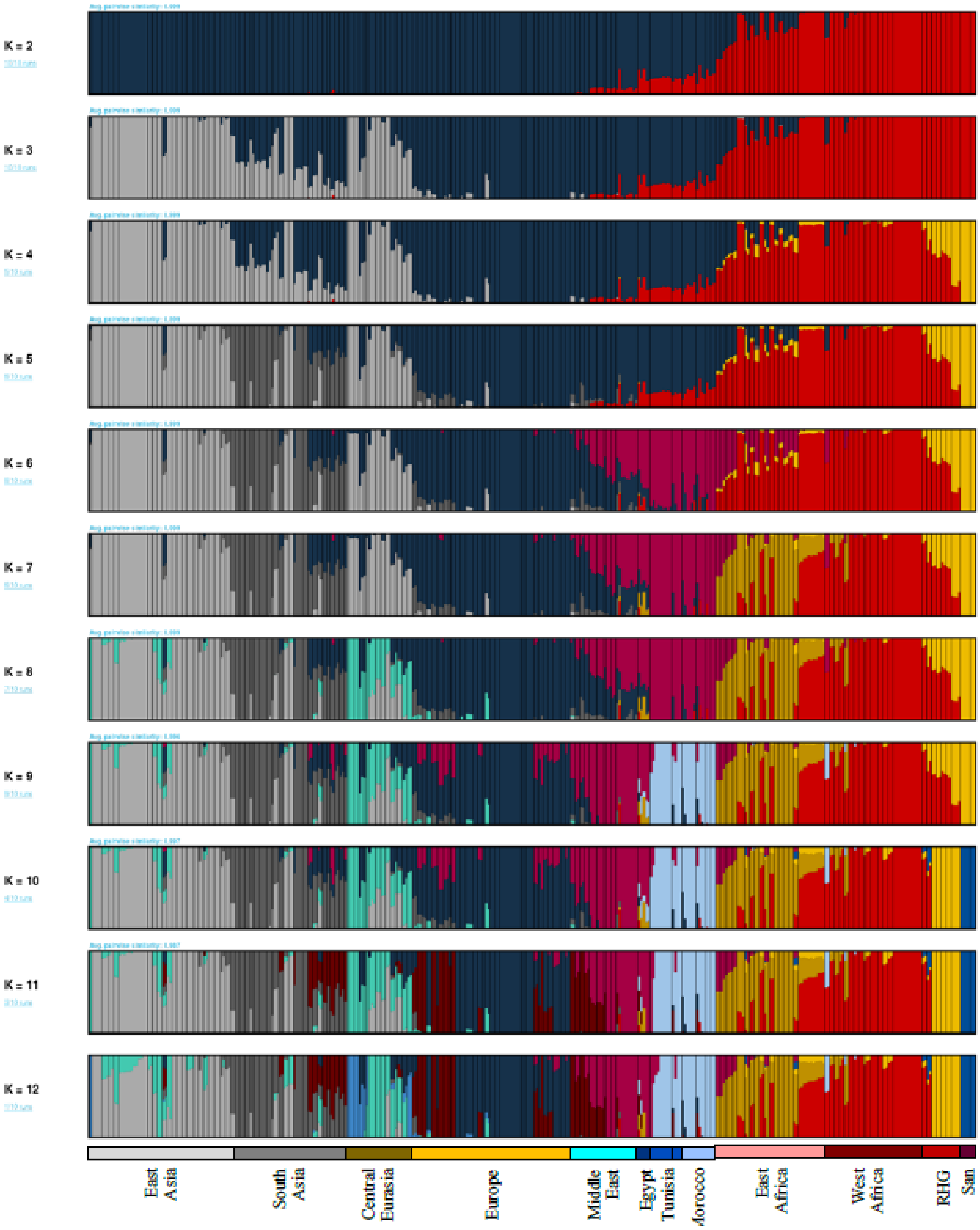

Fig. S 3: ADMIXTURE analysis on 364 individuals with K=2 to K=12

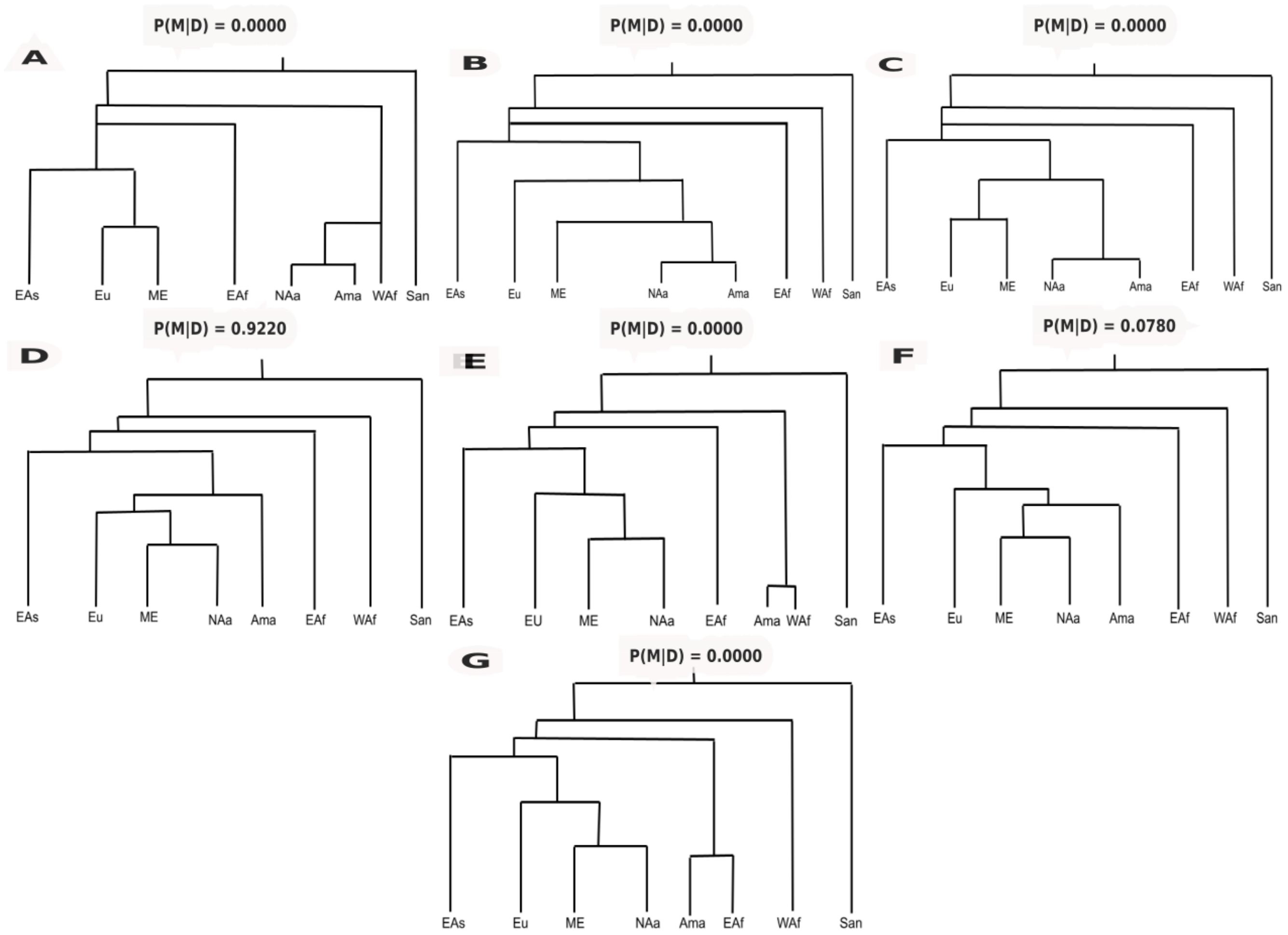

Fig. S 4: Competing topologies tested in ABC-DL *analysis*. Seven different topologies included on the ABC-DL analyses considering North African Arab (NAa), North Africa Amazigh (Ama), Middle Eastern (ME), European (Eu), East Asian (EAs), East African (EAf), West African (WAf), and Ju/’hoansi (San) populations.

	ModelA	ModelB	ModelC	ModelD	ModelE	ModelF	ModelG
**ModelA**	**1**	0	0	0	0	0	0
**ModelB**	0	**0.7941**	0.1023	0.0122	0	0.0914	0
**ModelC**	0	0.0603	**0.9395**	0.0001	0	0.0001	0
**ModelD**	0	0.0145	0.002	**0.7579**	0	0.2256	0.0001
**ModelE**	0	0	0	0	**1**	0	0
**ModelF**	0	0.1565	0.002	0.1881	0	**0.6534**	0.0001
**ModelG**	0	0	0	0	0	0	**1**

Table S1: Confusion matrix computed with the 7 models under evaluation. 50 randomly sampled simulations per model were used as "observed" data for the ABC-DL algorithm. Diagonal, in bold, shows the probability of a model being correctly assigned by the A

ModelA	ModelB	ModelC	ModelD	ModelE	ModelF	ModelG
0	0	0	0.922	0	0.078	0

Table S 2:Proportion of accepted simulations using postpr function for the "abc" package with tolerance = 0.0008. Model D is present 92.2% of times in the 1000 closest simulations to the observed data.

	ModelA	ModelB	ModelC	ModelD	ModelE	ModelF	ModelG
**ModelA**	NA	NA	NA	Inf	NA	Inf	NA
**ModelB**	NA	NA	NA	Inf	NA	Inf	NA
**ModelC**	NA	NA	NA	Inf	NA	Inf	NA
**ModelD**	0	0	0	1	0	0.08459	0
**ModelE**	NA	NA	NA	Inf	NA	Inf	NA
**ModelF**	0	0	0	11.8205	0	1	0
**ModelG**	NA	NA	NA	Inf	NA	Inf	NA

Table S 3: Bayes factor for the ABC-DL topology discrimination analysis. Model D is 11.8 times better at explaining the observed data than the second-best model (Model F).

	Model D1	Model D2	Model D3	Model D4	Model D5
**Model D1**	**0.5888**	0.0058	0.0064	0.0061	0.3933
**Model D2**	0.0019	**0.3712**	0.3499	0.2759	0.0011
**Model D3**	0.0003	0.3507	**0.3557**	0.2931	0.0001
**Model D4**	0.0003	0.2729	0.3020	**0.4248**	0
**Model D5**	0.3888	0	0	0	**0.6112**

Table S 4: Confusion matrix computed with the five D models under evaluation. 50 randomly sampled simulations per model were used as "observed" data for the ABC-DL algorithm. Diagonal, in bold, shows the probability of a model being correctly assigned by the ABC.

Model D1	Model D2	Model D3	Model D4	Model D5
0.0167	0.0800	0.0944	0.7622	0.0468

Table S 5: Proportion of accepted simulations using postpr function for the "abc" package with tolerance = 0.001. Model D4 is present 76.22% of times in the 1000 closest simulations to the observed data.

	Model D1	Model D2	Model D3	Model D4	Model D5
**Model D1**	1	0.2087	0.1768	0.0219	0.3567
**Model D2**	4.7913	1	0.8471	0.1049	1.7093
**Model D3**	5.6560	1.1805	1	0.1238	2.0178
**Model D4**	45.6714	9.5321	8.0748	1	16.2932
**Model D5**	2.8031	0.5850	0.4956	0.0614	1

Table S 6: Bayes factor for the ABC-DL with different admixture patterns. Model D4 is 8.074 times better at explaining the observed data than the second-best model (Model D3).

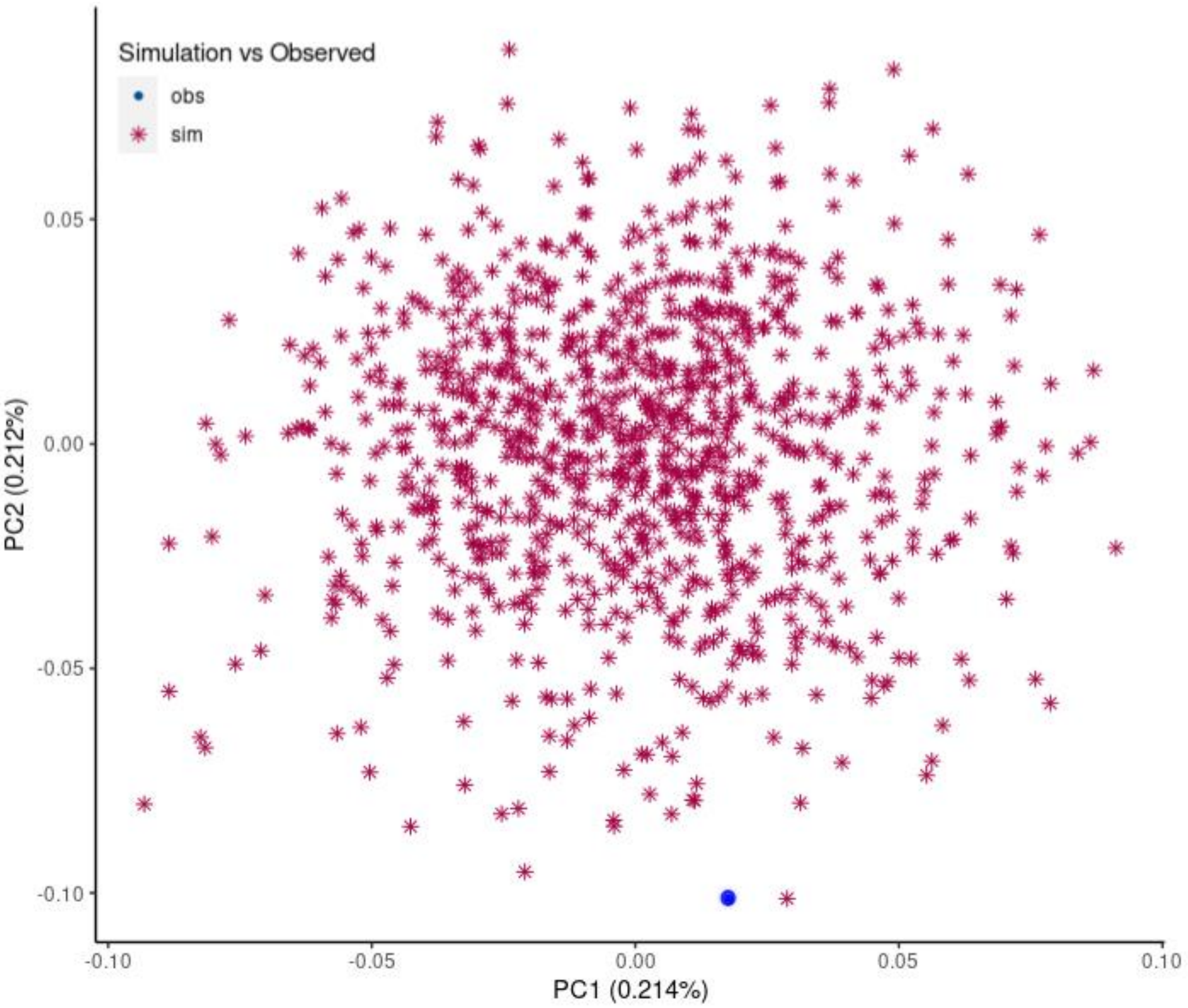

Fig. S 5: Replication PCA for Model D_4 in ABC-DL analysis. PCA for 1000 simulations of the model D_4, -the best model in the ABC-DL analysis- and the replication dataset of observed data. Observed data is an outlier in the PCA indicating that the ABC-DL model cannot properly replicate the diversity observed in the dataset.

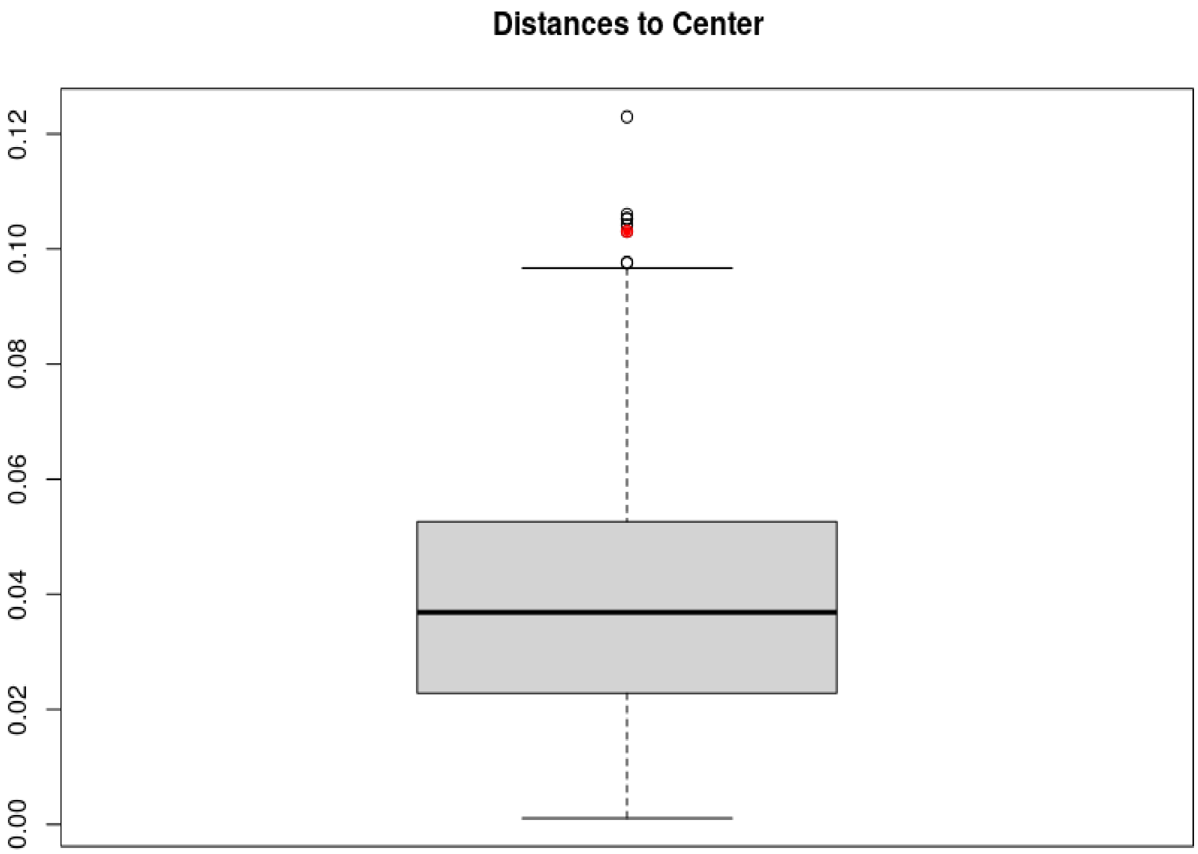

Fig. S 6: Box plot of the distances between each simulation in the PCA and the centroid of the PCA. The red dot represents the observed data as an outlier of the distances.

GP4PG Figures:

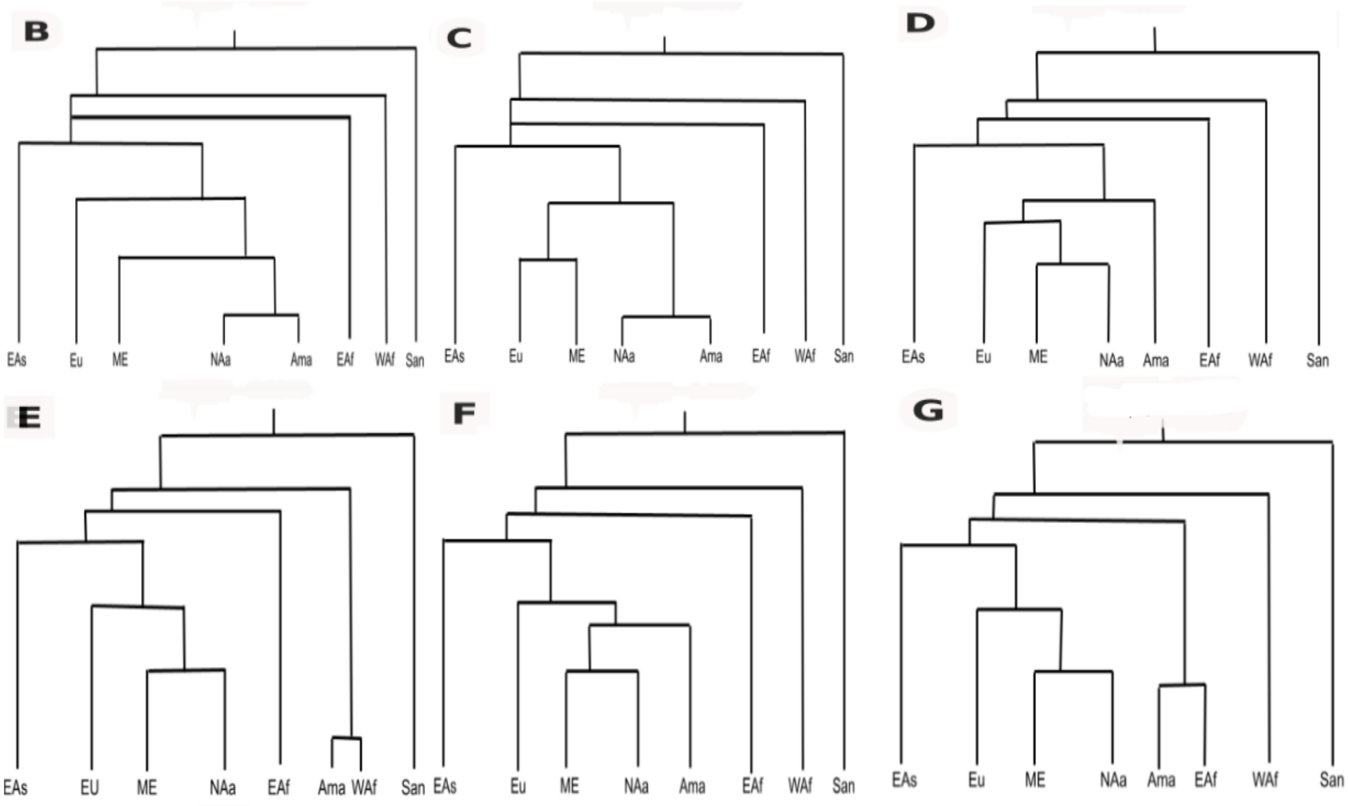

Fig. S 7: Competing topologies tested in GP4PG analysis. The competing topologies for the GP4PG analysis are the same as the ones used in the ABC-DL analysis but discarding Model A, due to being the worst performing one in previous analysis and due resource consumption.

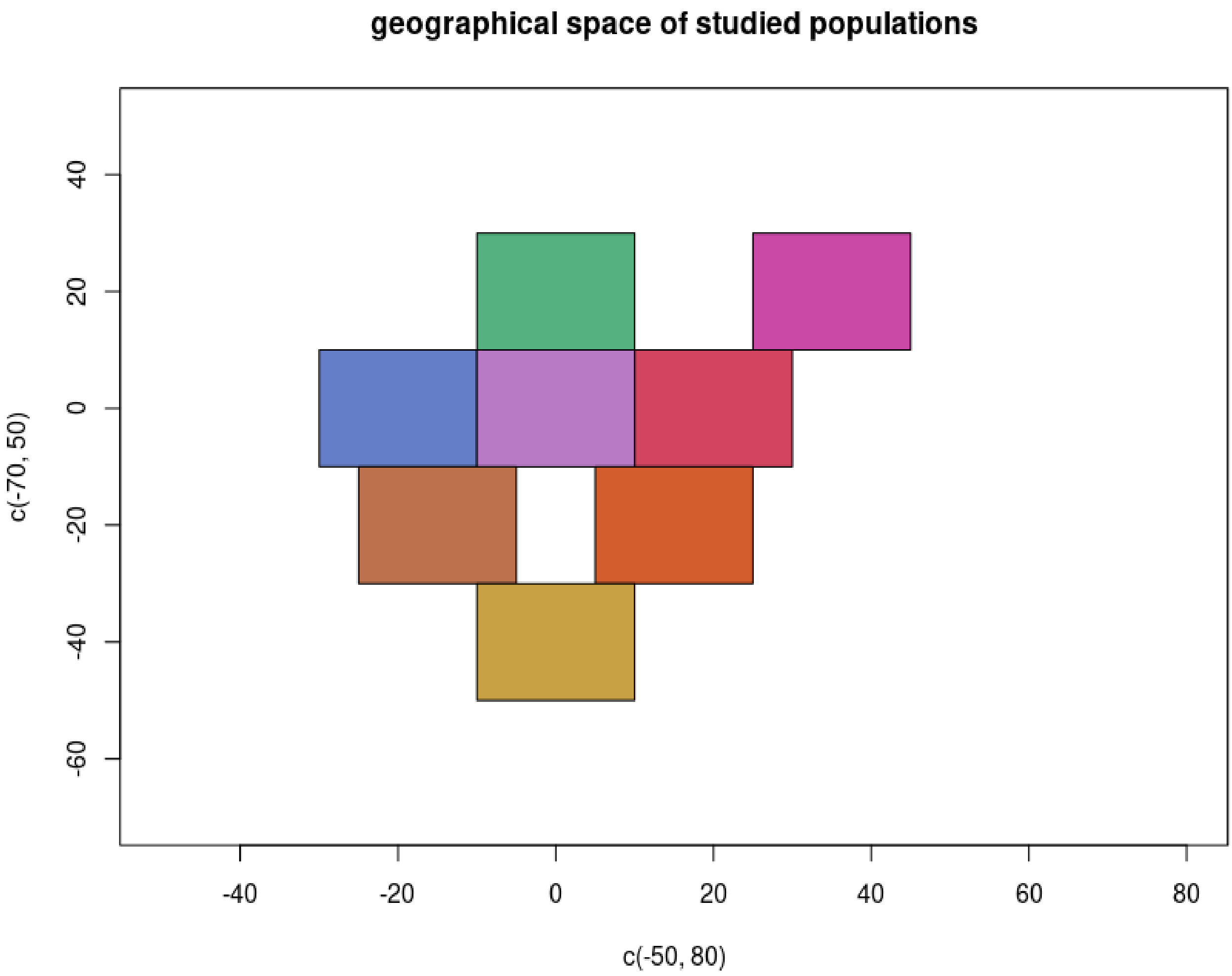

Fig. S 8: Coordinates of the different ecodemes we are testing in the GP4PG analysis. Each ecodeme has the exact same size and major geographical barriers such as seas and deserts has been removed for the sake of simplicity.

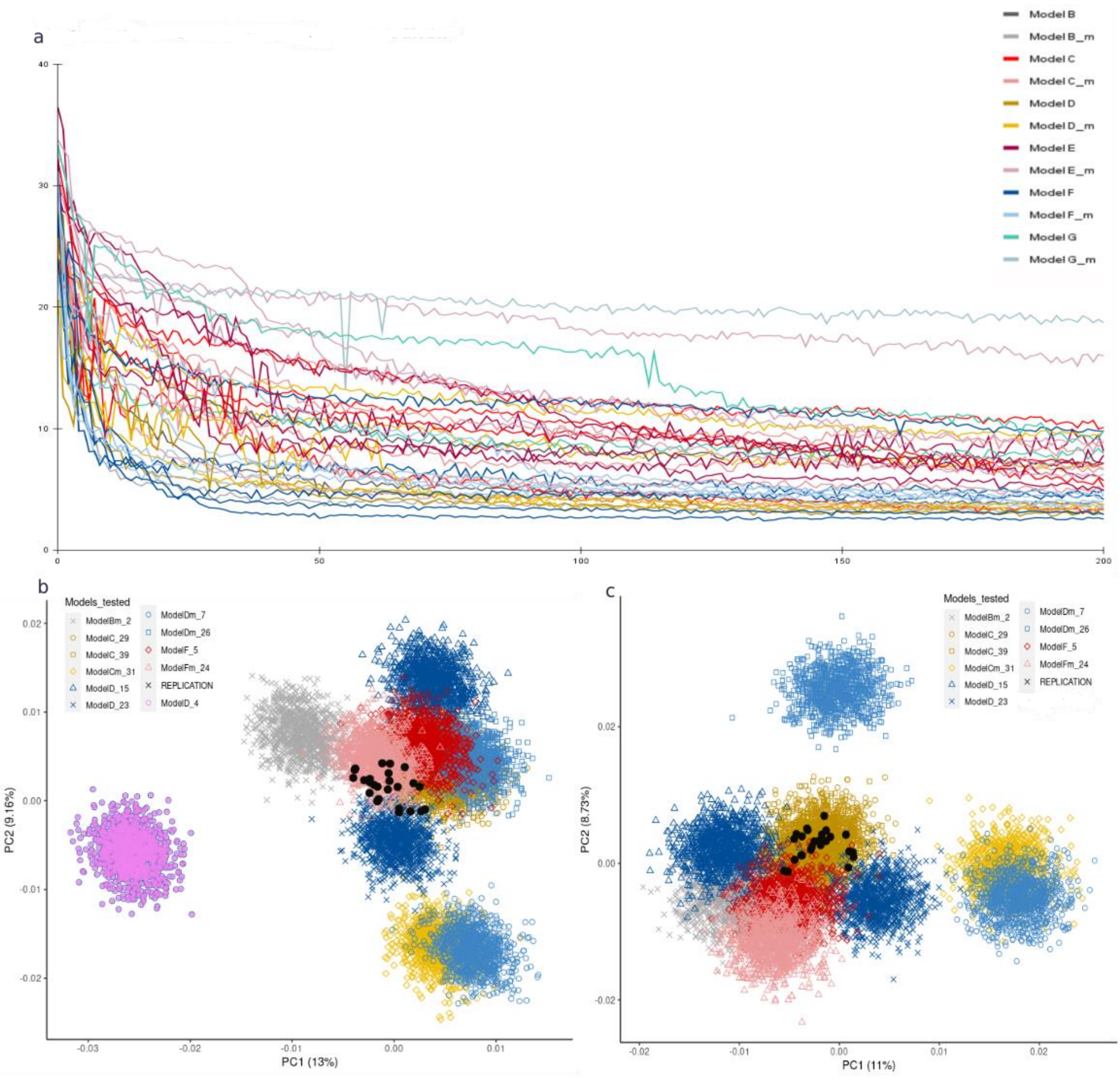

Fig. S 9: Fitness of the different runs of the genetic algorithm. **a.** Distribution of the fitness error of 40 independent iterations of the GP4PG algorithm with 6 competing topologies (B to G in ABC-DL) during 200 generations. Model D appears as the most selected model in a fourth of all the iterations, with D_15 as the model with the least error. **b.** PCA plot comparing the jSFS obtained from simulations of the best ABC-DL model with the 10 best GP4PG models. GP4PG simulations explain the observed data better than the ABC-DL. **c**. Same PCA plot as **b** but not including the simulations from ABC-DL result. Models C_29 and C_39 are the ones that show a more similar jSFS to the one produced by the observed data.

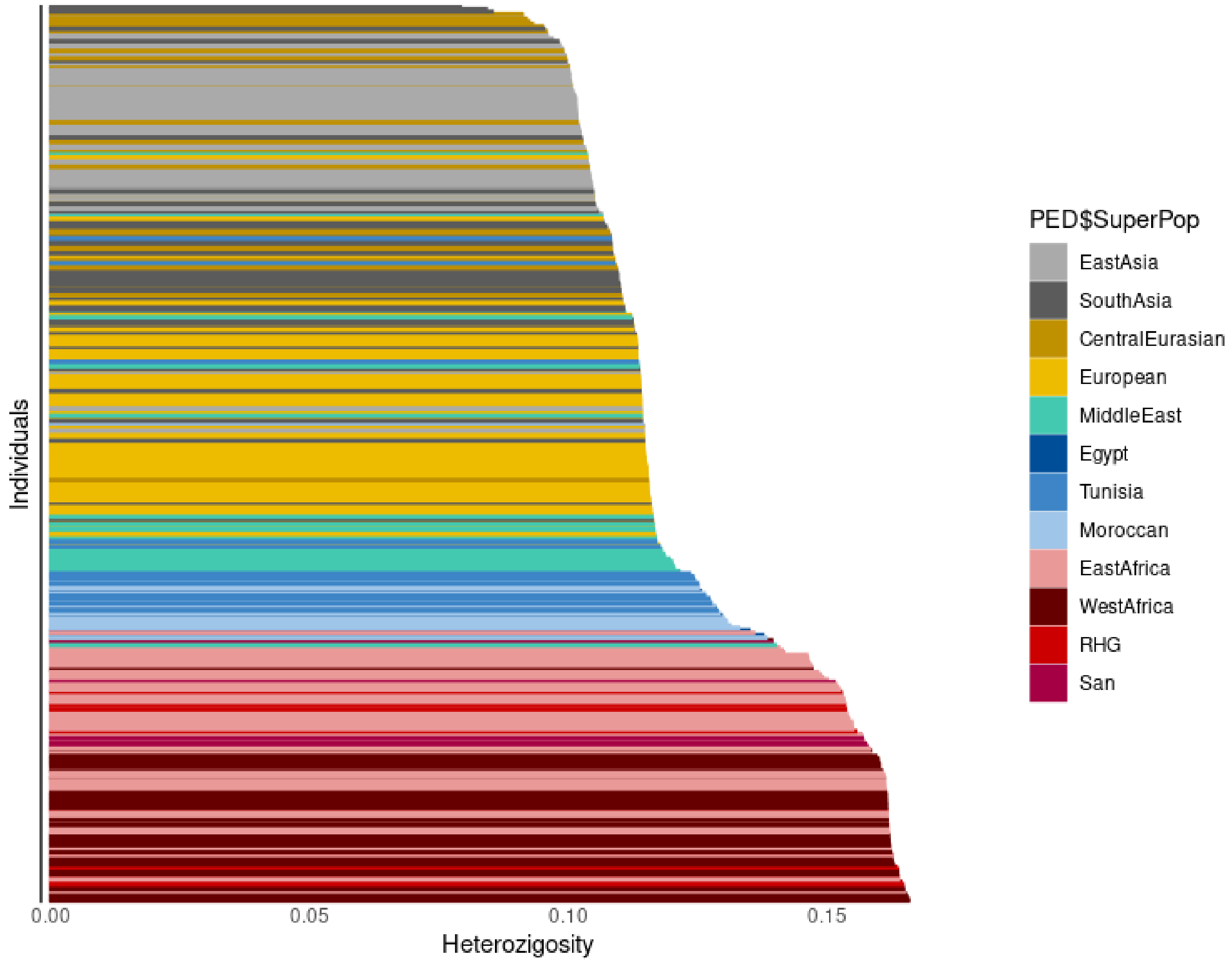

Fig. S 10: Observed heterozygosity per individual compared by superpopulation. Sub-Saharan populations present a higher heterozygosity than Eurasian populations, North African individuals have heterozygosity levels between the sub-Saharan and the Eurasians, probably due to gene flow from sub-Sharan populations to north African individuals.

SampleID	AccesionID	Population	Superpopulation	Reference
CEU01	SAME123392	Centre European Utah	European	1000 Genomes
CEU02	SAMN00801650	Centre European Utah	European	1000 Genomes
CHB01	SAME124093	Han Chinese	East Asia	1000 Genomes
CHB02	SAME123926	Han Chinese	East Asia	1000 Genomes
JHN01	SAMEA3302682	Ju|'hoan North	South Africa San	SGDP, Mallick 2016
JHN02	SAMEA3302894	Ju|'hoan North	South Africa San	SGDP, Mallick 2016
LWK04	SAMN00001054	Luhya	East Africa	1000 Genomes
LWK07	SAMN00001112	Luhya	East Africa	1000 Genomes
QTR01	SAMN03800116	Qatar	Middle East	Fakhro 2016
QTR02	SAMN03800117	Qatar	Middle East	Fakhro 2016
TUN10	SAMEA4969309	Tunisia Arab	North Africa Arab	Serra-Vidal 2019
TUN11	SAMEA4969310	Tunisia Arab	North Africa Arab	Serra-Vidal 2019
TUN12	SAMEA4969294	Tunisia Chenini	North Africa Amazigh	Serra-Vidal 2019
TUN13	SAMEA4969295	Tunisia Chenini	North Africa Amazigh	Serra-Vidal 2019
YRI01	SAME122984	Yoruba	West Africa	1000 Genomes
YRI02	SAME125386	Yoruba	West Africa	1000 Genomes

Table S 7: Samples for the demographic analysis of North Africa

Demographic Event	Prior probability of inclusion in GP4PG
Change in Ne	1
Extintion event of a topodeme	1
Expansion event of a topodeme	1
Change in migration rate	1
Admixture event from one topodeme to another	0.3

Table S 8: Possible demographic events in GP4PG algorithm.

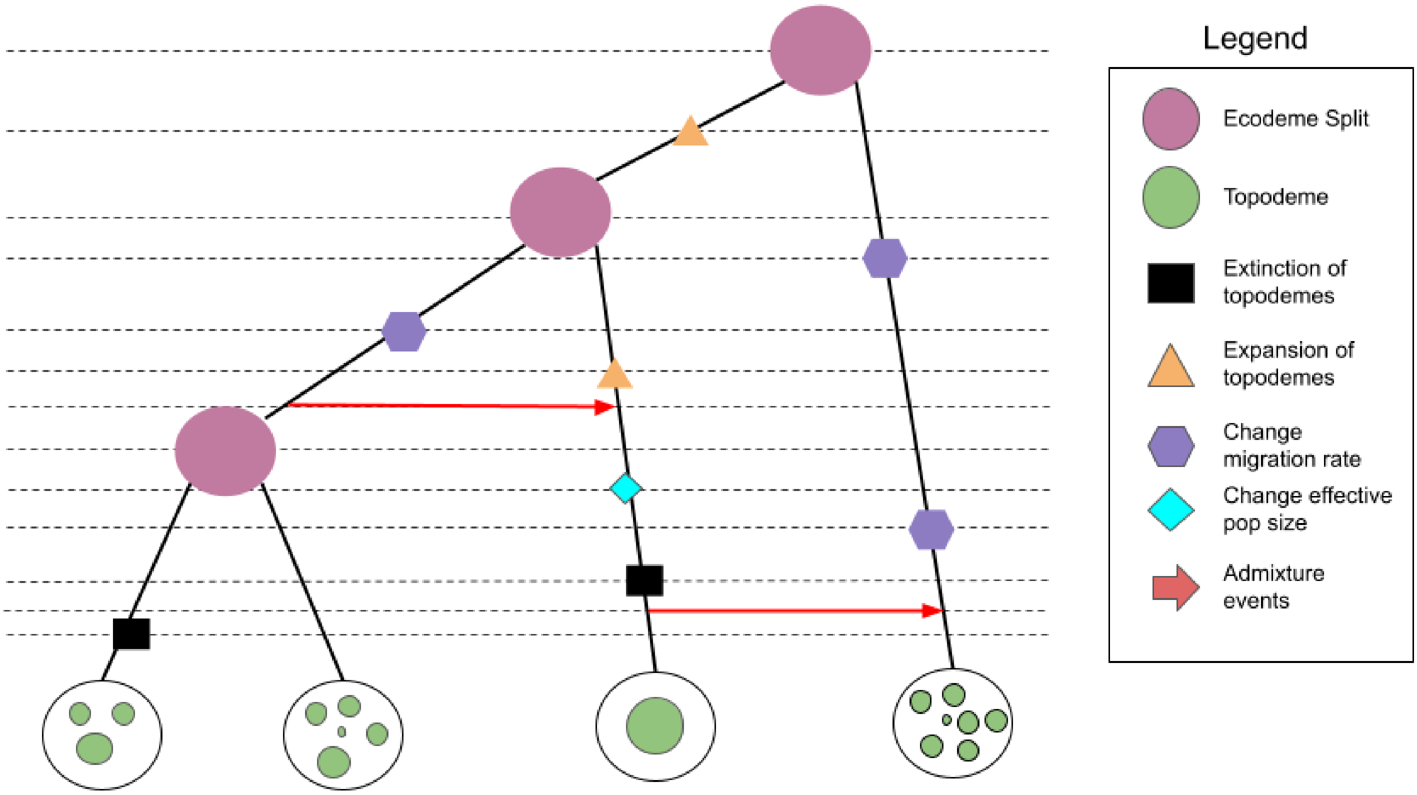

Fig. S 11: A tree-based depiction of the demographic relationships between different ecodemes and topodemes. Each node codes for a demographic event that occurs at a given time (dashed lines).

Parameter	Distribution	A	B	C	D	E	F	G
misclassification	U(0.0,5.0E-4)	X	X	X	X	X	X	X
NeSan	U(10000.0,90000.0)	X	X	X	X	X	X	X
NeYoruba (WAf)	U(10000.0,90000.0)	X	X	X	X	X	X	X
NeLuhya (EAf)	U(10000.0,60000.0)	X	X	X	X	X	X	X
NeTunisia_Chenini (NAfb)	U(1000.0,20000.0)	X	X	X	X	X	X	X
NeTunisia (NAfa)	U(1000.0,40000.0)	X	X	X	X	X	X	X
NeQatar (ME)	U(1000.0,40000.0)	X	X	X	X	X	X	X
NeCEU (EU)	U(1000.0,40000.0)	X	X	X	X	X	X	X
NeHan (EAs)	U(1000.0,40000.0)	X	X	X	X	X	X	X
tME_EU	U(200.0,800.0)	X		X				
NeME_EU	U(1000.0,20000.0)	X		X				
tNAfb_NAfa	U(10.0,150.0)	X	X	X				
NeNAfb_NAfa	U(1000.0,20000.0)	X	X	X				
tME_EU_EAs	U(tME_EU,3000.0)	X						
NeME_EU_EAs	U(1000.0,7000.0)	X						
tWAf_NAfb_NAfa	U(tNAfb_NAfa,600.0)	X						
NeWAf_NAfb_NAfa	U(1000.0,30000.0)	X						
tEAf_ME_EU_EAs	U(tME_EU_EAs,4000.0)	X						
NeEAf_ME_EU_EAs	U(1000.0,10000.0)	X						
tWAf_EAf_EAs_ME_EU_NAfb_NAfa	U(tEAf_ME_EU_EAs,6000.0)	X	X	X	X	X	X	X
NeWAf_EAf_EAs_ME_EU_NAfb_NAfa	U(1000.0,30000.0)	X	X	X	X	X	X	X
tSan_WAf_EAf_EAs_ME_EU_NAfb_NAfa	U(tWAf_EAf_EAs_ME_EU_NAfb_NAfa, 12500.0)	X	X	X	X	X	X	X
NeSan_WAf_EAf_EAs_ME_EU_NAfb_NAfa	U(1000.0,30000.0)	X	X	X	X	X	X	X
tNAfb_NAfa_ME	U(tNAfb_NAfa,600.0)		X					
NeNAfb_NAfa_ME	U(1000.0,15000.0)		X					
tNAfb_NAfa_ME_EU	U(tNAfb_NAfa_ME,800.0)		X					
NeNAfb_NAfa_ME_EU	U(1000.0,20000.0)		X	X	X		X	
tNAfb_NAfa_ME_EU_EAs	U(tNAfb_NAfa_ME_EU,3000.0)		X	X	X		X	

NeNAfb_NAfa_ME_EU_EAs	U(1000.0,7000.0)	X	X	X		X	
tNAfb_NAfa_ME_EU_EAs_EAf	U(tNAfb_NAfa_ME_EU_EAs,4000.0)	X	X	X		X	X
NeNAfb_NAfa_ME_EU_EAs_EAf	U(1000.0,30000.0)	X	X	X		X	X
tNAfb_NAfa_ME_EU	U(tME_EU,1000.0)		X				
tNAfa_ME	U(150.0,600.0)			X	X	X	X
NeNAfa_ME	U(1000.0,20000.0)			X	X	X	X
tNAfa_ME_EU	U(tNAfa_ME,800.0)			X	X		X
NeNAfa_ME_EU	U(1000.0,20000.0)			X	X		X
tNAfb_NAfa_ME_EU	U(tNAfa_ME_EU,1000.0)			X			
tNAfa_ME_EU_EAs	U(tNAfa_ME_EU,1000.0)				X		
NeNAfa_ME_EU_EAs	U(1000.0,7000.0)				X		X
tWAf_NAfb	U(150.0,600.0)				X		
NeWAf_NAfb	U(1000.0,30000.0)				X		
tNAfa_ME_EU_EAs_EAf	U(tNAfa_ME_EU_EAs,4000.0)				X		
NeNAfa_ME_EU_EAs_EAf	U(1000.0,30000.0)				X		
tNAfb_NAfa_ME	U(tNAfb_NAfa,800.0)					X	
NeNAfb_NAfa_ME	U(1000.0,20000.0)					X	
tNAfb_NAfa_ME_EU	U(tNAfb_NAfa_ME,1000.0)					X	
tNAfa_ME_EU_EAs	U(tNAfa_ME_EU,3000.0)						X
tEAf_NAfb	U(150.0,1000.0)						X
NeEAf_NAfb	U(1000.0,30000.0)						X

Table S 9: Parameters and prior distributions of the seven considered models in Fig. S4

Parameter	Distribution	D	D 2	D 3	D 4	D 5
misclassification	U(0.0, 5.0E-4)	X	X	X	X	X
NeSan	U(10000.0,90000.0)	X	X	X	X	X
NeYoruba	U(10000.0,90000.0)	X	X	X	X	X
NeLuhya	U(10000.0,60000.0)	X	X	X	X	X
NeTunisia_Chenini	U(1000.0,20000.0)	X	X	X	X	X
NeTunisia	U(1000.0,40000.0)	X	X	X	X	X
NeQatar	U(1000.0,40000.0)	X	X	X	X	X
NeCEU	U(1000.0,40000.0)	X	X	X	X	X
NeHan	U(1000.0,40000.0)	X	X	X	X	X
NeBasal_Eurasian_ghost (BEi)	U(1000.0,20000.0)			X	X	X
NeAfrican_ghost (Xa)	U(1000.0,20000.0)				X	X
migrationNAfa_NAfb	U(0.0,5.0E-4)		X	X	X	
migrationNAfb_NAfa	U(0.0,5.0E-4)		X	X	X	
migrationME_NAfb	U(0.0,5.0E-4)		X	X	X	
migrationNAfb_ME	U(0.0,5.0E-4)		X	X	X	
migrationME_NAfa	U(0.0,5.0E-4)		X	X	X	
migrationNAfa_ME	U(0.0,5.0E-4)		X	X	X	
migrationEU_ME	U(0.0,5.0E-4)		X	X	X	
migrationEU_NAfb	U(0.0,5.0E-4)		X	X	X	
migrationNAfb_EU	U(0.0,5.0E-4)		X	X	X	
migrationEU_NAfa	U(0.0,5.0E-4)		X	X	X	
migrationNAfa_EU	U(0.0,5.0E-4)		X	X	X	
migrationEAs_EU	U(0.0,5.0E-4)		X	X	X	
migrationEU_EAs	U(0.0,5.0E-4)		X	X	X	
migrationEAf_NAfb	U(0.0,5.0E-4)		X	X	X	
migrationNAfb_EAf	U(0.0,5.0E-4)		X	X	X	
migrationEAf_NAfa	U(0.0,5.0E-4)		X	X	X	
migrationNAfa_EAf	U(0.0,5.0E-4)		X	X	X	
migrationEAf_ME	U(0.0,5.0E-4)		X	X	X	

migrationME_EAf	U(0.0,5.0E-4)		X	X	X	
migrationEAf_WAf	U(0.0,5.0E-4)		X	X	X	
migrationWAf_EAf	U(0.0,5.0E-4)		X	X	X	
migrationWAf_NAfb	U(0.0,5.0E-4)		X	X	X	
migrationNAfb_WAf	U(0.0,5.0E-4)		X	X	X	
migrationWAf_NAfa	U(0.0,5.0E-4)		X	X	X	
migrationNAfa_WAf	U(0.0,5.0E-4)		X	X	X	
migrationSan_to_WAf	U(0.0,5.0E-4)		X	X	X	
migrationSan_to_EAf	U(0.0,5.0E-4)		X	X	X	
tNAfa_ME	U(150.0,600.0)	X	X	X	X	X
NeNAfa_ME	U(1000.0,20000.0)	X	X	X	X	X
tNAfa_ME_EU	U(tNAfa_ME,800.0)	X	X	X	X	X
NeNAfa_ME_EU	U(1000.0,20000.0)	X	X	X	X	X
tNAfb_NAfa_ME_EU	U(tNAfa_ME_EU,1000.0)	X	X	X	X	X
tNAfb_NAfa_ME_EU_EAs	U(tNAfb_NAfa_ME_EU,20000.0)	X	X	X	X	X
NeNAfb_NAfa_ME_EU_EAs	U(1000.0,7000.0)	X	X	X	X	X
tNAfb_NAfa_ME_EU_EAs_EAf	U(tNAfb_NAfa_ME_EU_EAs,4000.0)	X	X	X	X	X
NeNAfb_NAfa_ME_EU_EAs_EAf	U(1000.0,30000.0)	X	X	X	X	X
tWAf_EAf_EAs_ME_EU_NAfb_NAfa	U(tEAf_ME_EU_EAs,6000.0)	X	X	X	X	X
NeWAf_EAf_EAs_ME_EU_NAfb_NAfa	U(1000.0,30000.0)	X	X	X	X	X
tSan_WAf_EAf_EAs_ME_EU_NAfb_NAfa	U(tWAf_EAf_EAs_ME_EU_NAfb_NAfa, 12500.0)	X	X	X	X	X
NeSan_WAf_EAf_EAs_ME_EU_NAfb_NAfa	U(1000.0,30000.0)	X	X	X	X	X
tSan_WAf_EAf_EAs_ME_EU_NAfb_NAfa_Xa	U(tNAfb_NAfa_ME_EU_BEi_EAs_WAf_San, 14000.0)				X	X
NeSan_WAf_EAf_EAs_ME_EU_NAfb_NAfa_Xa	U(1000.0,30000.0)				X	X
tNAfb_NAfa_ME_EU_BEi	U(tNAfb_NAfa_ME_EU,2000.0)			X	X	X
NeNAfb_NAfa_ME_EU_BEi	U(1000.0,20000.0)			X	X	X
tAdmxME_NA	U(60.0,tNAfa_ME)		X	X	X	X

admixtureME_NA	U(0.001,0.20)	X	X	X	X
tAdmxEU_NA	U(20.0,tNAfa_ME)			X	X
admixtureEU_NA	U(0.001,0.10)			X	X
tAdmxEU_NAb	U(20.0,tNAfa_ME)			X	X
admixtureEU_NAb	U(0.001,0.10)			X	X
tAdmxME_NAb	U(60.0,tNAfa_ME)			X	X
admixtureME_NAb	U(0.001,0.20)			X	X
tAdmxMENA_Amazigh	U(tNAfa_ME,tNAfa_ME_EU)	X	X	X	X
admixtureMENA_Amazigh	U(0.001,0.10)	X	X	X	X
tAdmxWAf_Amazigh	U(10.0,tNAfb_NAfa_ME_EU)	X	X	X	X
admixtureWAf_Amazigh	U(0.001,0.10)	X	X	X	X
tAdmxWAf_Arab	U(10.0,tNAfa_ME)	X	X	X	X
admixtureWAf_Arab	U(0.001,0.10)	X	X	X	X
tAdmxEAf_Amazigh	U(10.0,tNAfb_NAfa_ME_EU)	X	X	X	X
admixtureEAf_Amazigh	U(0.001,0.10)	X	X	X	X
tAdmxEAf_Arab	U(10.0,tNAfa_ME)	X	X	X	X
admixtureEAf_Arab	U(0.001,0.10)	X	X	X	X
tAdmxBEi_MENAU	U(tNAfa_ME_EU, tNAfb_NAfa_ME_EU)		X	X	X
admixtureBEi_MENAU	U(0.001,0.20)		X	X	X
tAdmxBEi_AMENAU	U(tNAfb_NAfa_ME_EU, tNAfb_NAfa_ME_EU_BEi)		X	X	X
admixtureBEi_AMENAU	U(0.001,0.20)		X	X	X
tAdmxXa_San	U(700.0,tNAfb_NAfa_ME_EU_BEi_EAs_WAf_San)			X	X
admixtureXa_San	U(0.001,0.05)			X	X
tAdmxXa_WAf	U(700.0,tNAfb_NAfa_ME_EU_BEi_EAs_WAf)			X	X
admixtureXa_WAf	U(0.001,0.05)			X	X

Table S 10: Parameters and prior distributions of the five considered models in Fig. 2.
