# Supplementary figures and images for: Modelling the demographic history of human North African genomes points to a recent soft split divergence between populations
Source: Genome Biol. 2024 Jul 30;25:201. doi: 10.1186/s13059-024-03341-4 (PMC11290046; doi:10.1186/s13059-024-03341-4)

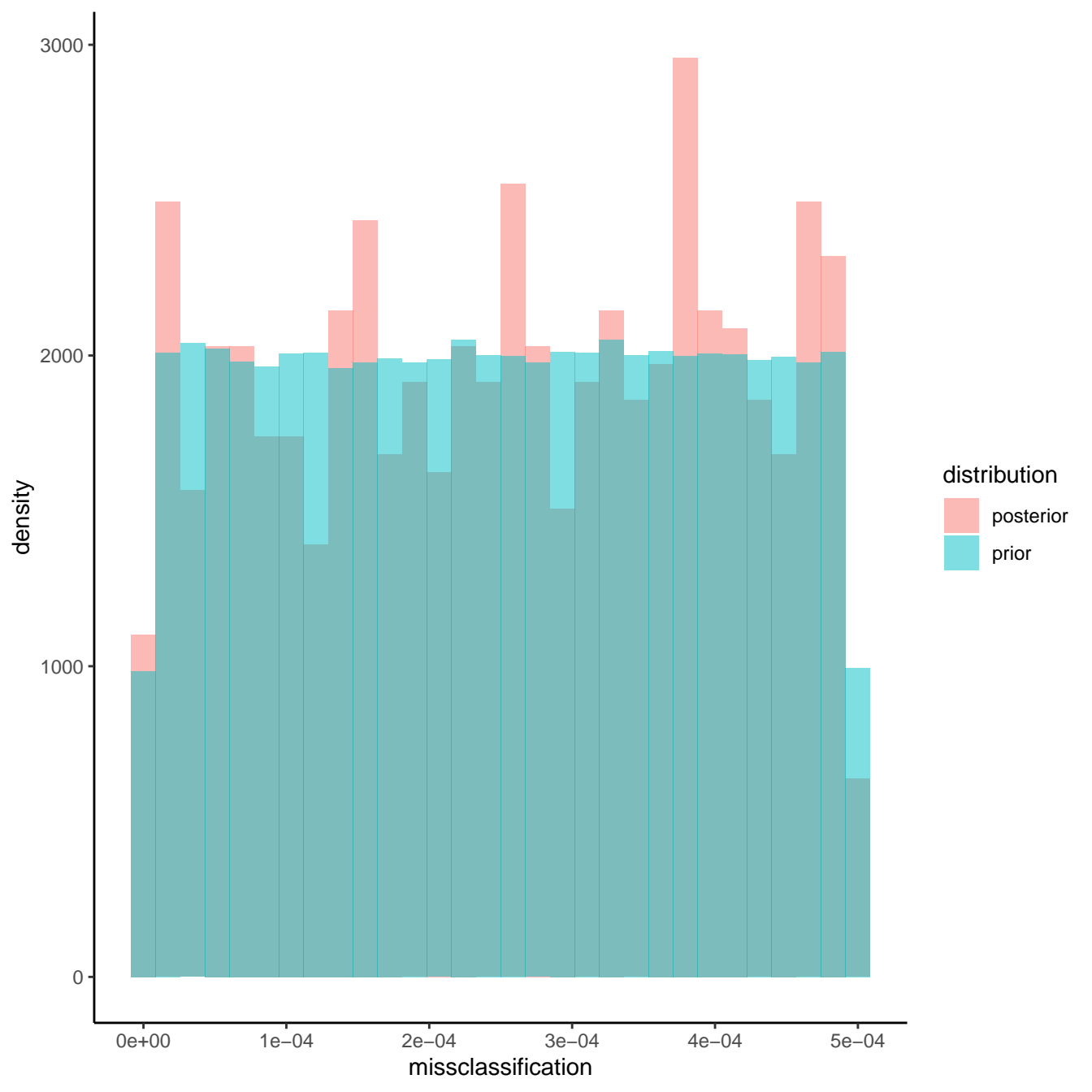

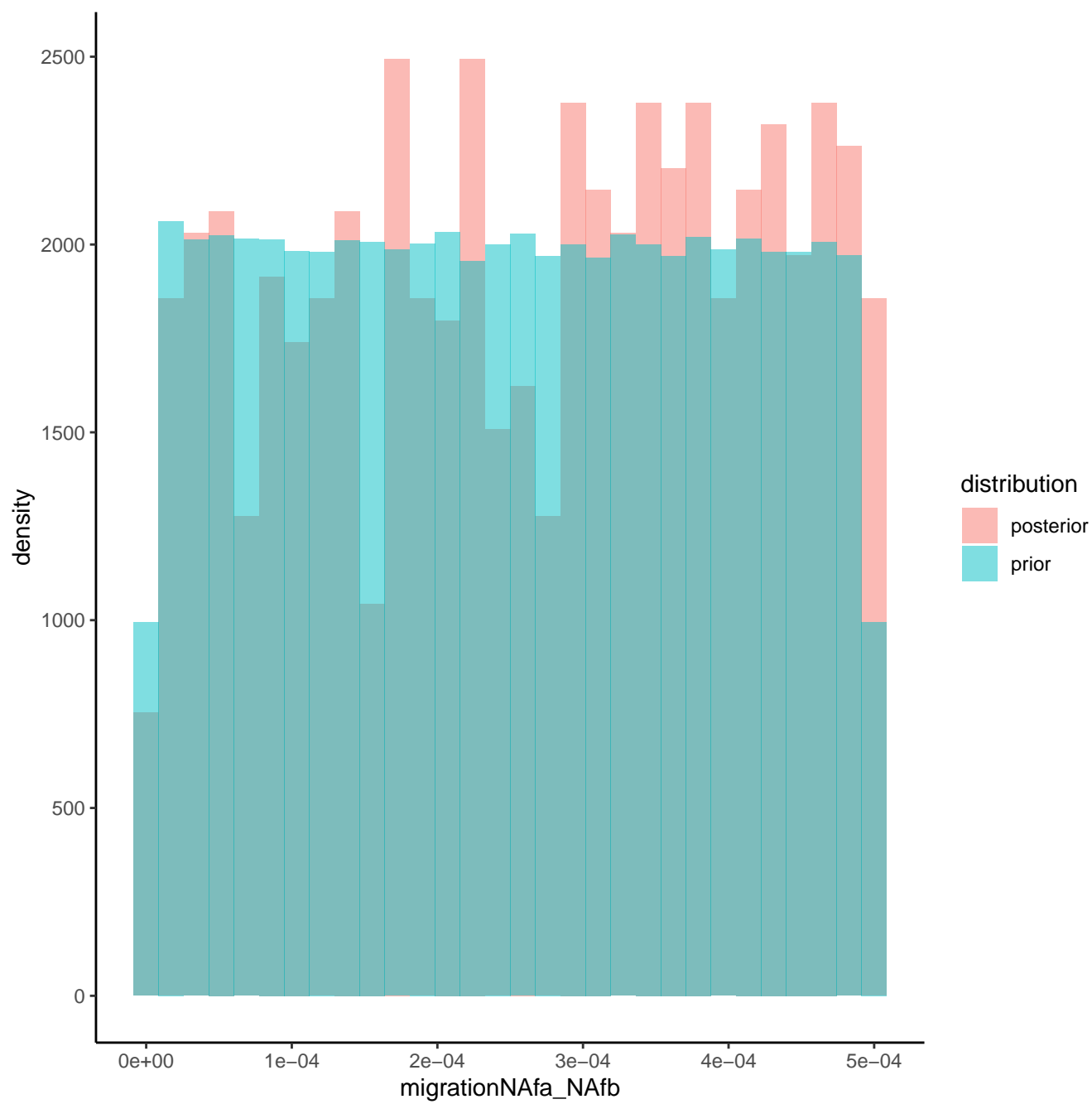

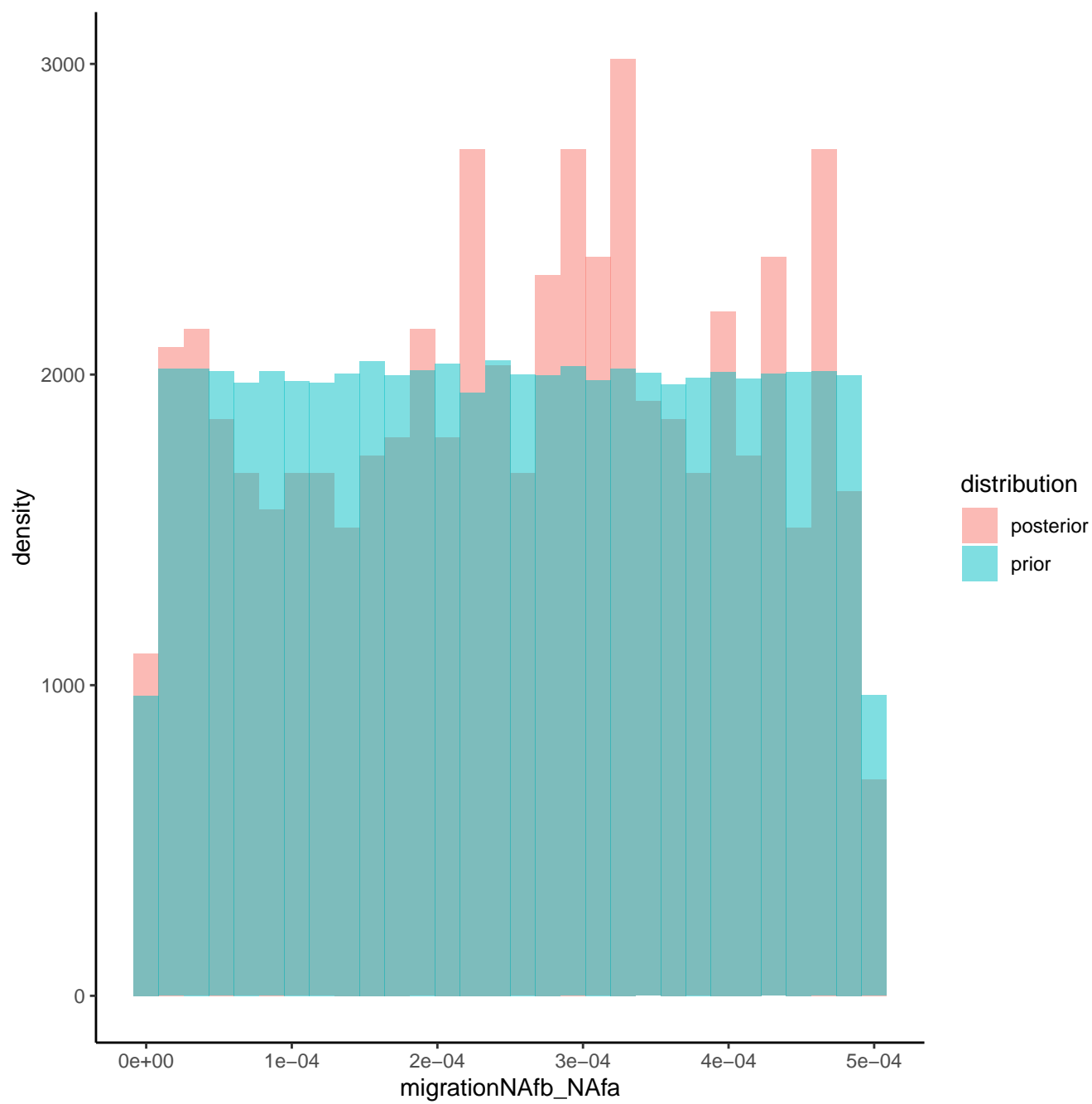

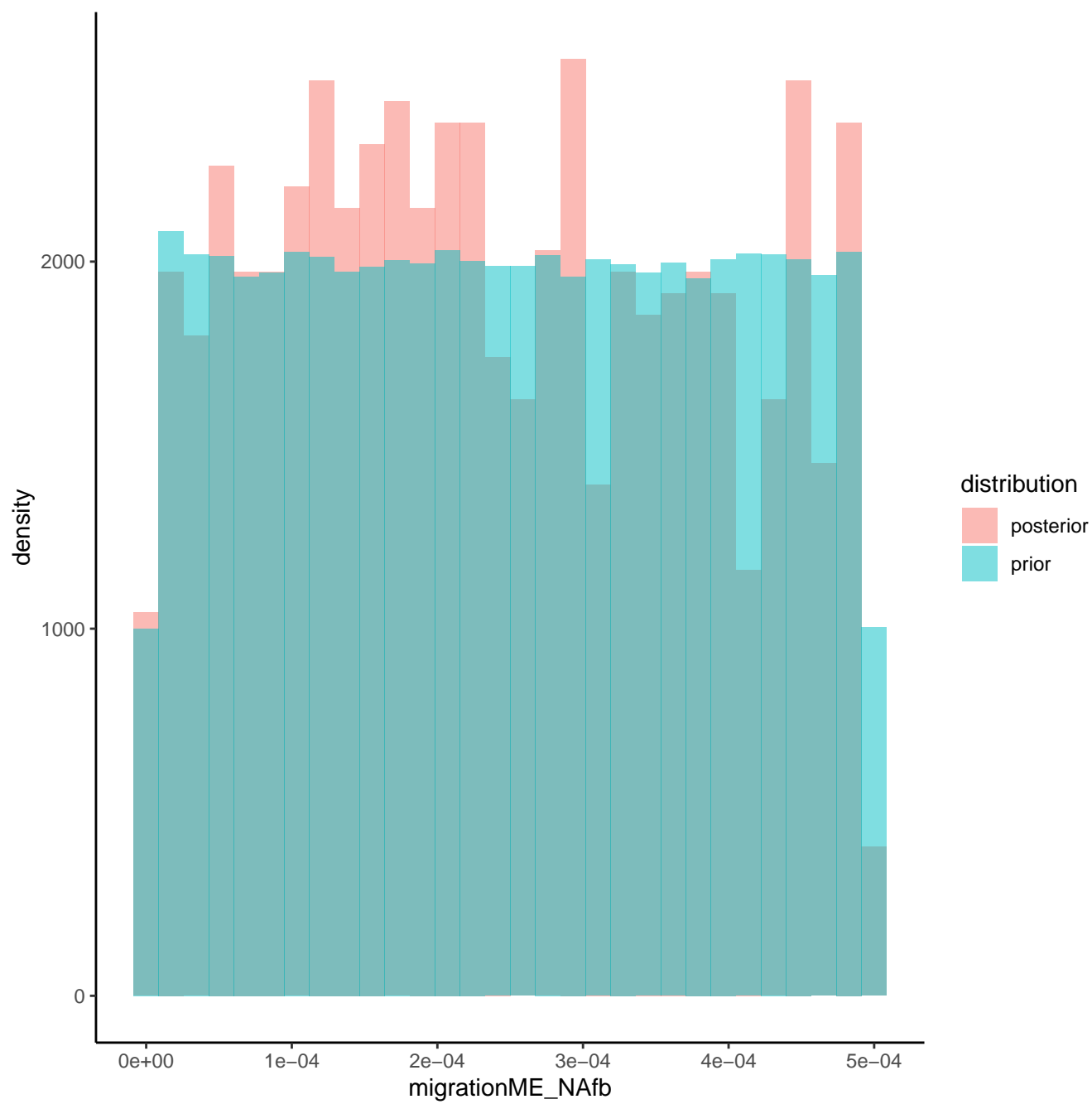

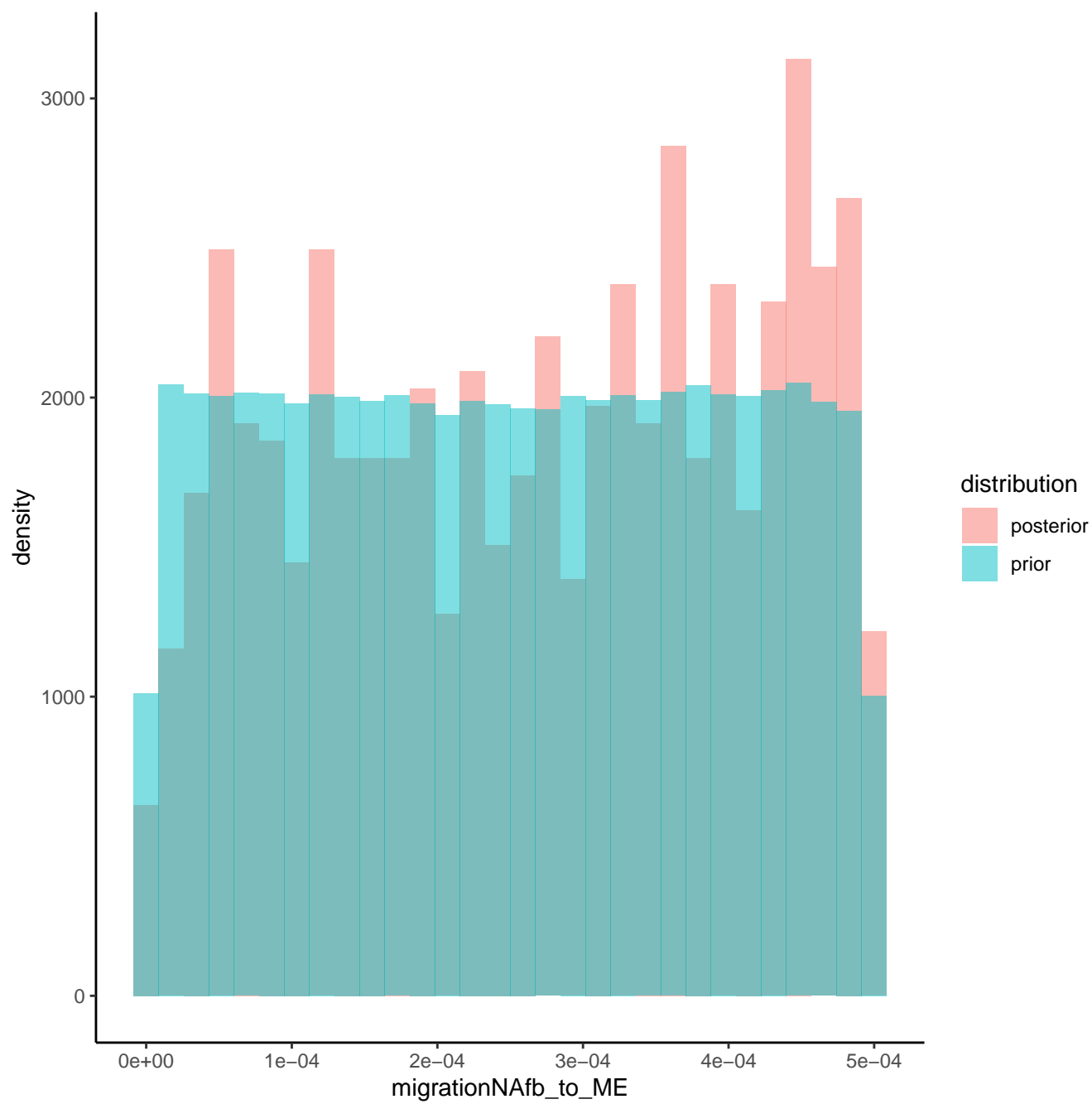

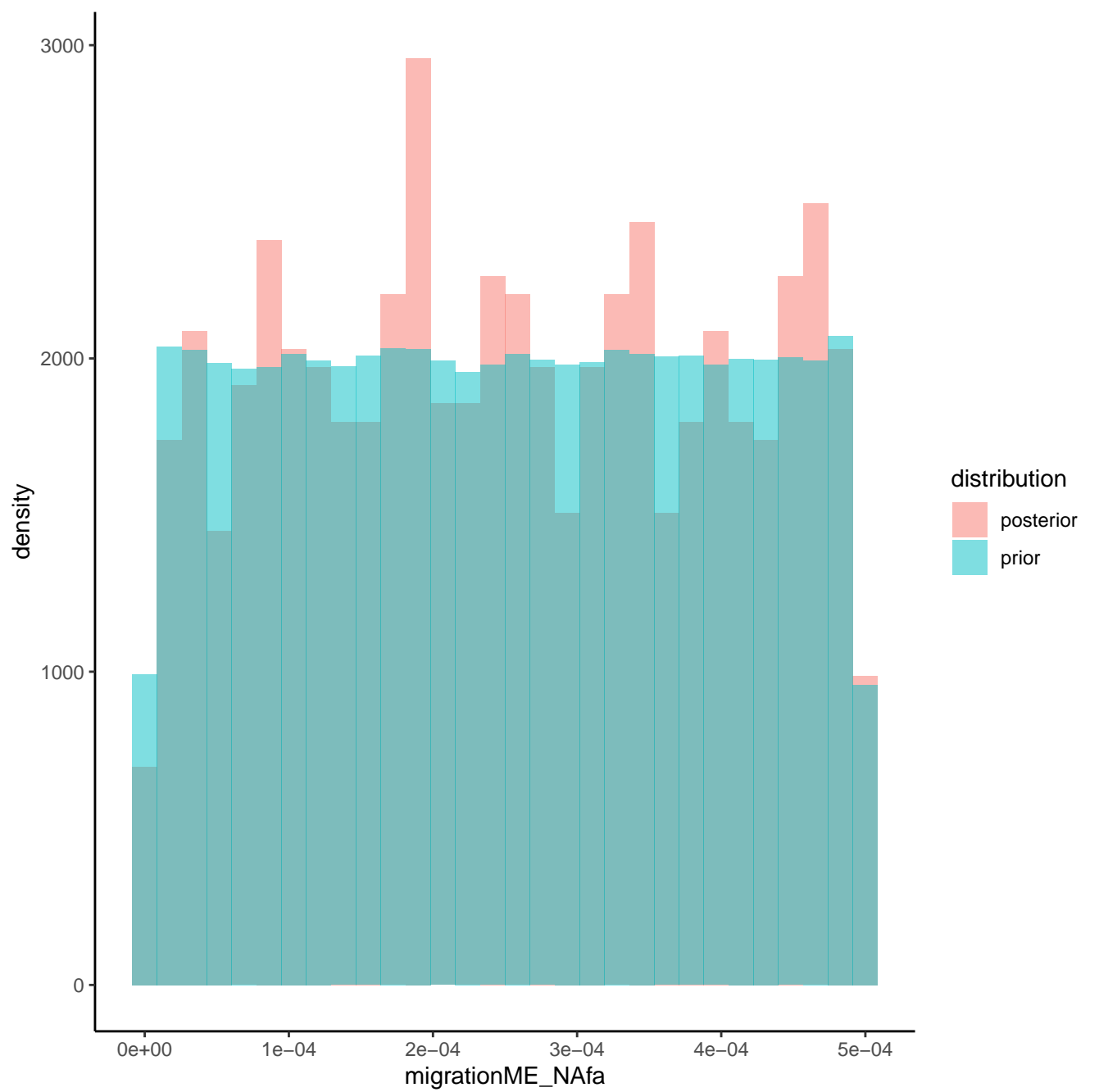

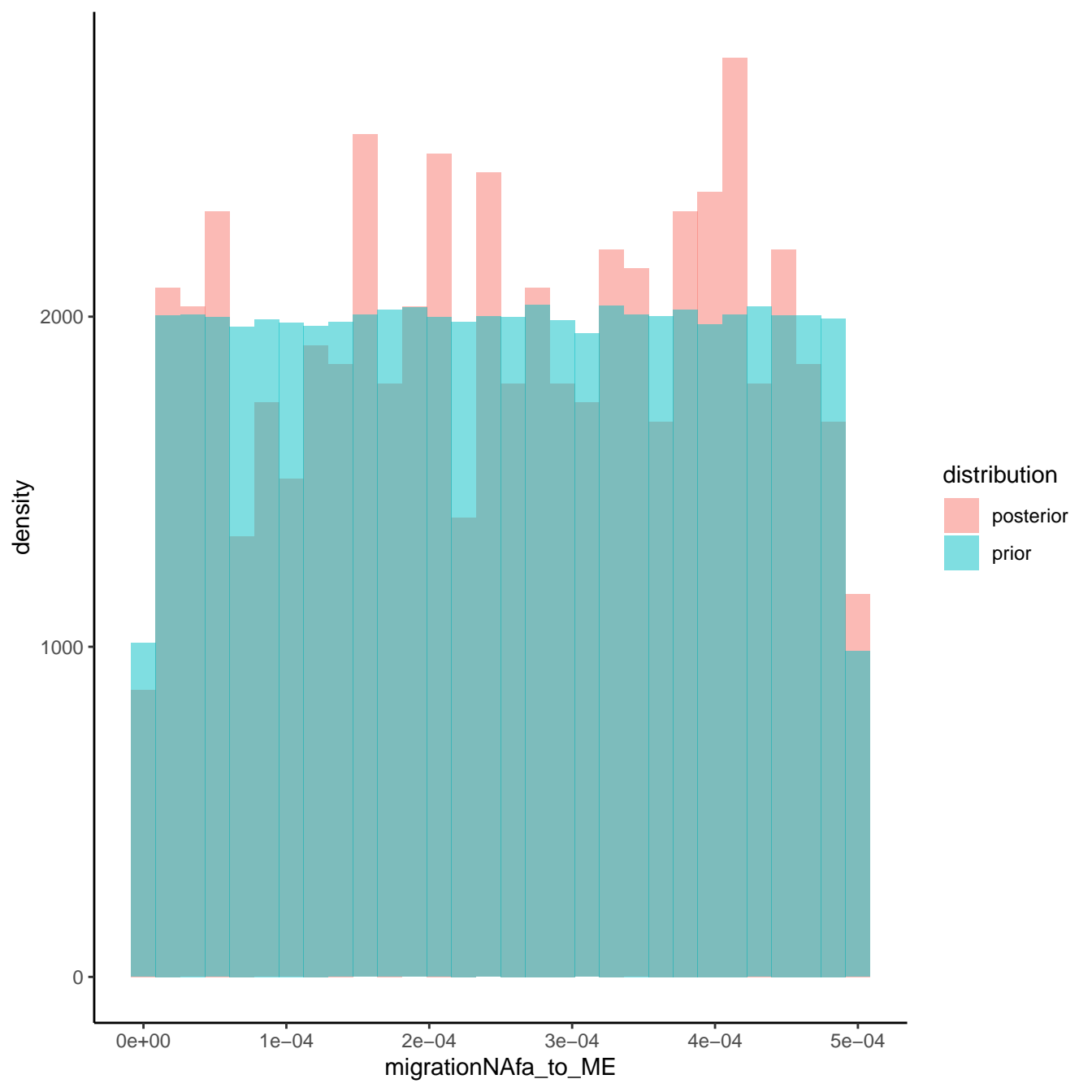

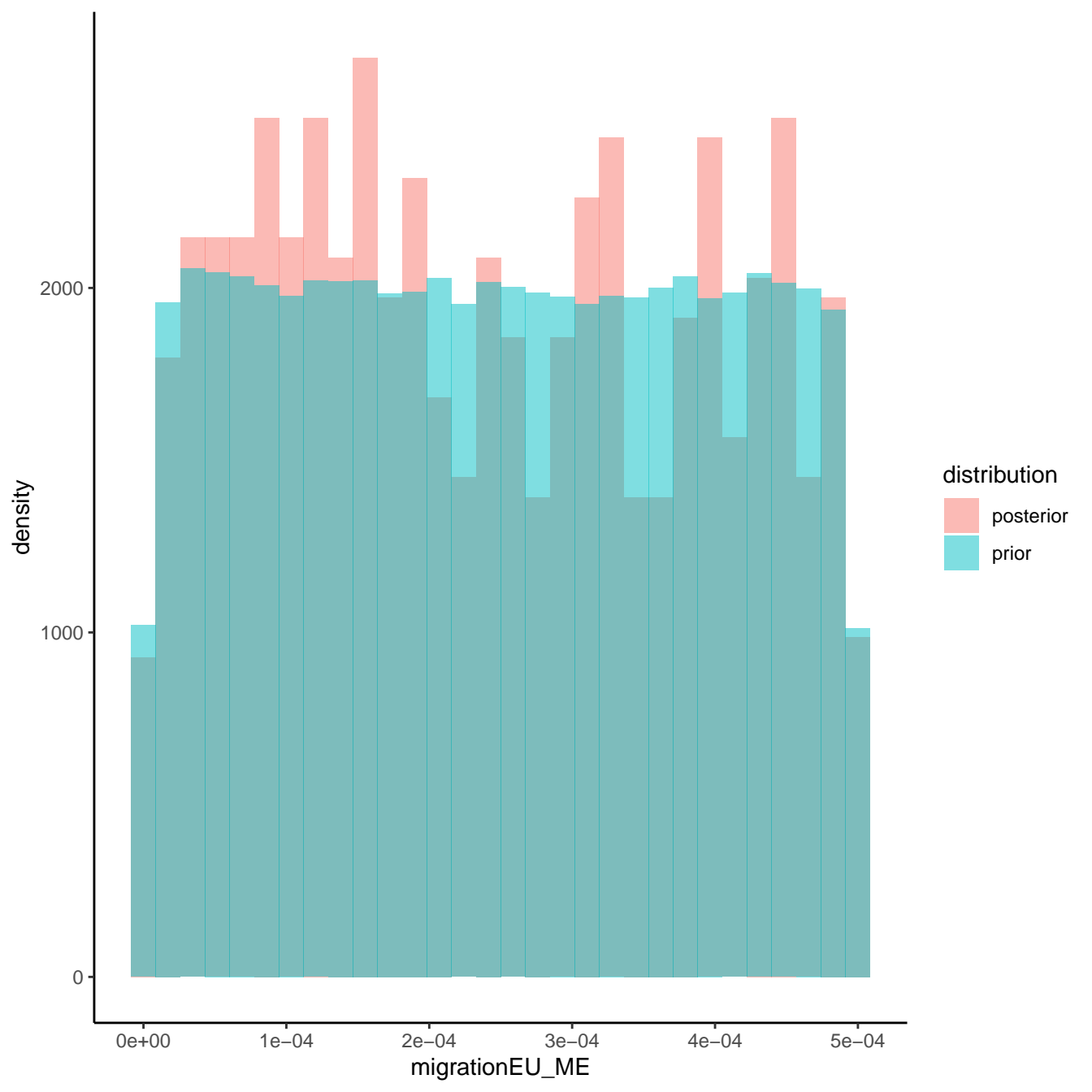

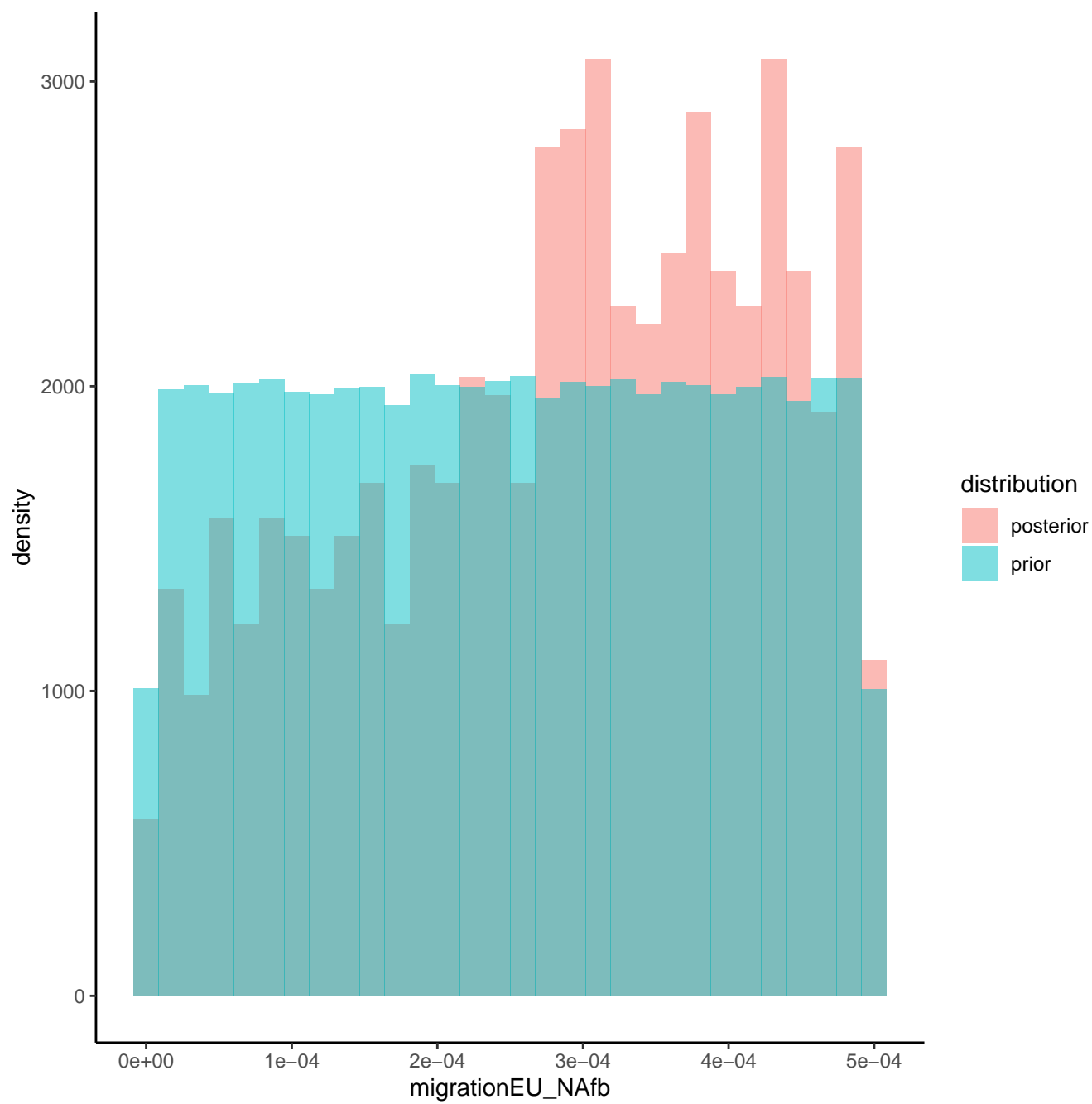

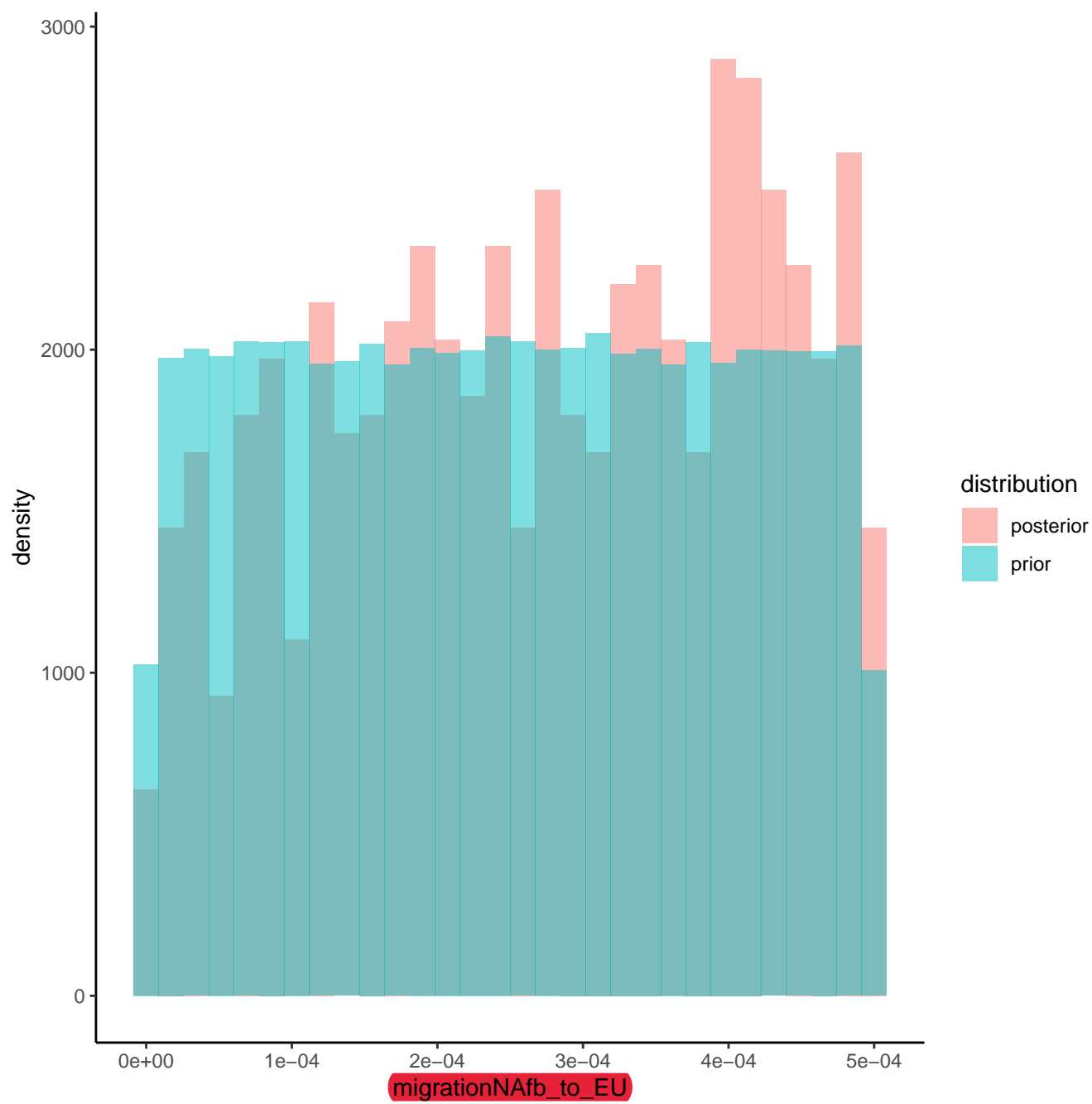

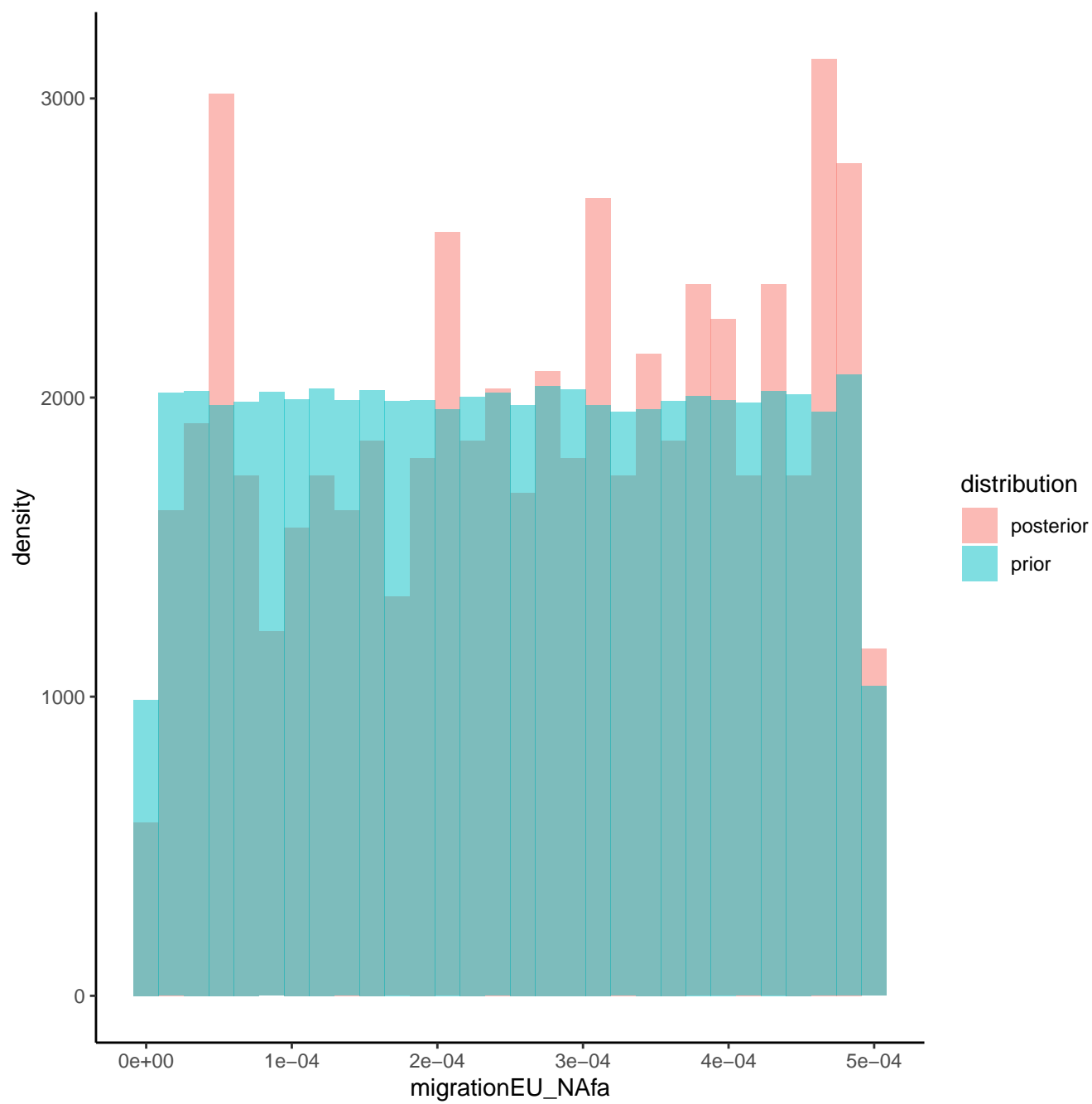

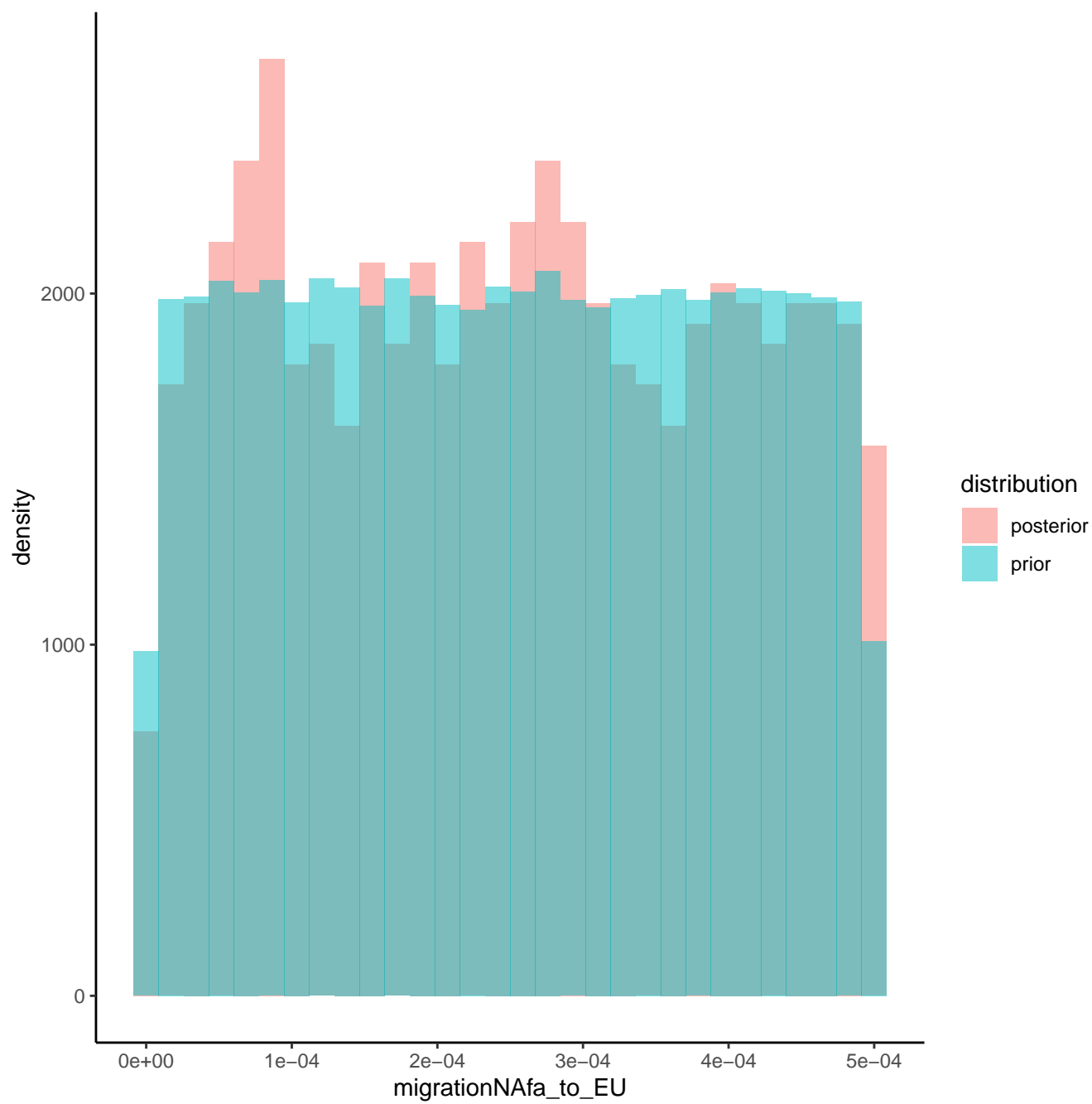

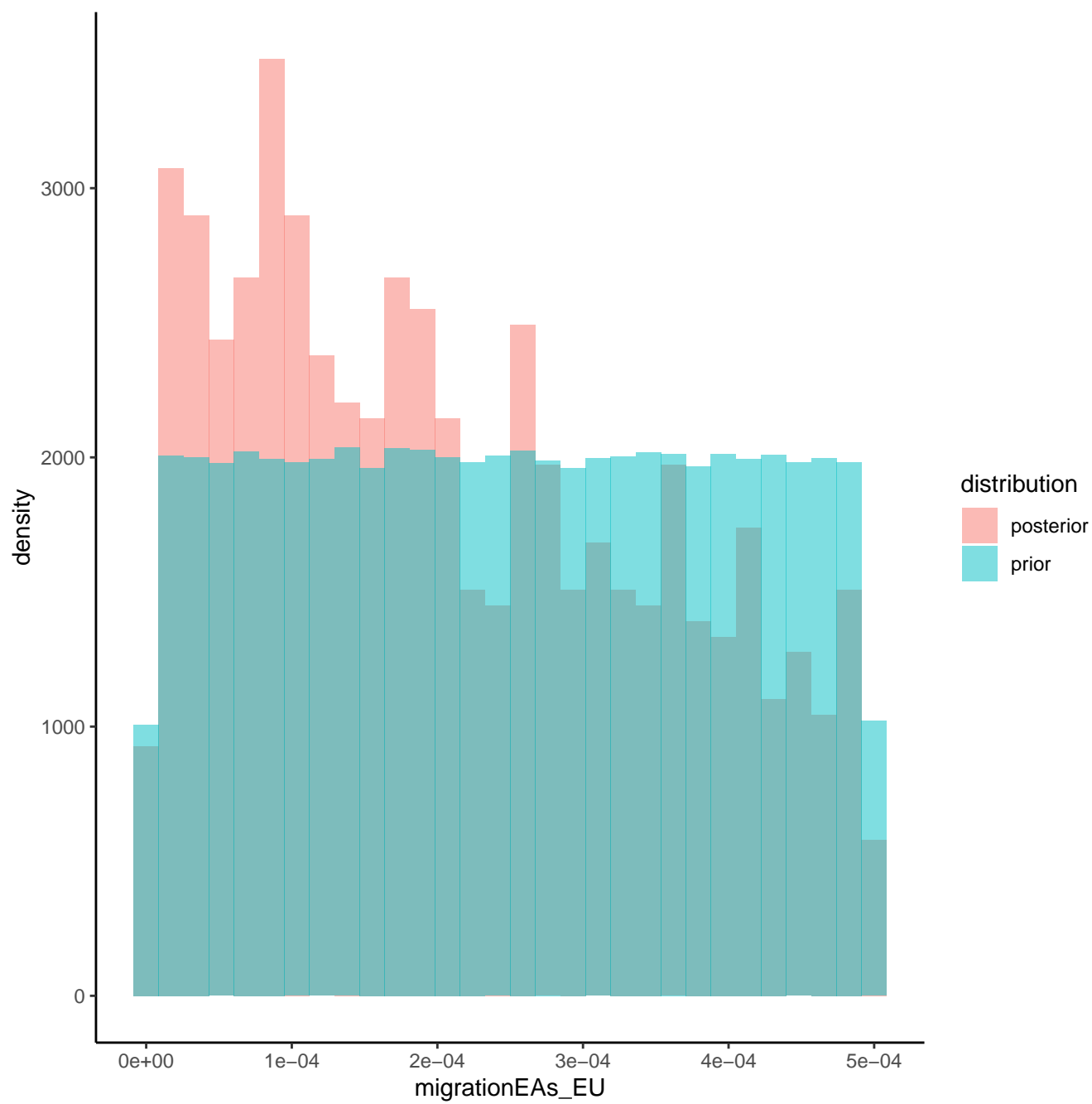

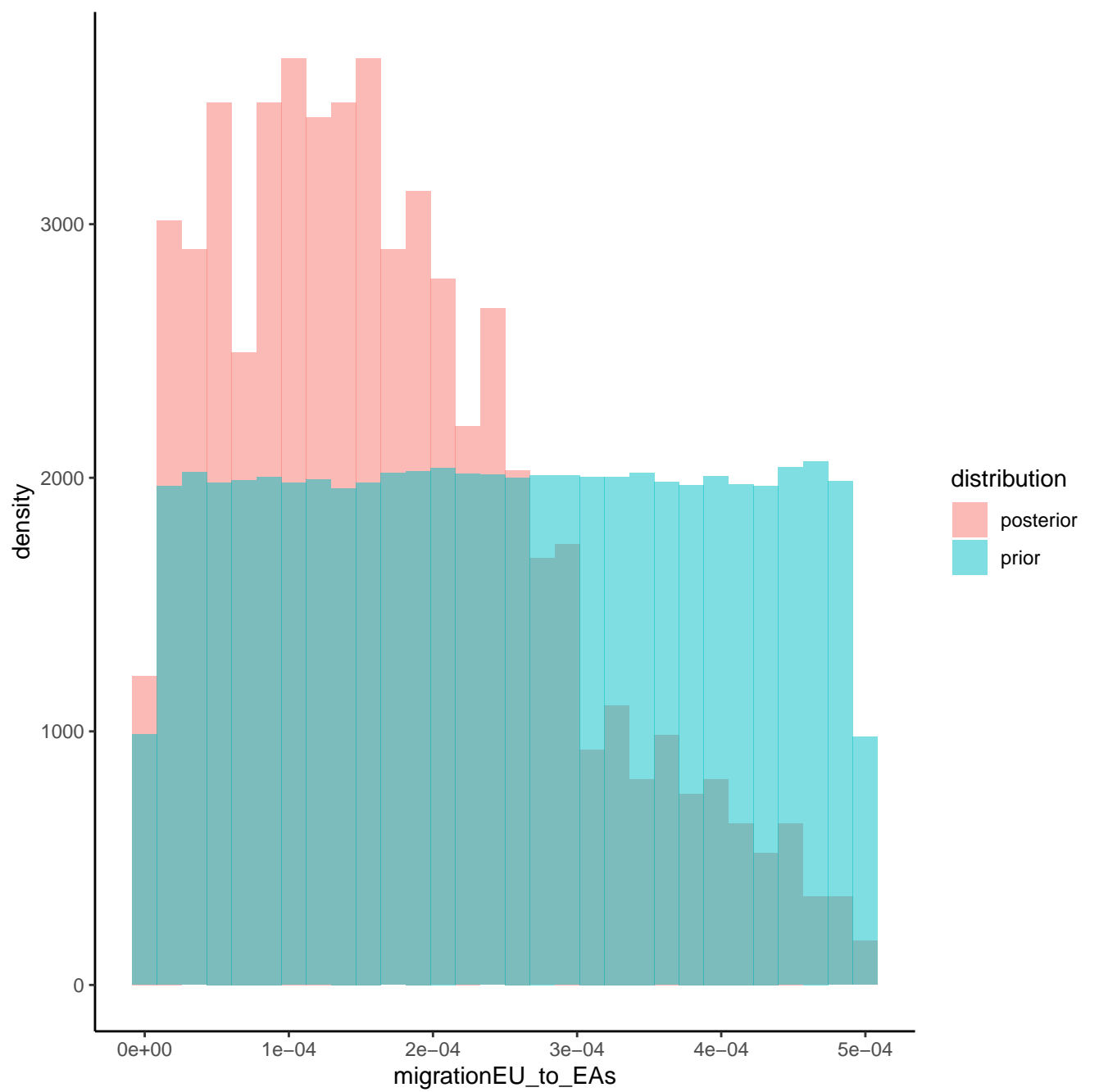

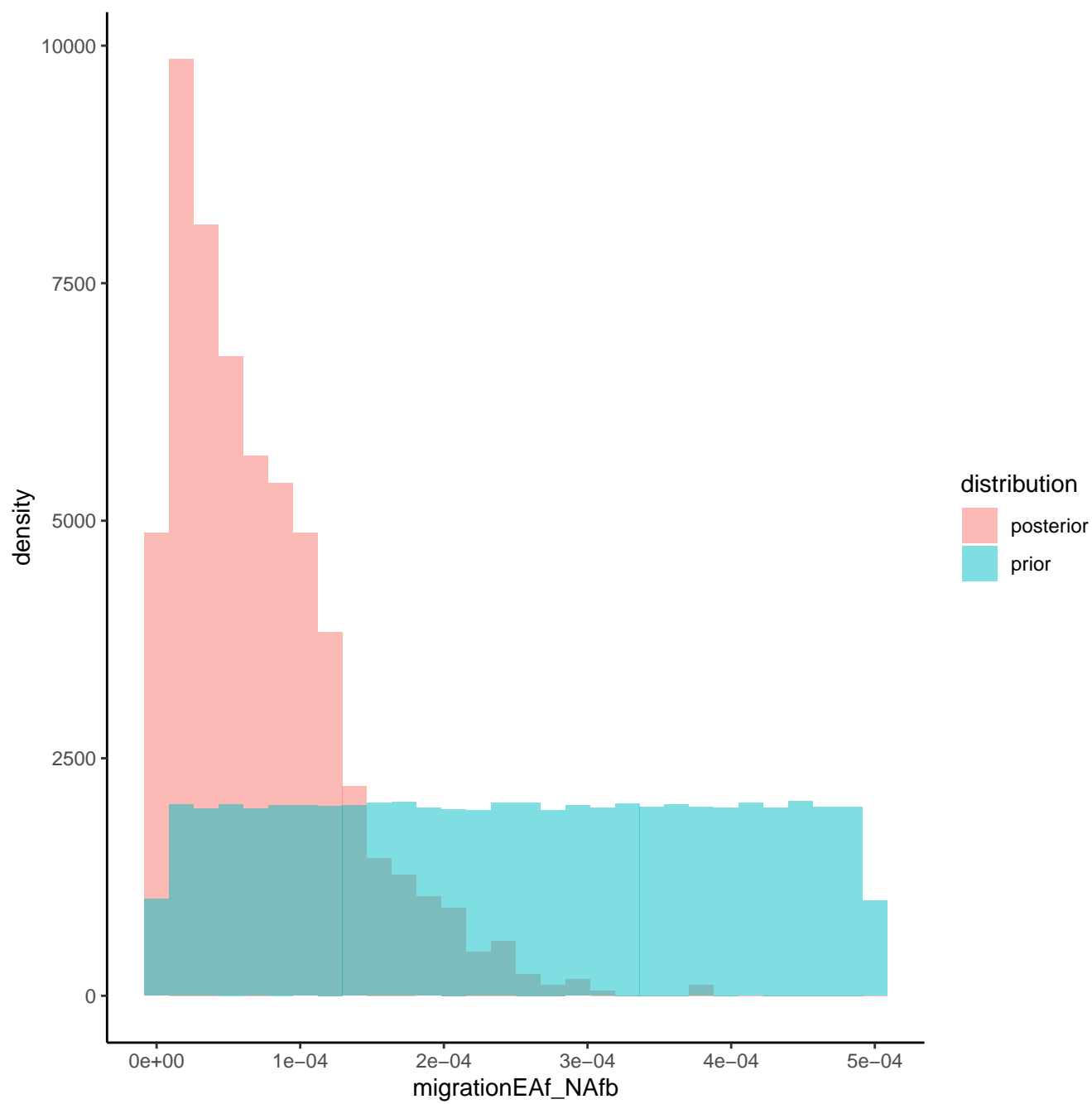

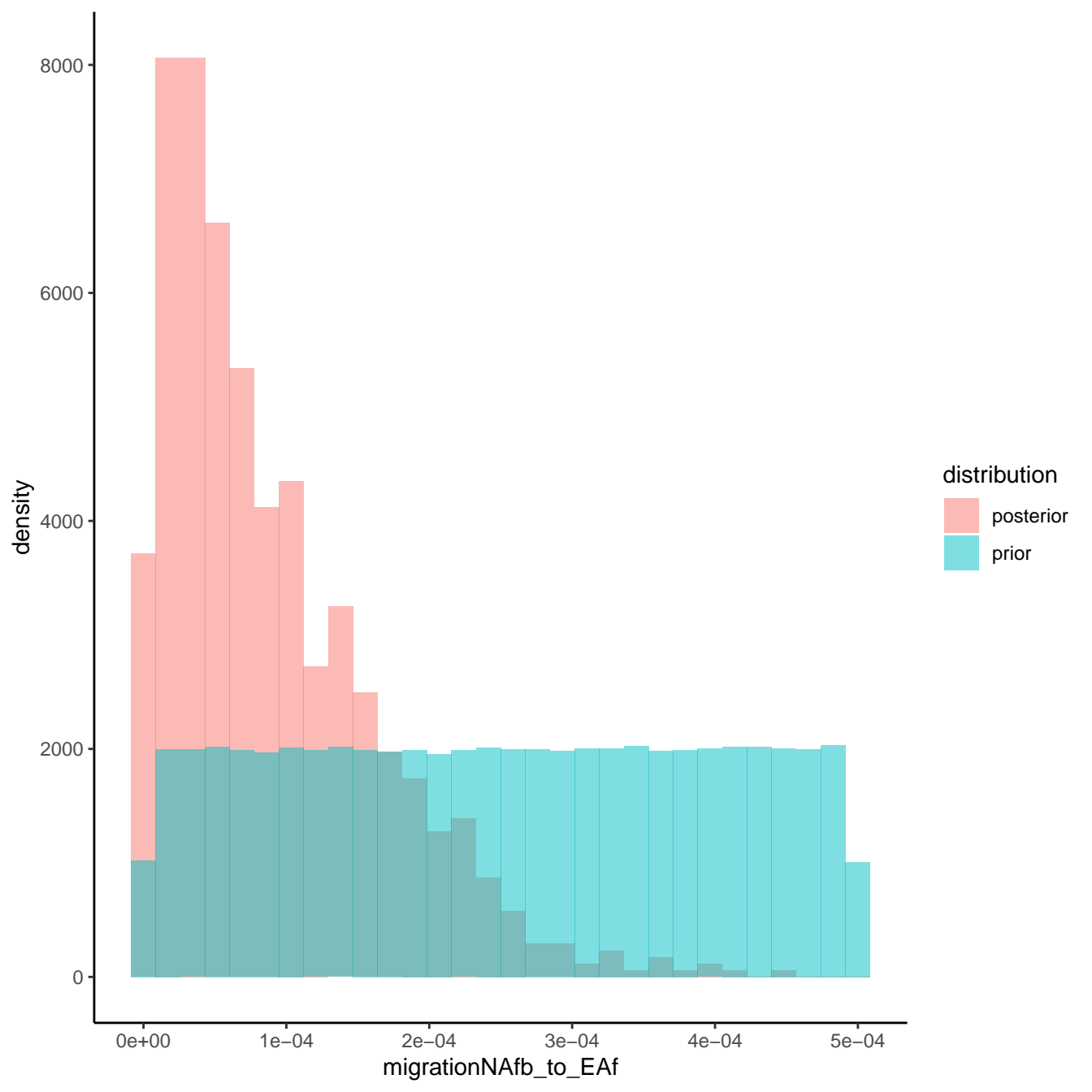

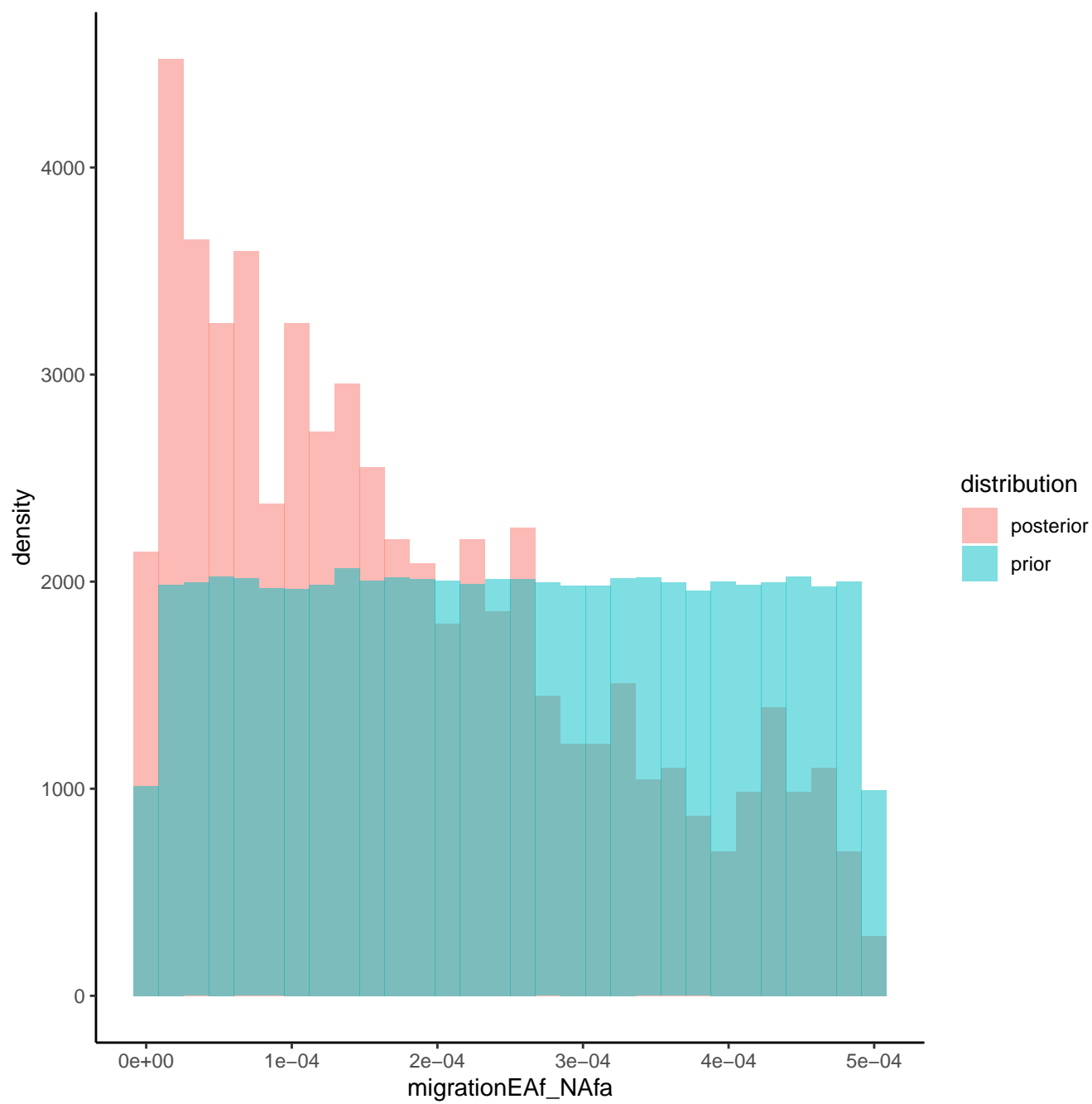

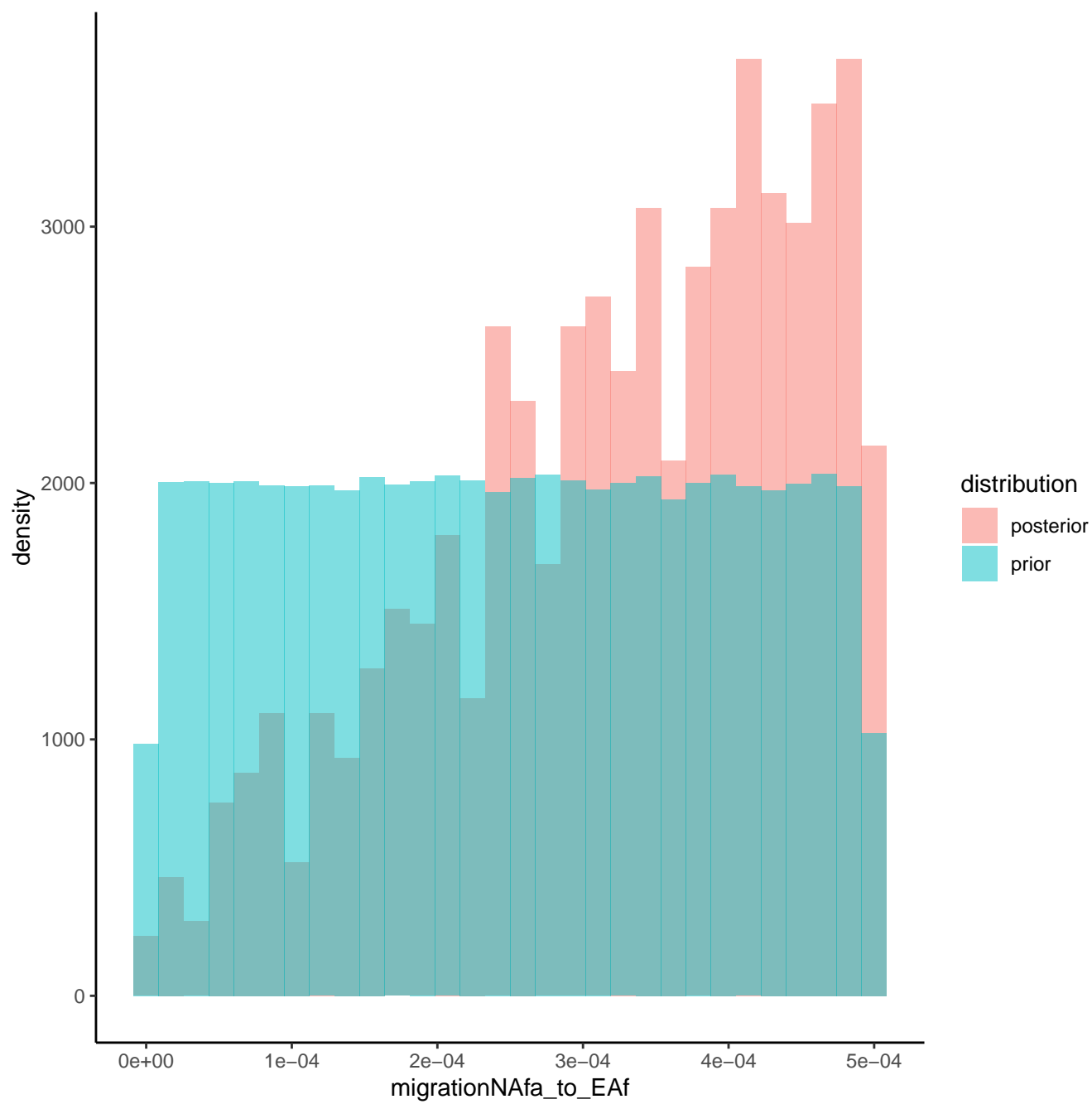

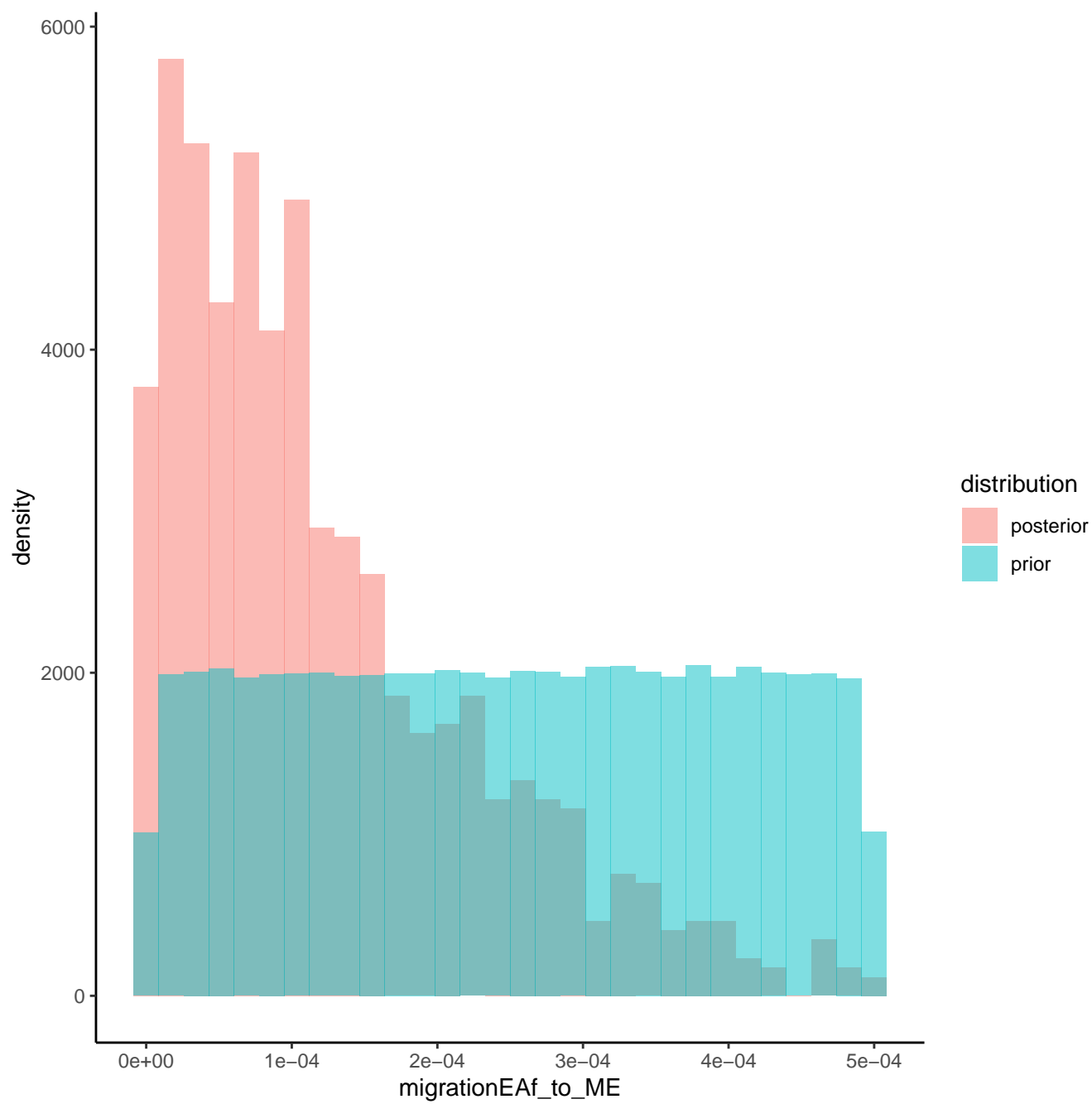

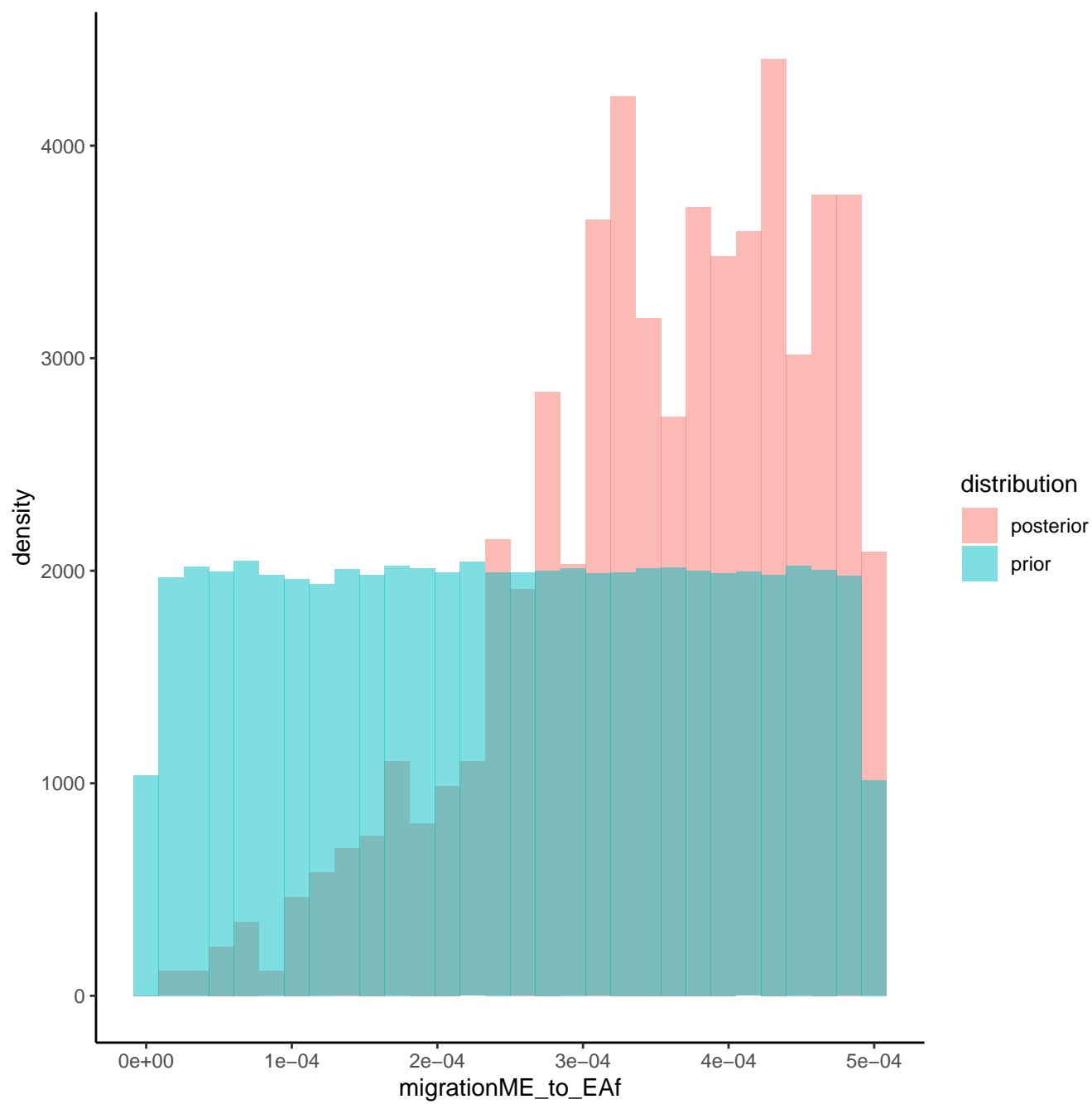

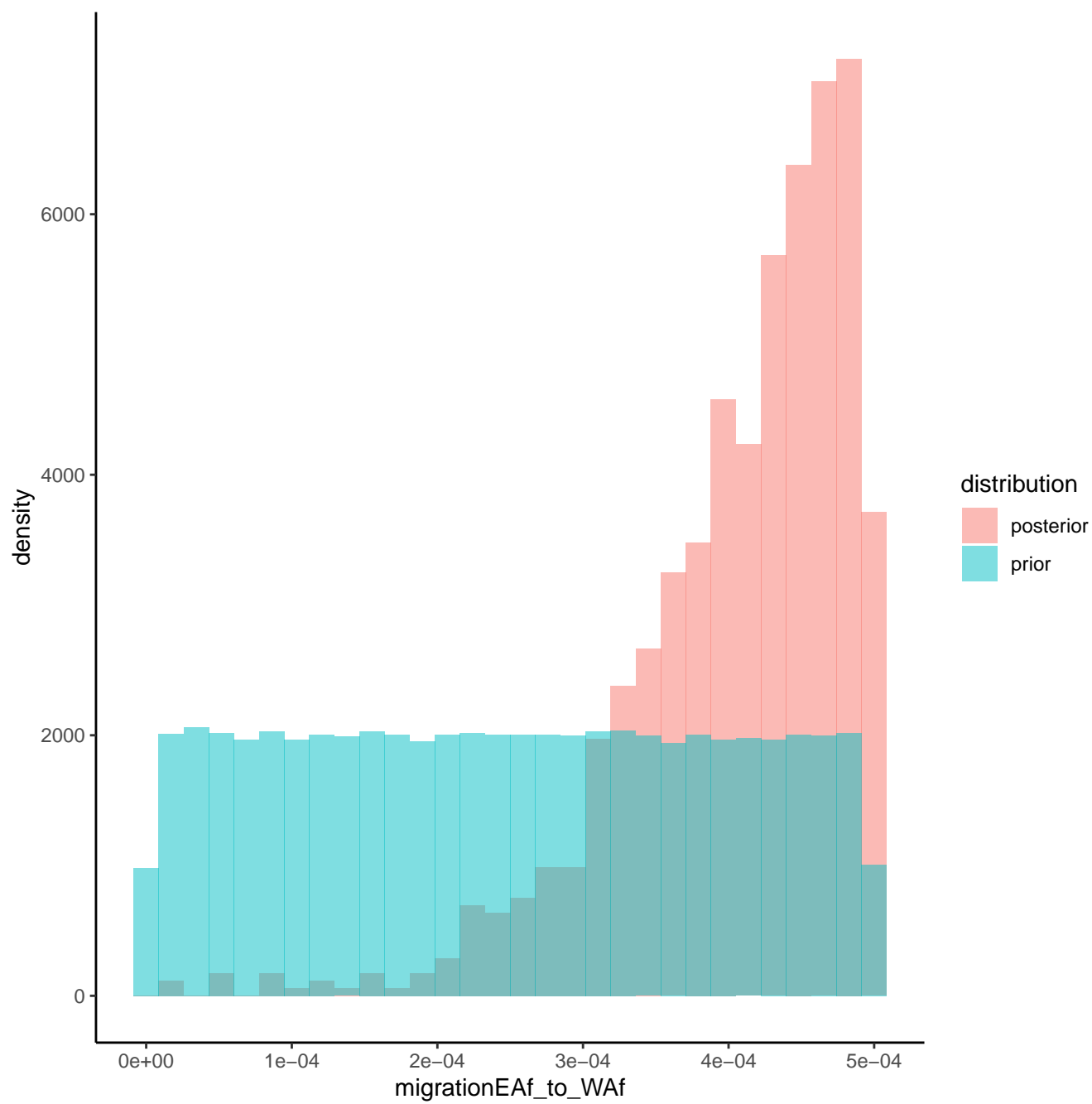

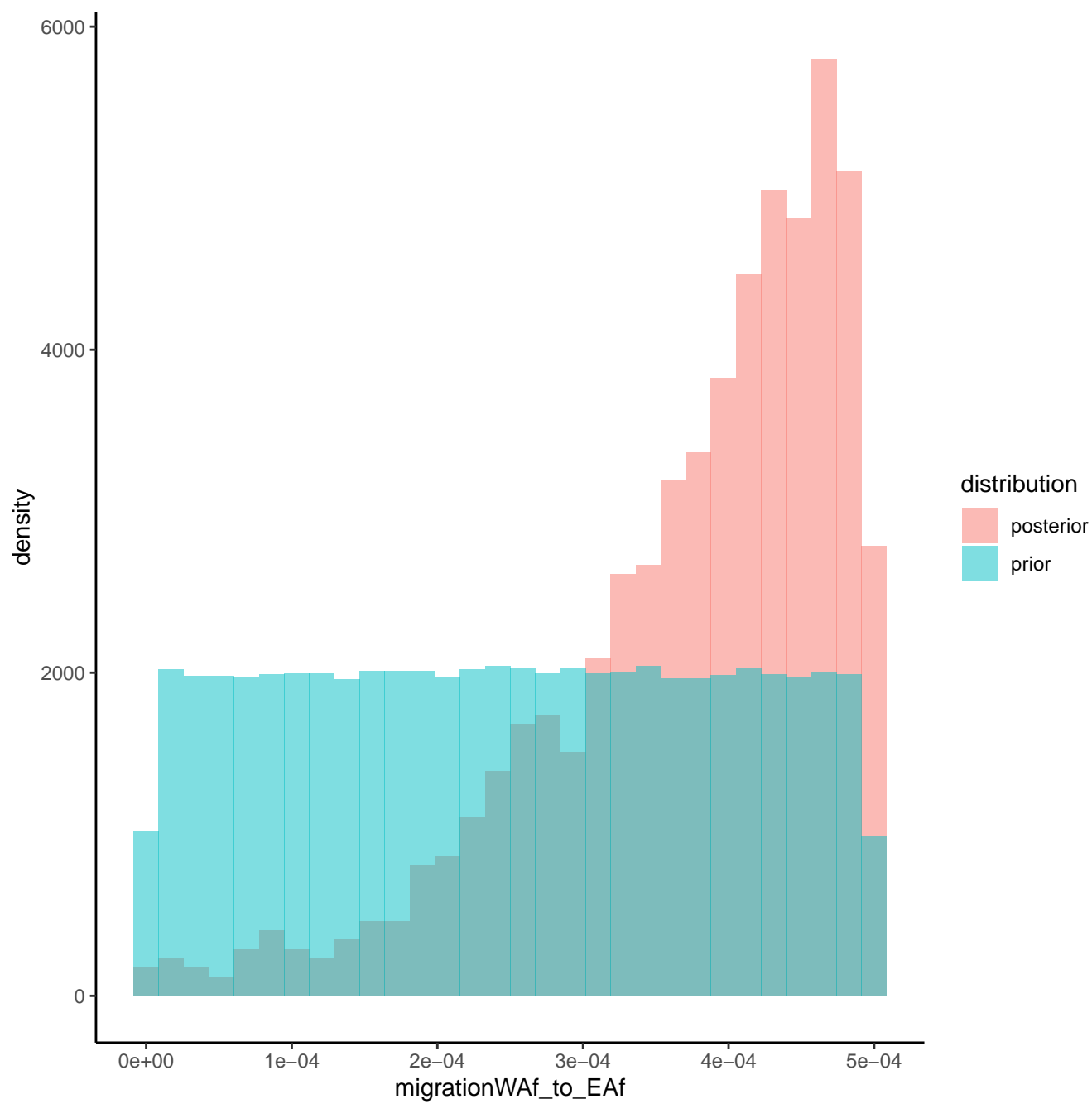

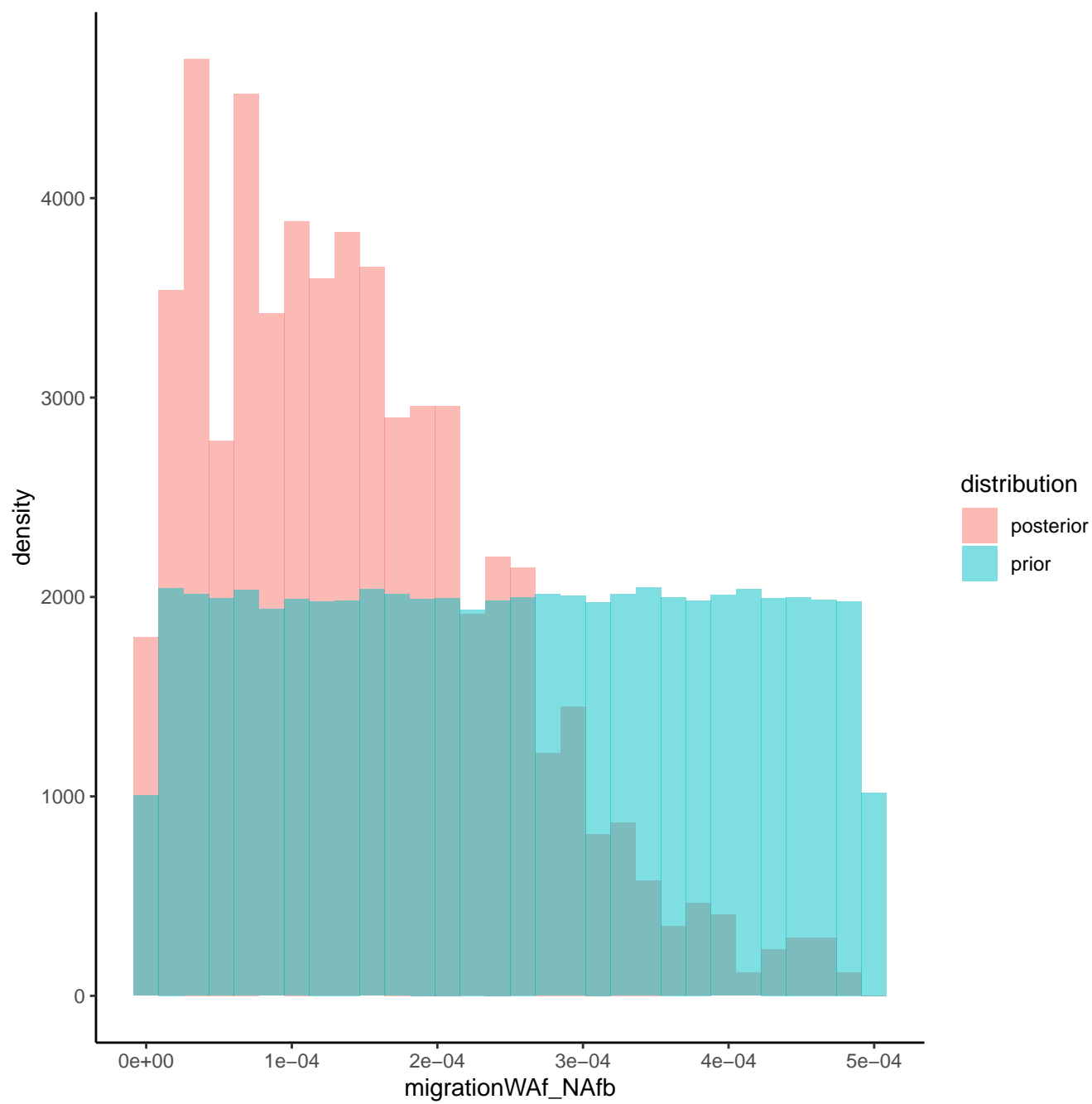

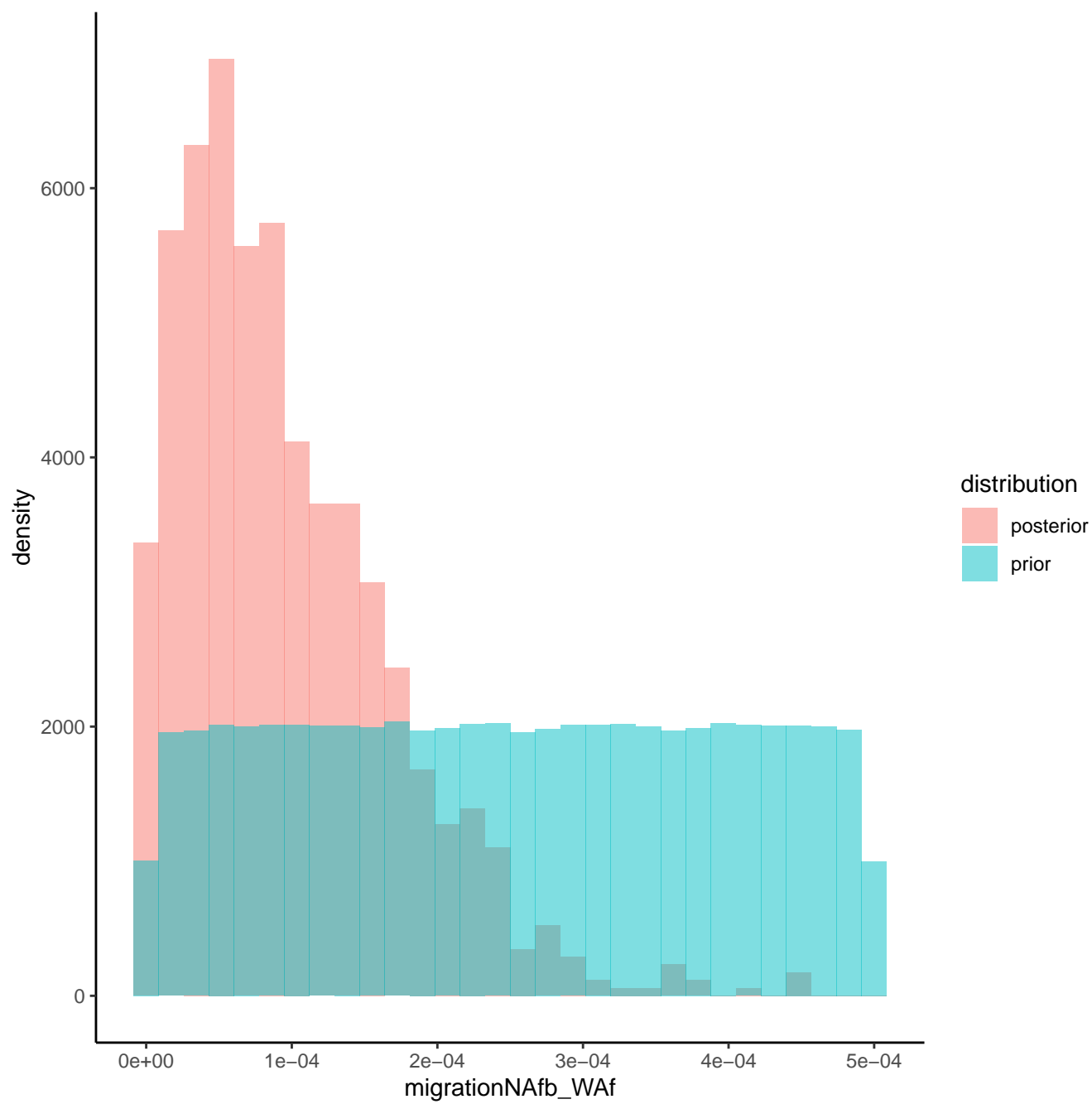

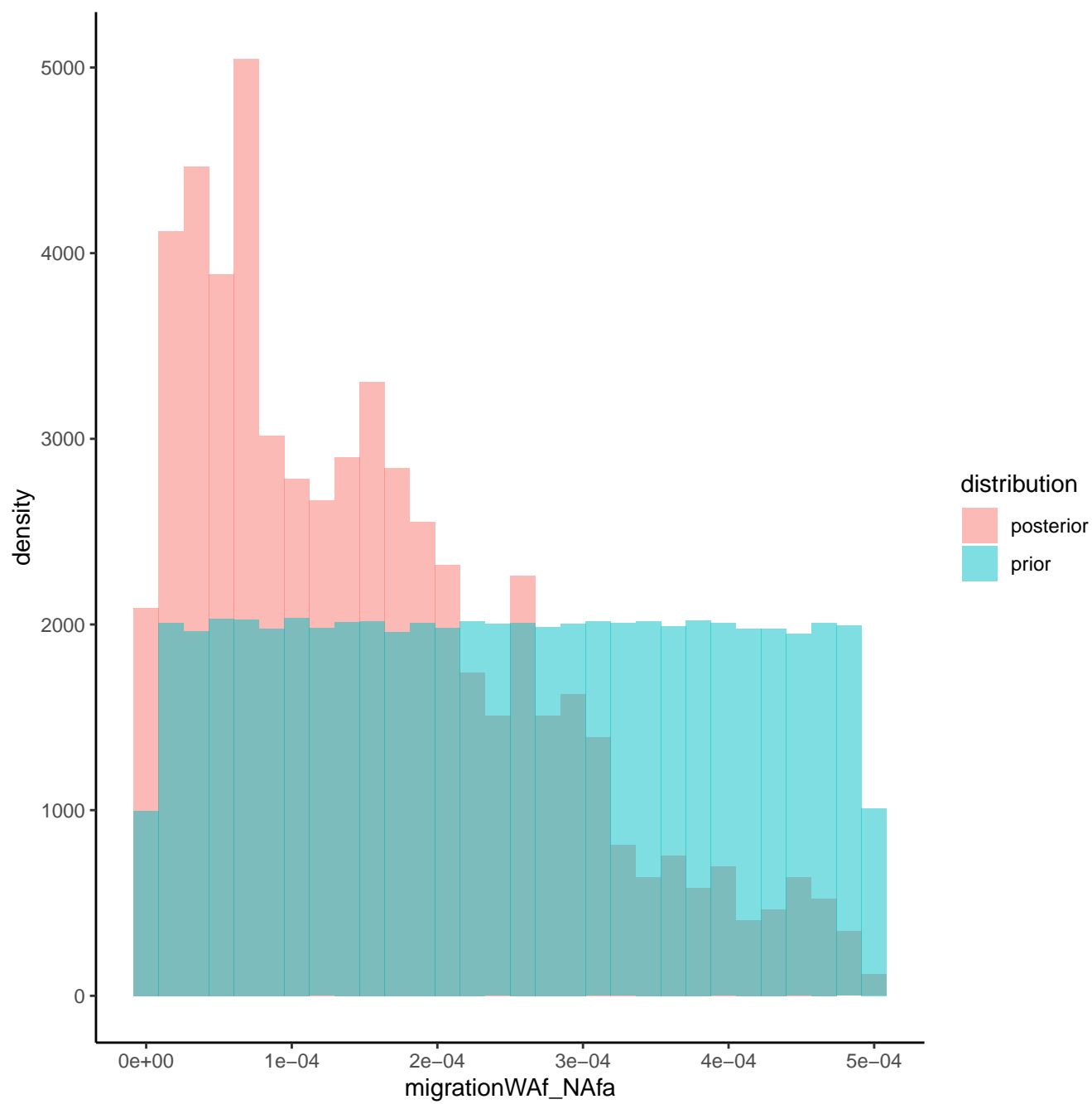

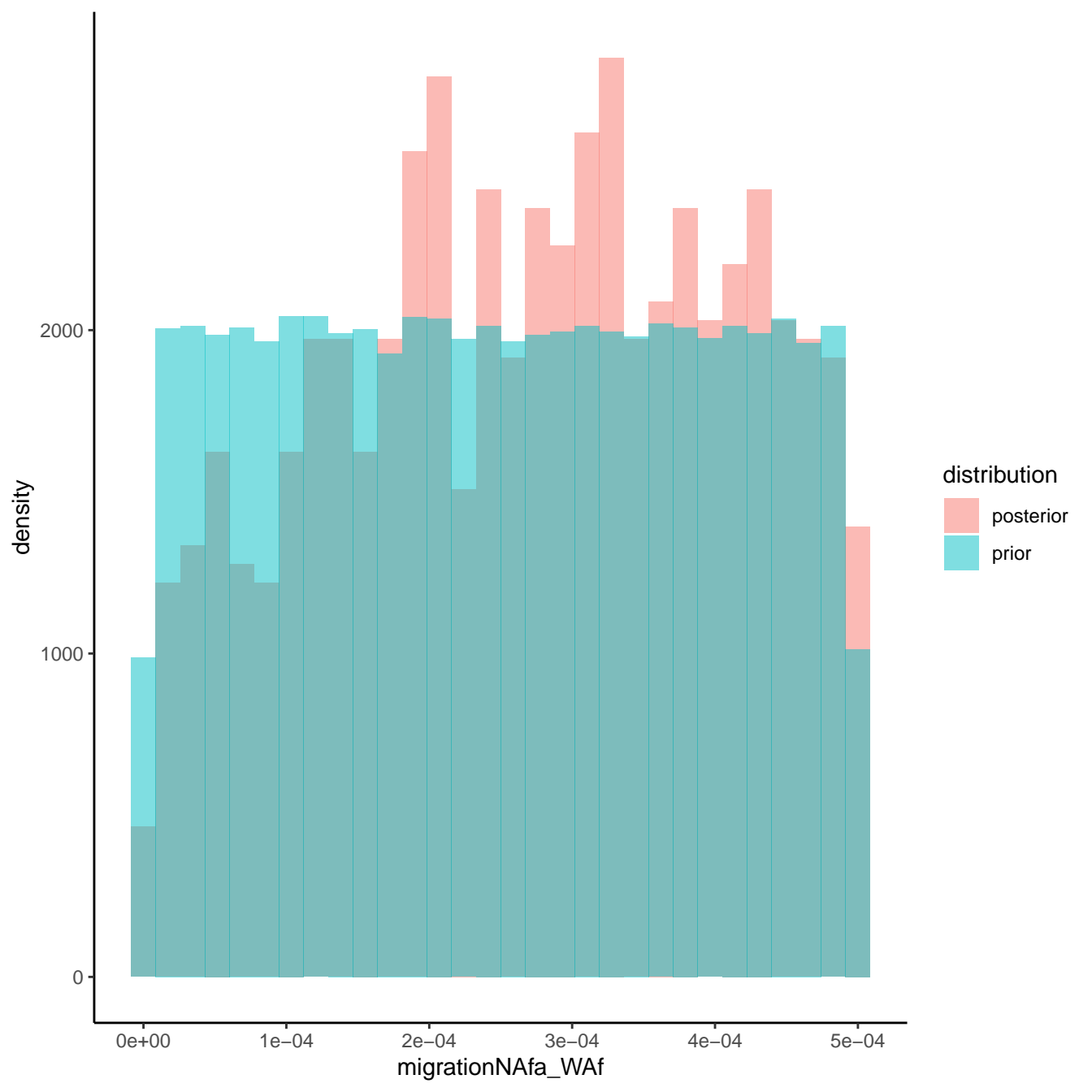

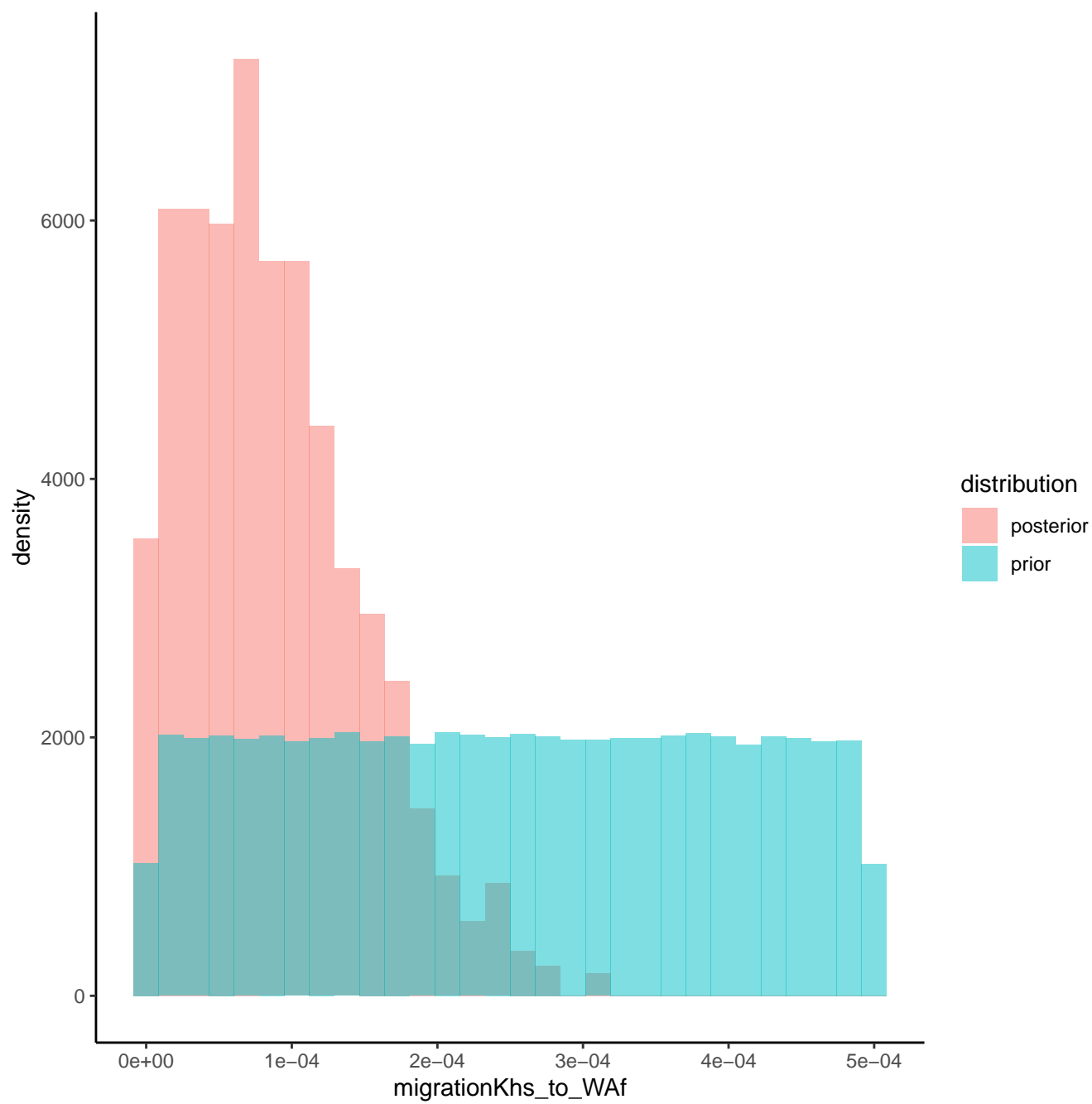

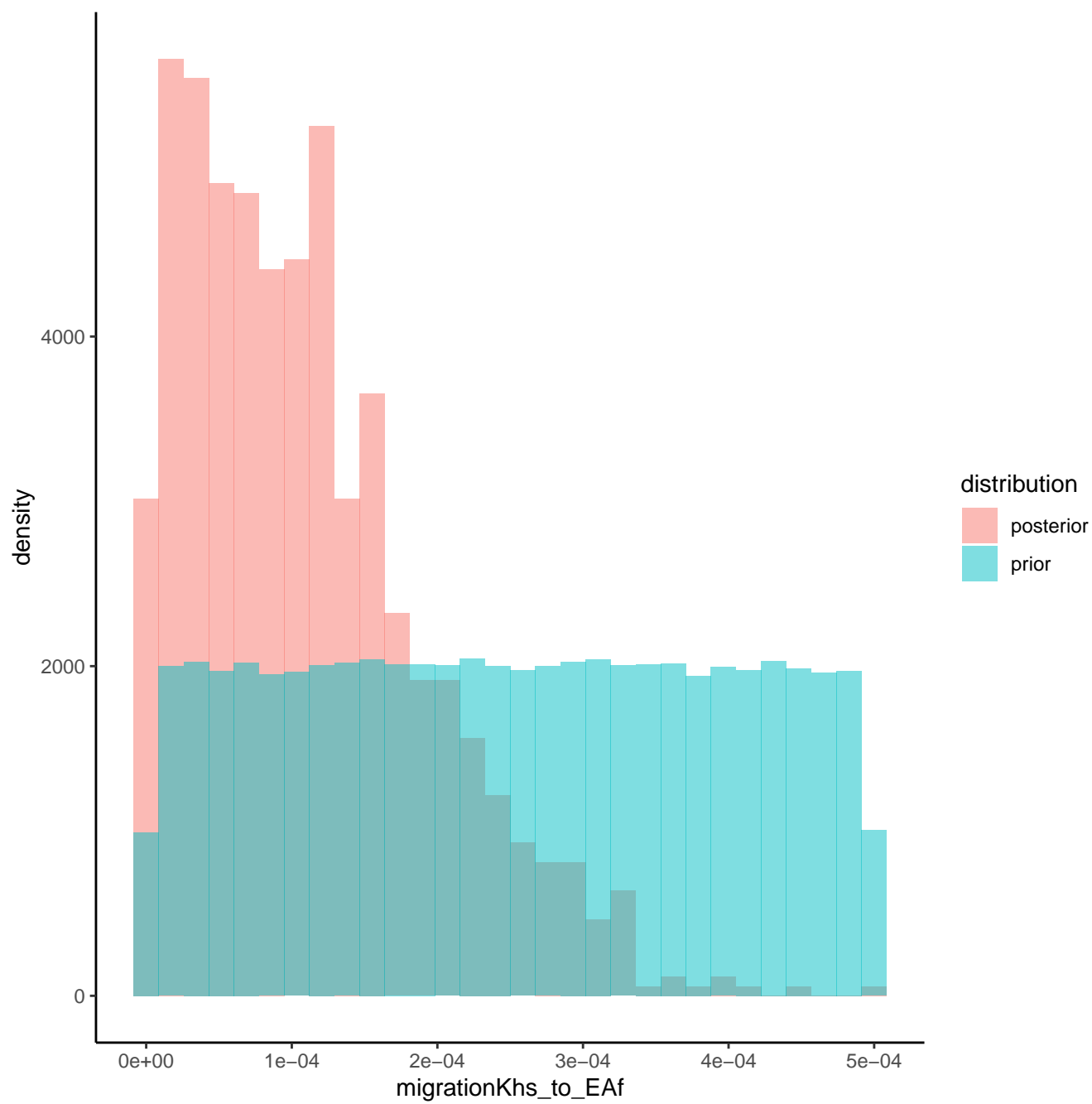

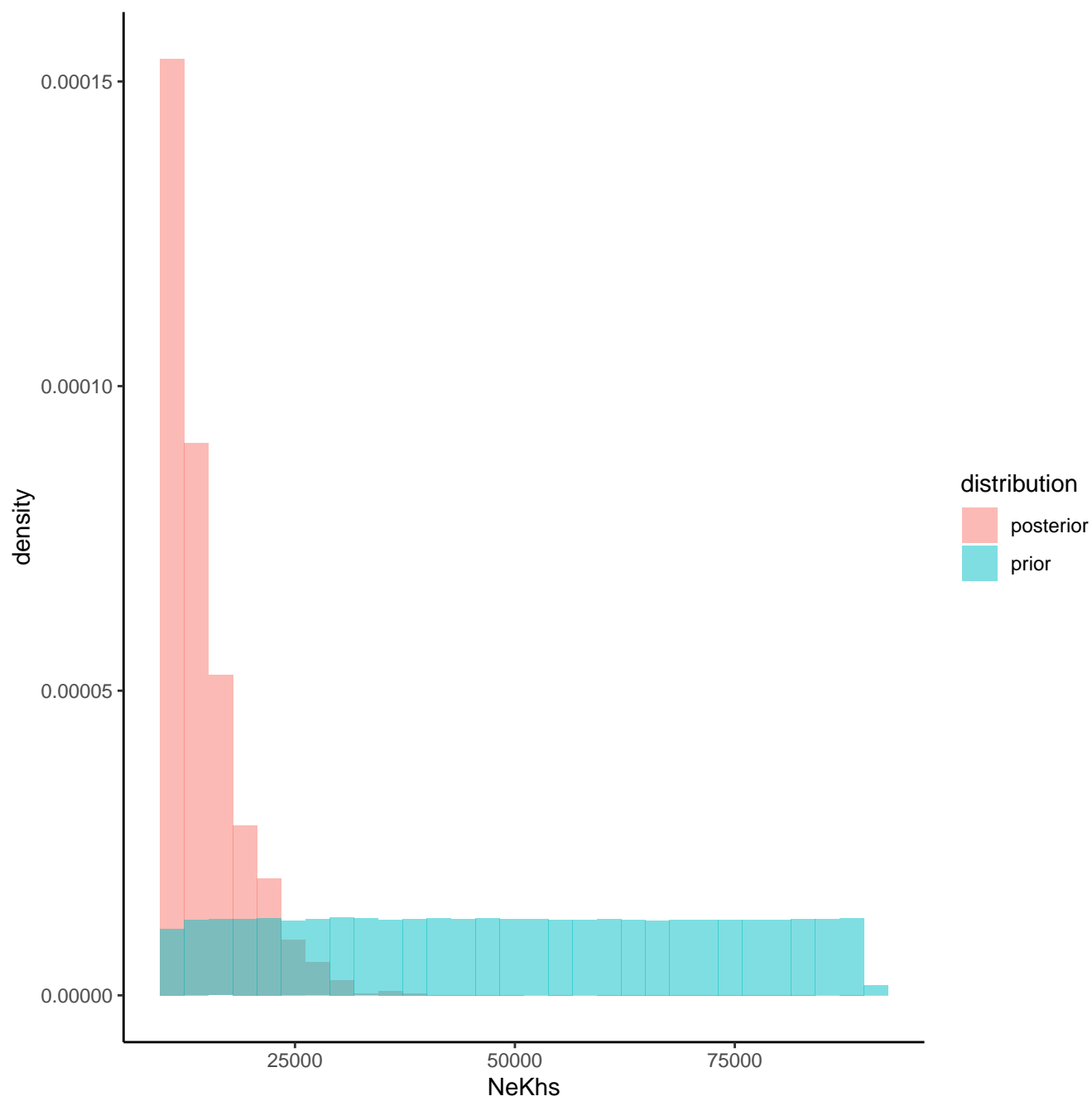

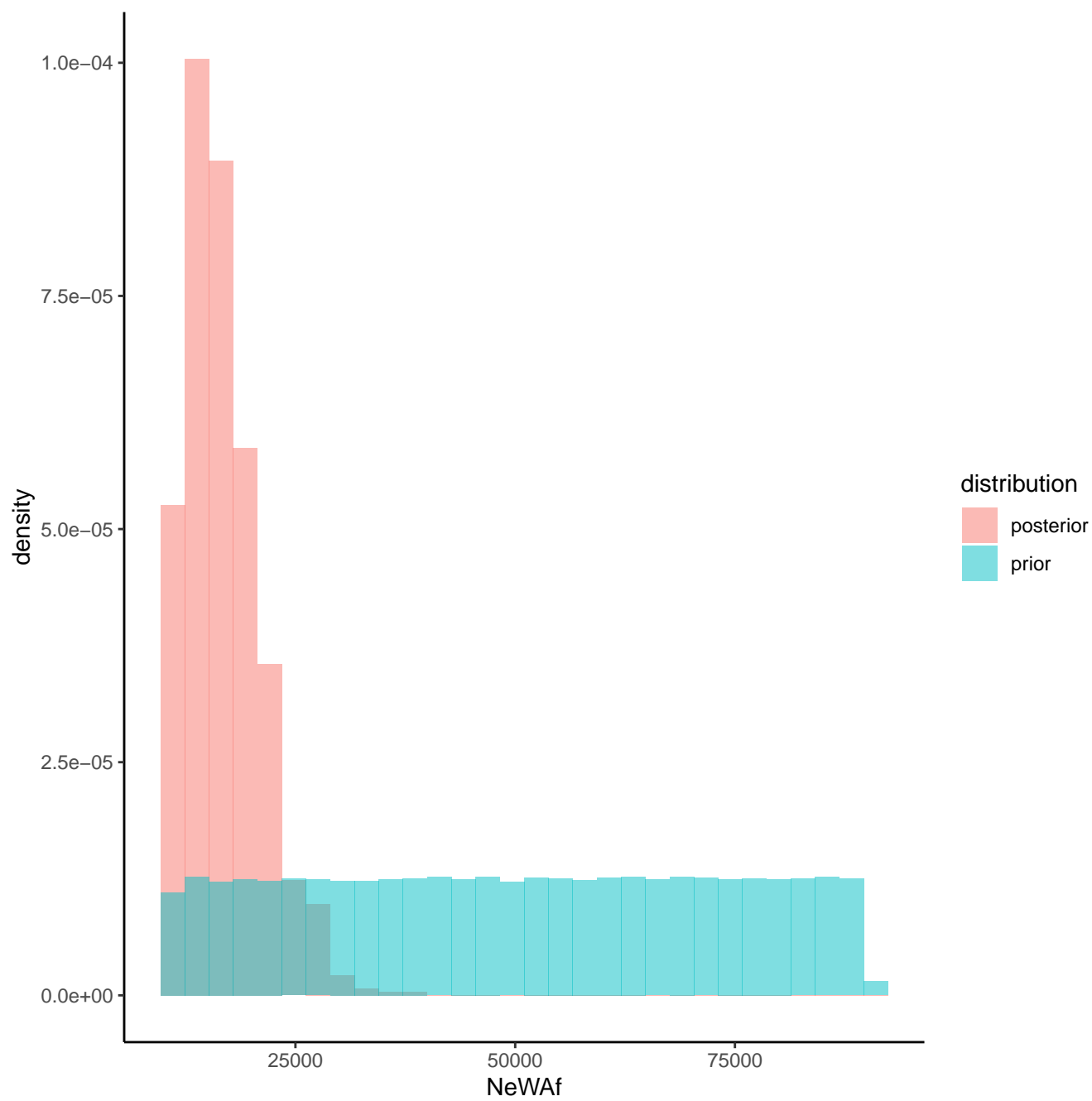

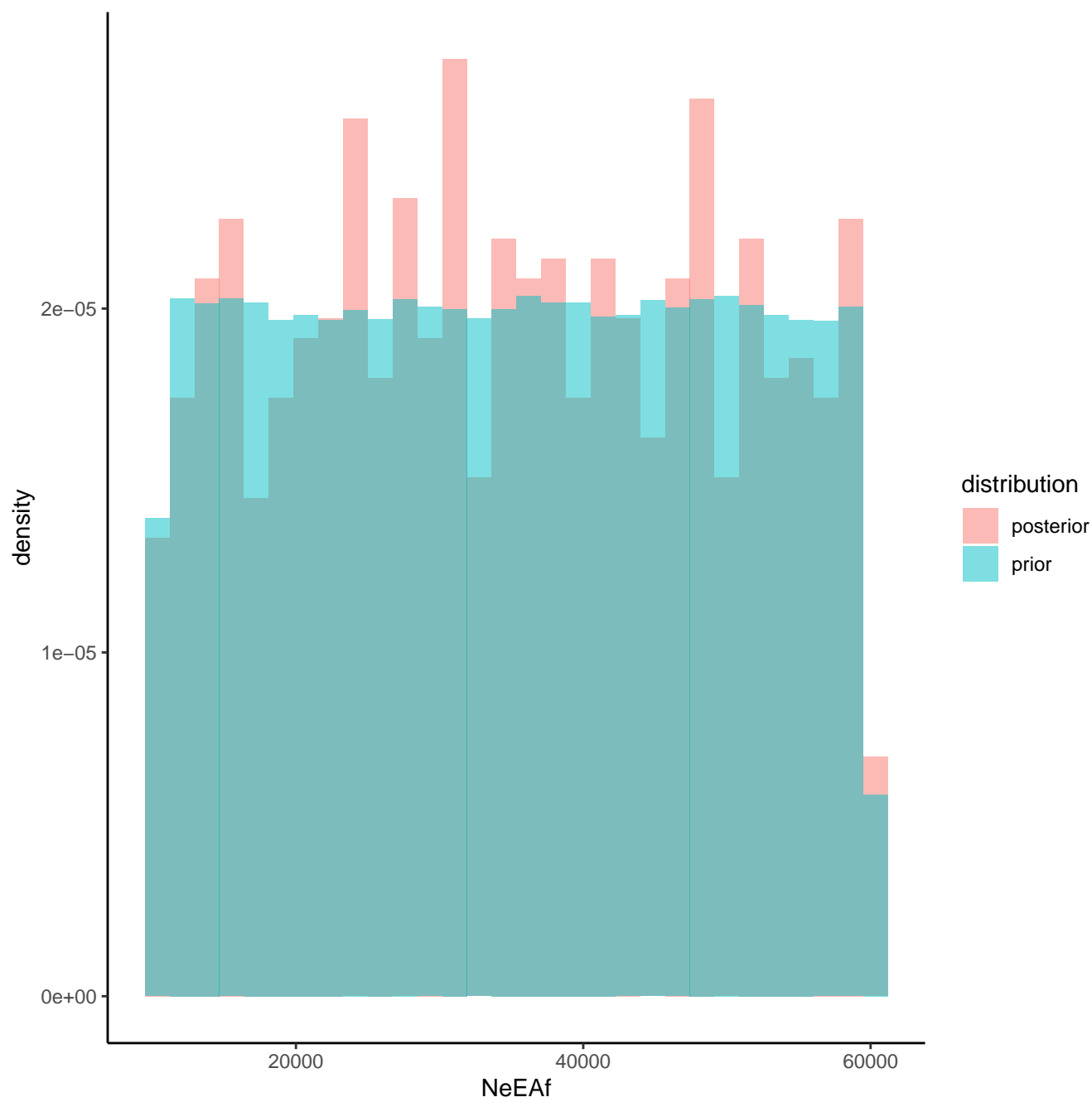

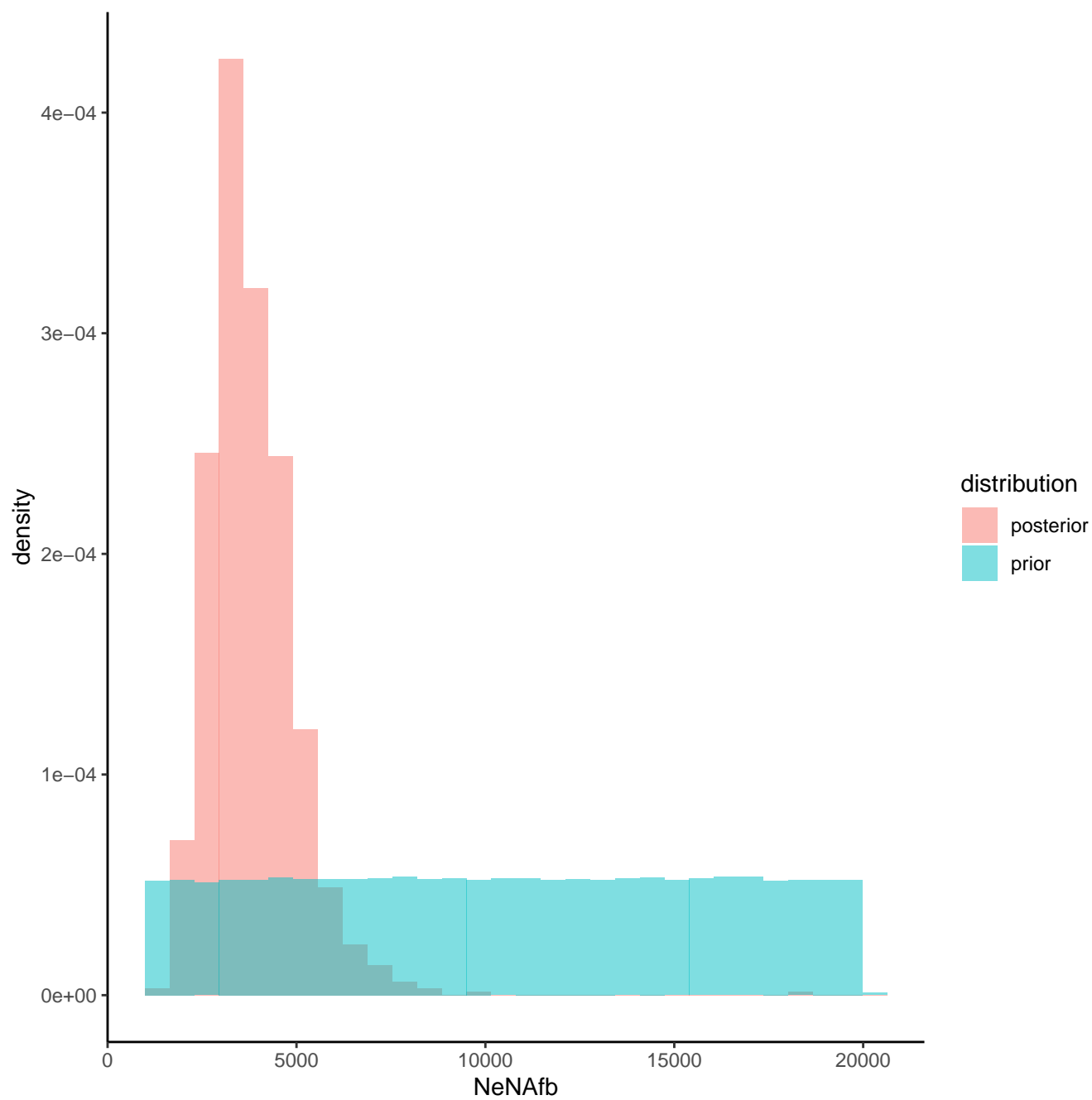

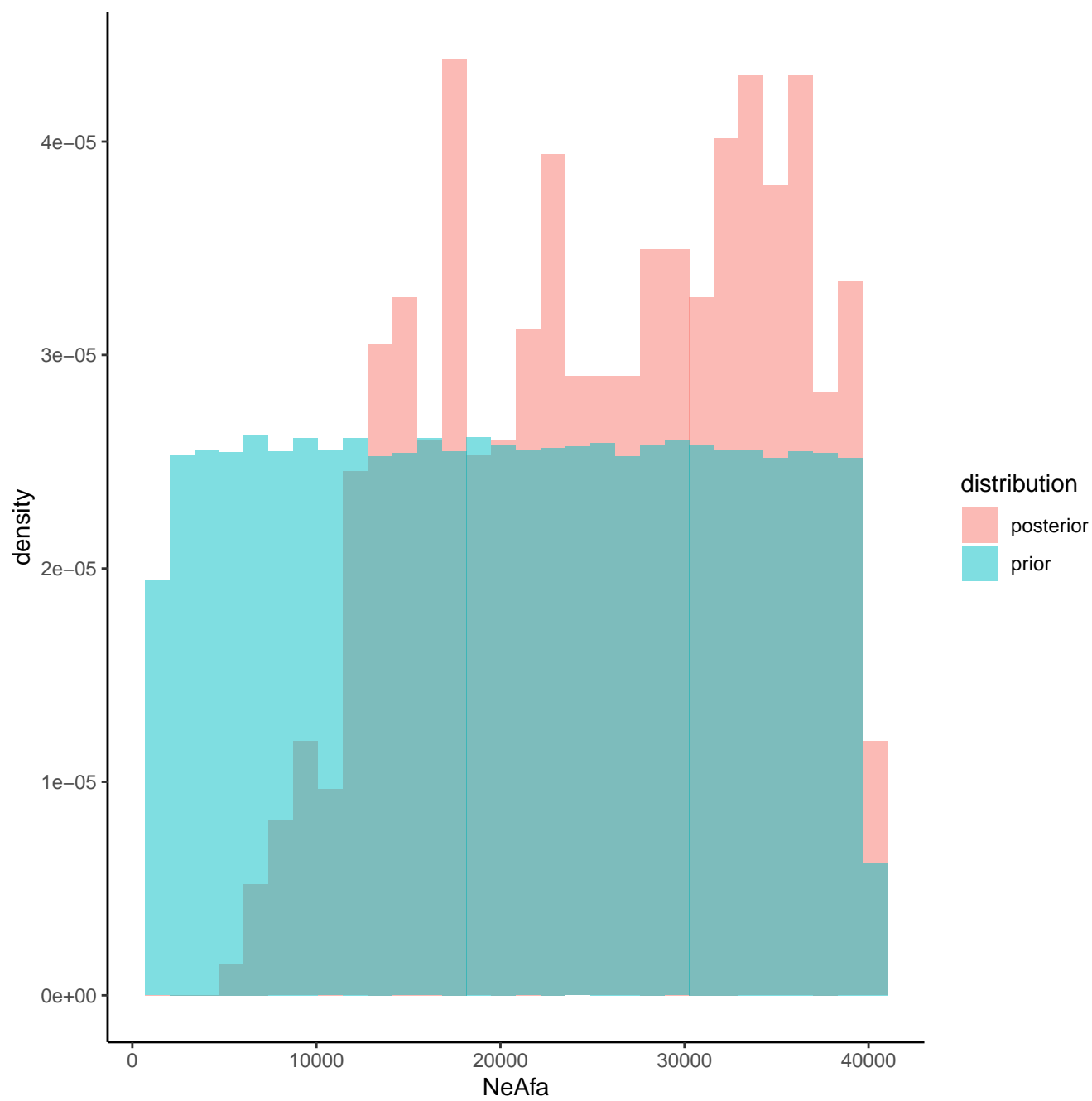

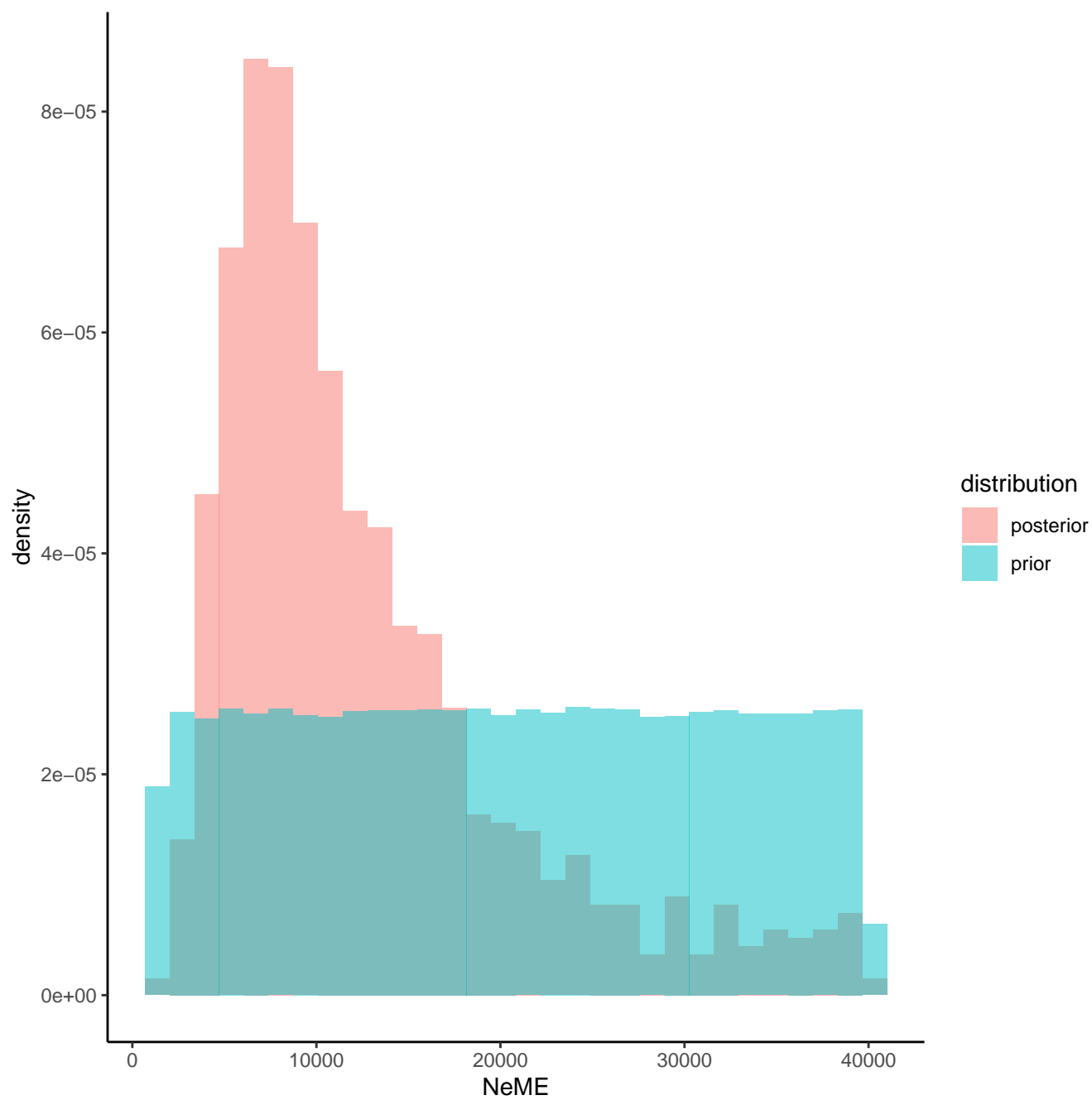

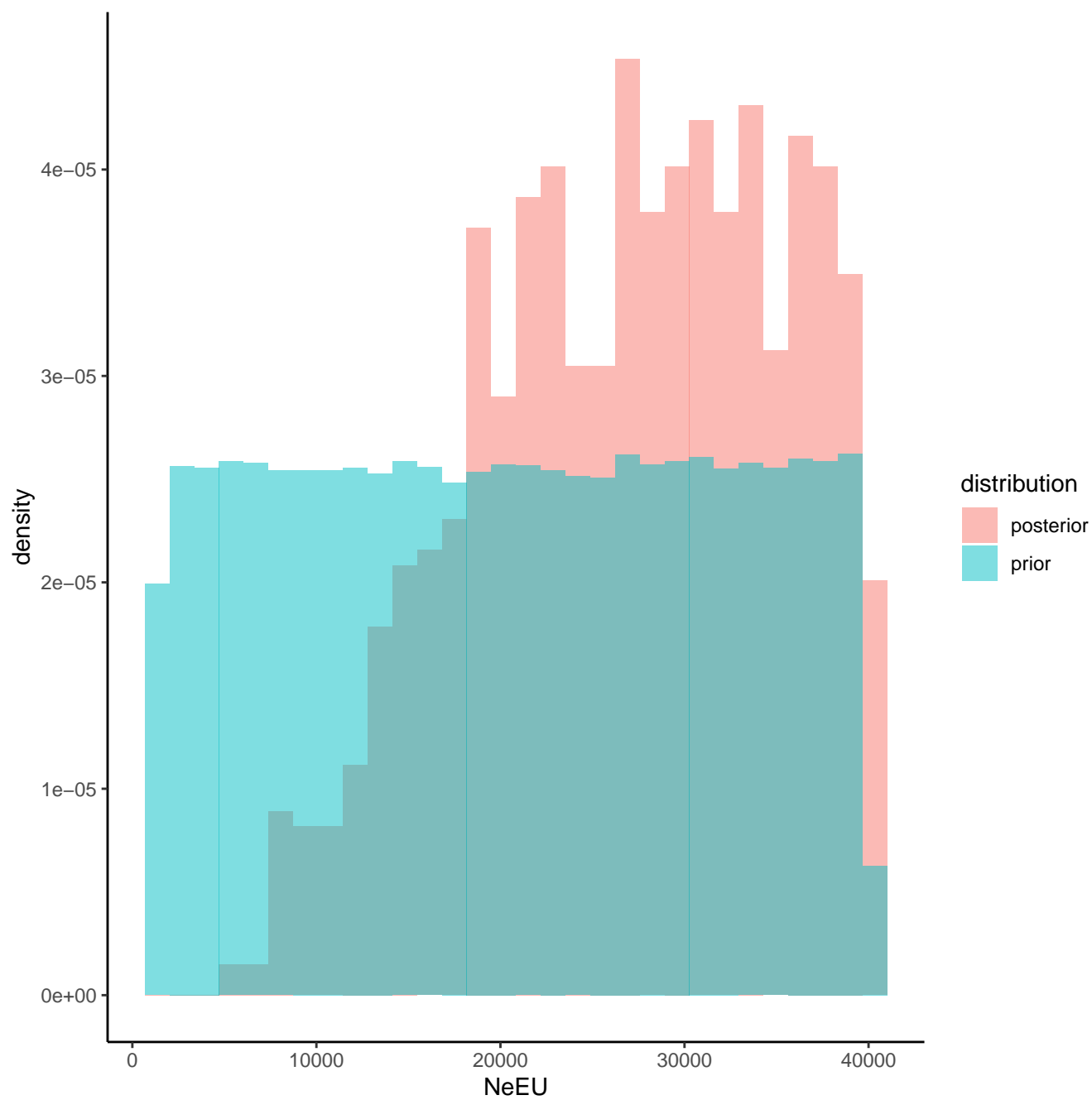

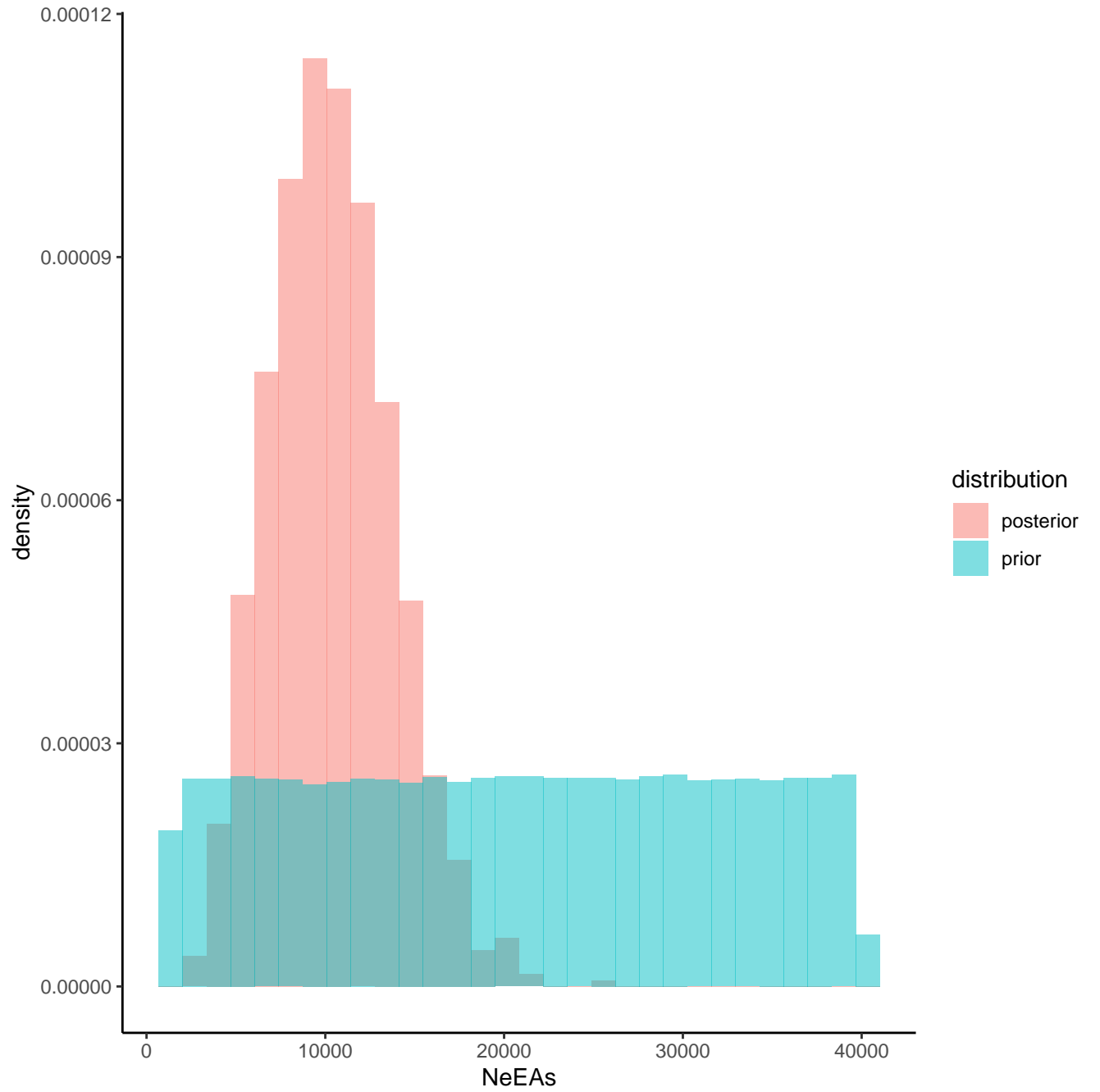

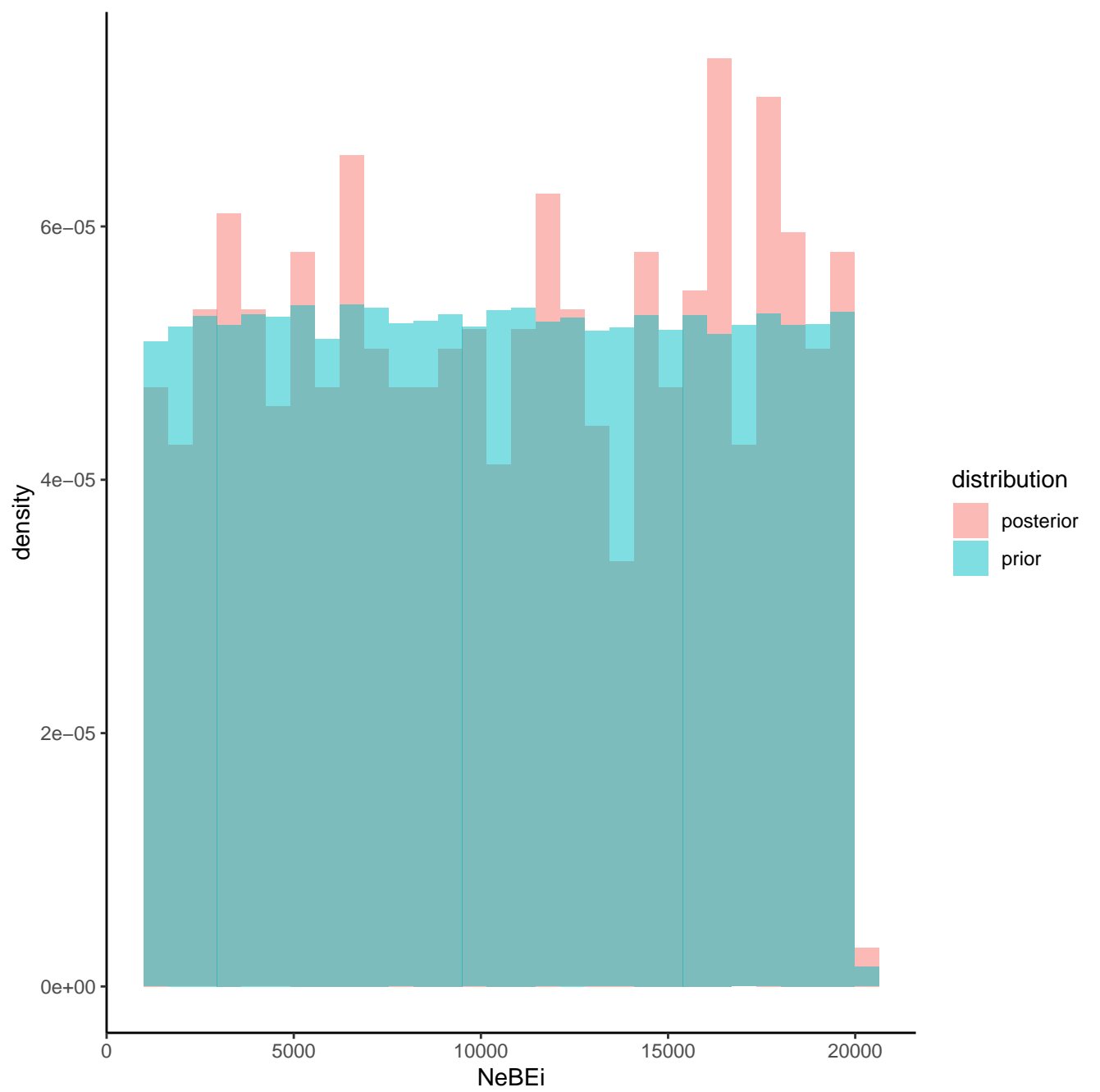

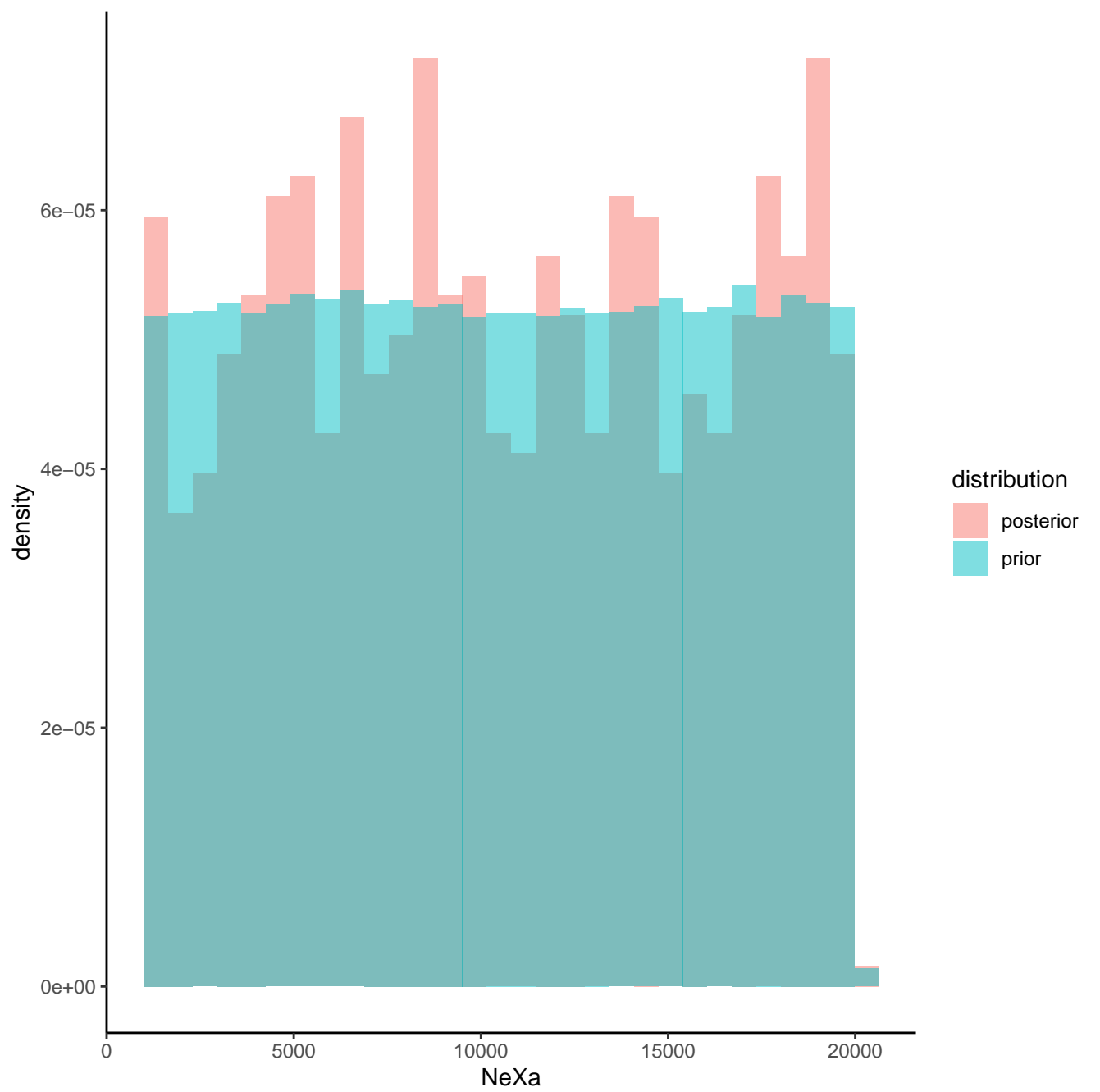

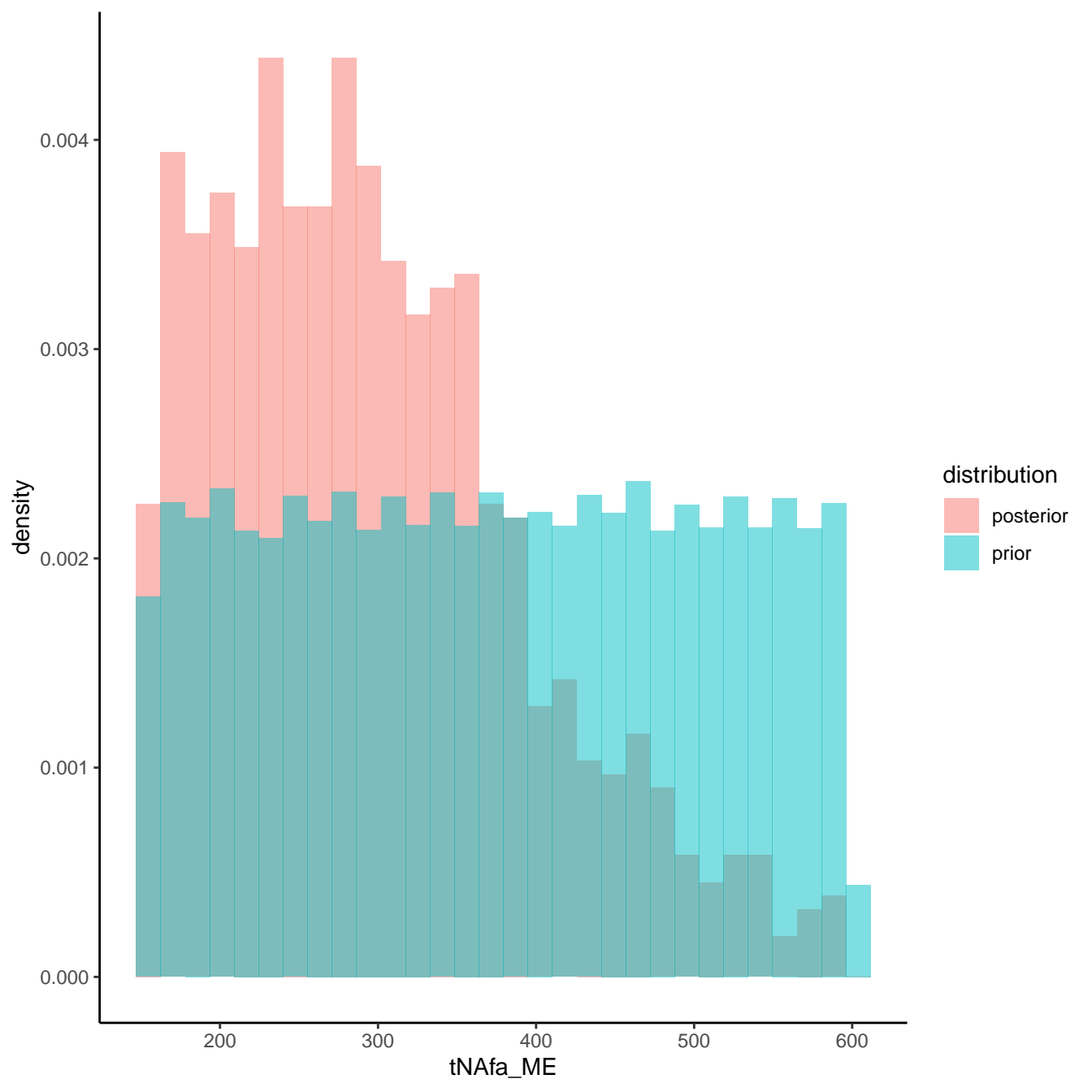

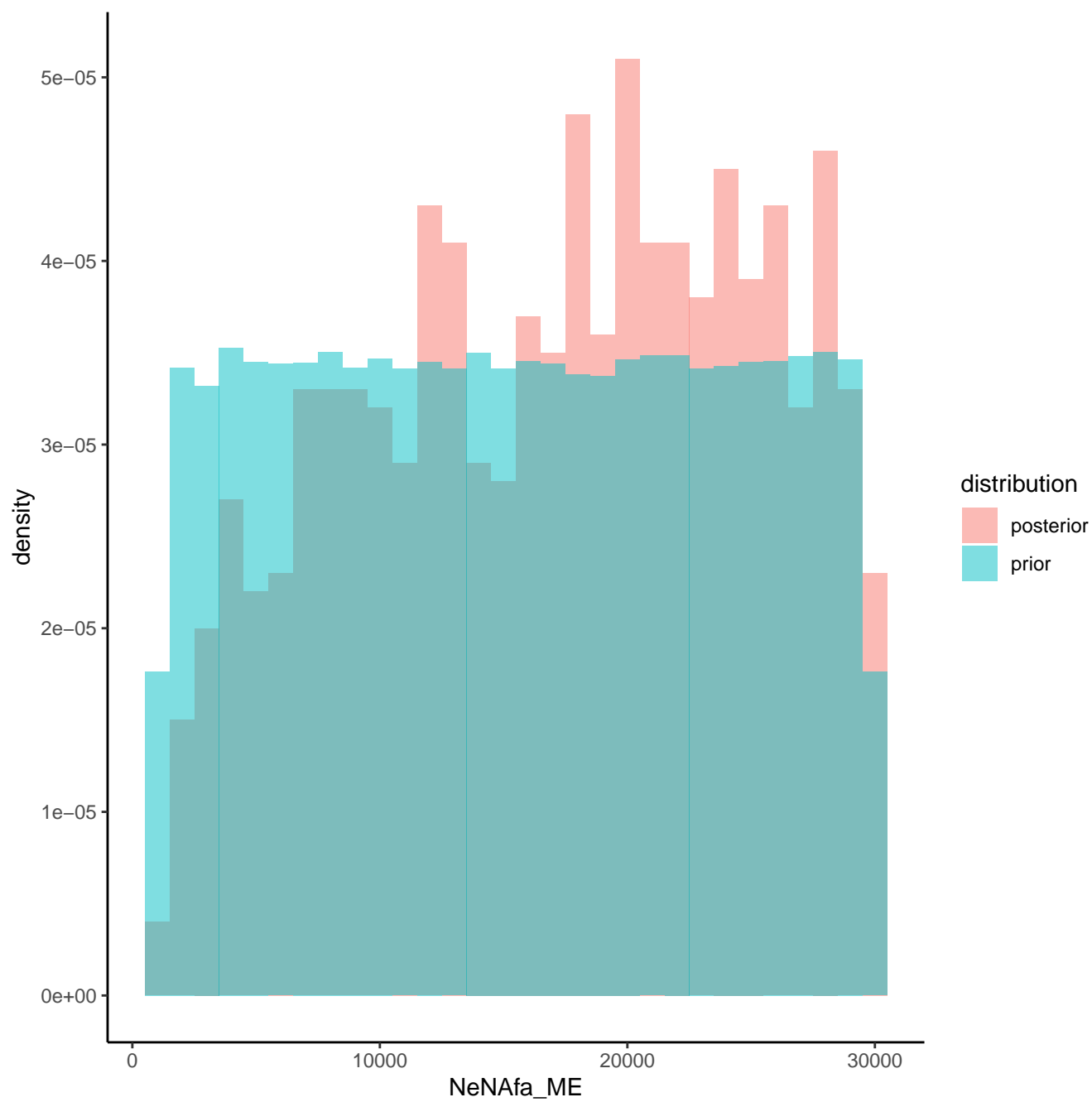

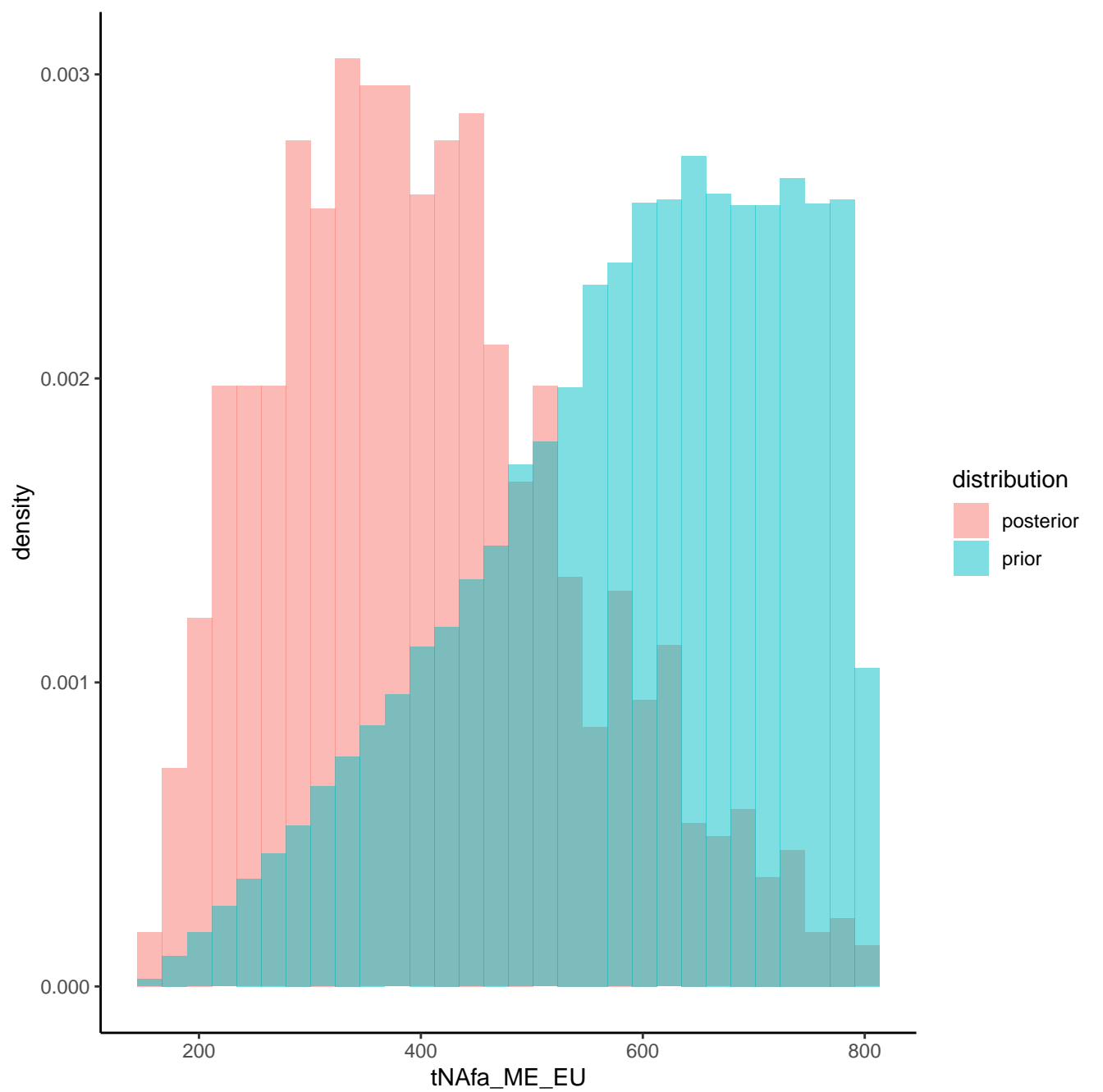

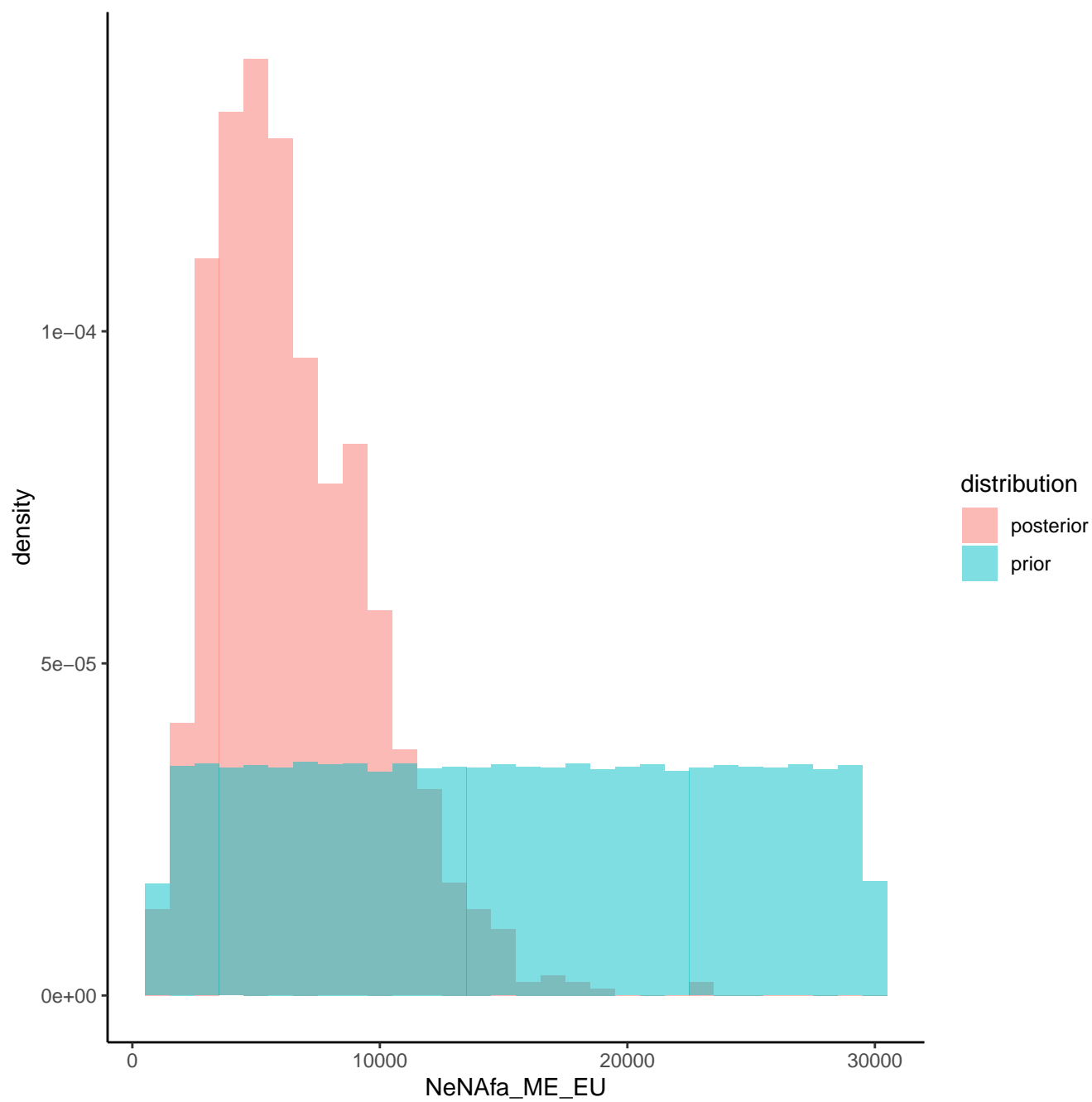

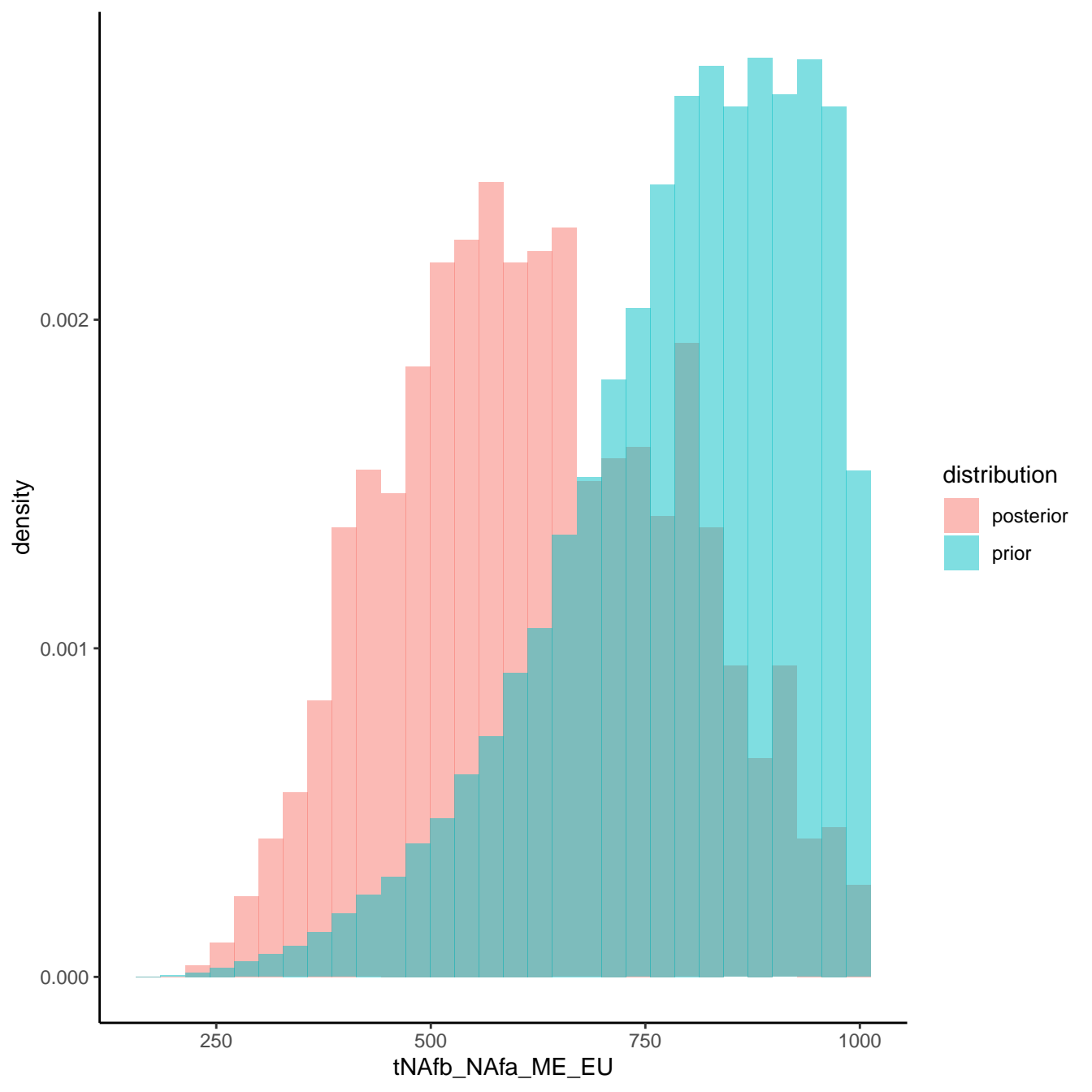

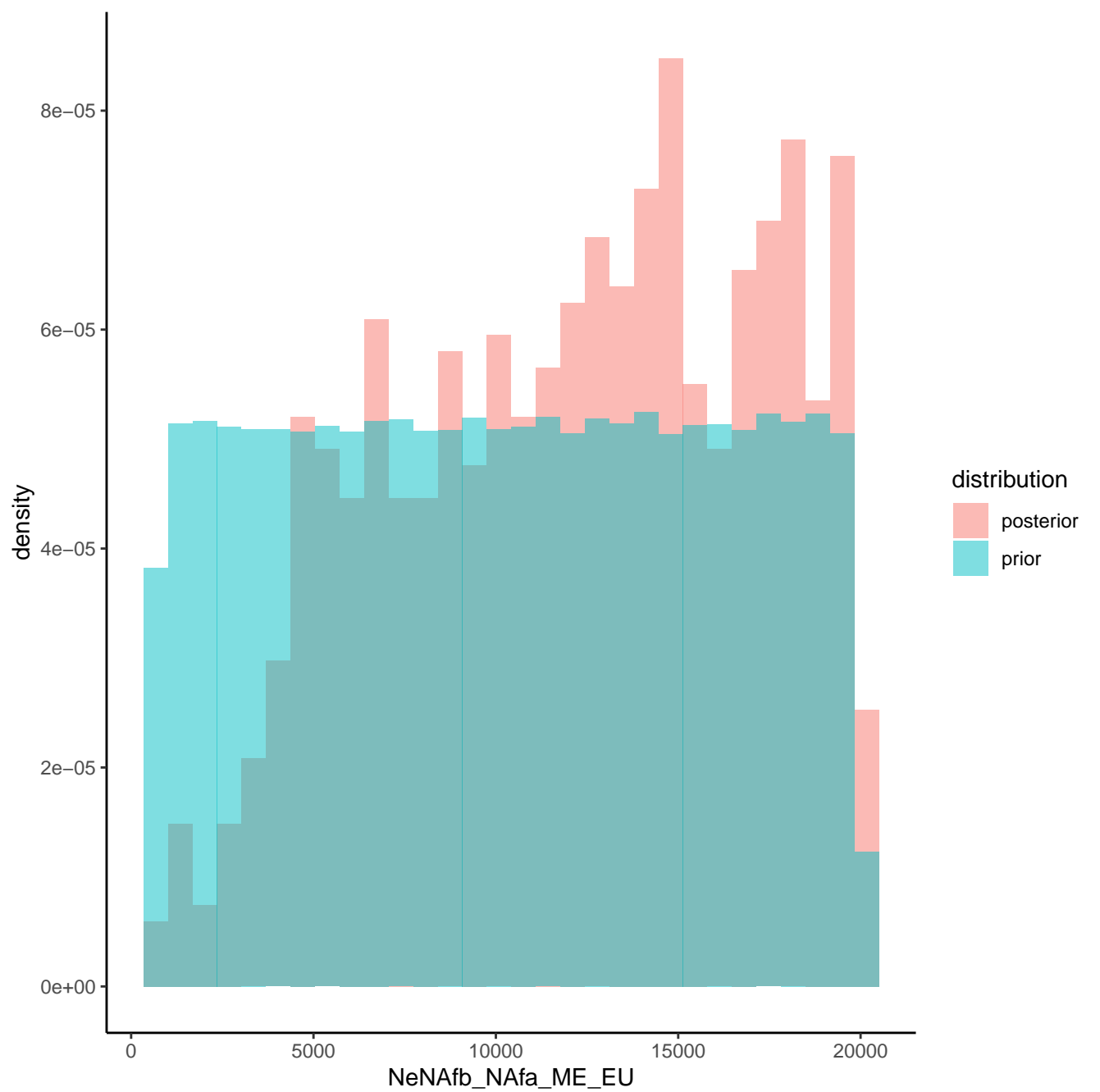

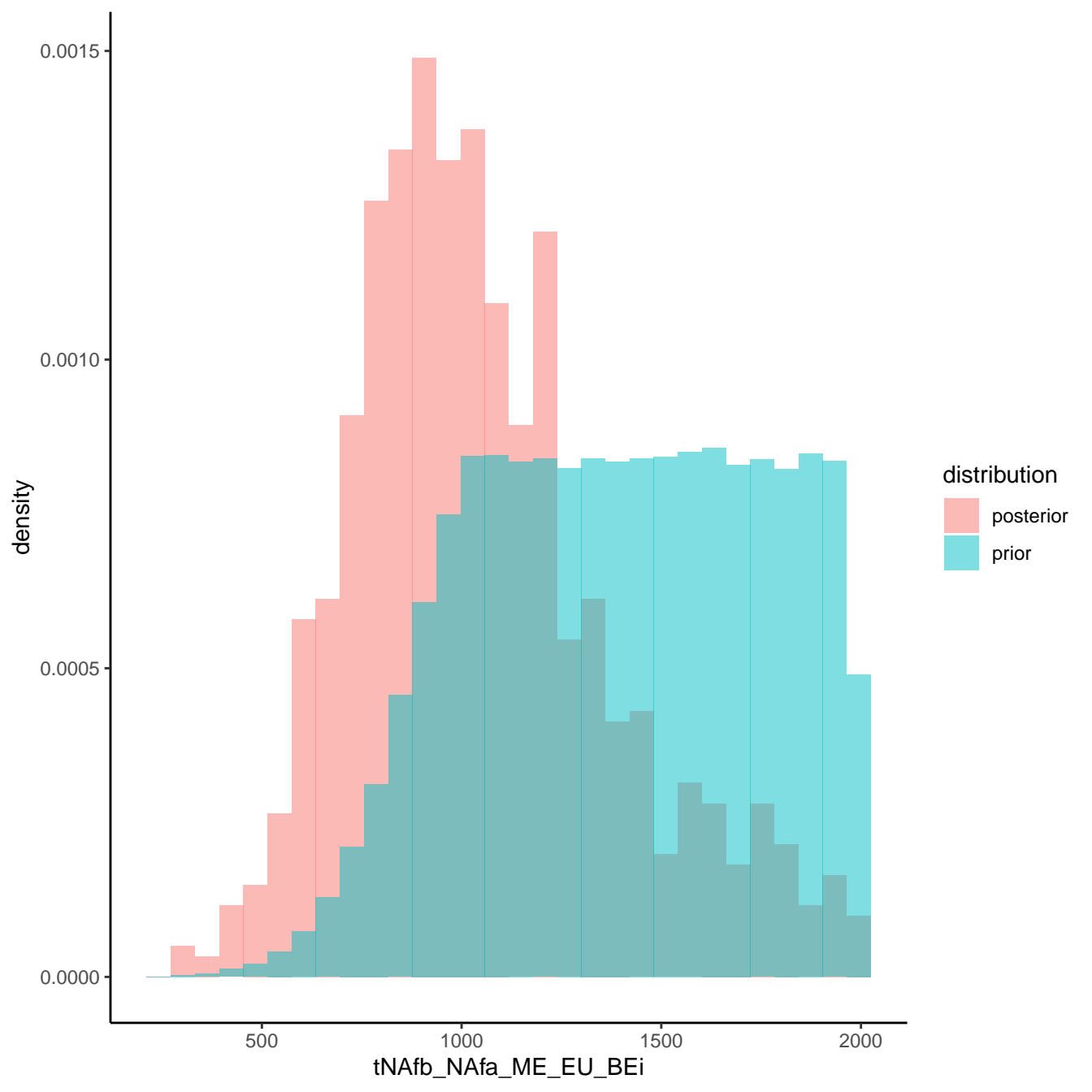

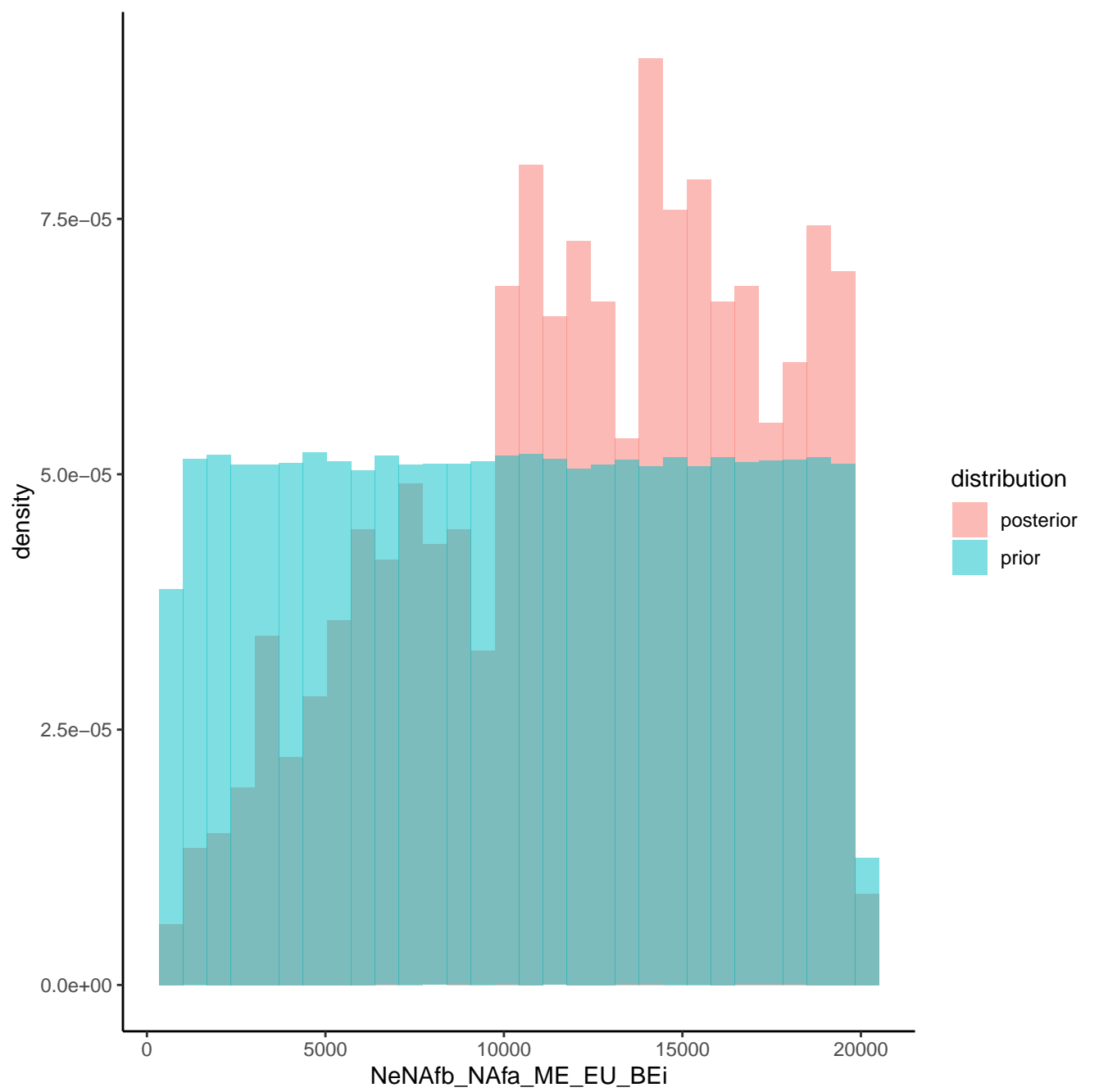

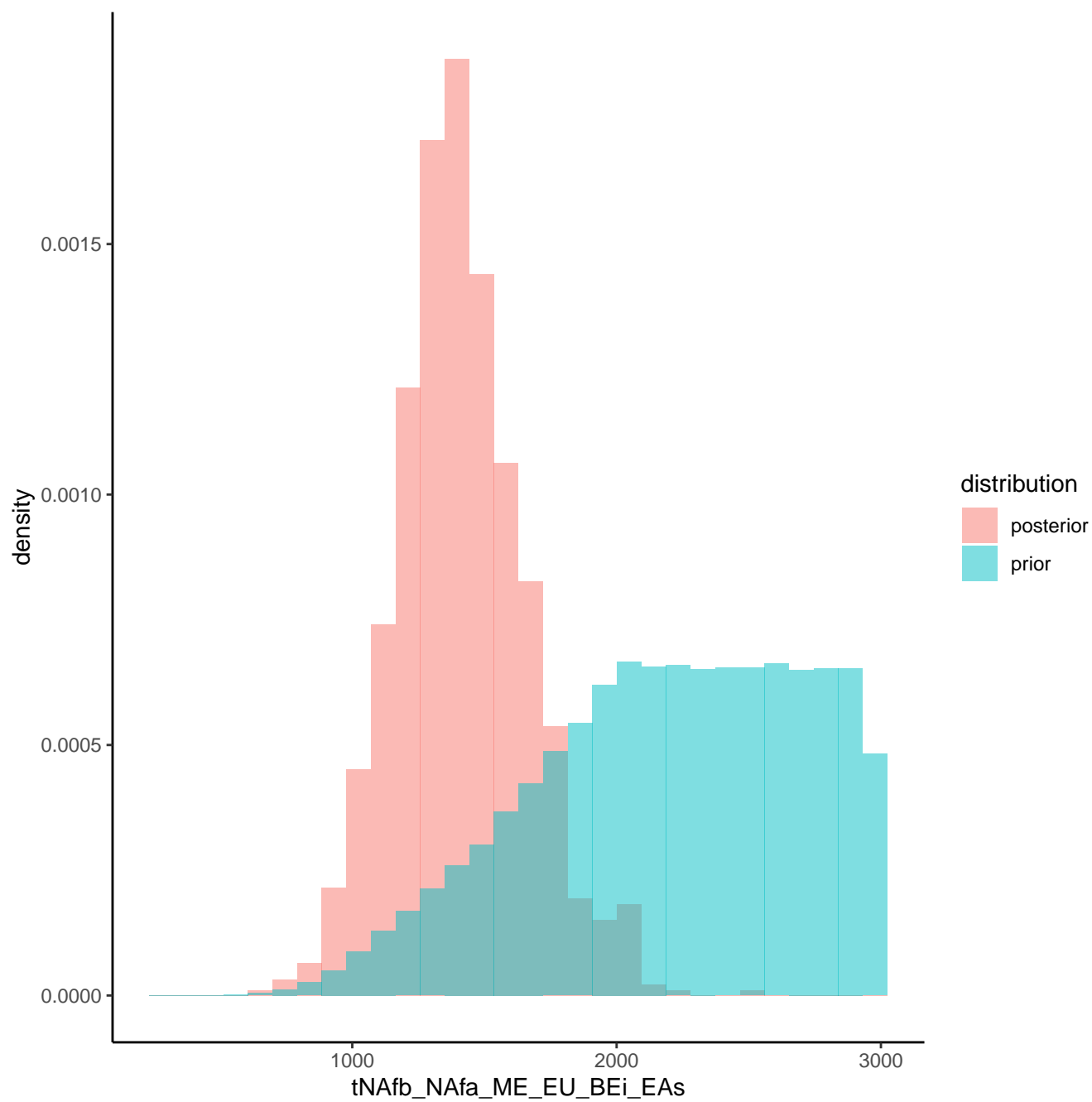

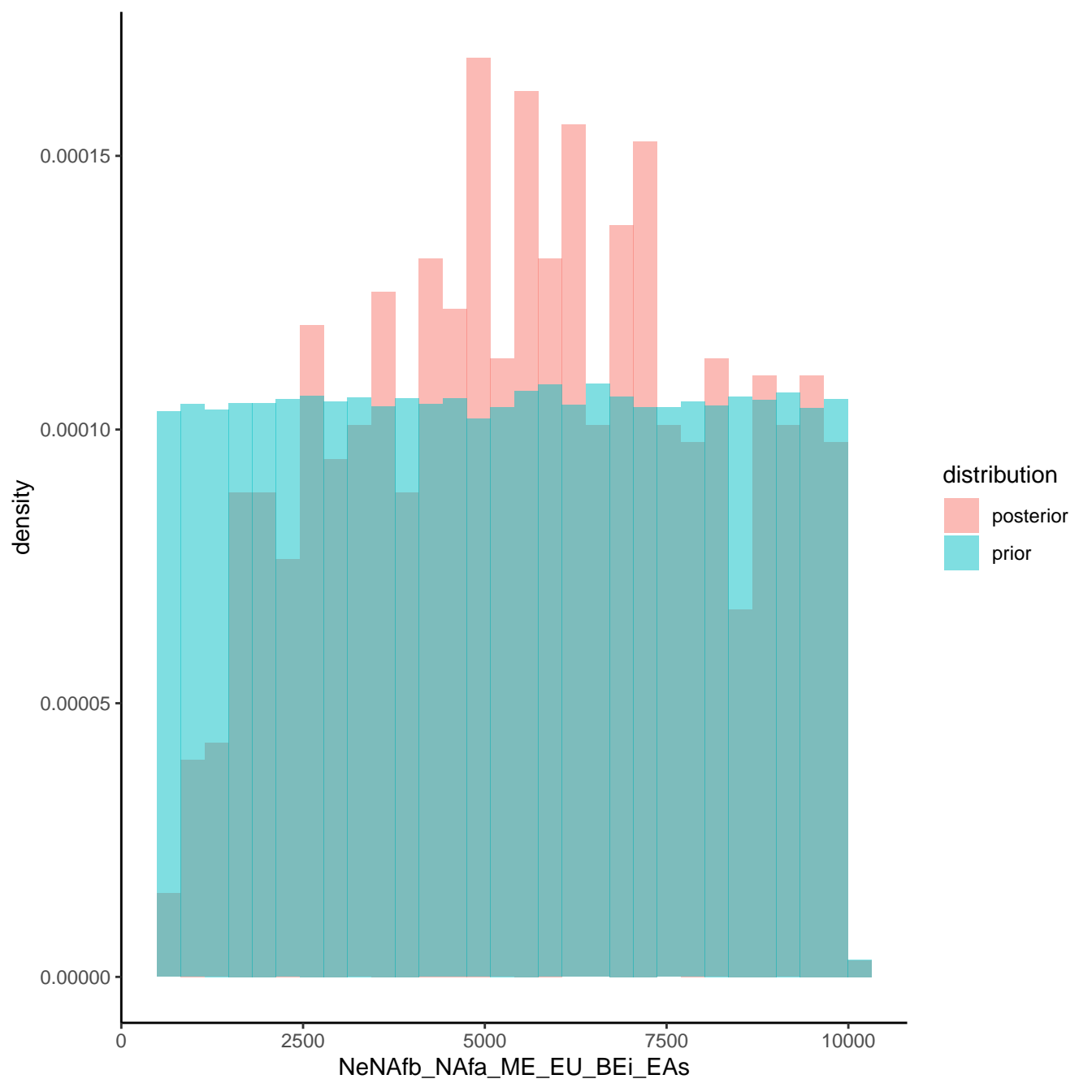

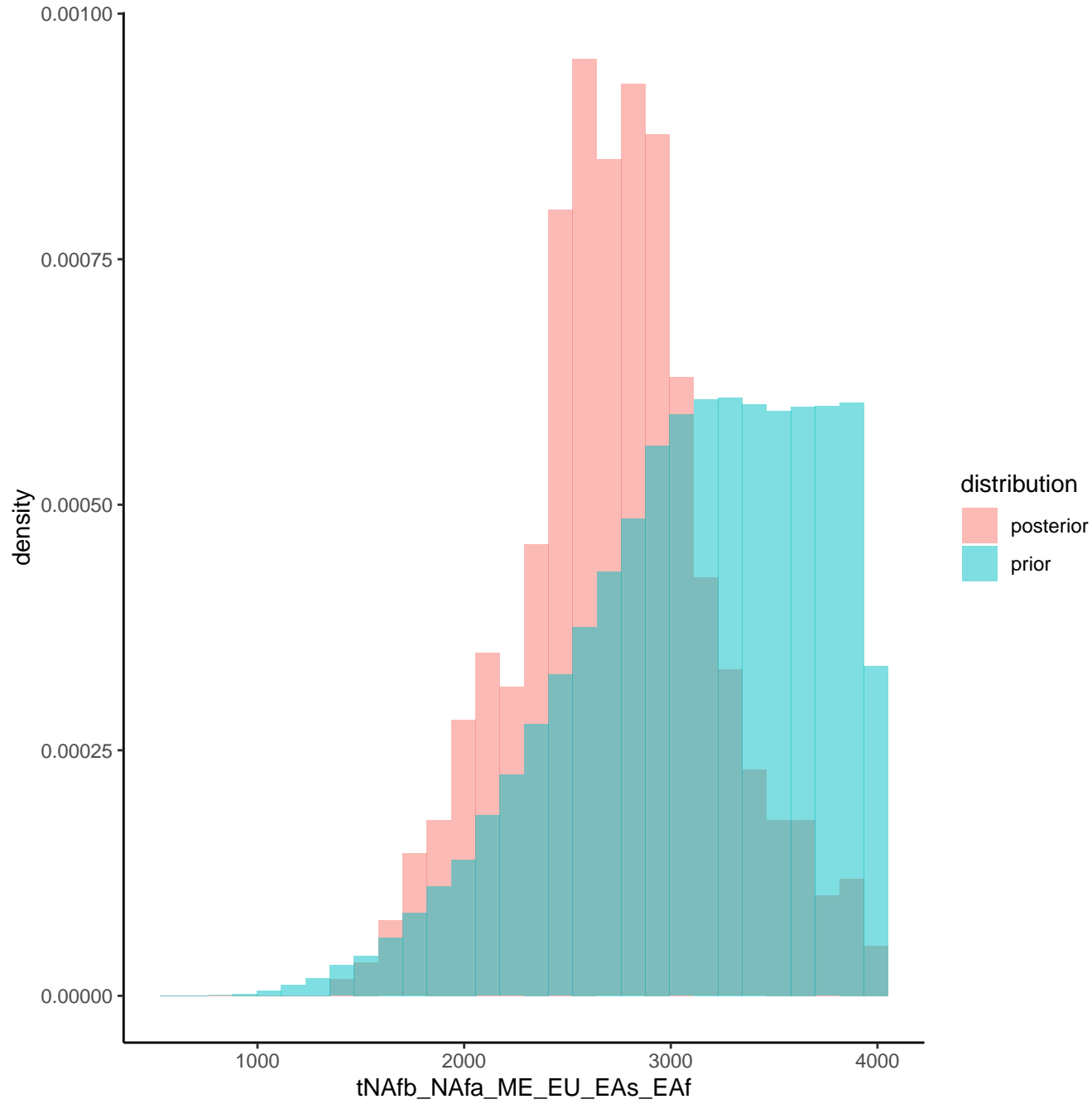

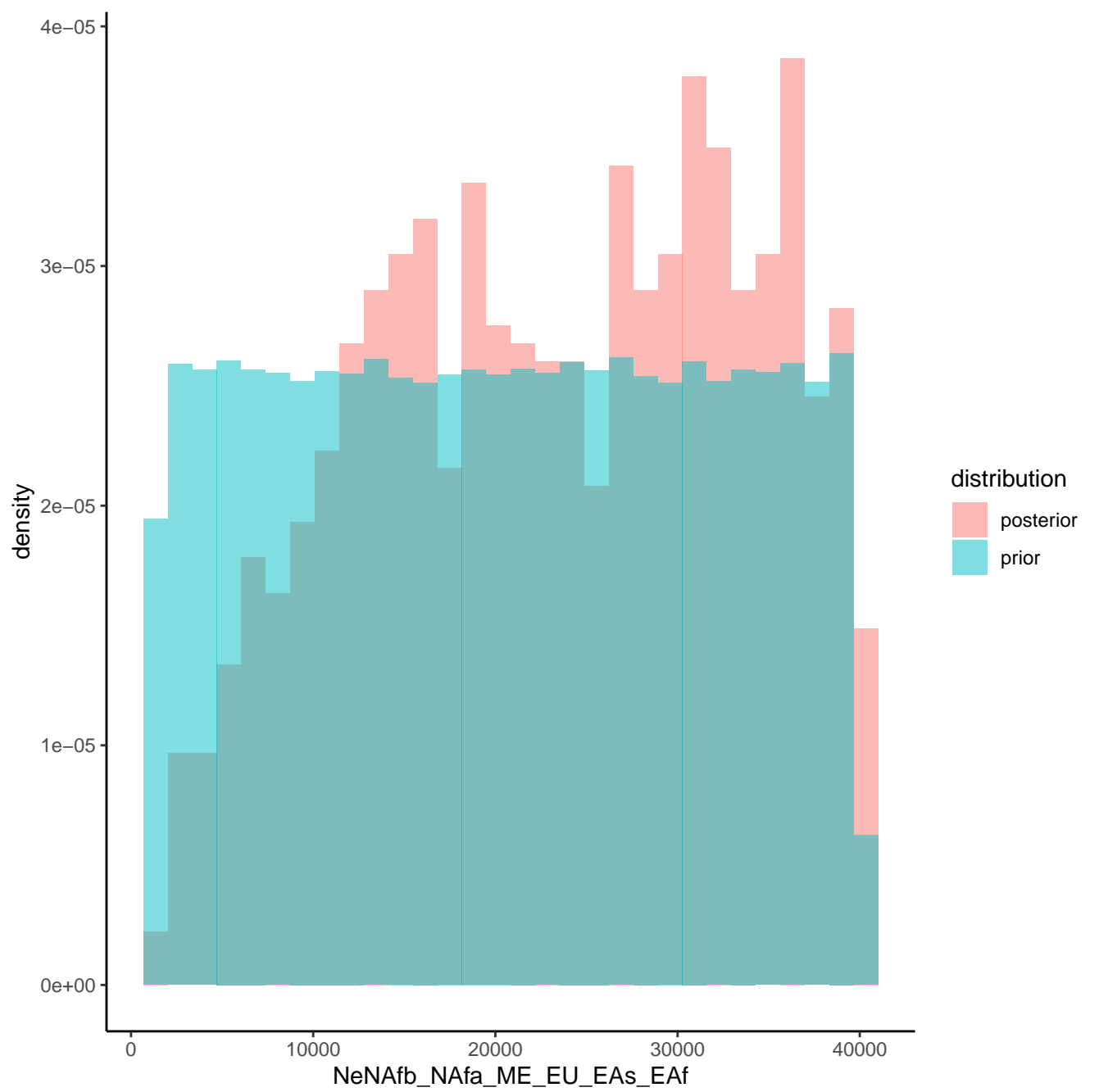

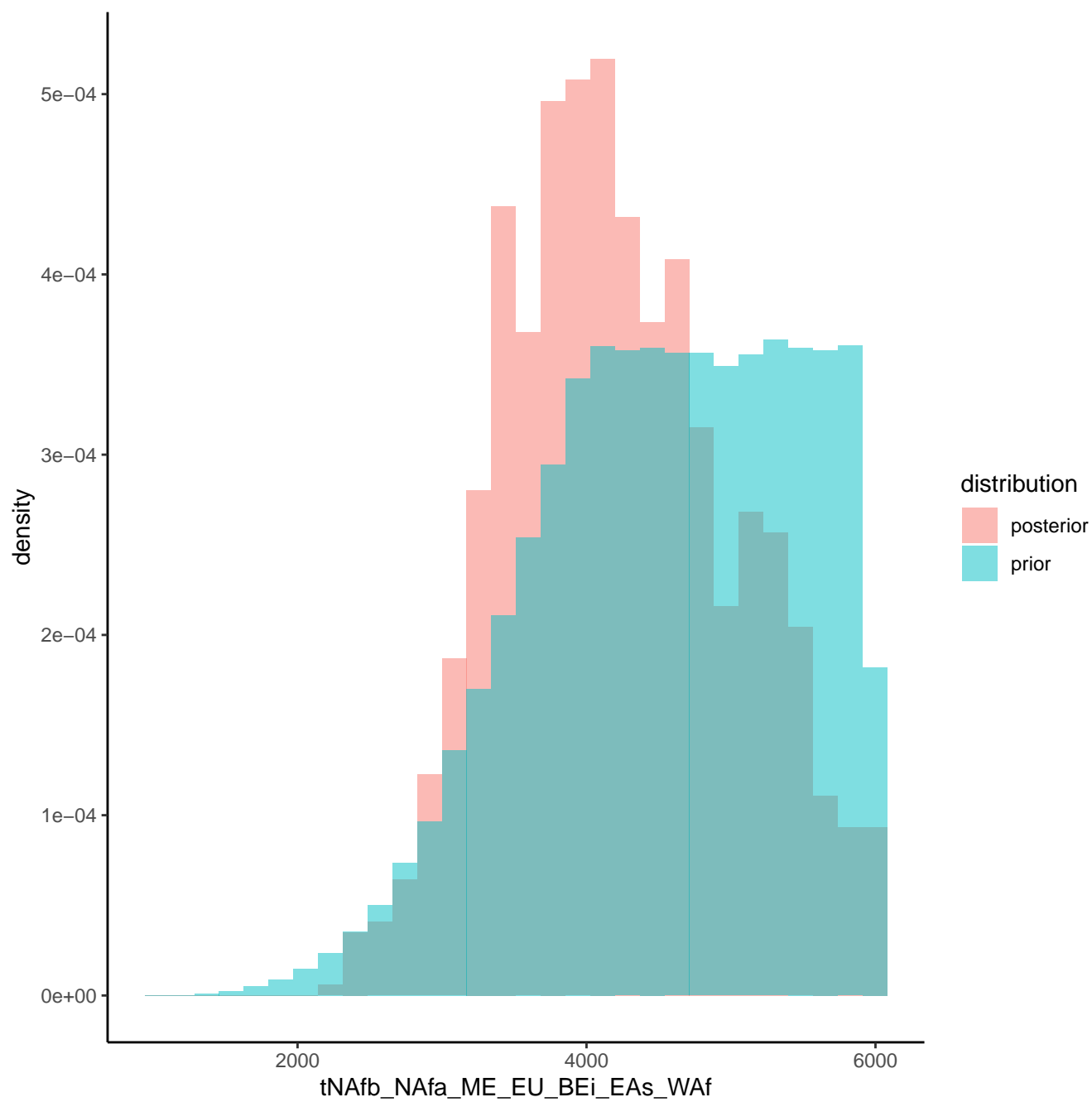

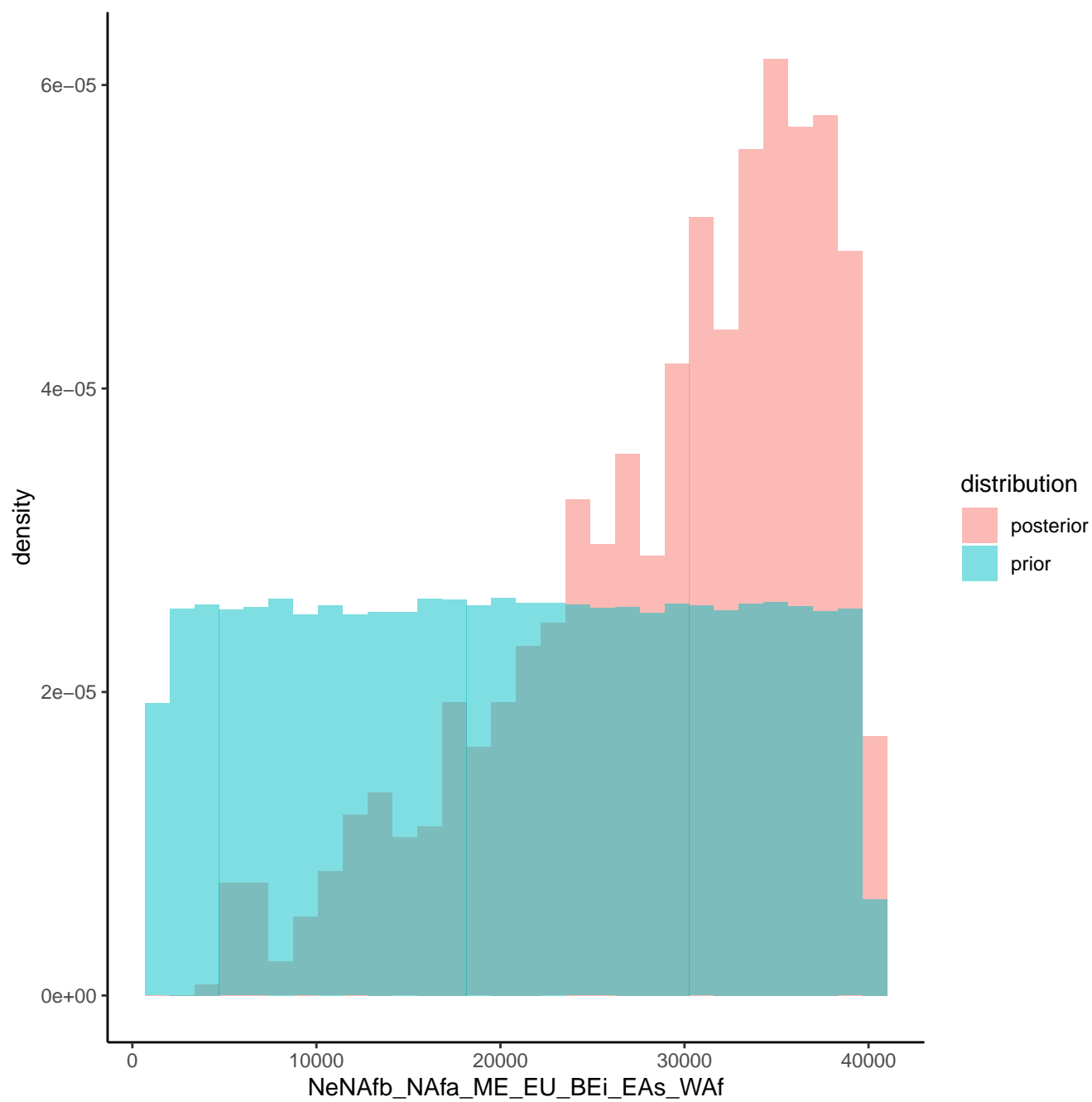

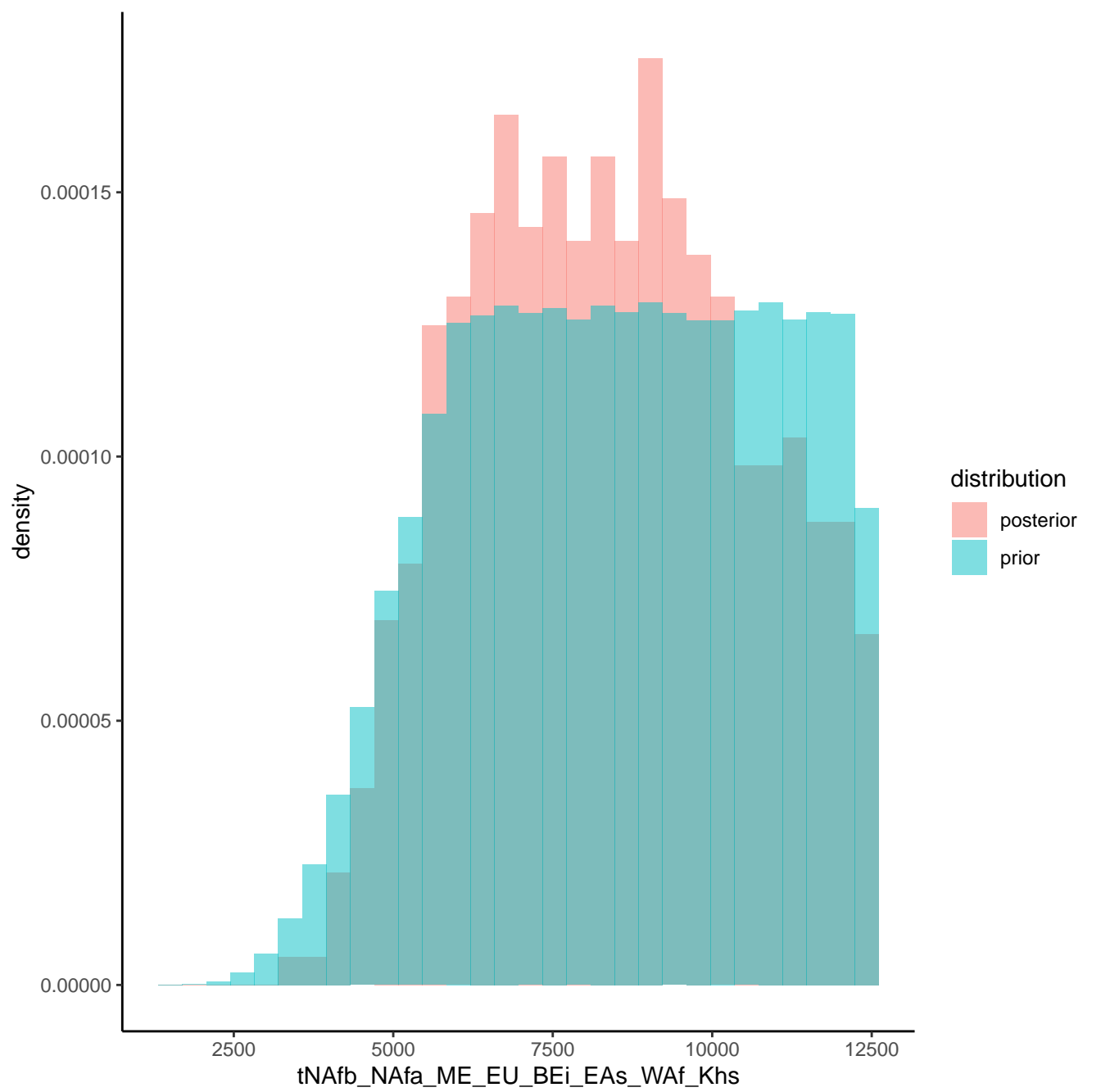

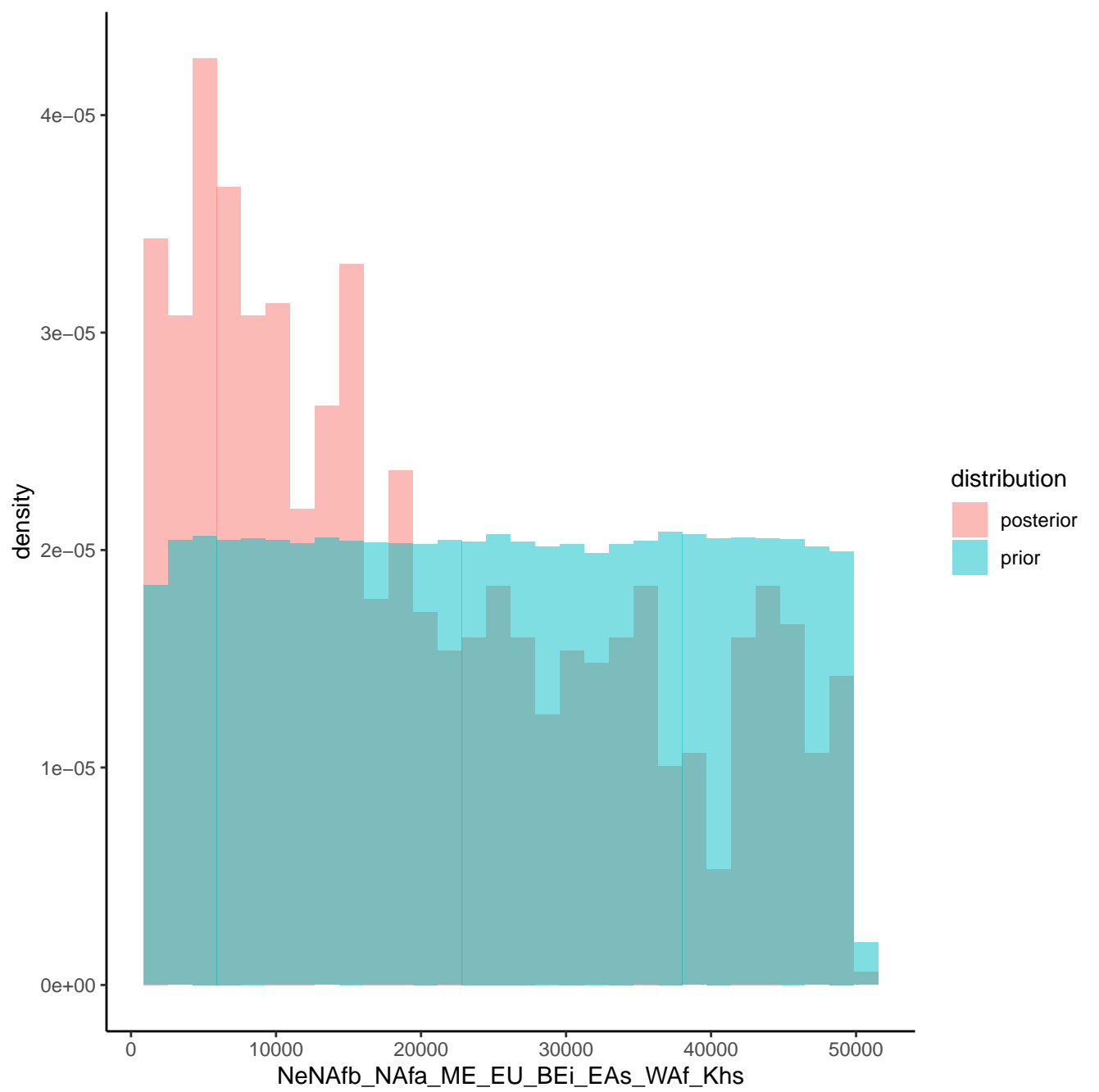

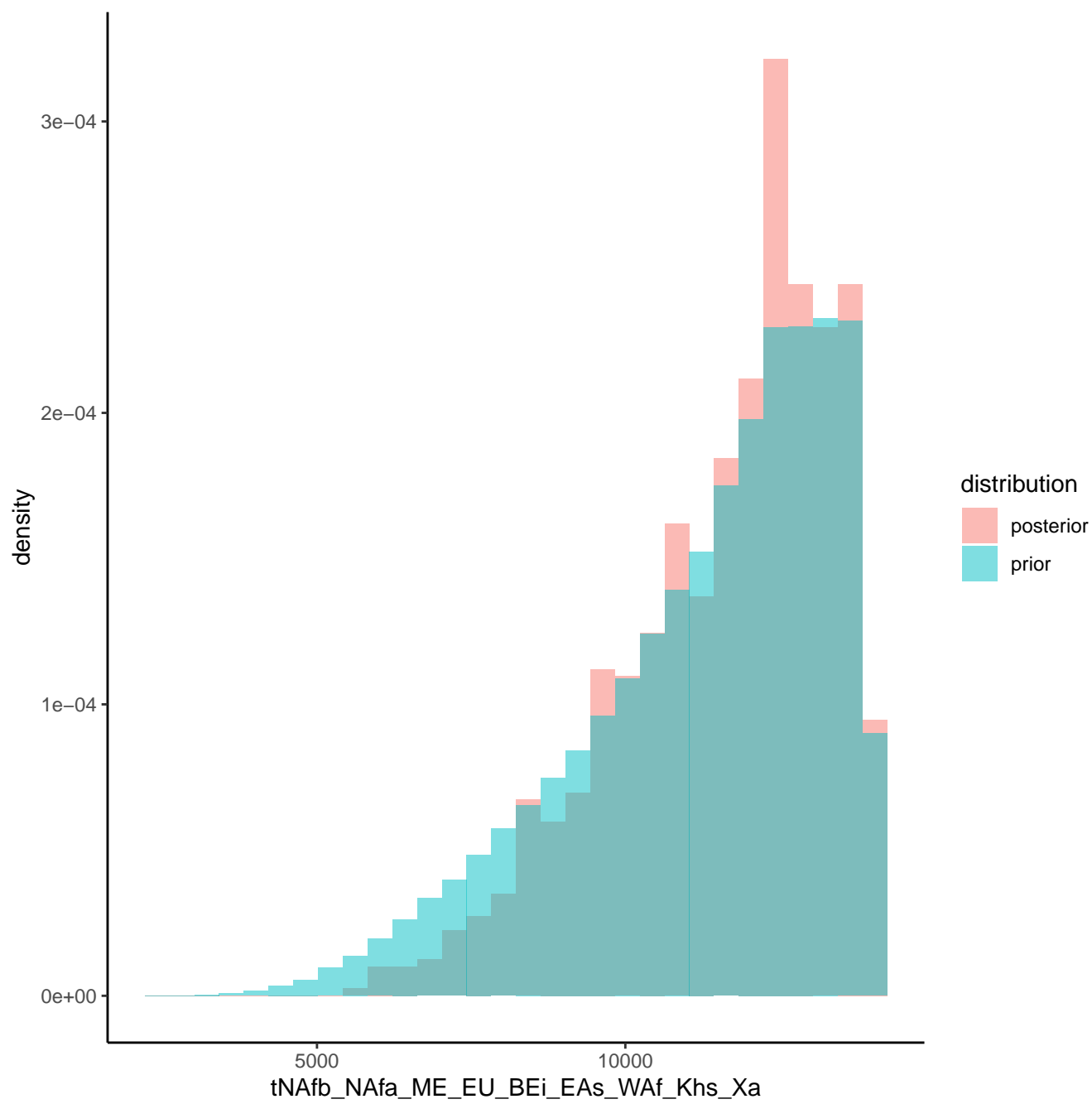

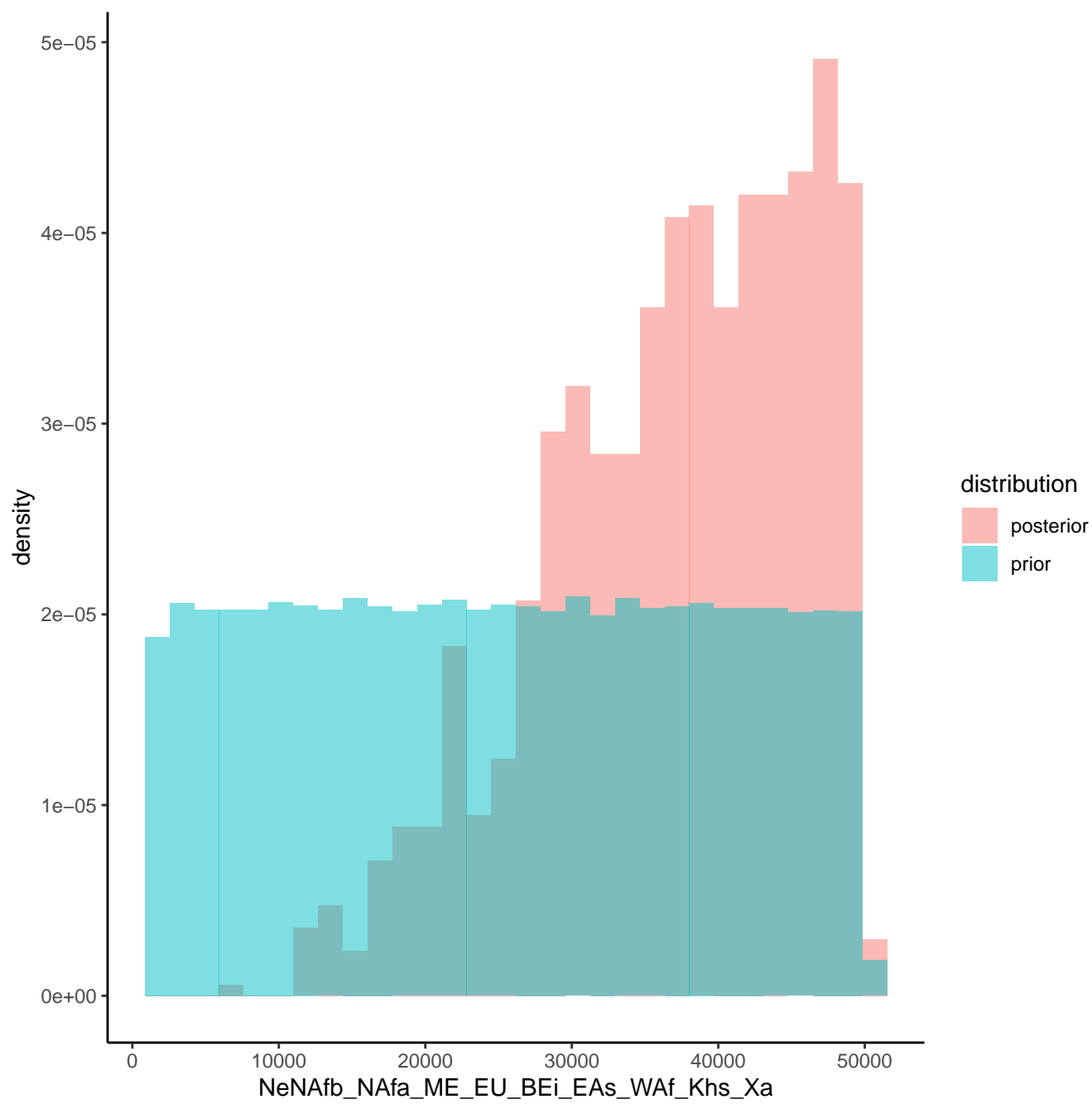

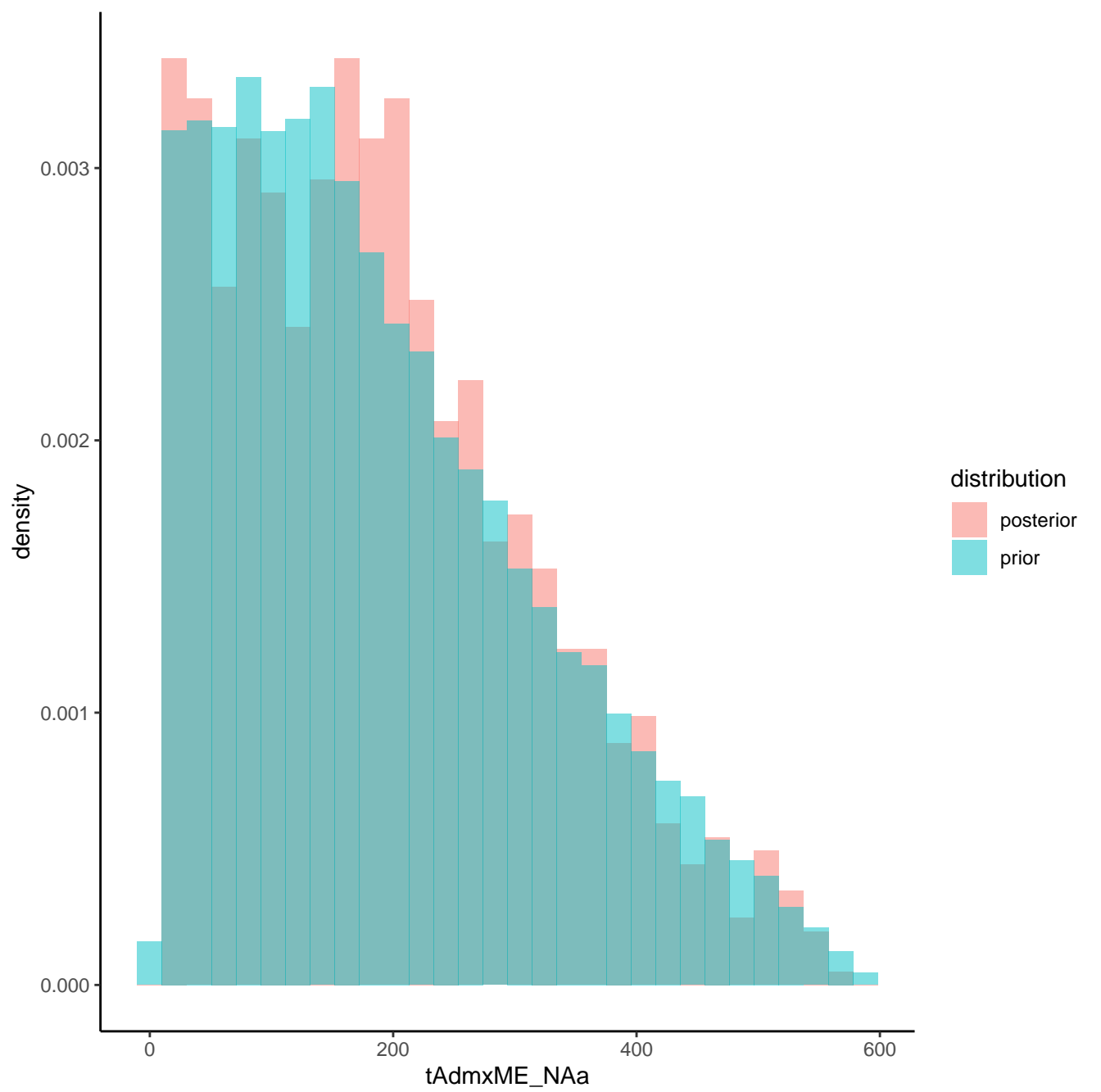

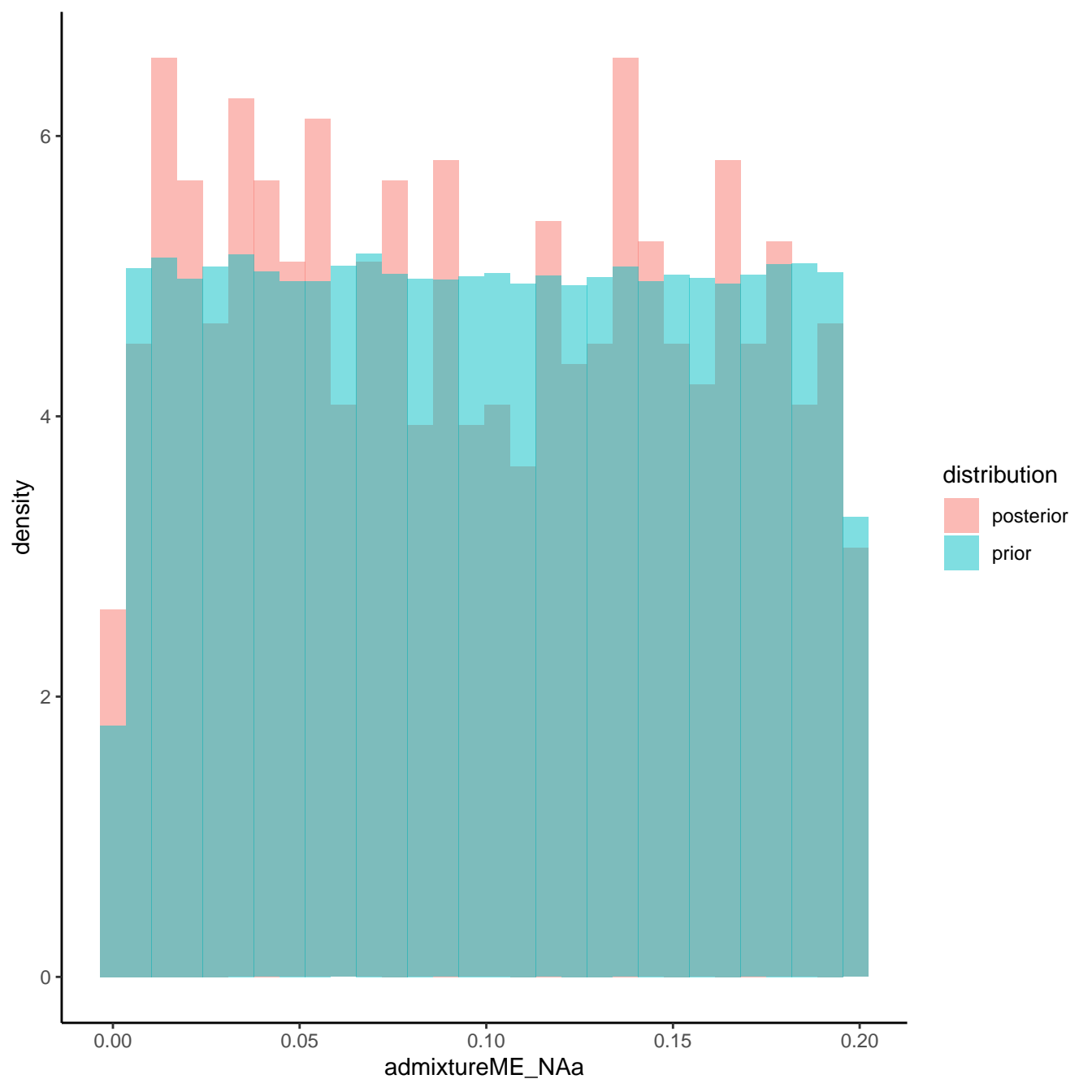

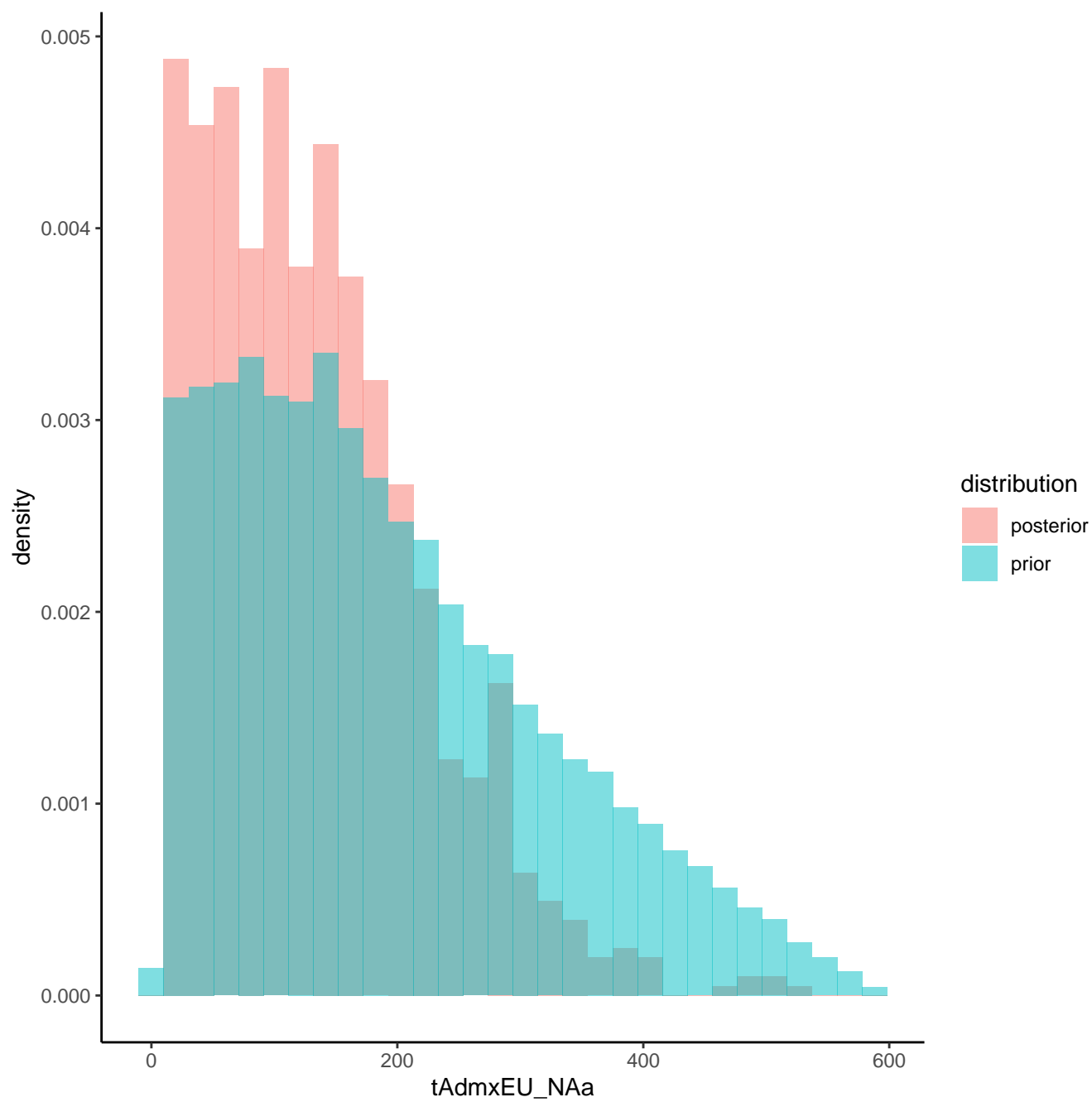

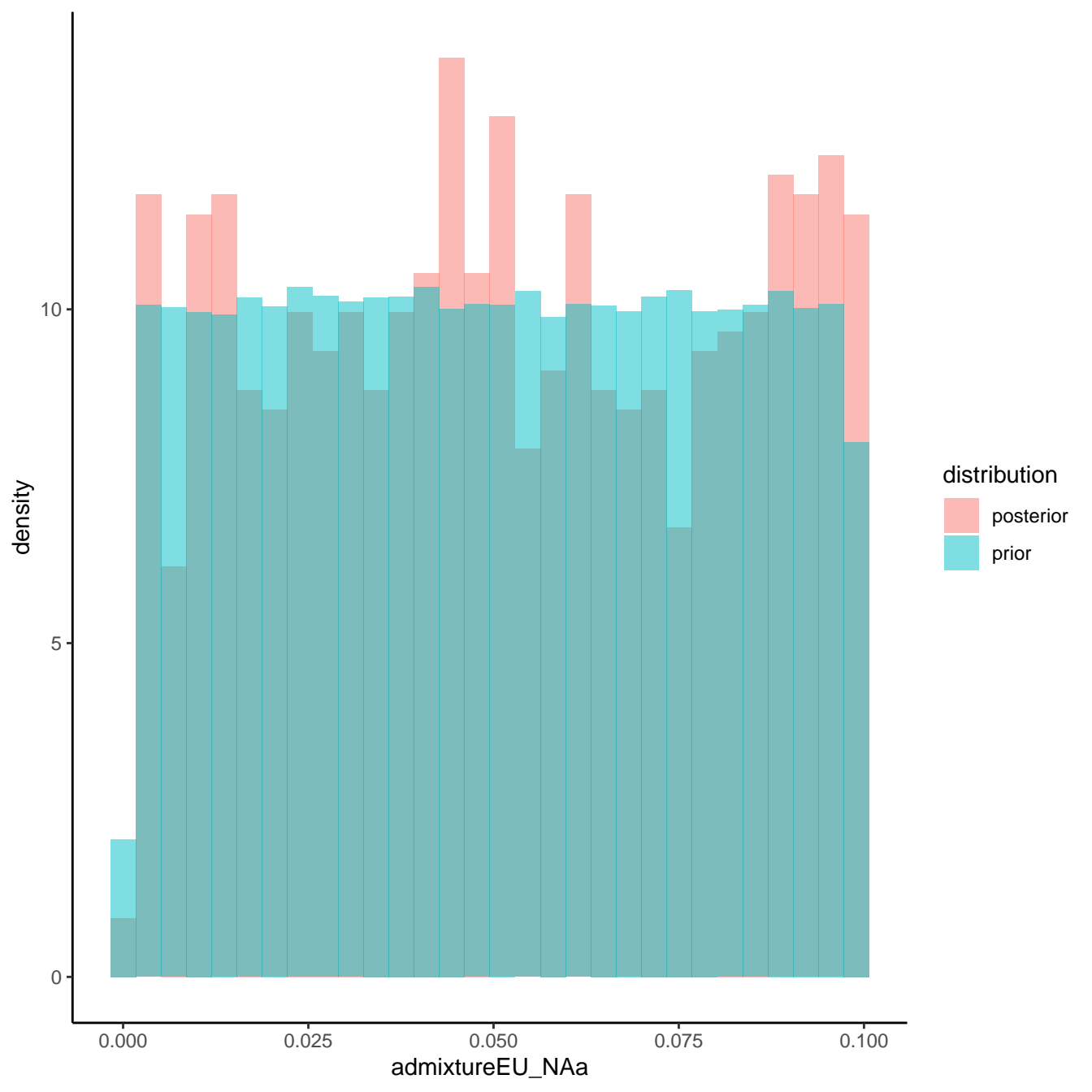

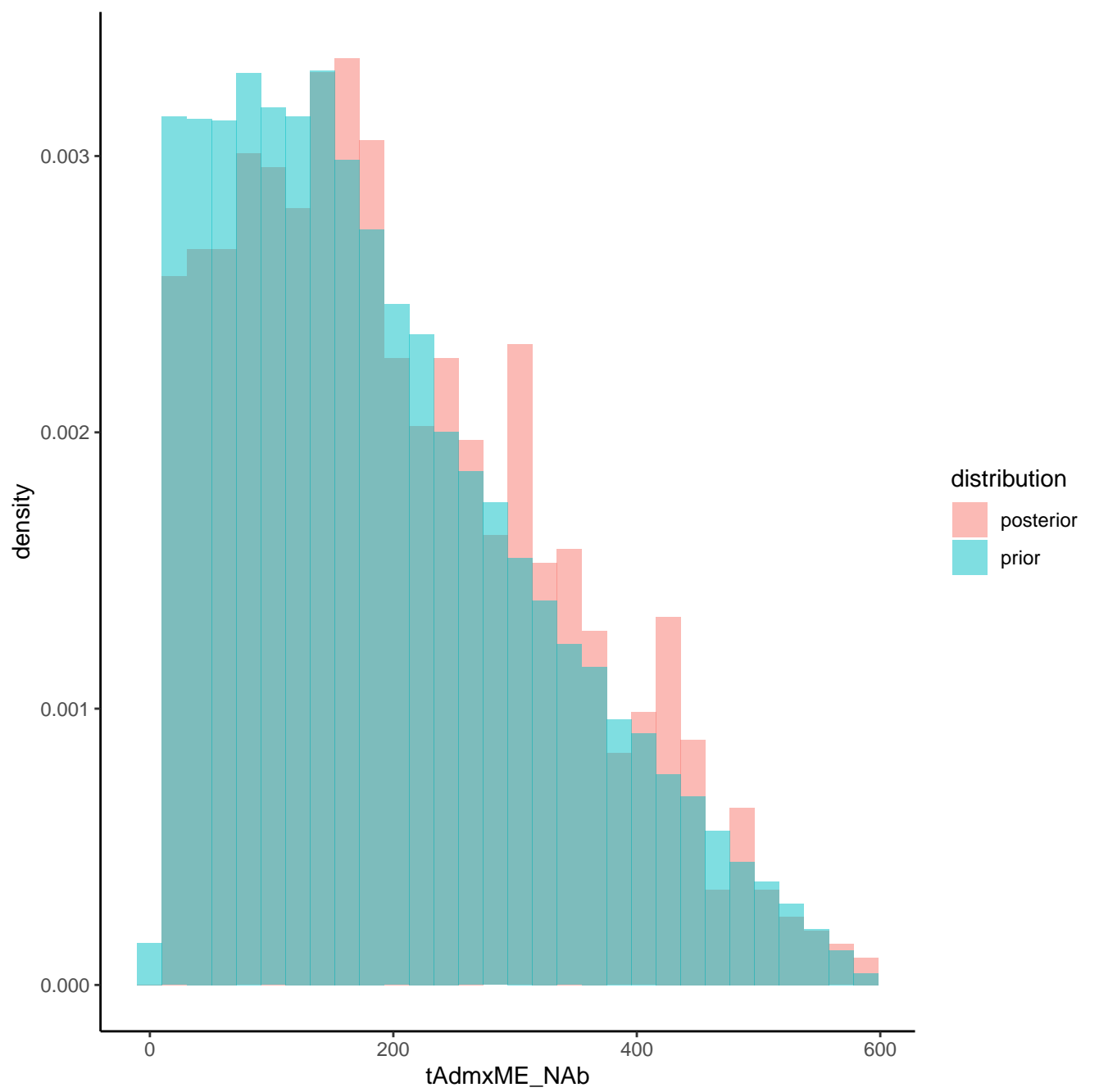

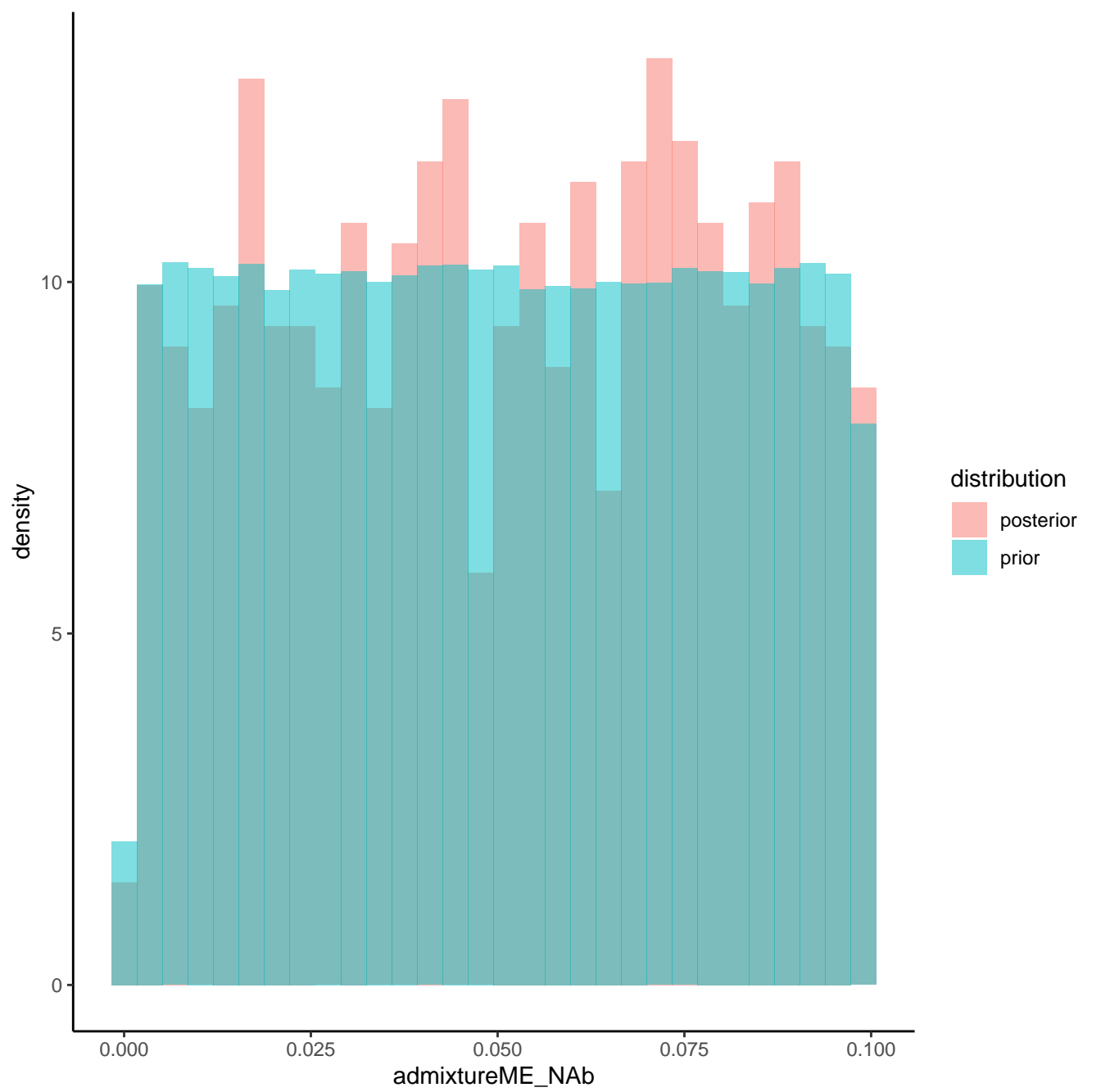

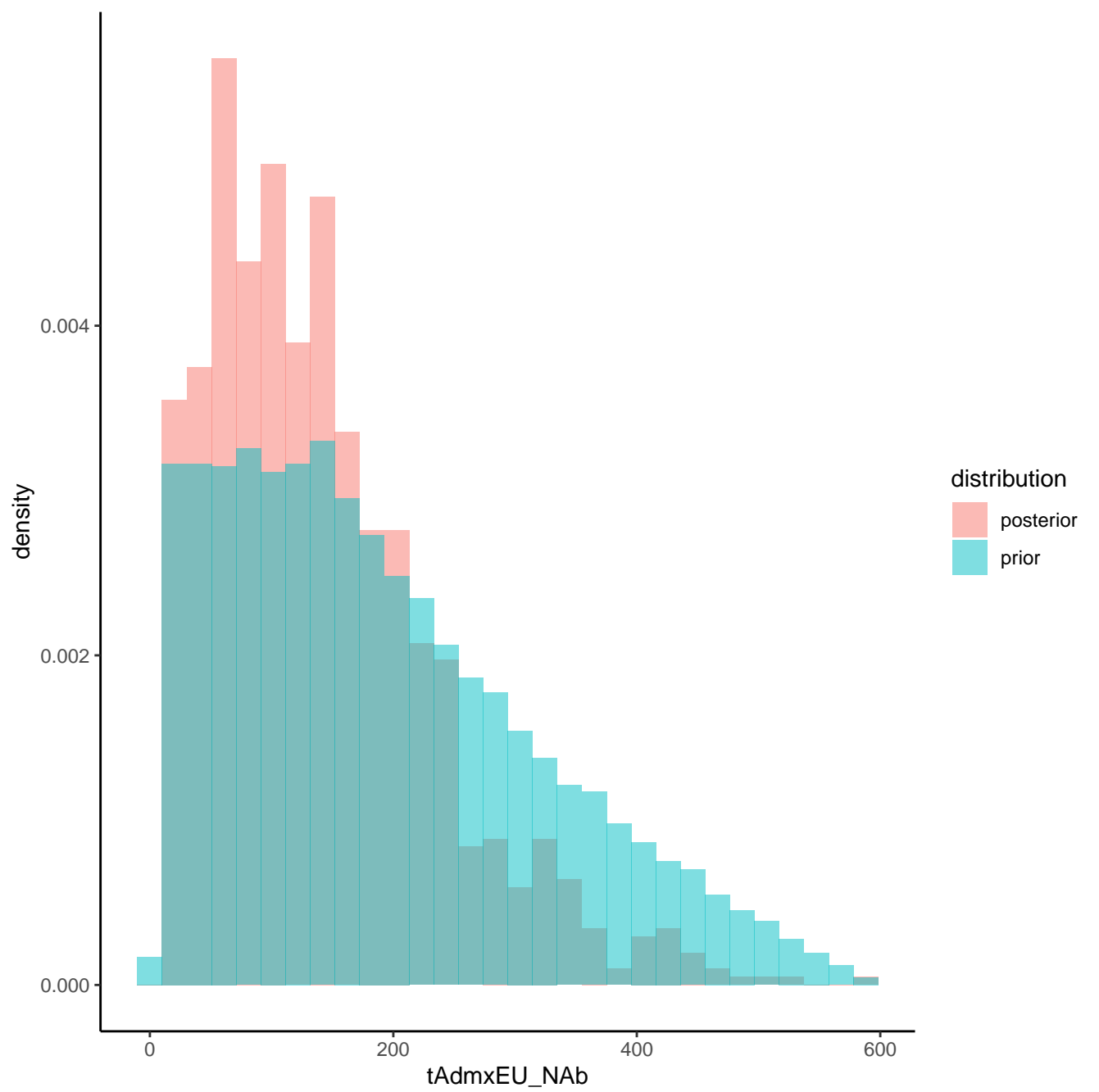

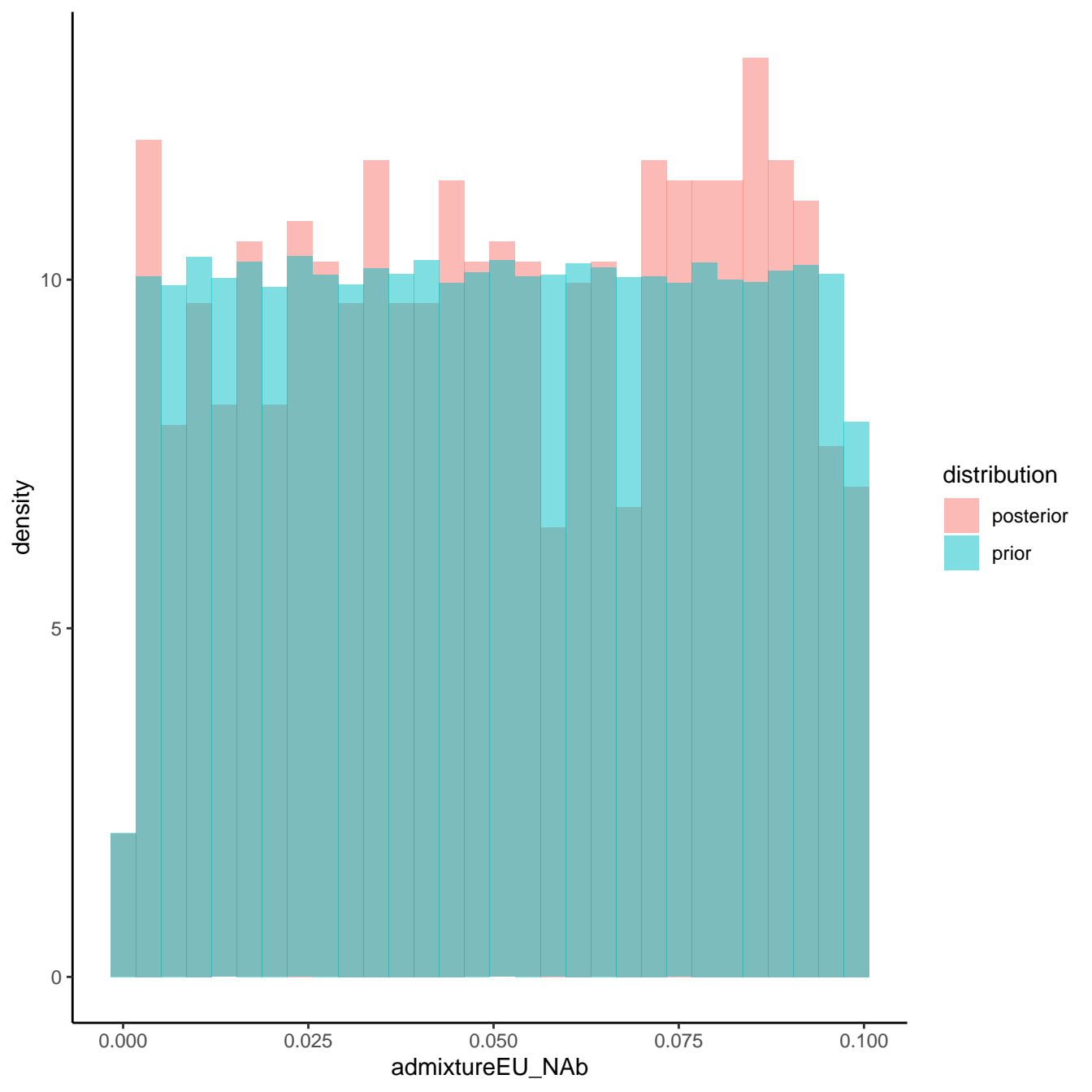

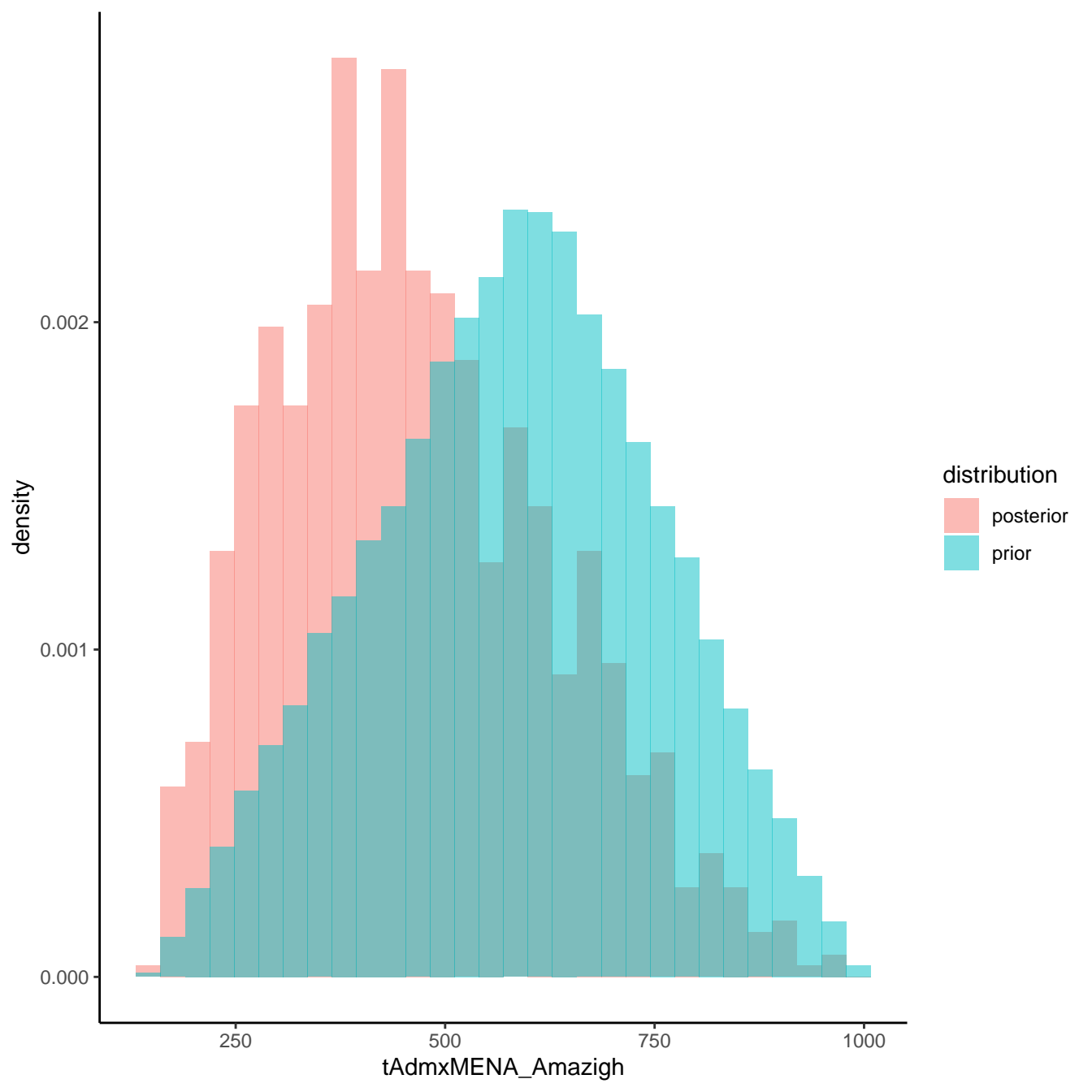

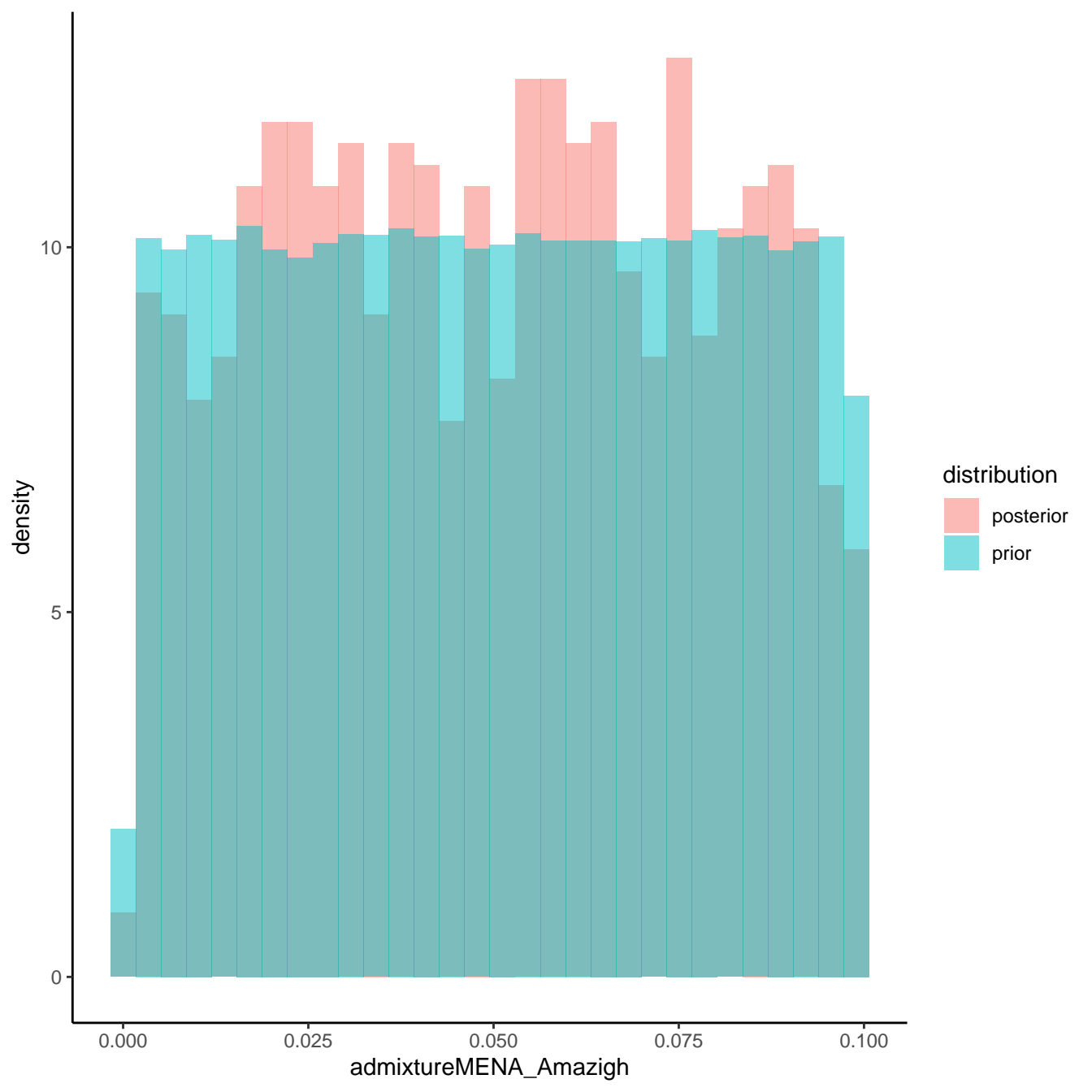

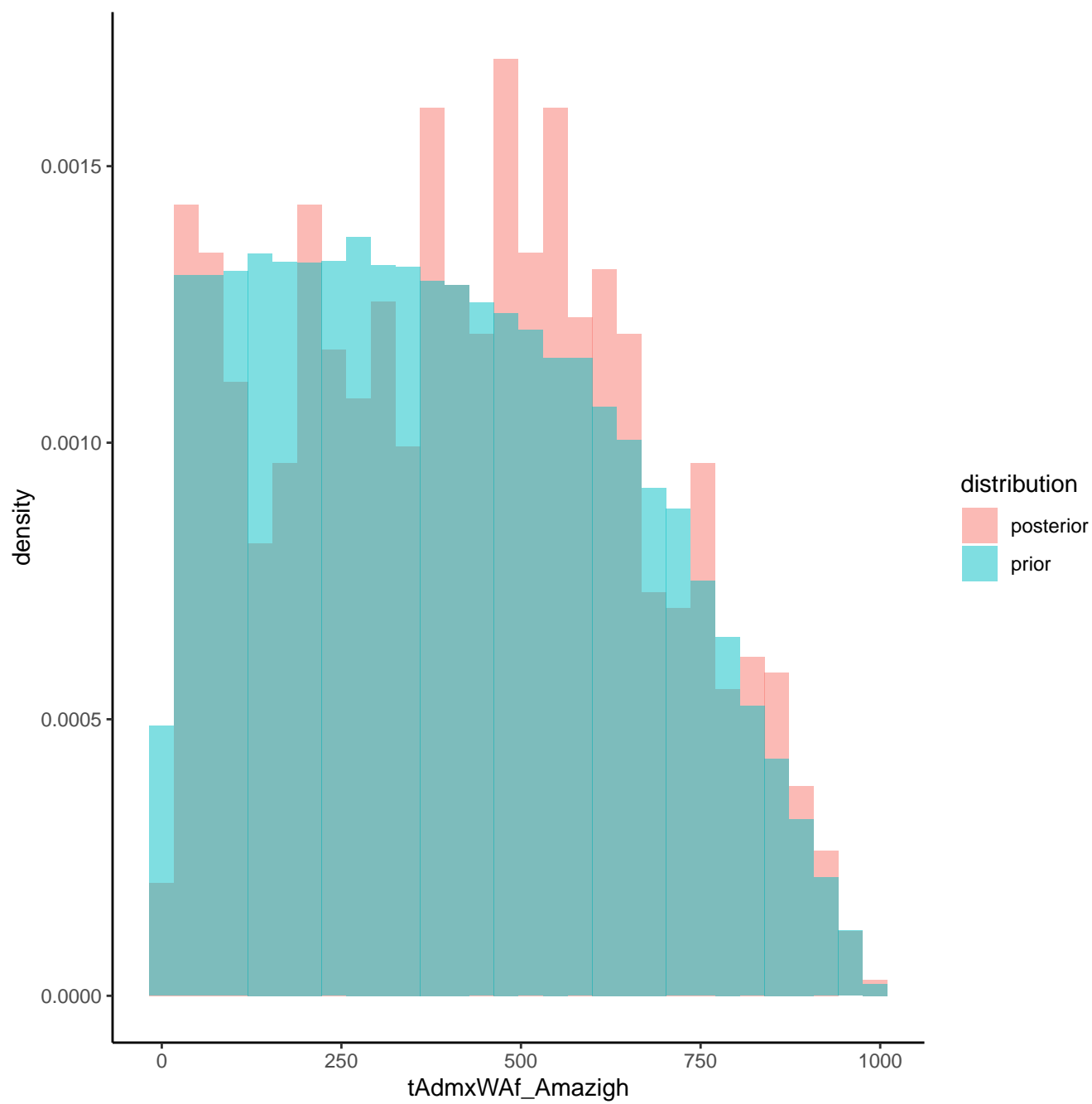

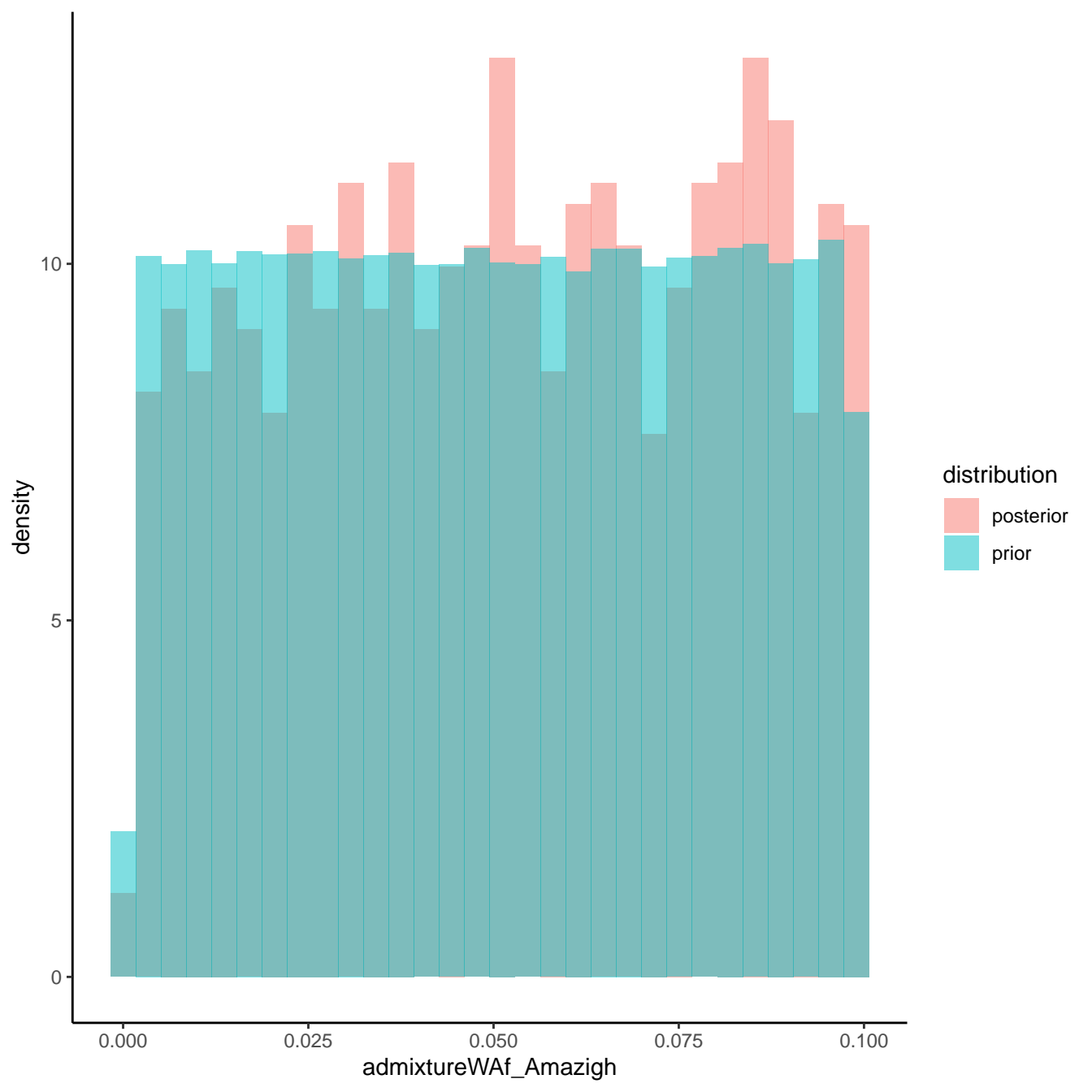

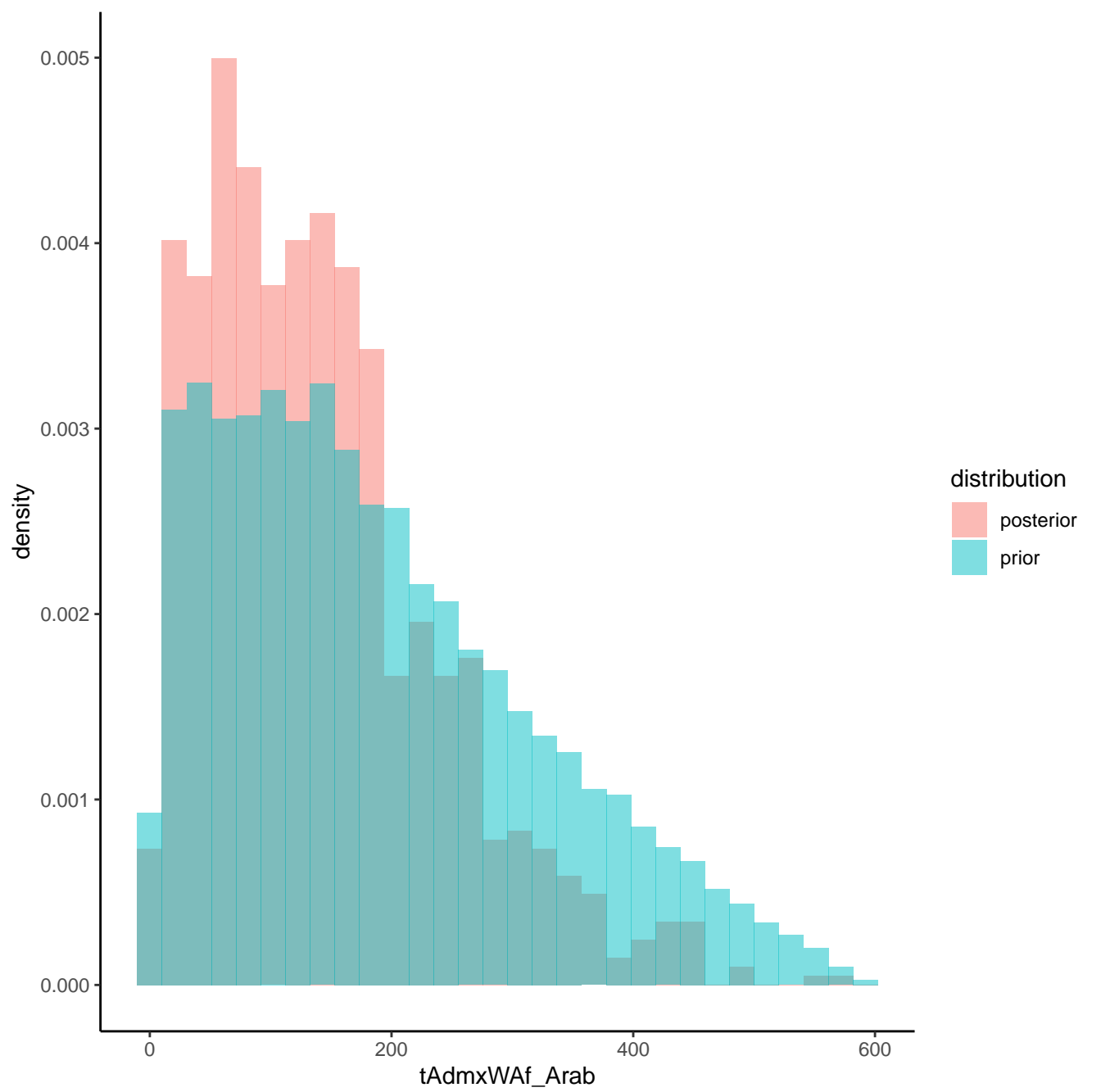

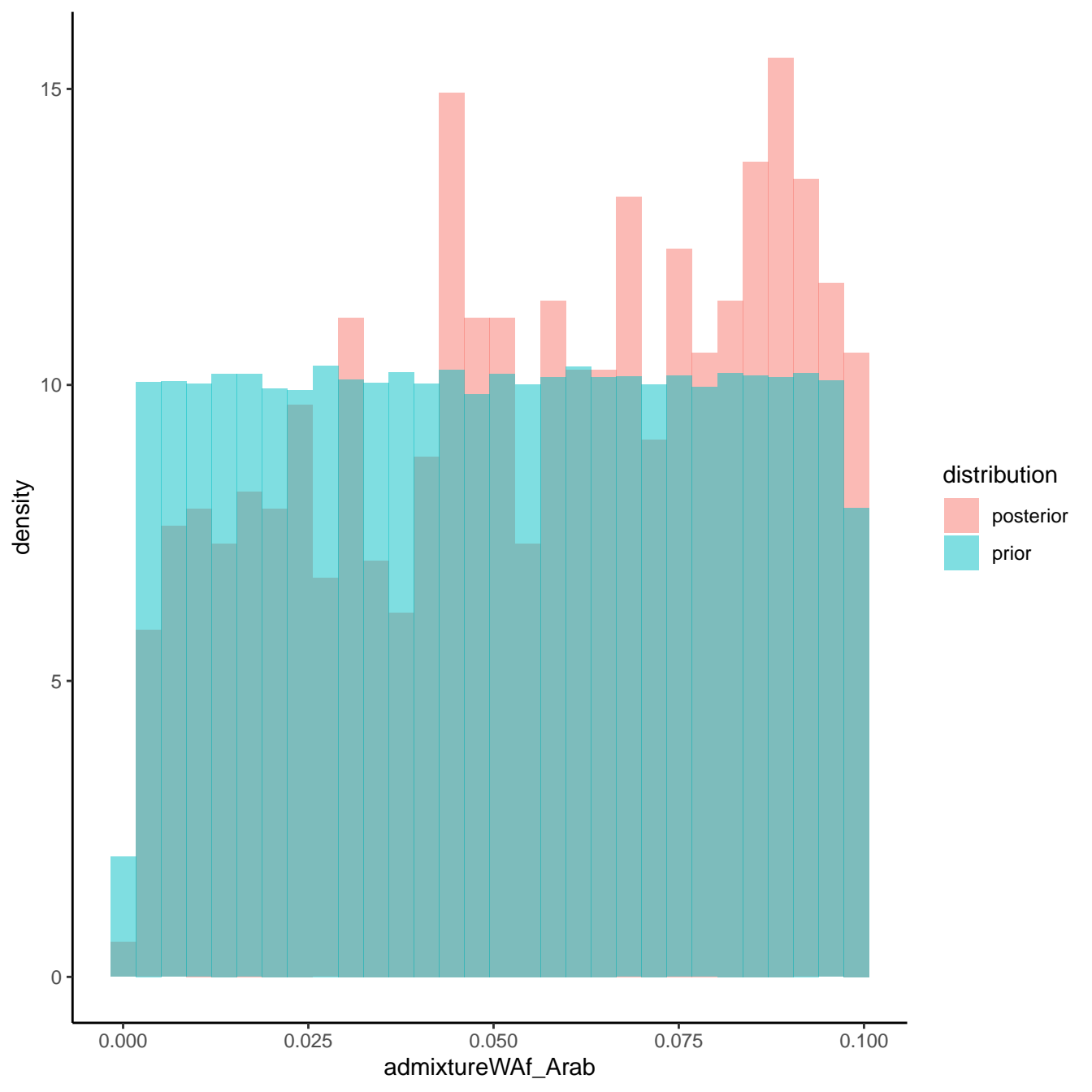

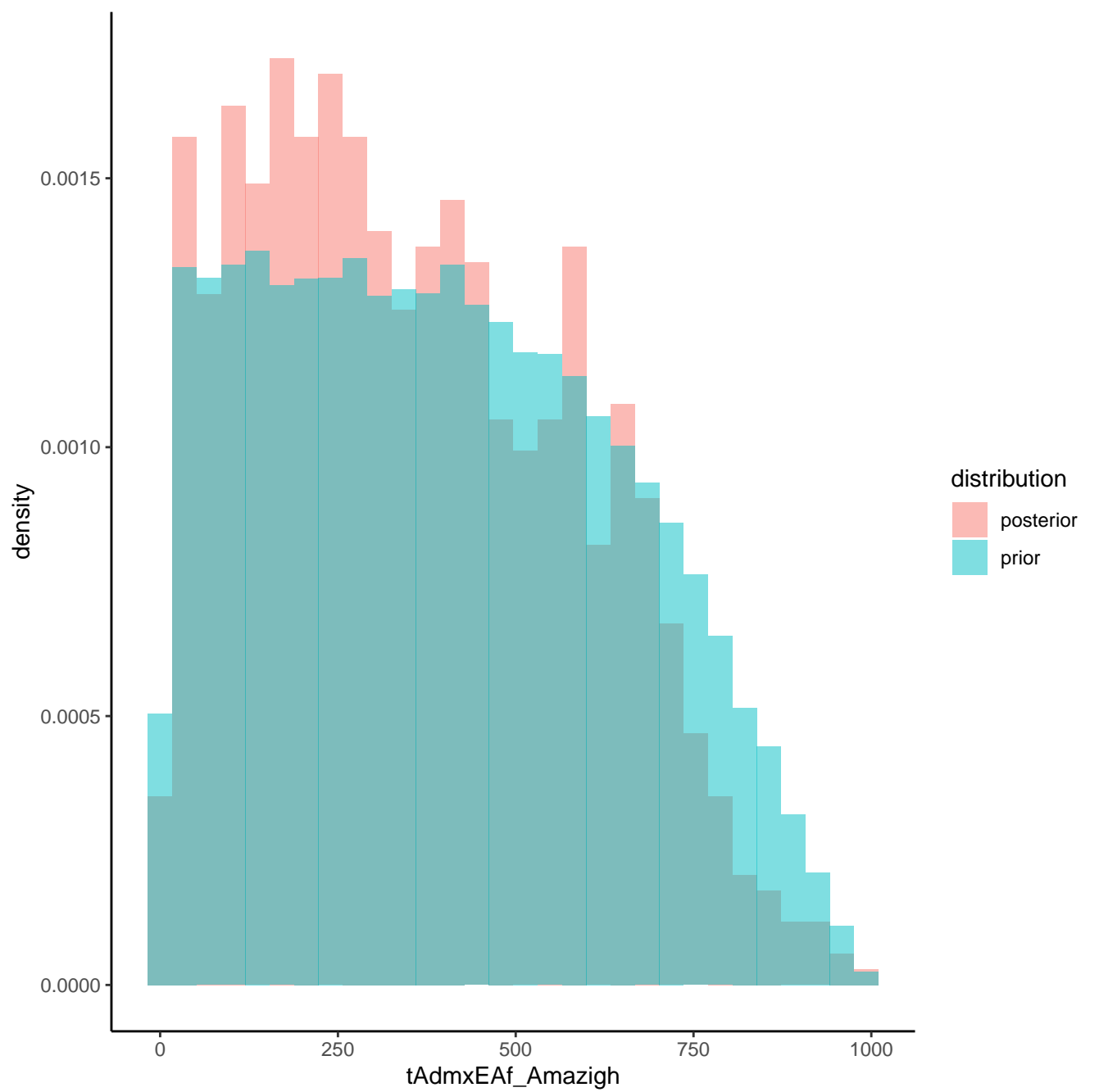

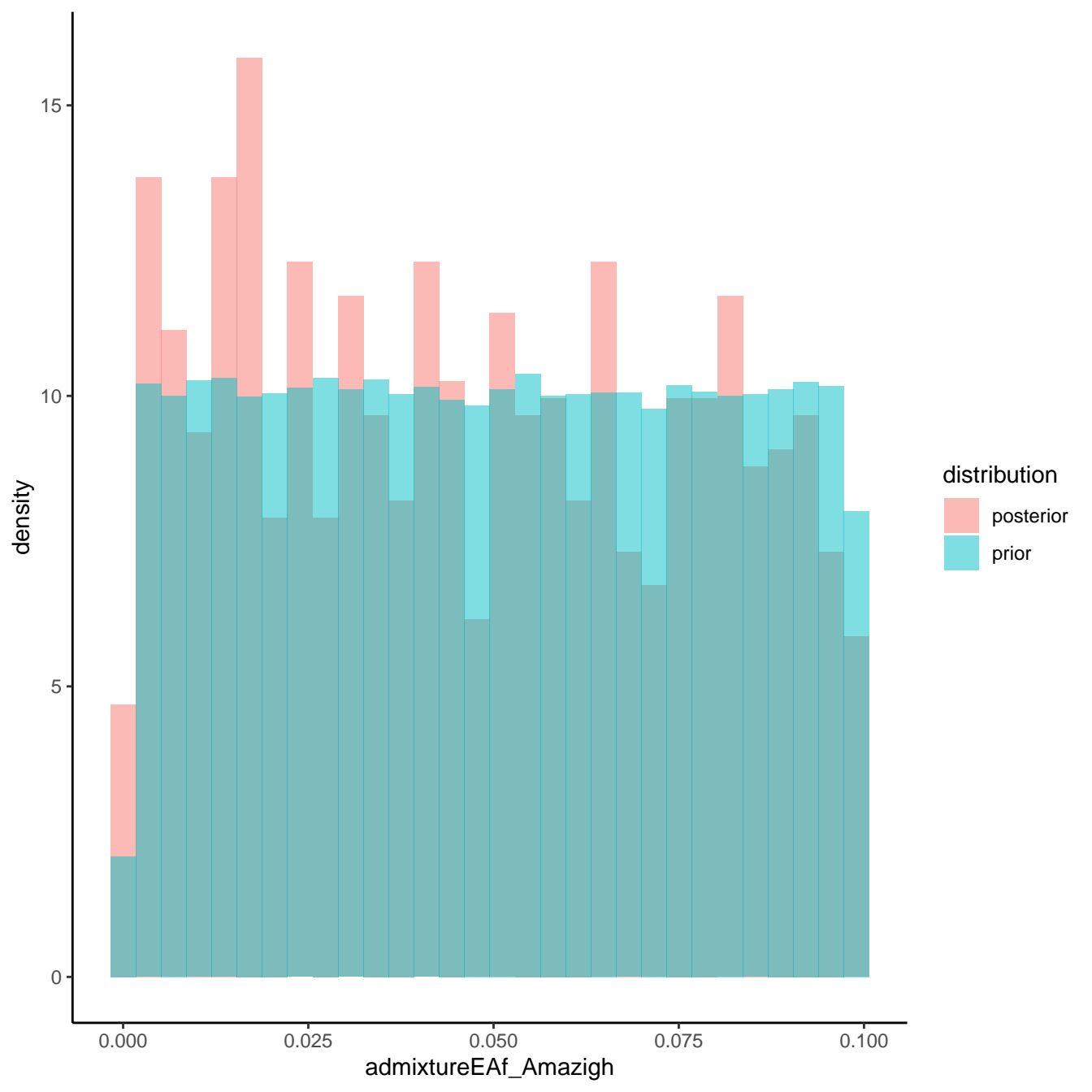

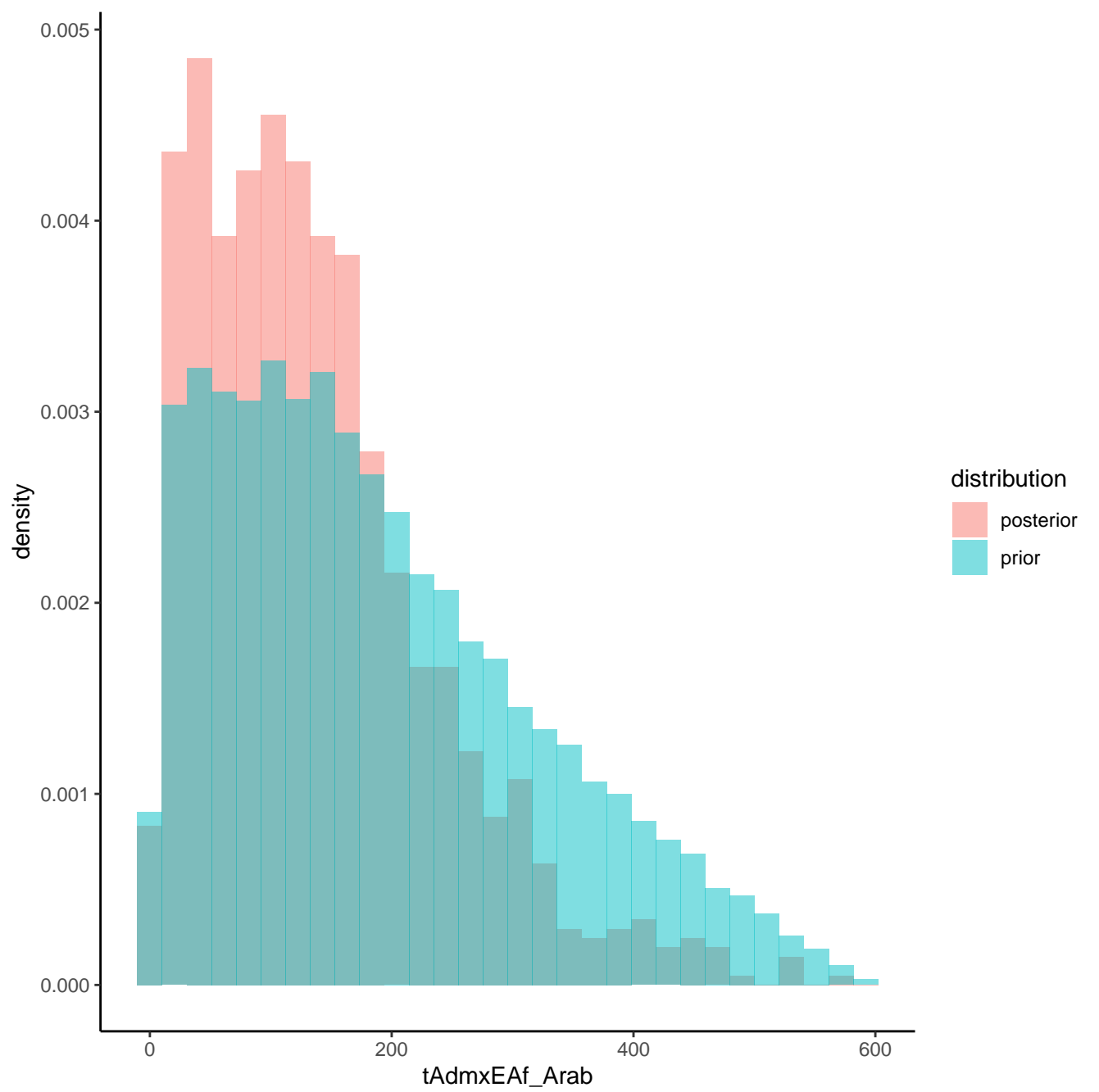

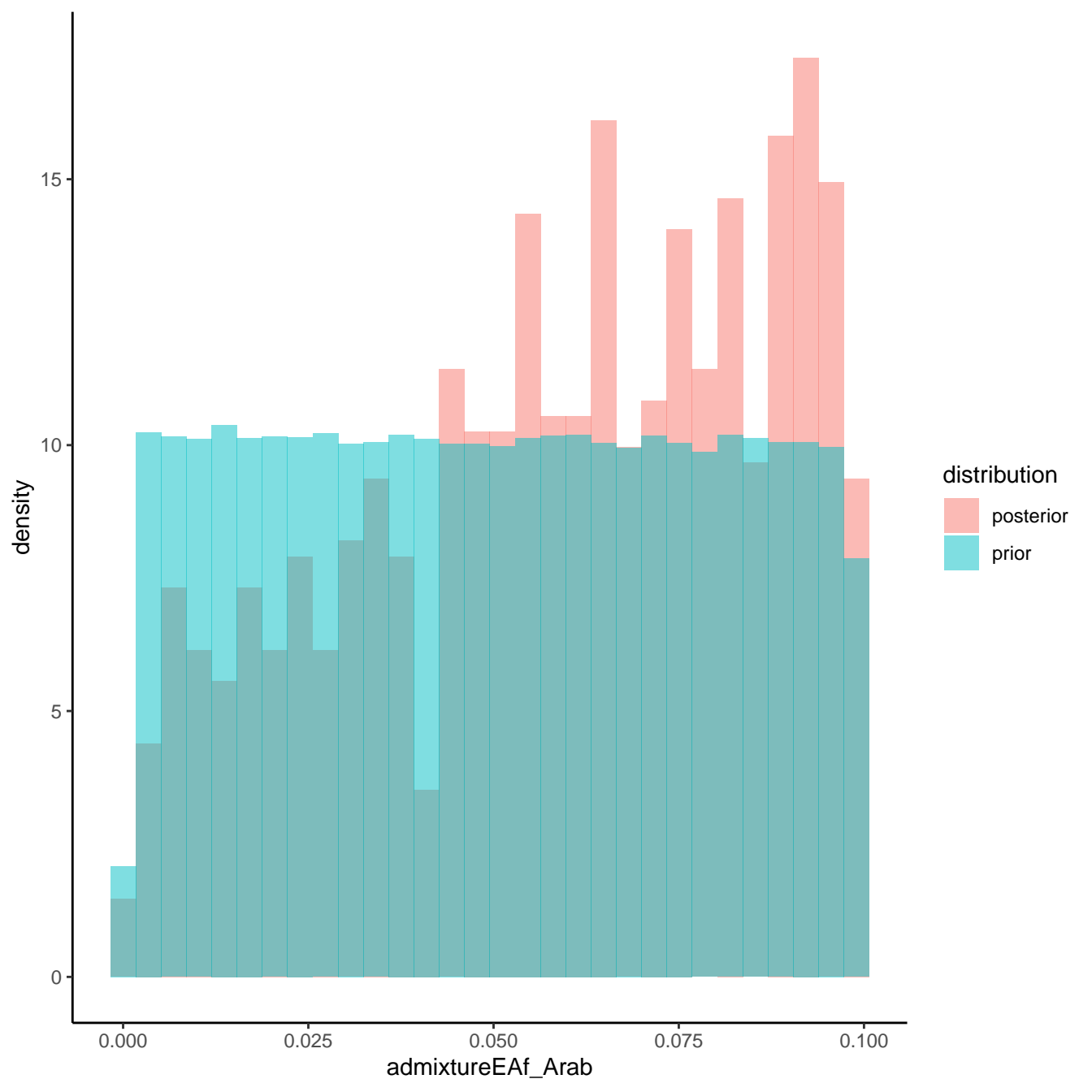

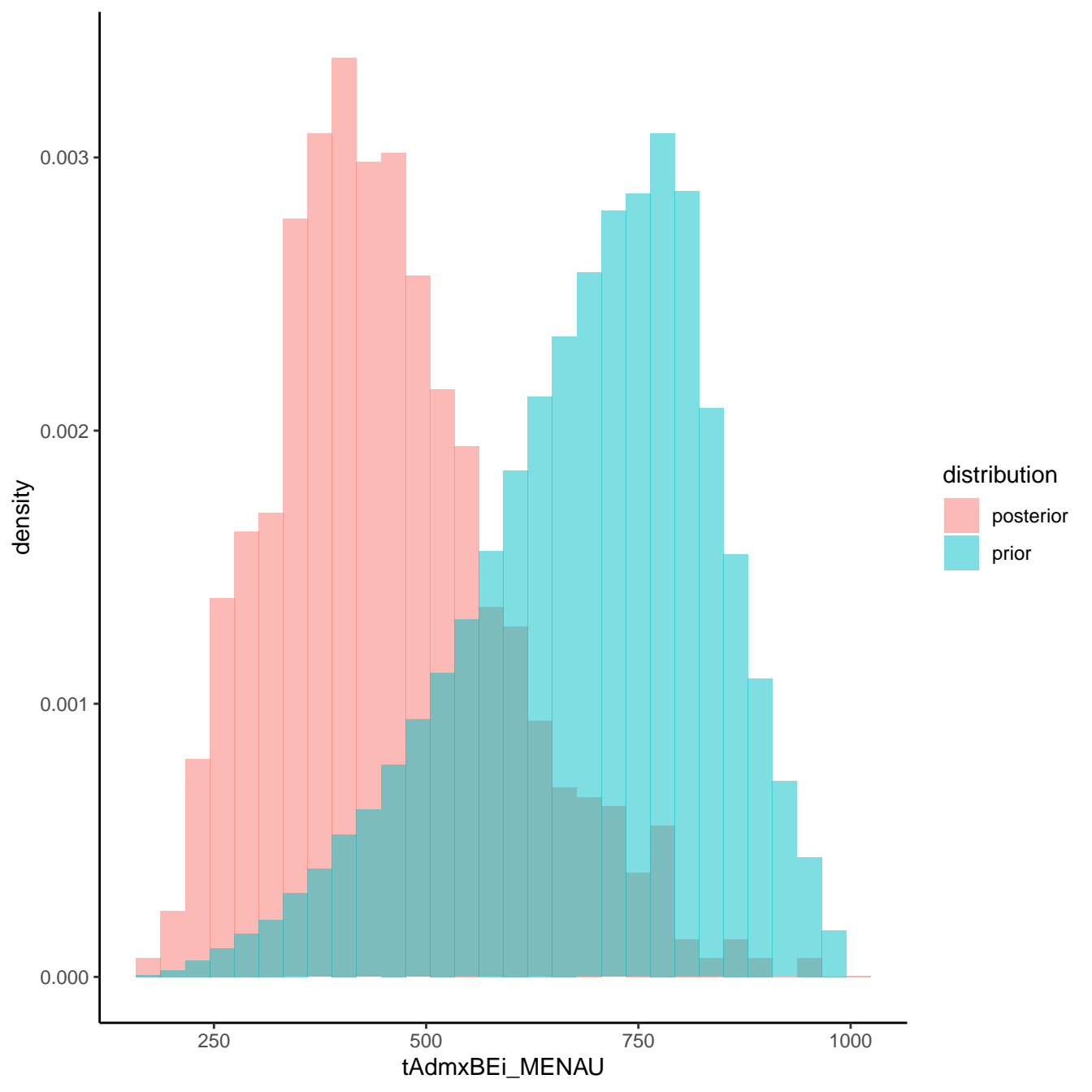

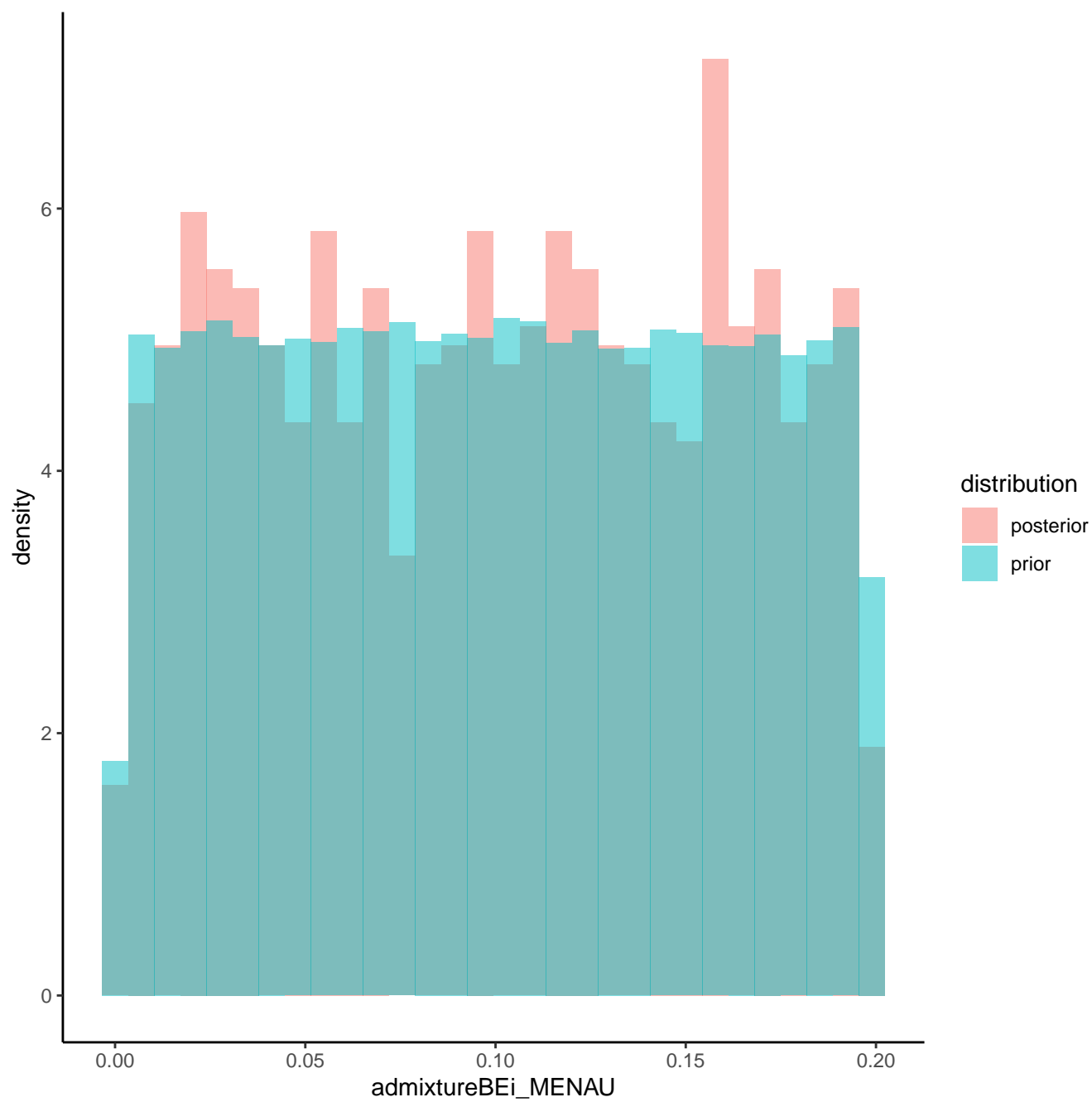

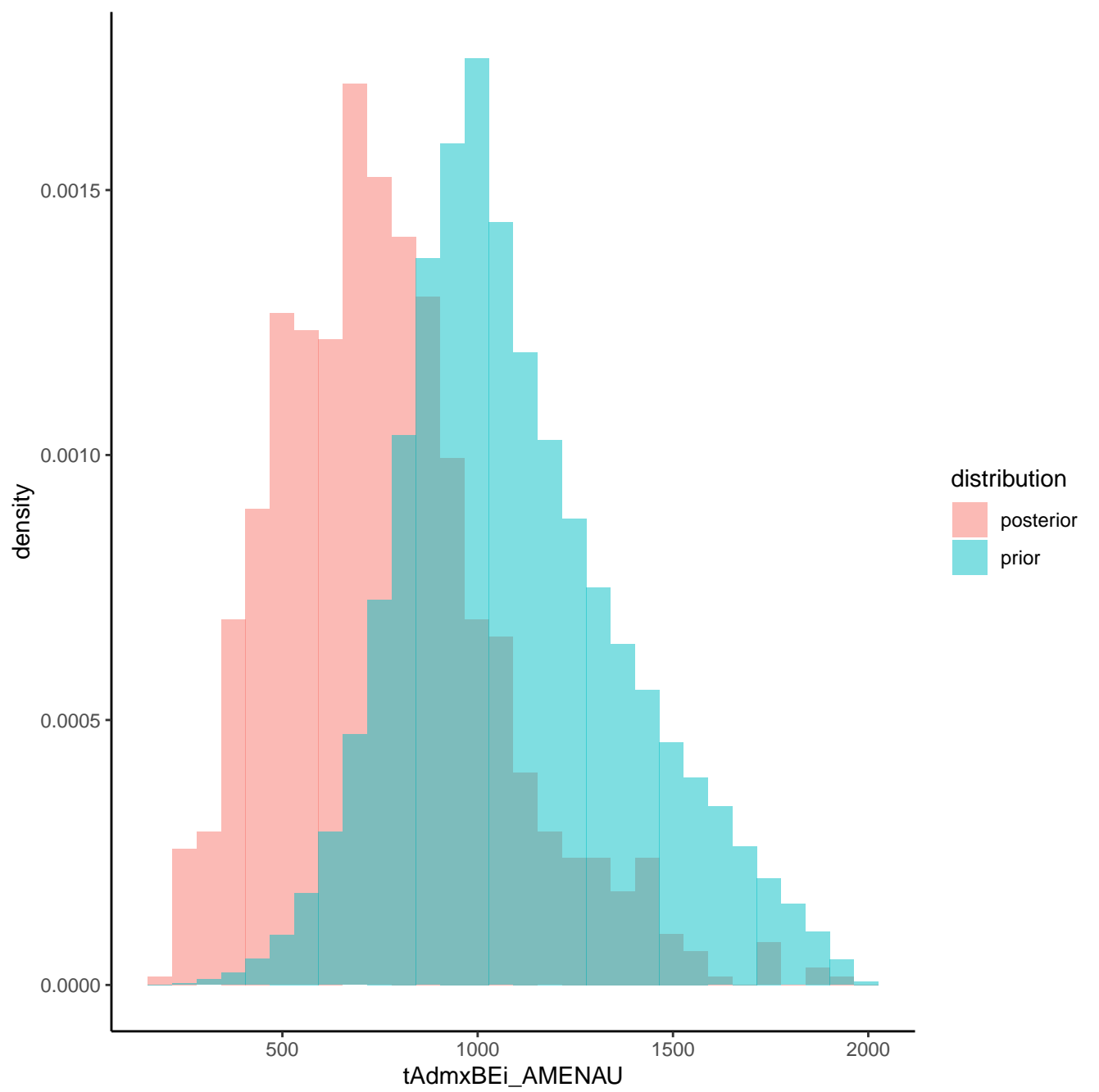

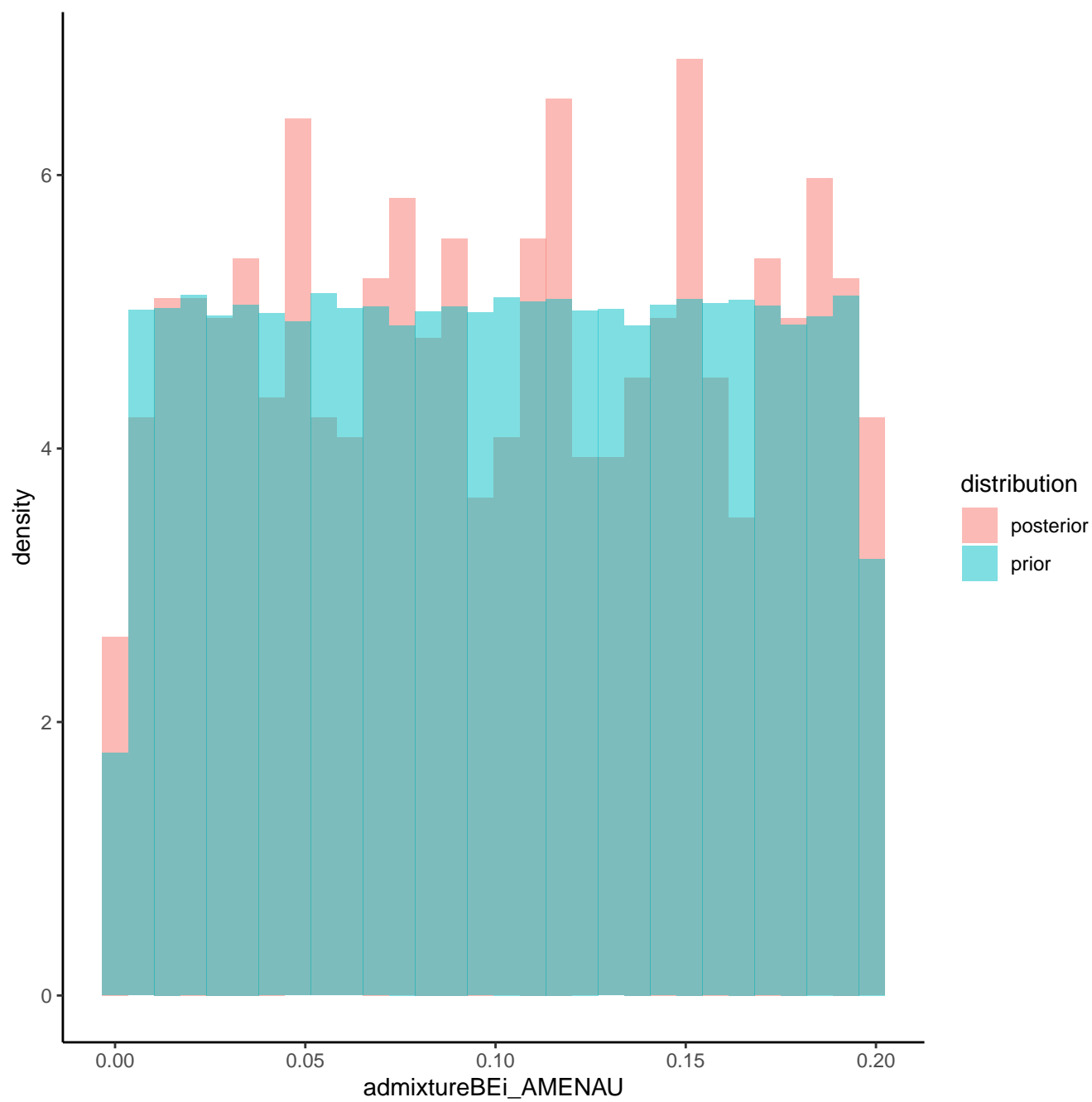

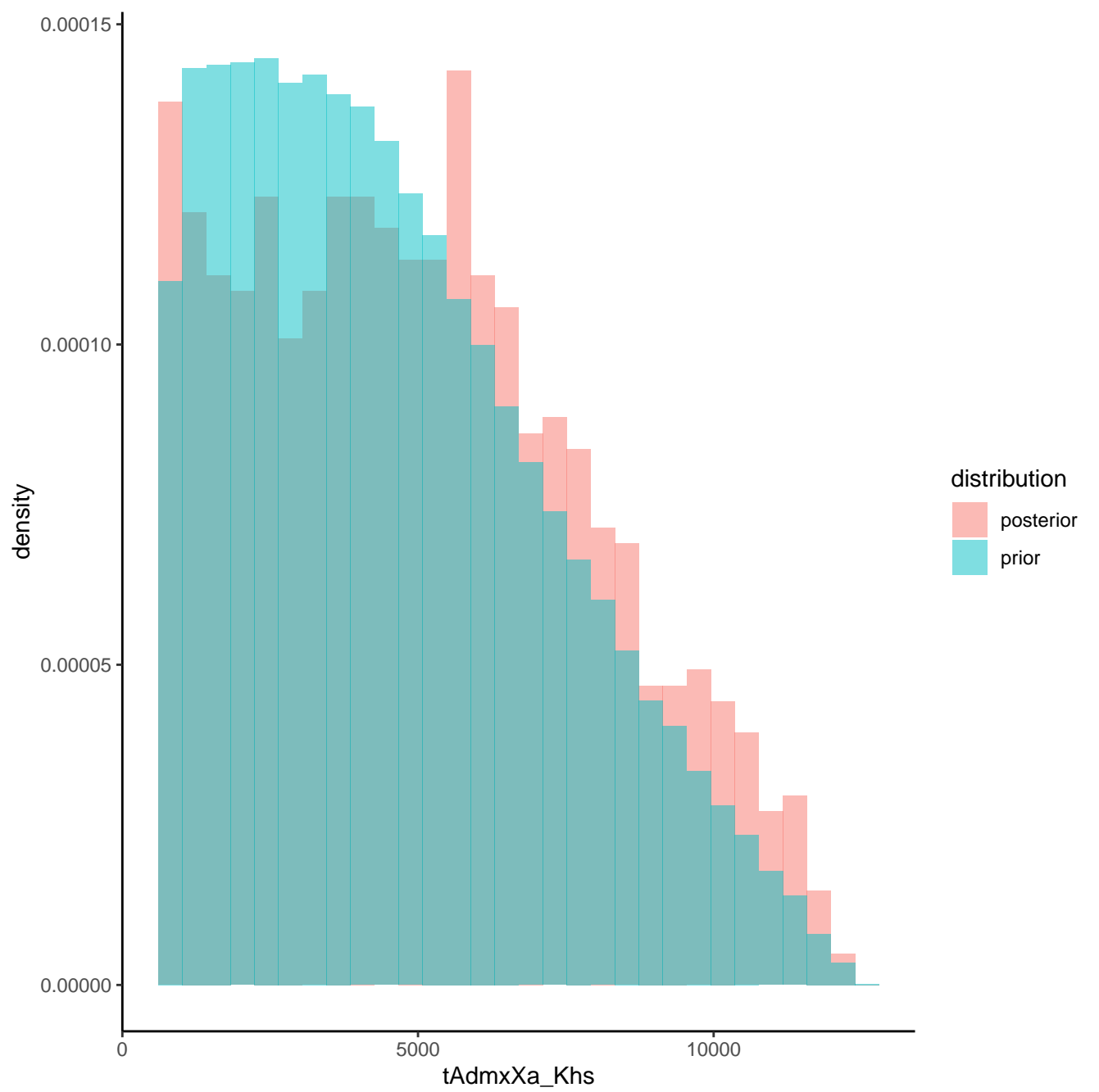

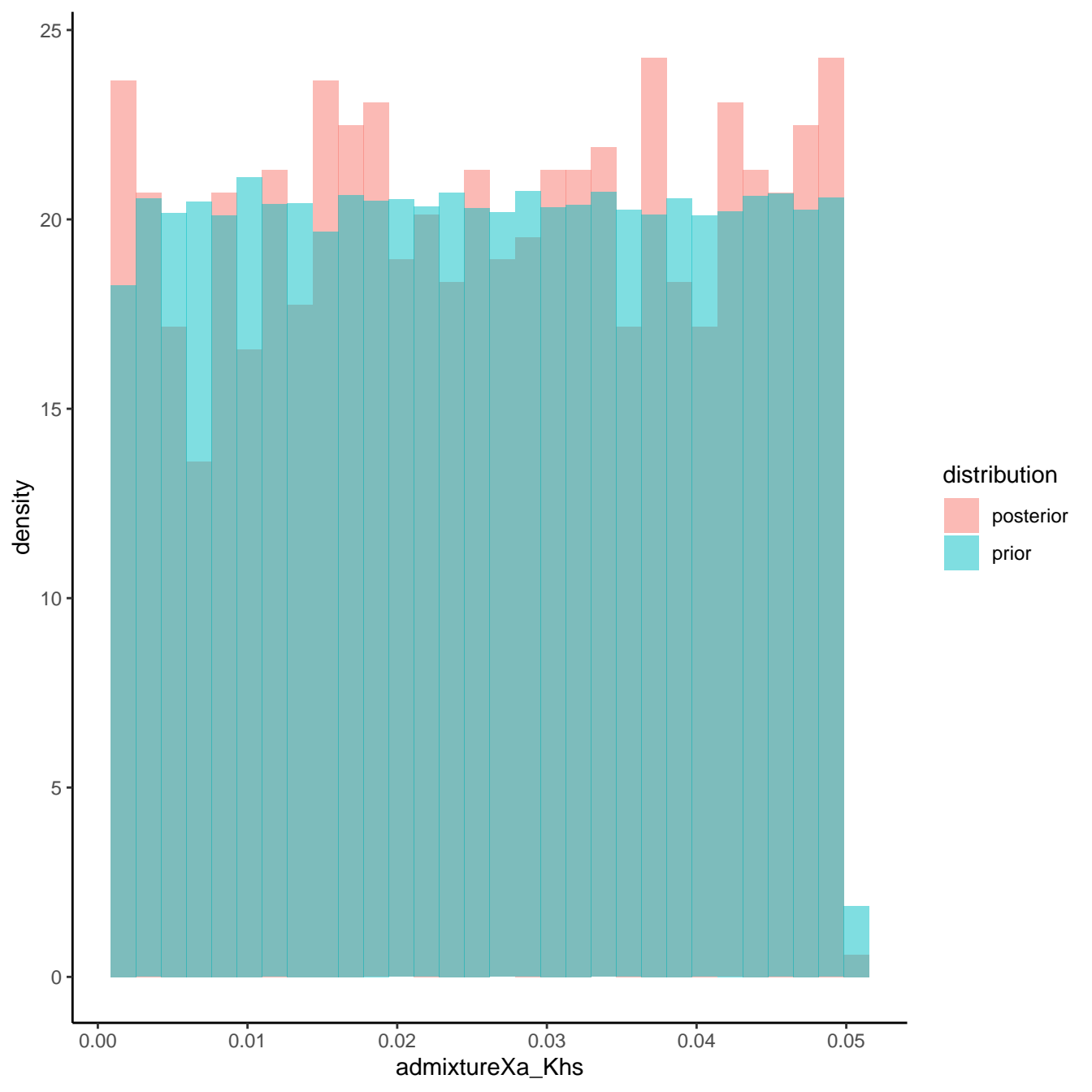

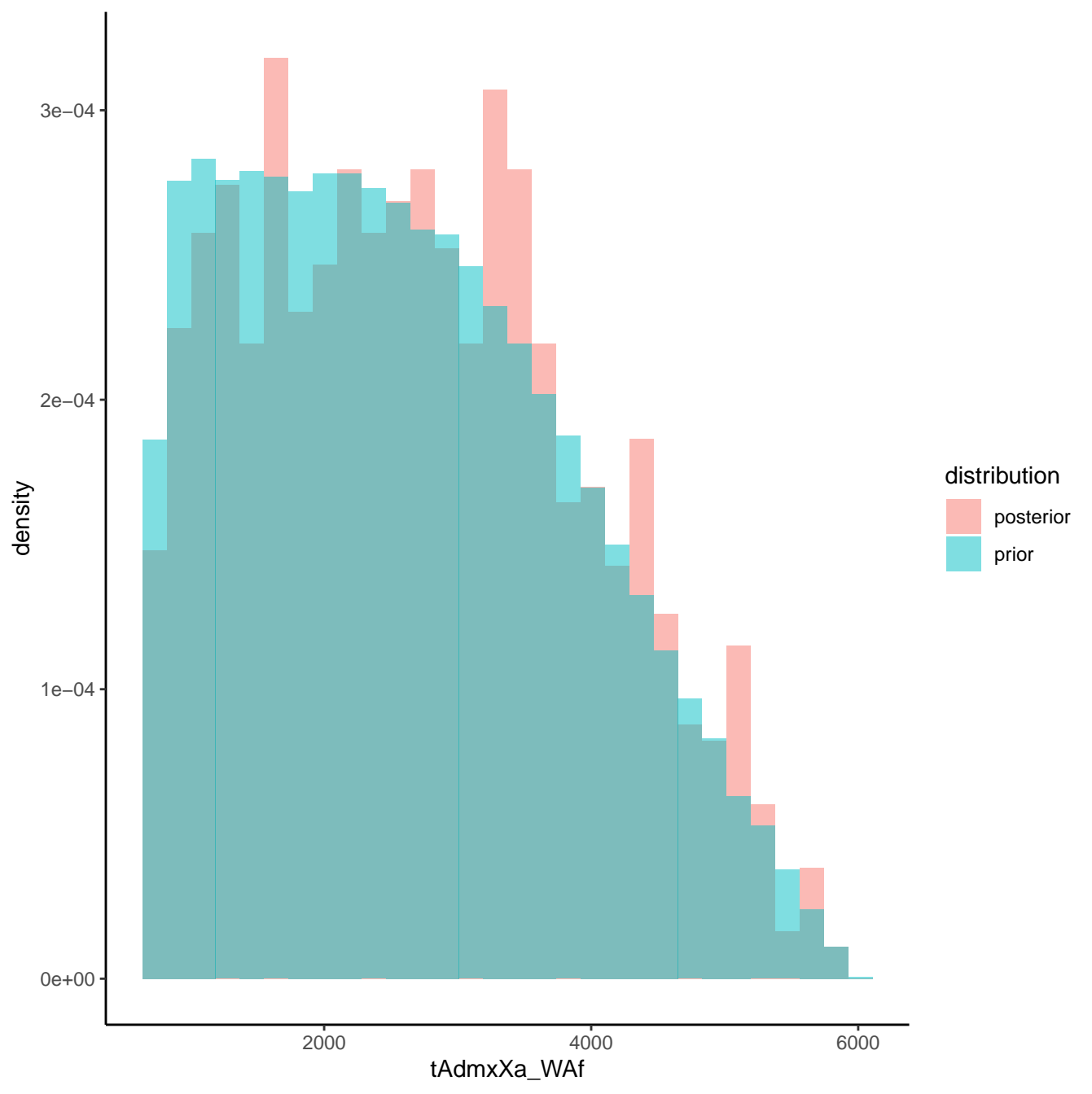

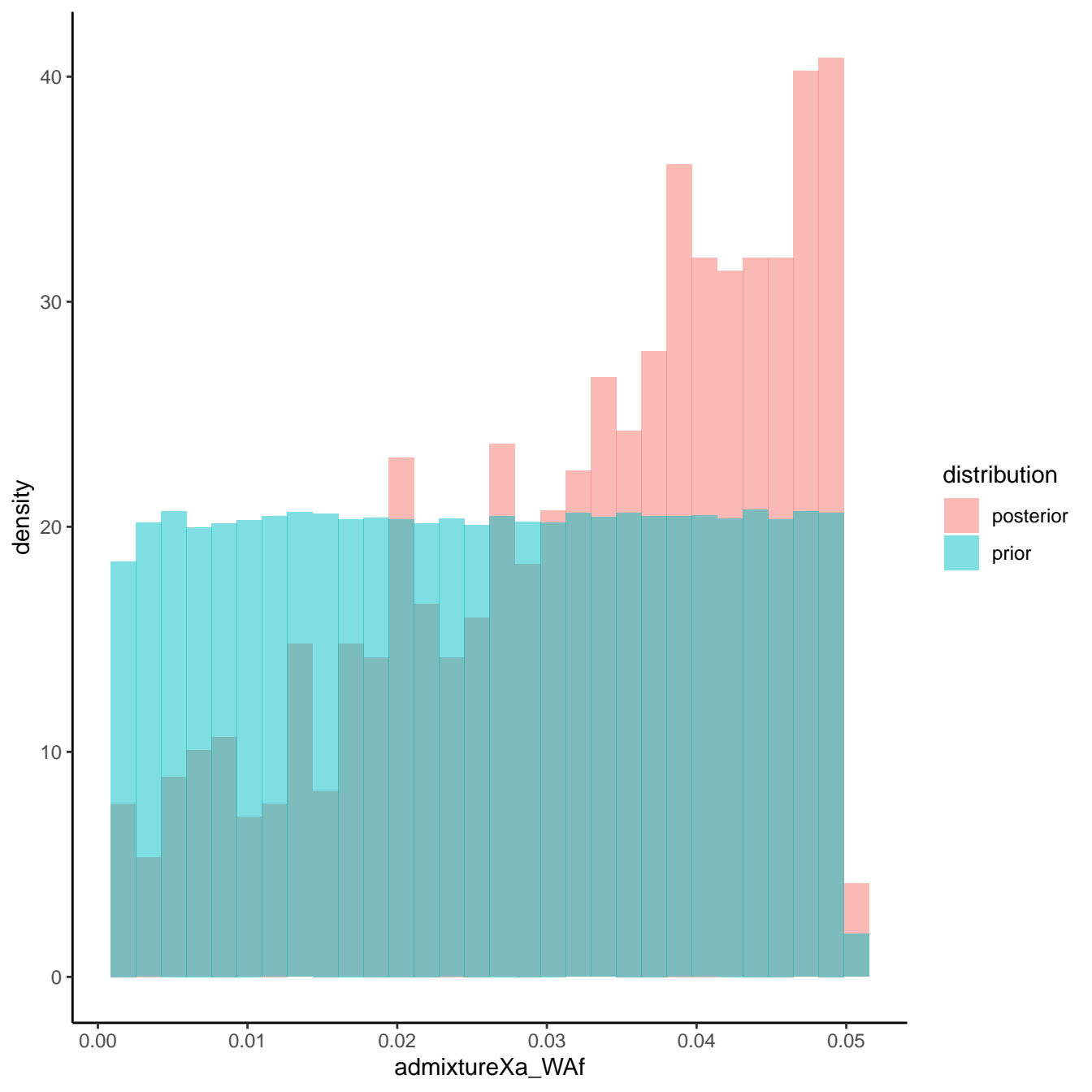

Supplement: Supplementary file 3 — Additional file 3. Histograms with the posterior versus prior distributions of all parameters for the best model in ABC DL analysis. [file 13059_2024_3341_MOESM3_ESM.pdf]
