# Supplementary material for: Modelling the demographic history of human North African genomes points to a recent soft split divergence between populations
Source: Genome Biol. 2024 Jul 30;25:201. doi: 10.1186/s13059-024-03341-4 (PMC11290046; doi:10.1186/s13059-024-03341-4)
Supplement: Supplementary file 4 — Additional file 4. Spearman correlation plots for all parameters in the best model in the ABC-DL analysis. [file 13059_2024_3341_MOESM4_ESM.pdf]

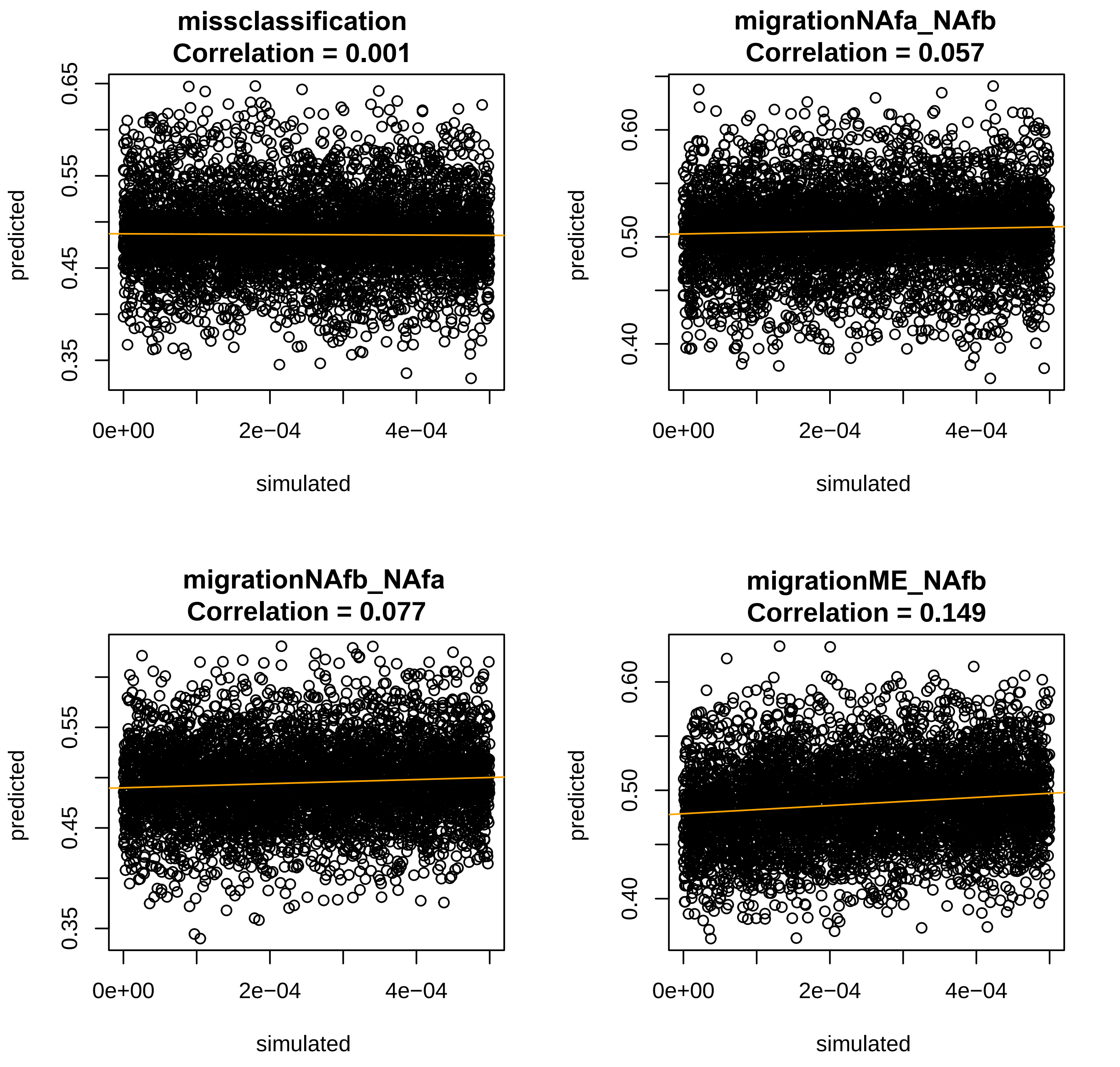
missclassification
Correlation = 0.001
migrationNAfa_NAfb
Correlation = 0.057
migrationNAfb_NAfa
Correlation = 0.077
migrationME_NAfb
Correlation = 0.149
predicted
simulated
0e+00
2e−04
4e−04
0.35
0.45
0.55
0.65
0.40
0.50
0.60

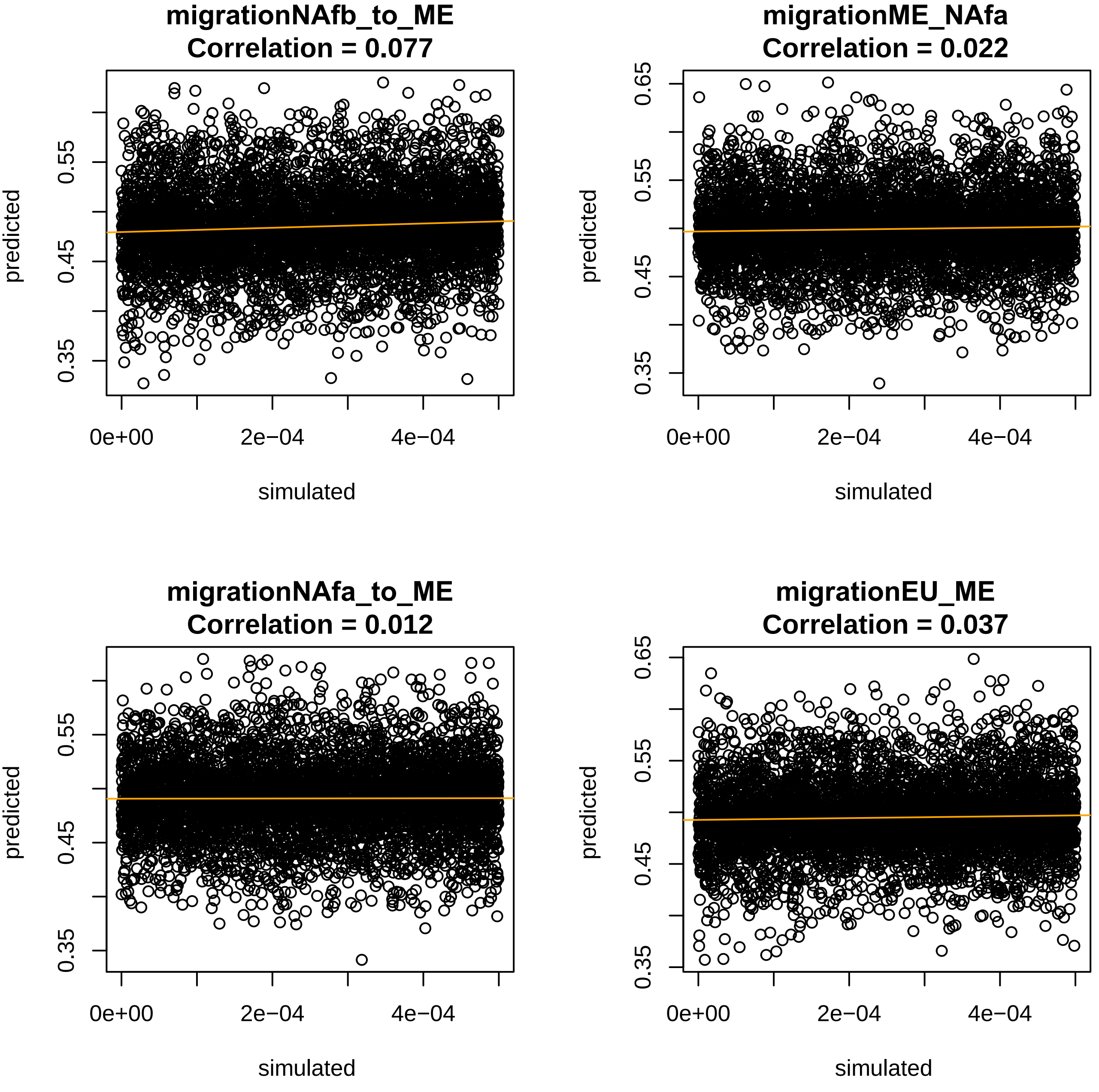
migrationNAfb_to_ME
Correlation = 0.077
migrationME_NAfa
Correlation = 0.022
migrationNAfa_to_ME
Correlation = 0.012
migrationEU_ME
Correlation = 0.037
predicted
simulated
0e+00
2e−04
4e−04
0.35
0.45
0.55
0.65

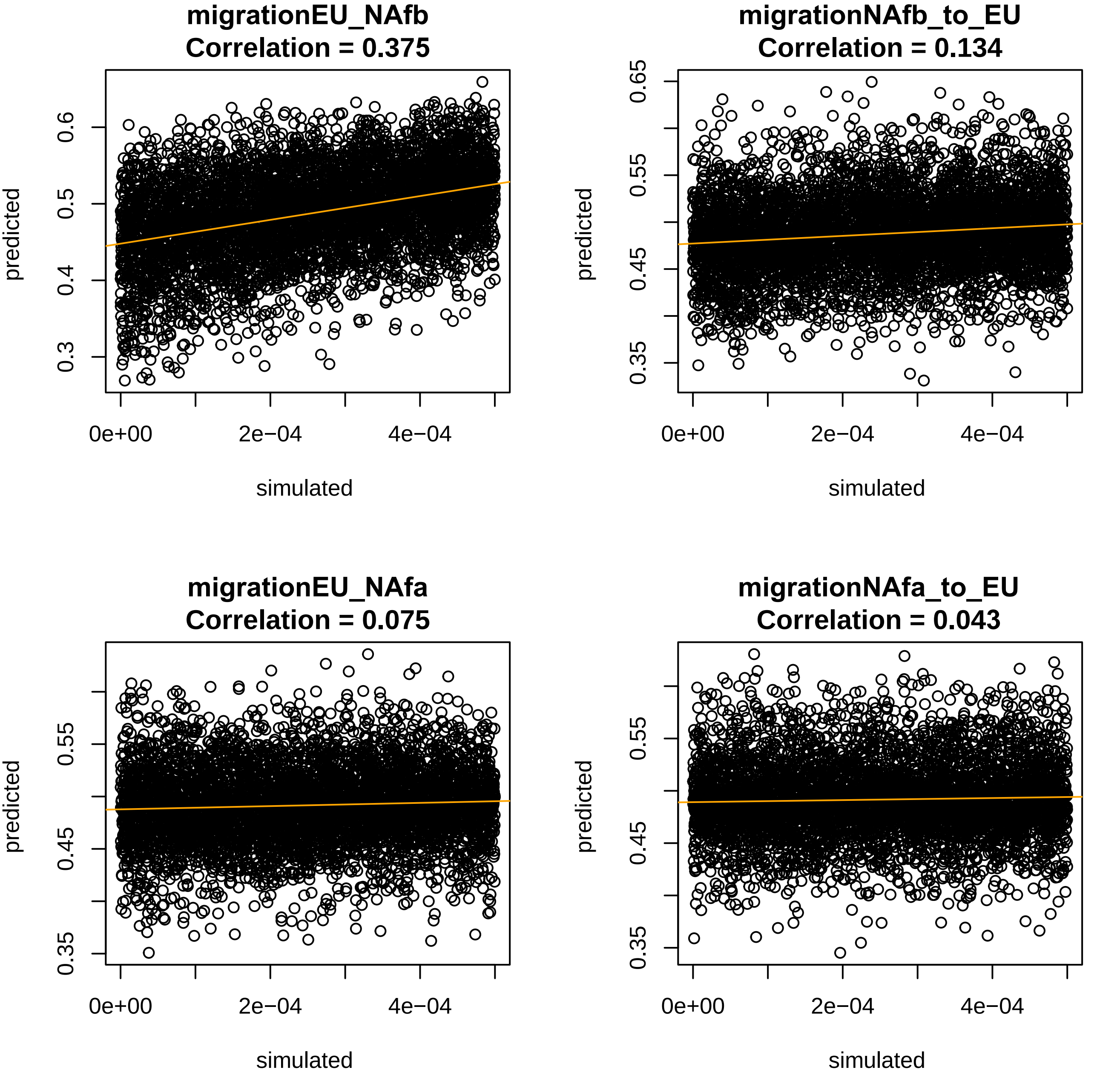
migrationEU_NAfb
Correlation = 0.375
predicted
simulated
0.3
0.4
0.5
0.6
0e+00
2e−04
4e−04
migrationNAfb_to_EU
Correlation = 0.134
0.35
0.45
0.55
0.65
migrationEU_NAfa
Correlation = 0.075
0.35
0.45
0.55
migrationNAfa_to_EU
Correlation = 0.043

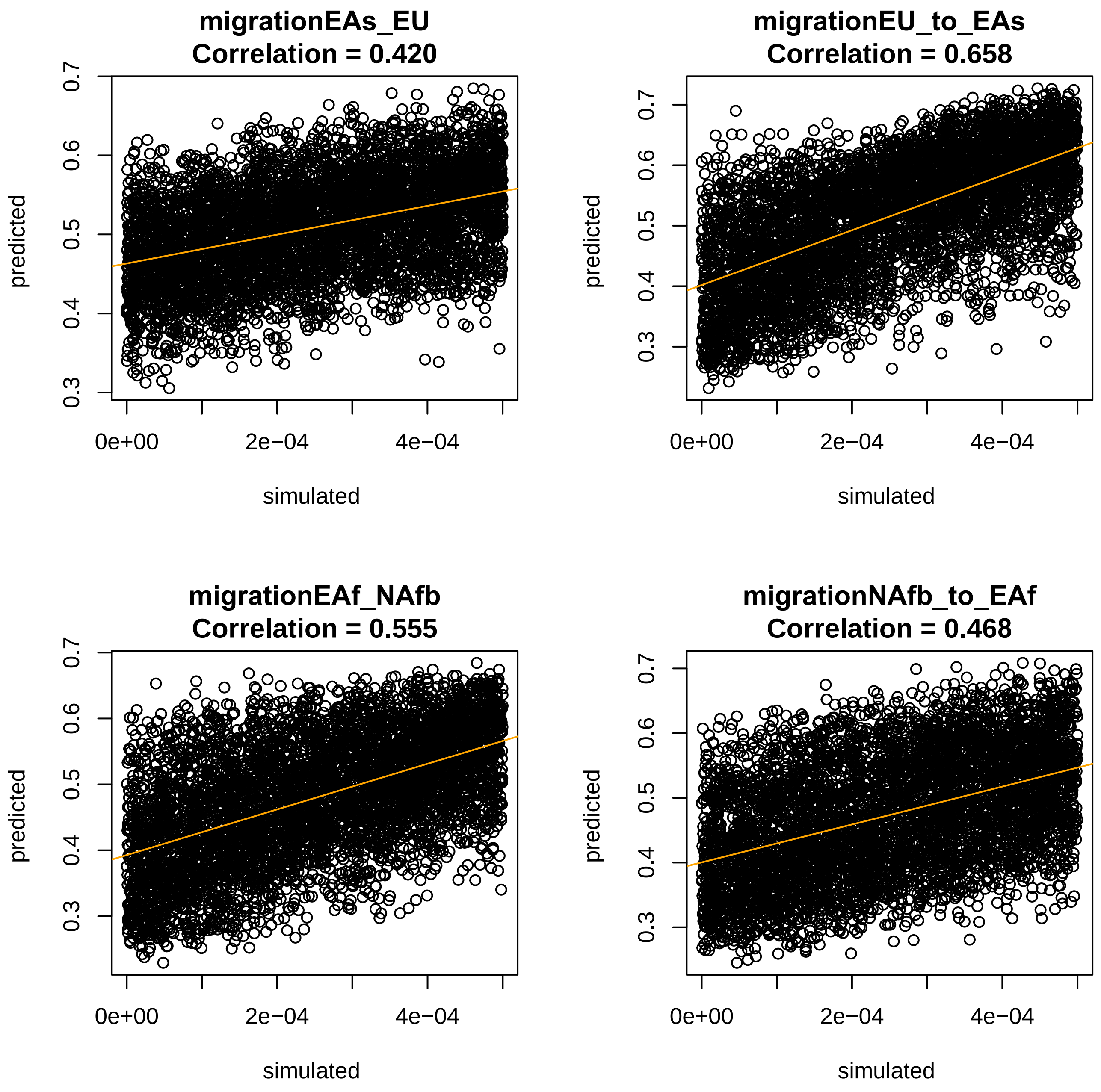
migrationEAs_EU
Correlation = 0.420
migrationEU_to_EAs
Correlation = 0.658
migrationEAf_NAfb
Correlation = 0.555
migrationNAfb_to_EAf
Correlation = 0.468
predicted
simulated
0.3
0.4
0.5
0.6
0.7
0e+00
2e−04
4e−04

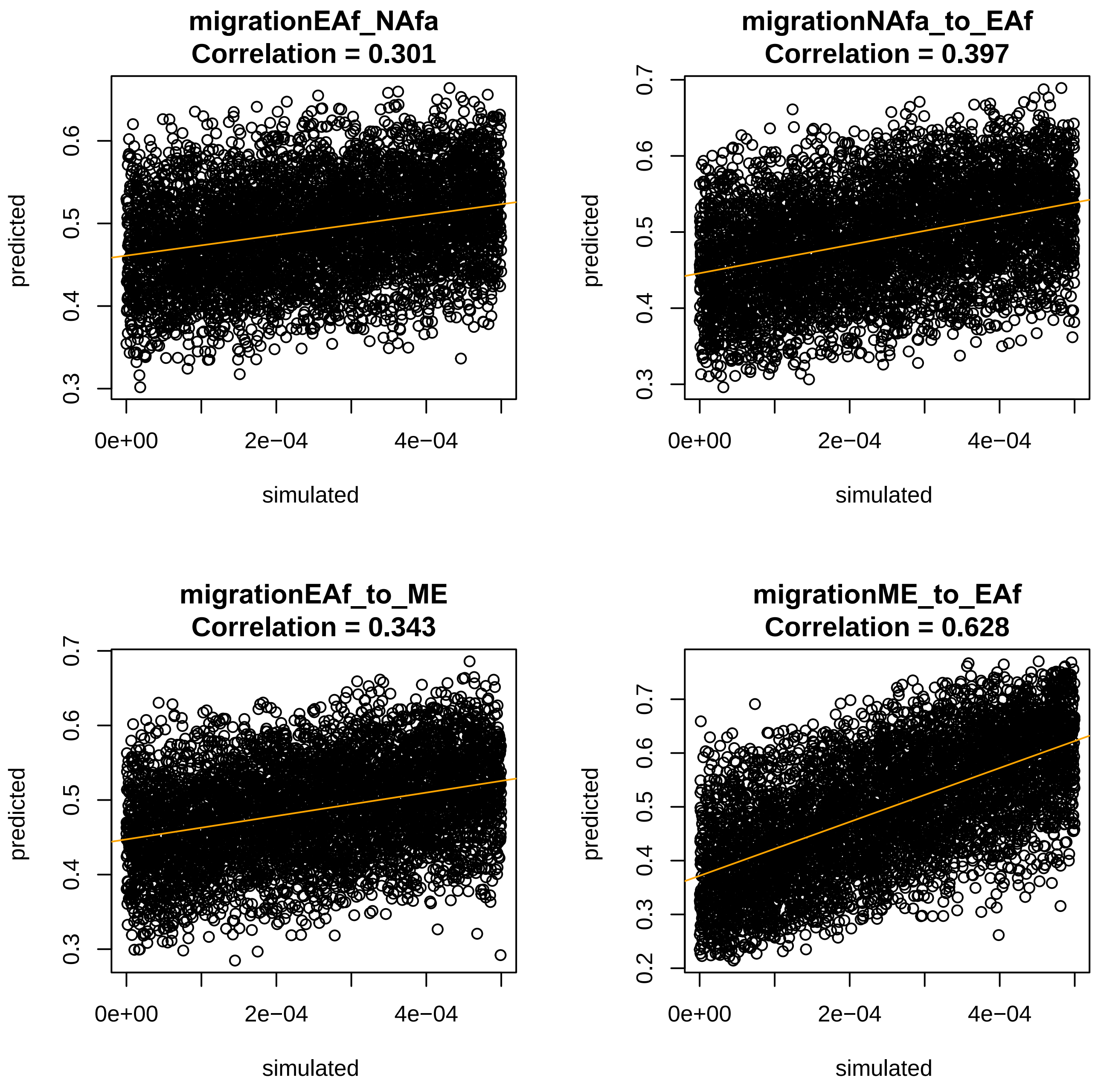
migrationEAf_NAfa
Correlation = 0.301
migrationNAfa_to_EAf
Correlation = 0.397
migrationEAf_to_ME
Correlation = 0.343
migrationME_to_EAf
Correlation = 0.628
predicted
simulated
0e+00
2e−04
4e−04
0.2
0.3
0.4
0.5
0.6
0.7

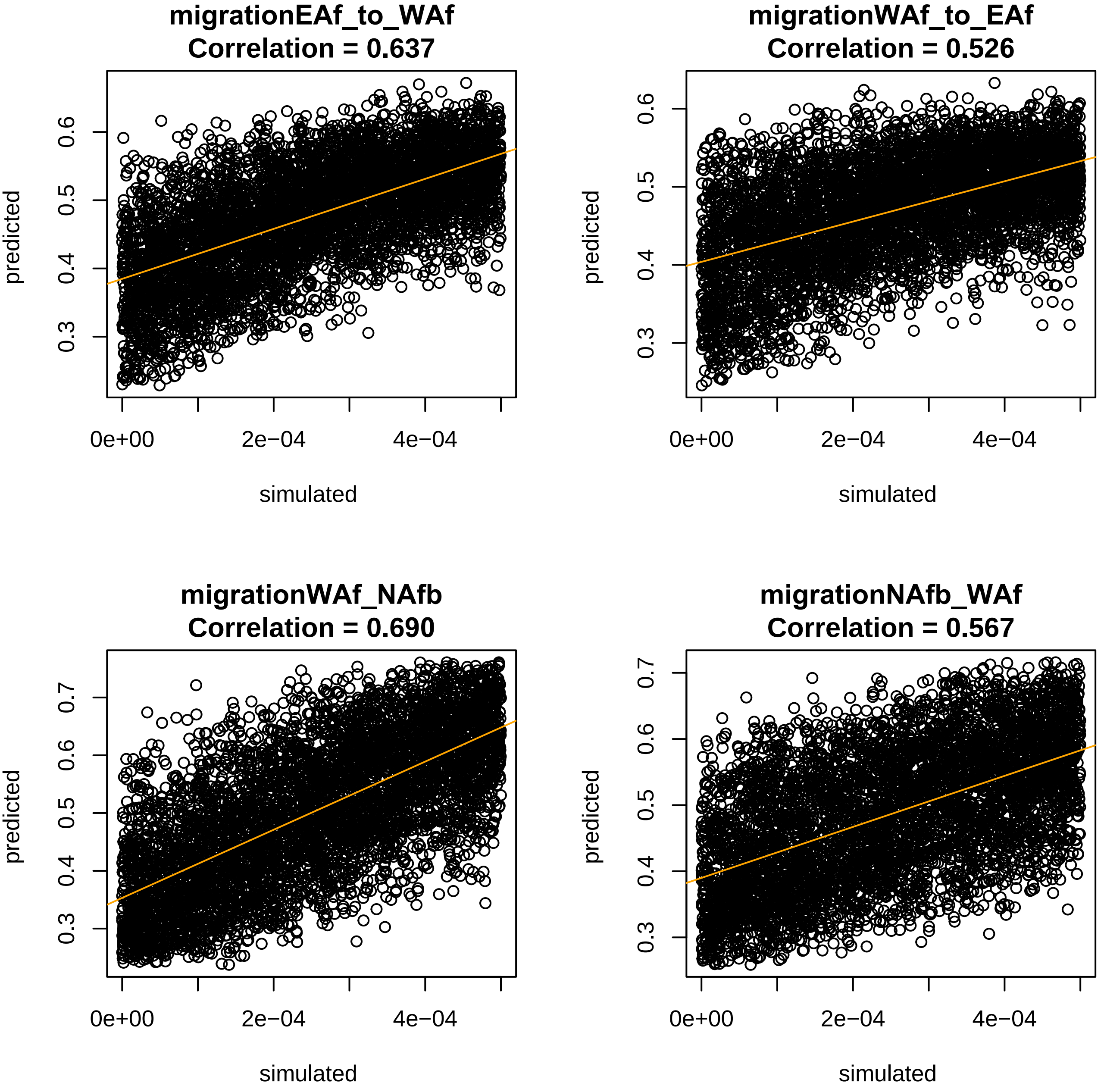
migrationEAf_to_WAf
Correlation = 0.637
predicted
simulated
0.3
0.4
0.5
0.6
0e+00
2e−04
4e−04
migrationWAf_to_EAf
Correlation = 0.526
predicted
simulated
0.3
0.4
0.5
0.6
0e+00
2e−04
4e−04
migrationWAf_NAfb
Correlation = 0.690
predicted
simulated
0.3
0.4
0.5
0.6
0.7
0e+00
2e−04
4e−04
migrationNAfb_WAf
Correlation = 0.567
predicted
simulated
0.3
0.4
0.5
0.6
0.7
0e+00
2e−04
4e−04

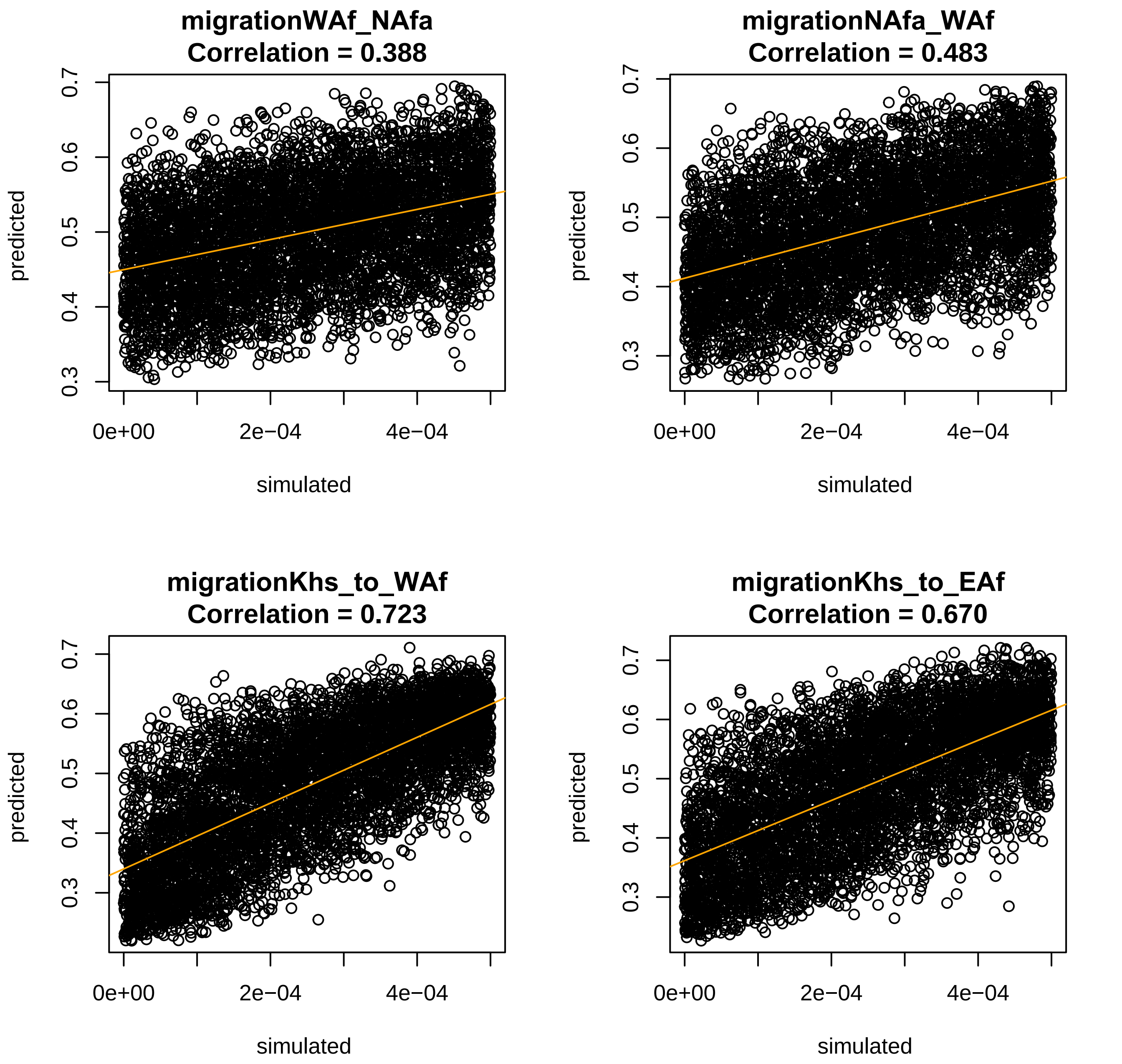
migrationWAf_NAfa
Correlation = 0.388
migrationNAfa_WAf
Correlation = 0.483
migrationKhs_to_WAf
Correlation = 0.723
migrationKhs_to_EAf
Correlation = 0.670
predicted
simulated
0e+00
2e−04
4e−04
0.3
0.4
0.5
0.6
0.7

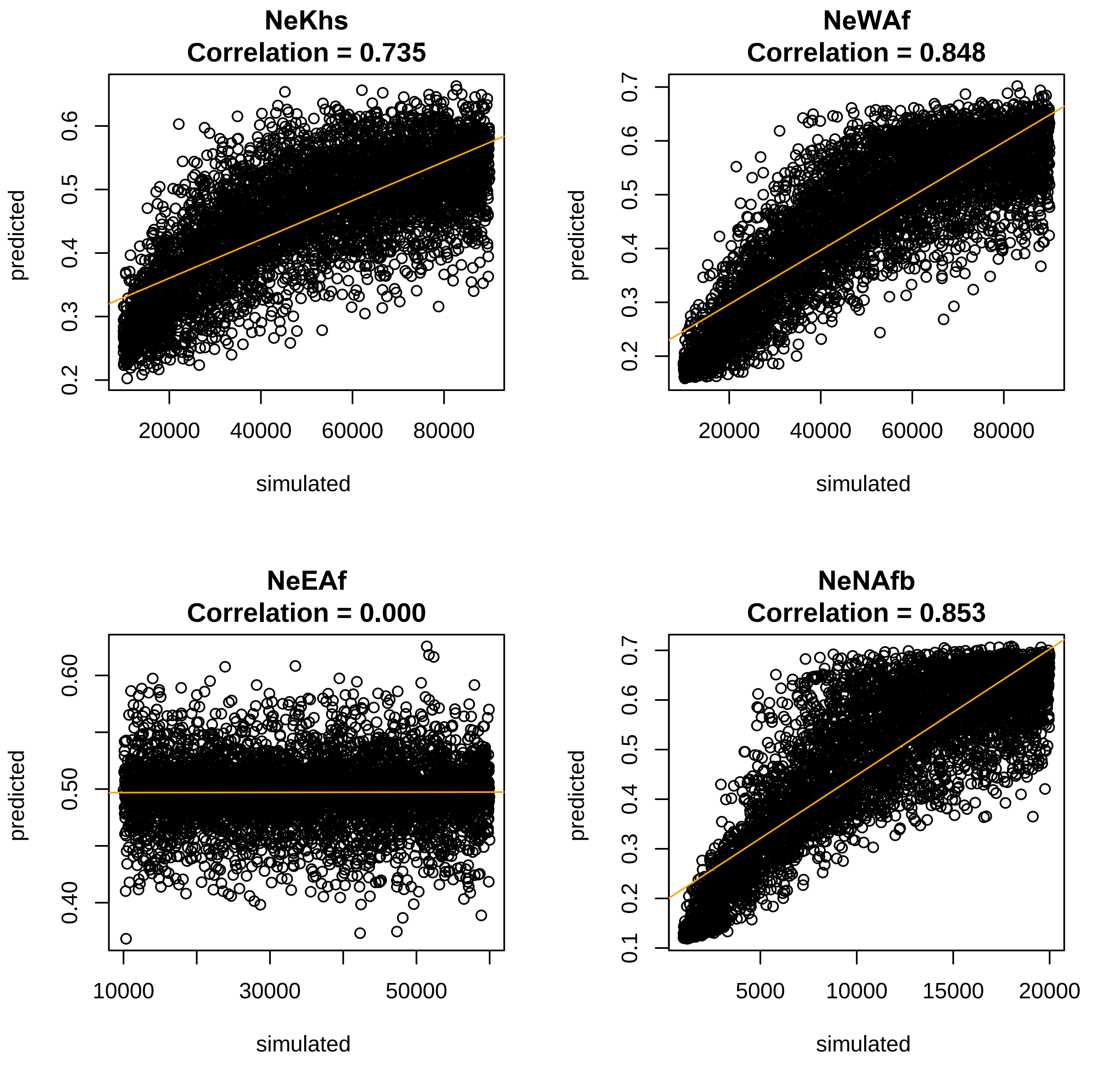
NeKhs
Correlation = 0.735
NeWAf
Correlation = 0.848
NeEAf
Correlation = 0.000
NeNAfb
Correlation = 0.853
predicted
simulated
0.2
0.3
0.4
0.5
0.6
0.7
0.1
0.40
0.50
0.60
20000
40000
60000
80000
10000
30000
50000
5000
15000

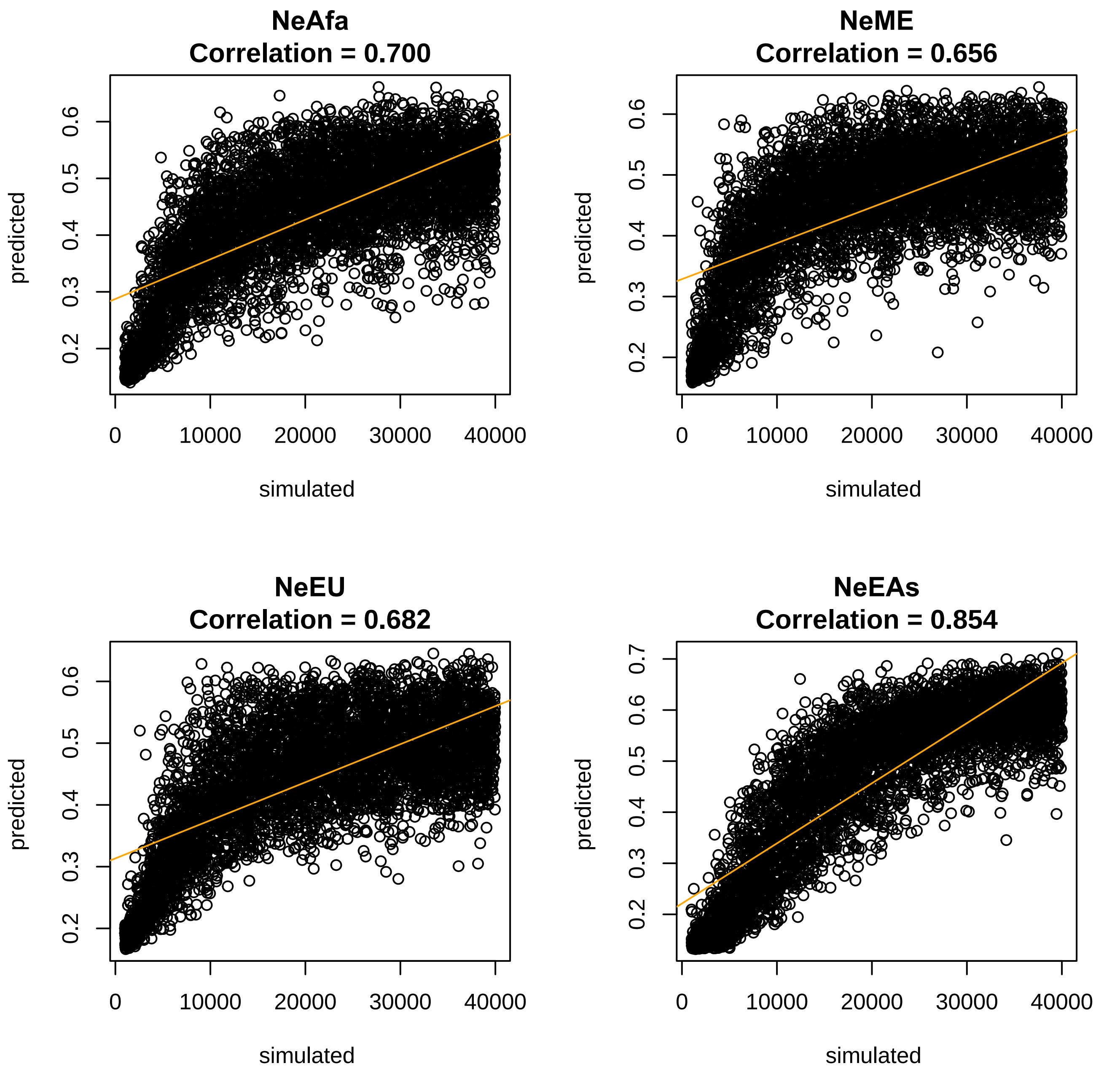
NeAfa
Correlation = 0.700
NeME
Correlation = 0.656
NeEU
Correlation = 0.682
NeEAs
Correlation = 0.854
predicted
simulated
0
10000
20000
30000
40000
0.2
0.3
0.4
0.5
0.6
0.7

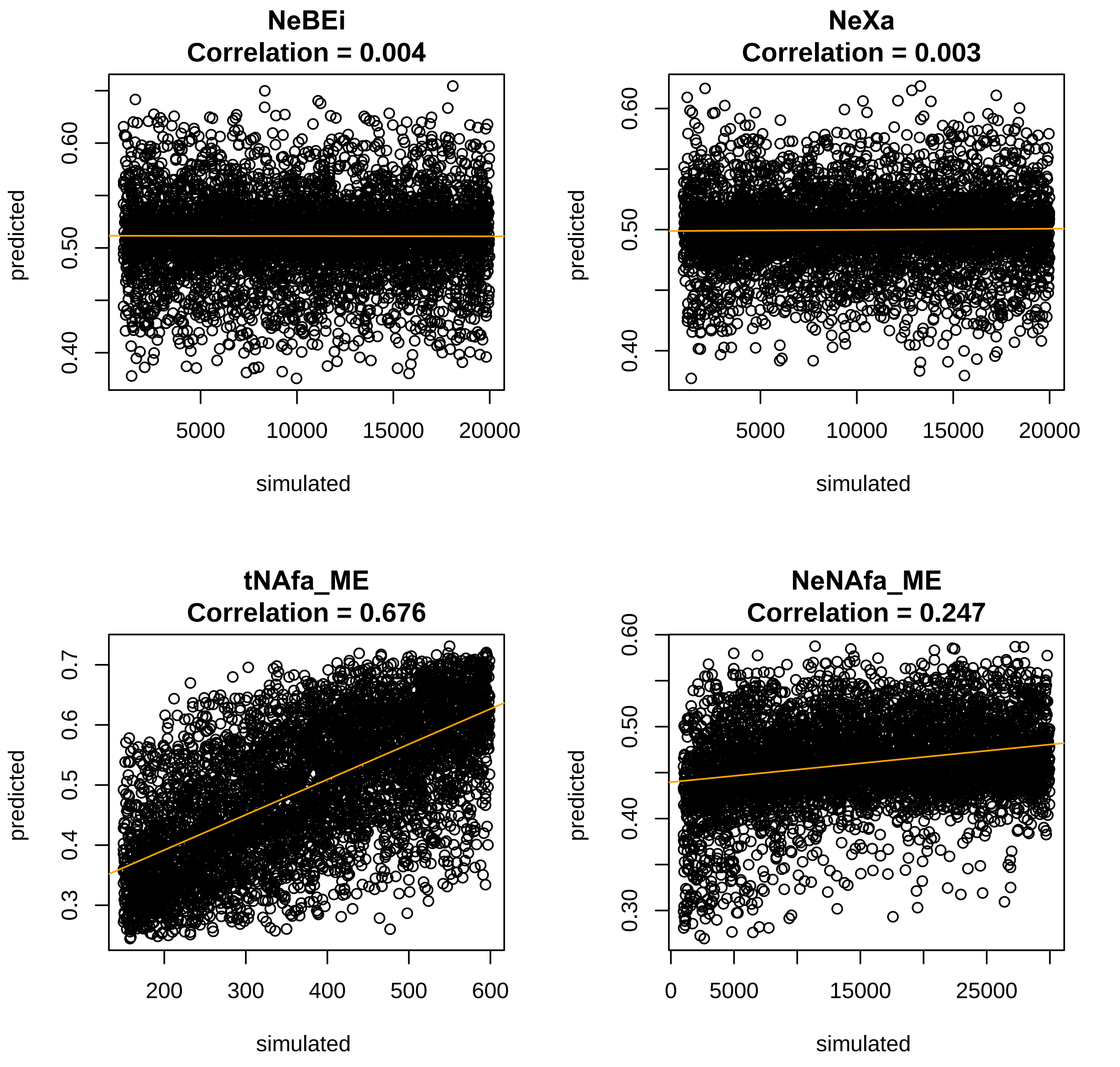
NeBEi
Correlation = 0.004
NeXa
Correlation = 0.003
tNAfa_ME
Correlation = 0.676
NeNAfa_ME
Correlation = 0.247
predicted
simulated
0.40
0.50
0.60
0.3
0.4
0.5
0.6
0.7
0.30
5000
10000
15000
20000
25000
0
200
300
400
500
600

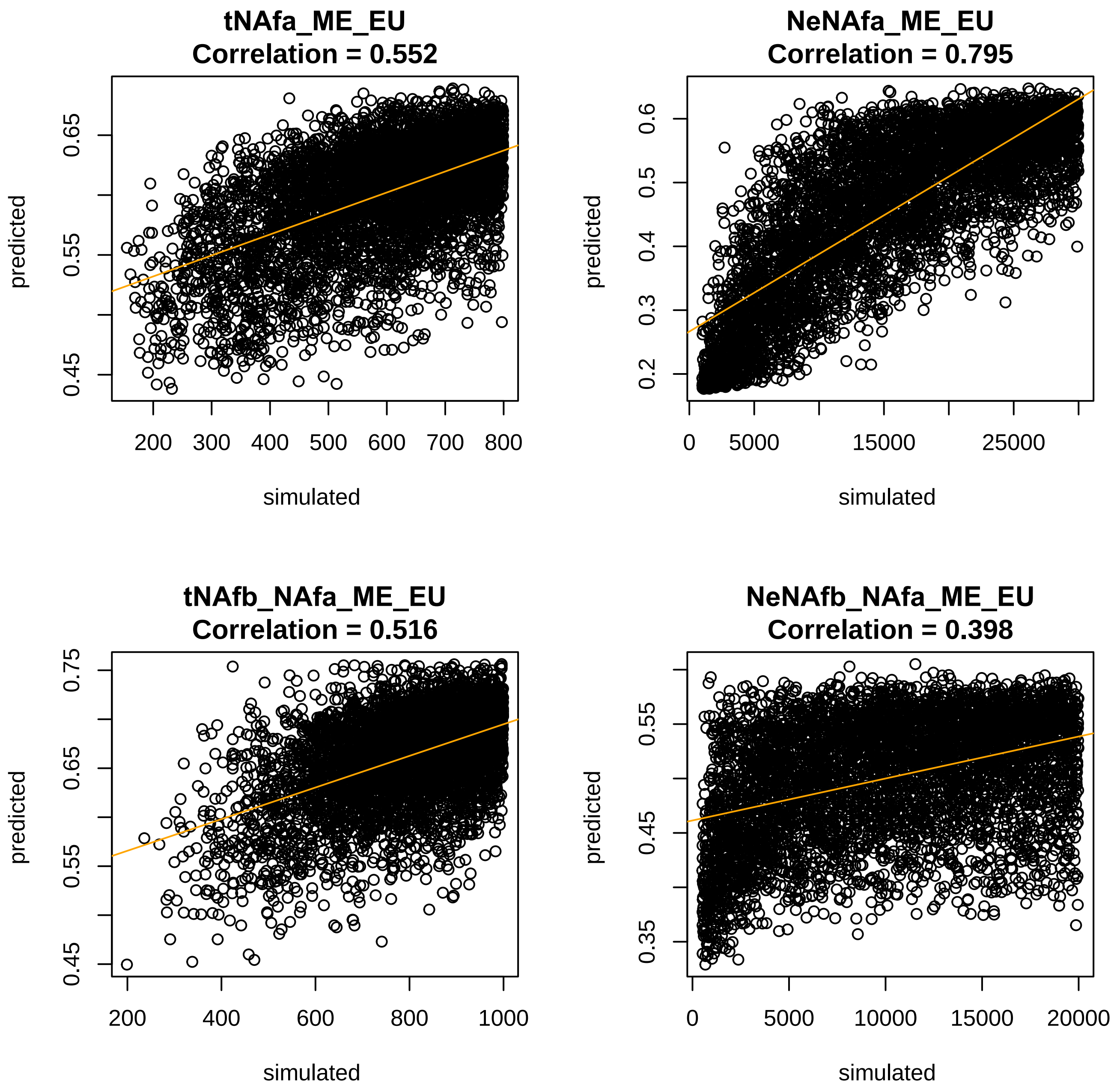
tNAfa_ME_EU
Correlation = 0.552
NeNAfa_ME_EU
Correlation = 0.795
tNAfb_NAfa_ME_EU
Correlation = 0.516
NeNAfb_NAfa_ME_EU
Correlation = 0.398
predicted
simulated
0.45
0.55
0.65
0.75
0.2
0.3
0.4
0.5
0.6
0.35
200
300
400
500
600
700
800
1000
0
5000
10000
15000
20000
25000

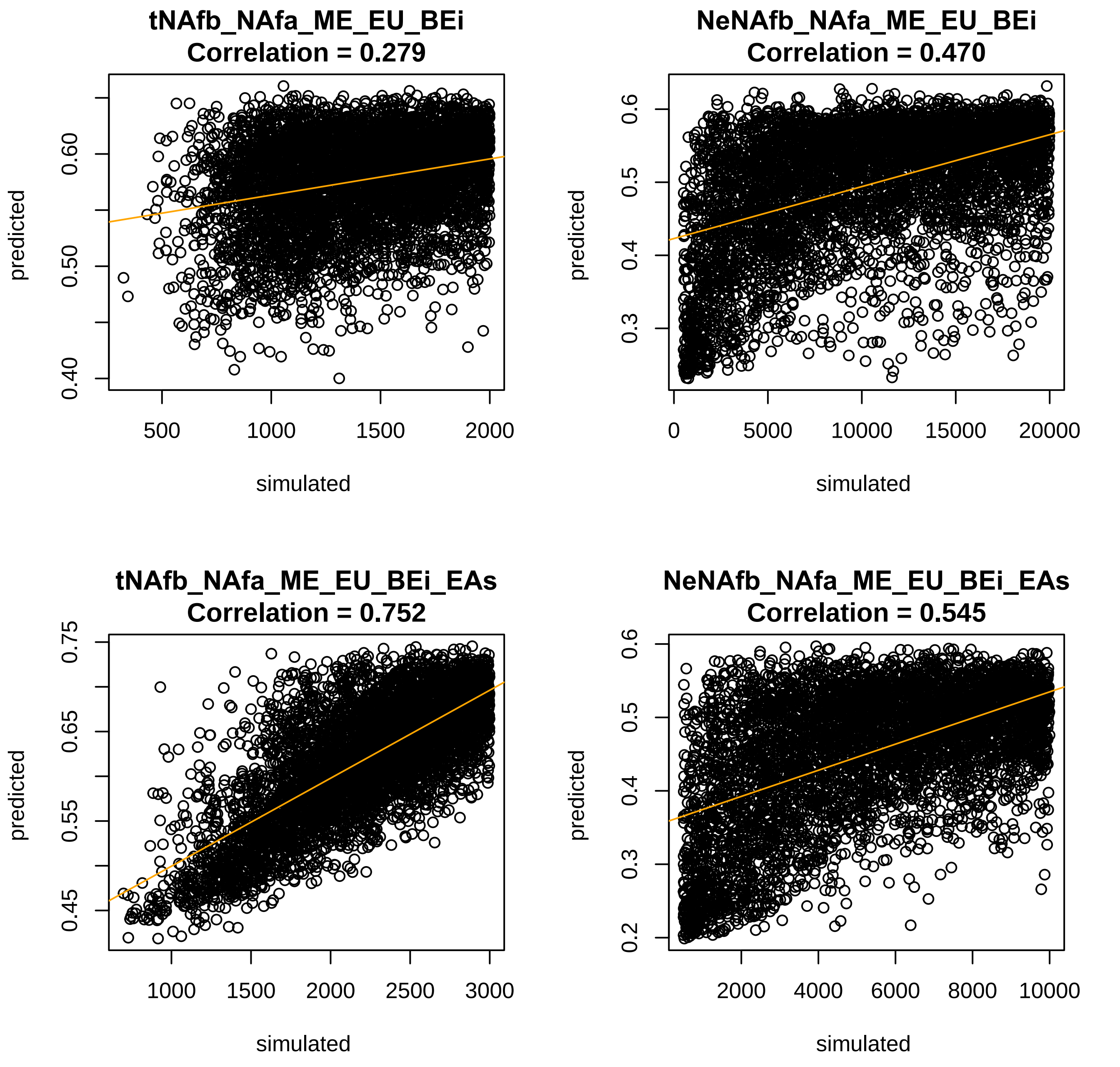
tNAfb_NAfa_ME_EU_BEi
Correlation = 0.279
predicted
simulated
0.40
0.50
0.60
500
1000
1500
2000
NeNAfb_NAfa_ME_EU_BEi
Correlation = 0.470
predicted
simulated
0.3
0.4
0.5
0.6
0
5000
10000
15000
20000
tNAfb_NAfa_ME_EU_BEi_EAs
Correlation = 0.752
predicted
simulated
0.45
0.55
0.65
0.75
1000
1500
2000
2500
3000
NeNAfb_NAfa_ME_EU_BEi_EAs
Correlation = 0.545
predicted
simulated
0.2
0.3
0.4
0.5
0.6
2000
4000
6000
8000
10000

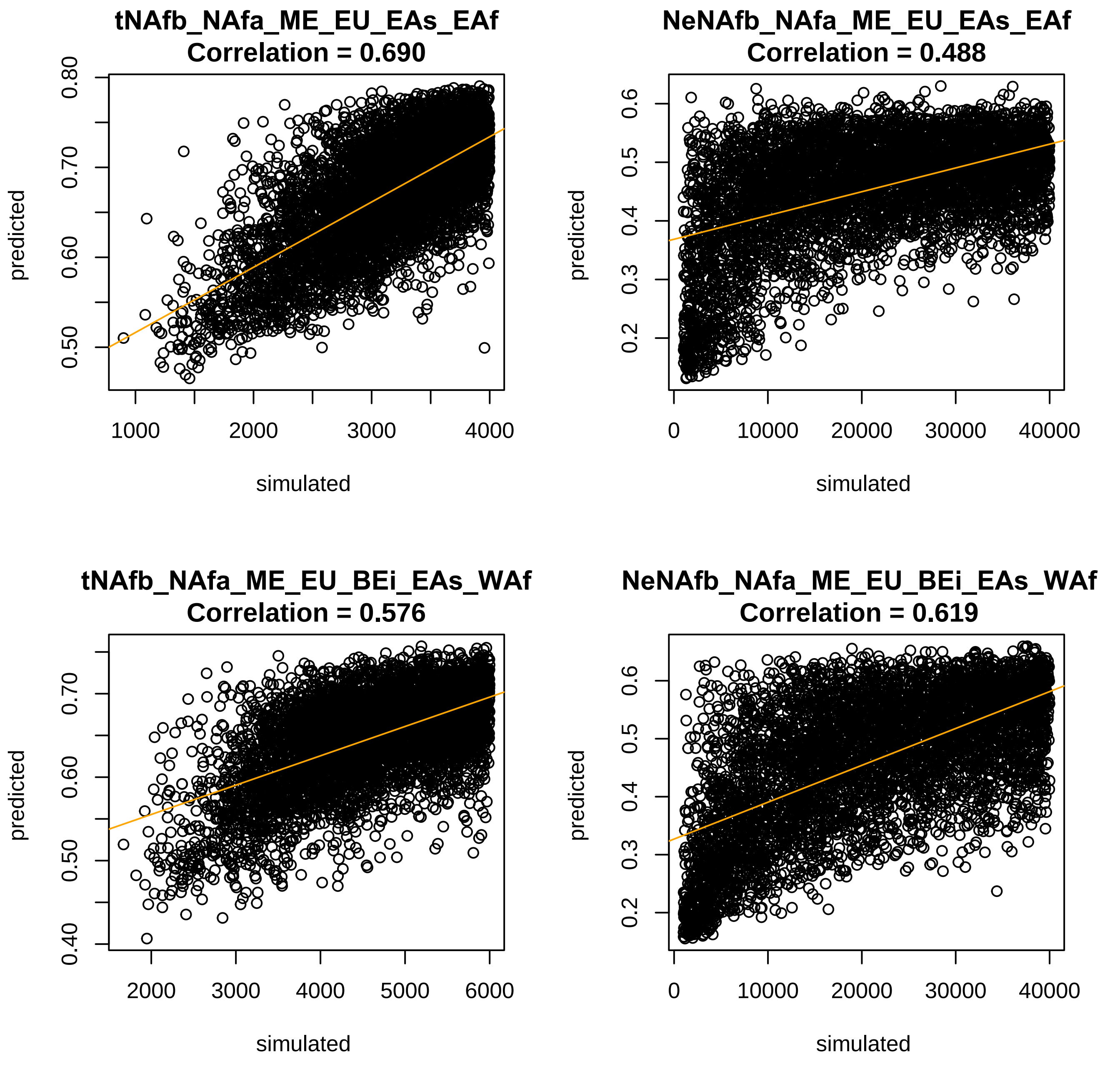

tNAfb_NAfa_ME_EU_EAs_EAf
Correlation = 0.690
predicted
simulated
0.50
0.60
0.70
0.80
1000
2000
3000
4000
NeNAfb_NAfa_ME_EU_EAs_EAf
Correlation = 0.488
predicted
simulated
0.2
0.3
0.4
0.5
0.6
0
10000
20000
30000
40000
tNAfb_NAfa_ME_EU_BEi_EAs_WAf
Correlation = 0.576
predicted
simulated
0.40
0.50
0.60
0.70
2000
3000
4000
5000
6000
NeNAfb_NAfa_ME_EU_BEi_EAs_WAf
Correlation = 0.619
predicted
simulated
0.2
0.3
0.4
0.5
0.6
0
10000
20000
30000
40000

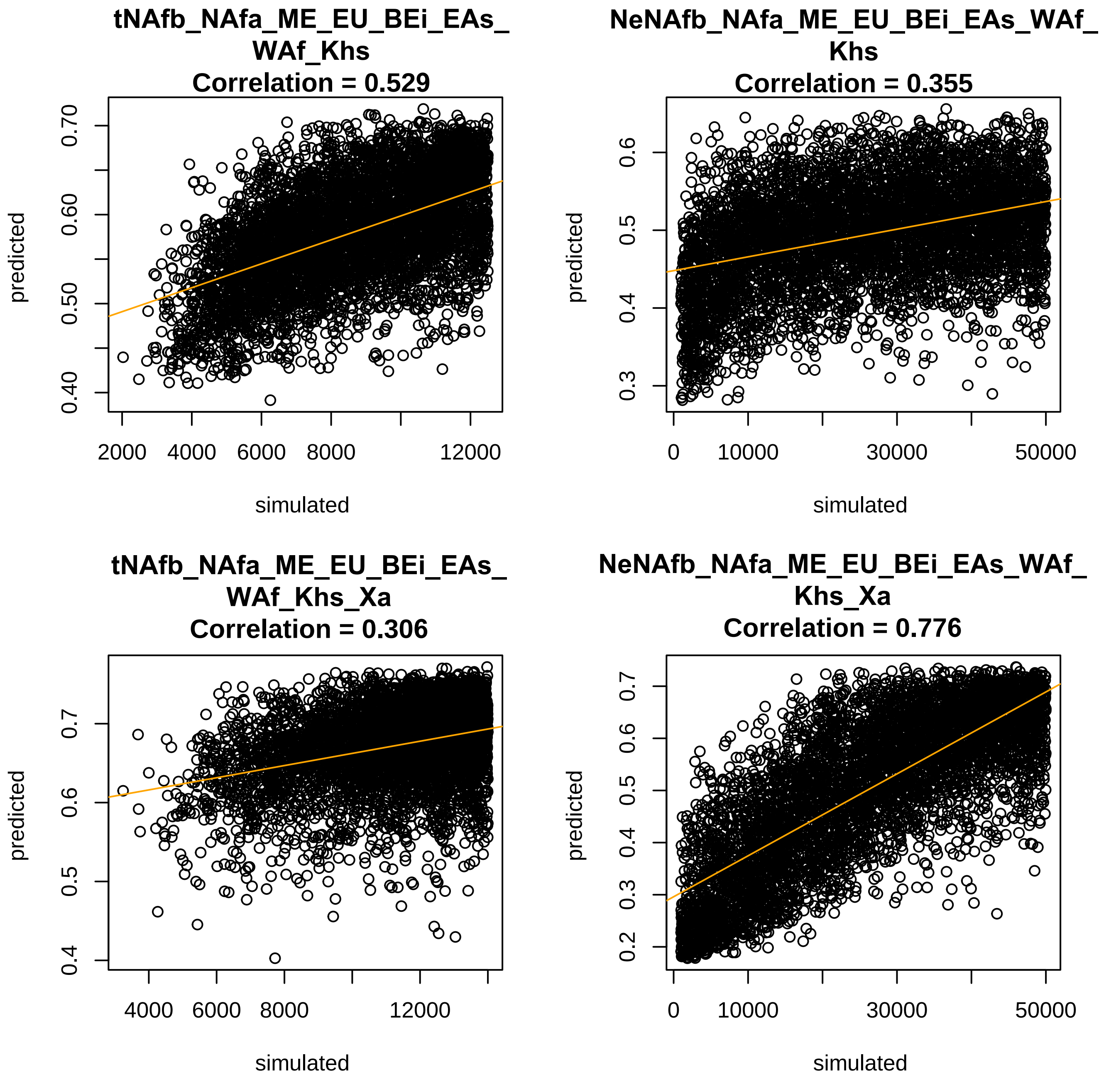
tNAfb_NAfa_ME_EU_BEi_EAs_
WAf_Khs
Correlation = 0.529
predicted
0.40
0.50
0.60
0.70
2000
4000
6000
8000
12000
simulated
NeNAfb_NAfa_ME_EU_BEi_EAs_WAf_
Khs
Correlation = 0.355
predicted
0.3
0.4
0.5
0.6
0
10000
30000
50000
simulated
tNAfb_NAfa_ME_EU_BEi_EAs_
WAf_Khs_Xa
Correlation = 0.306
predicted
0.4
0.5
0.6
0.7
4000
6000
8000
12000
simulated
NeNAfb_NAfa_ME_EU_BEi_EAs_WAf_
Khs_Xa
Correlation = 0.776
predicted
0.2
0.3
0.4
0.5
0.6
0.7
0
10000
30000
50000
simulated

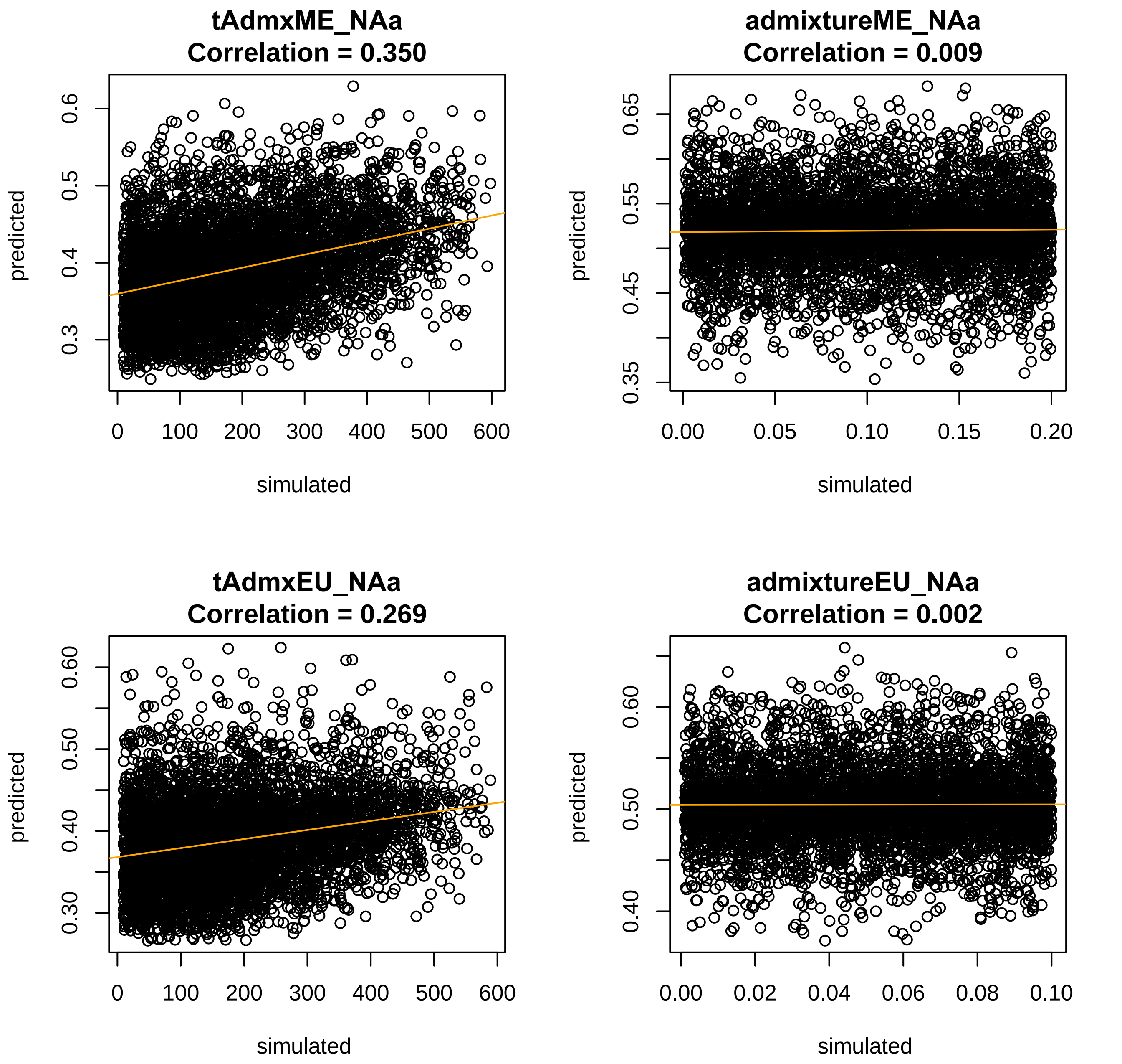
tAdmxME_NAa
Correlation = 0.350
admixtureME_NAa
Correlation = 0.009
tAdmxEU_NAa
Correlation = 0.269
admixtureEU_NAa
Correlation = 0.002
predicted
simulated
0.3
0.4
0.5
0.6
0
100
200
300
400
500
600
0.35
0.45
0.55
0.65
0.00
0.05
0.10
0.15
0.20
0.30
0.40
0.50
0.60
0.02
0.04
0.06
0.08

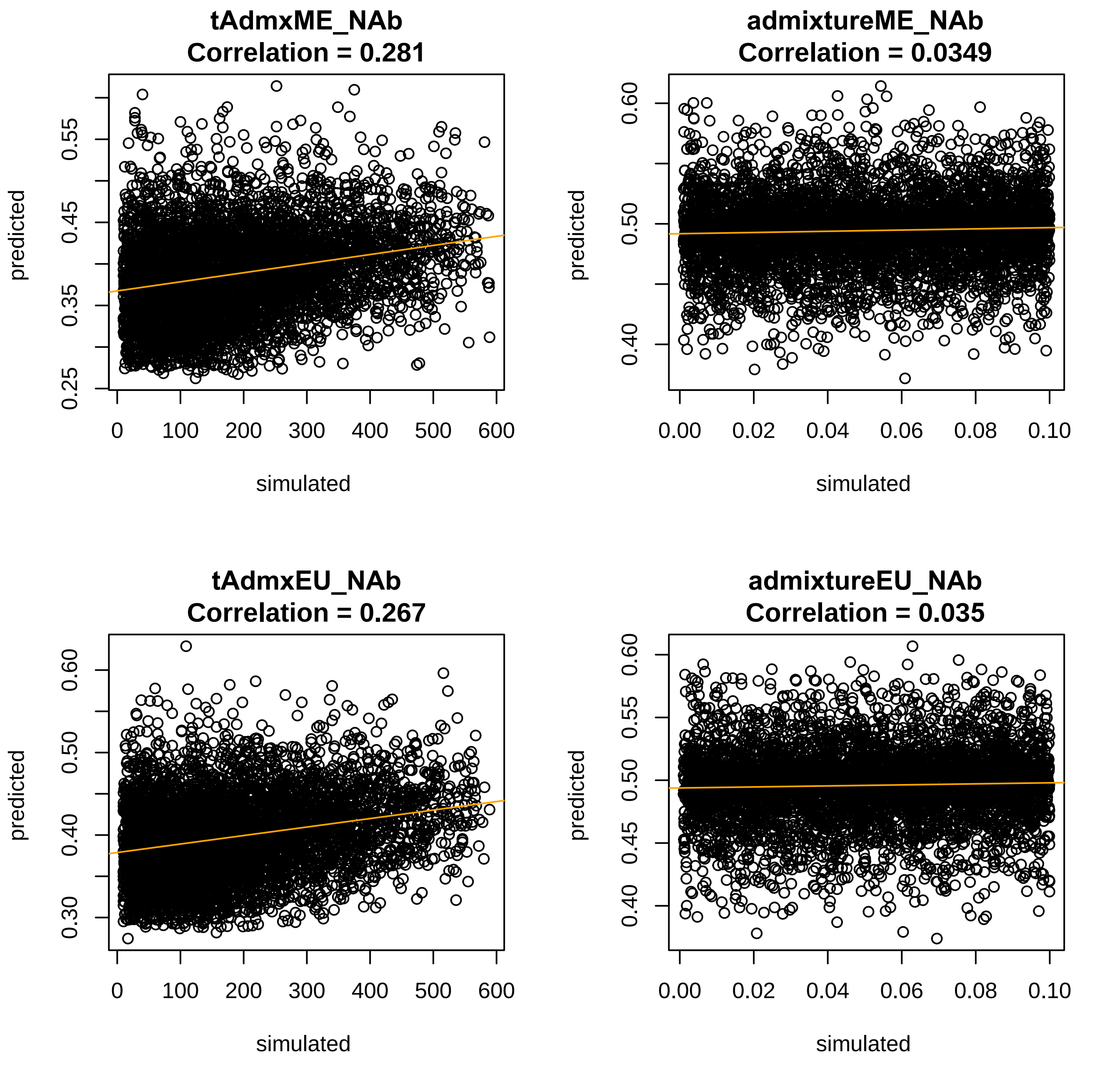
tAdmxME_NAb
Correlation = 0.281
admixtureME_NAb
Correlation = 0.0349
tAdmxEU_NAb
Correlation = 0.267
admixtureEU_NAb
Correlation = 0.035
predicted
simulated

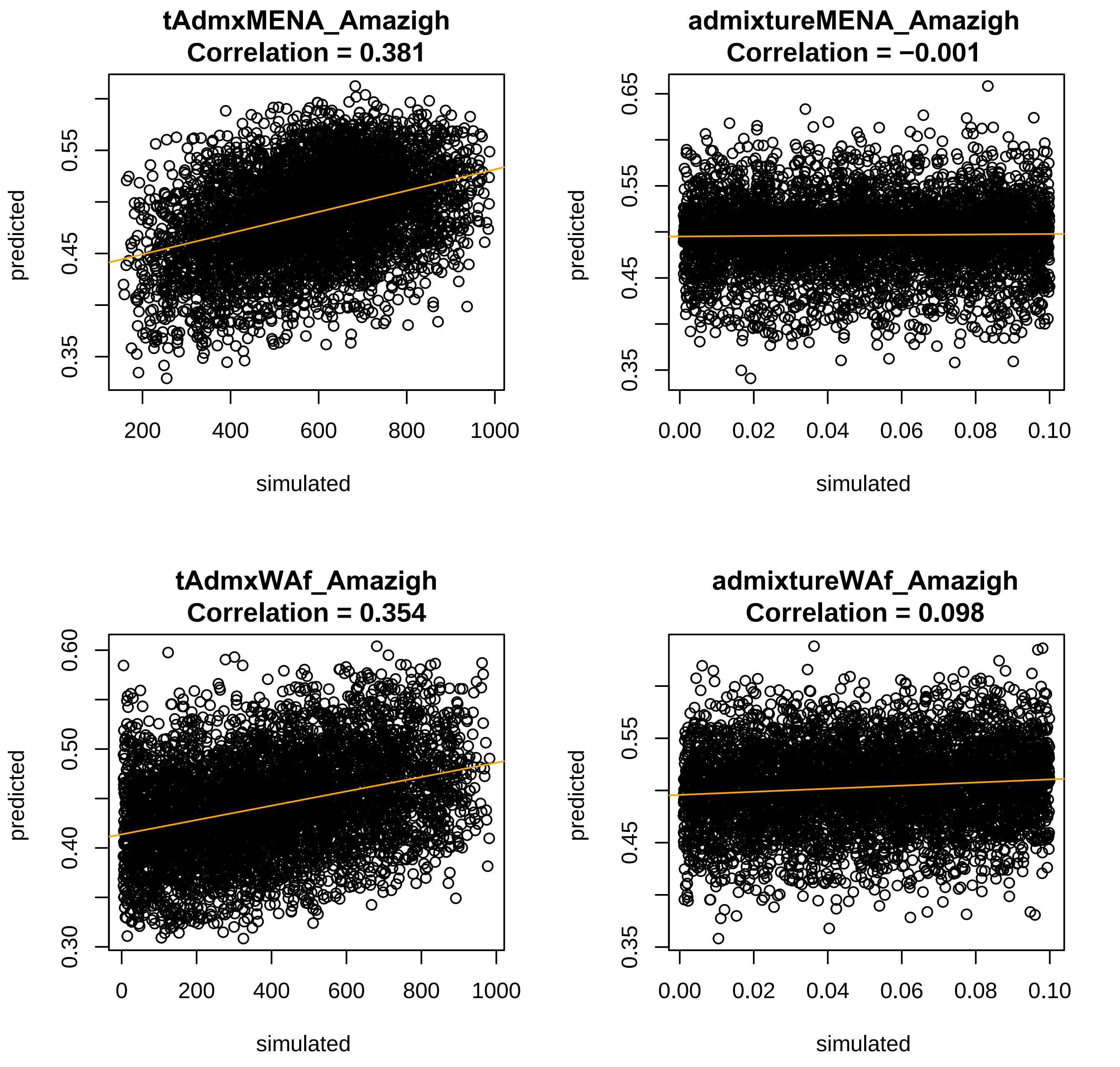
tAdmxMENA_Amazigh
Correlation = 0.381
admixtureMENA_Amazigh
Correlation = −0.001
tAdmxWAf_Amazigh
Correlation = 0.354
admixtureWAf_Amazigh
Correlation = 0.098
predicted
simulated
0.35 0.45 0.55
200 400 600 800 1000
0.35 0.45 0.55 0.65
0.00 0.02 0.04 0.06 0.08 0.10
0.30 0.40 0.50 0.60
0 200 400 600 800 1000

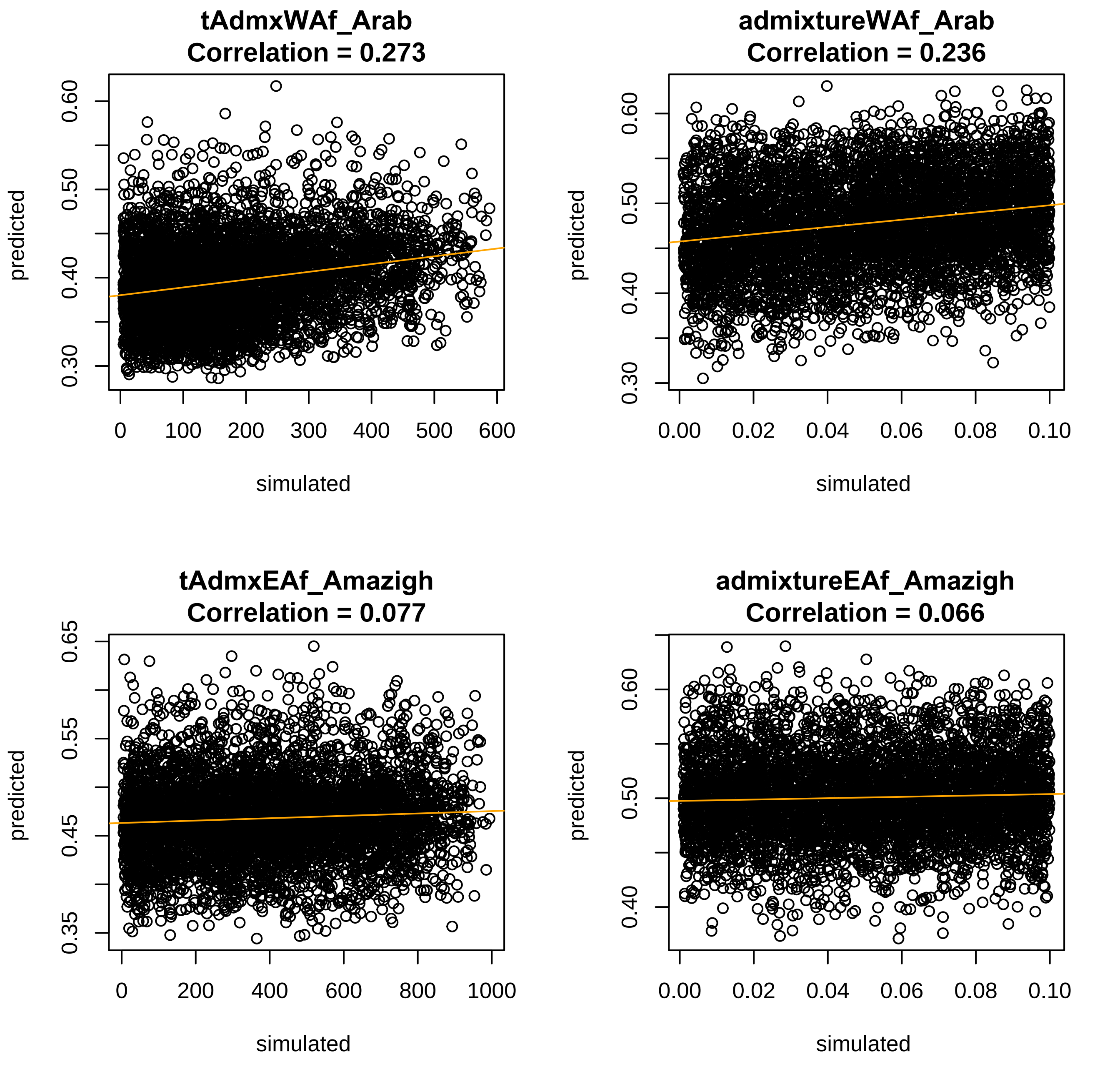
tAdmxWAf_Arab
Correlation = 0.273
admixtureWAf_Arab
Correlation = 0.236
tAdmxEAf_Amazigh
Correlation = 0.077
admixtureEAf_Amazigh
Correlation = 0.066
predicted
simulated
0.30
0.40
0.50
0.60
0.35
0.45
0.55
0.65
0
100
200
300
400
500
600
800
1000
0.00
0.02
0.04
0.06
0.08
0.10

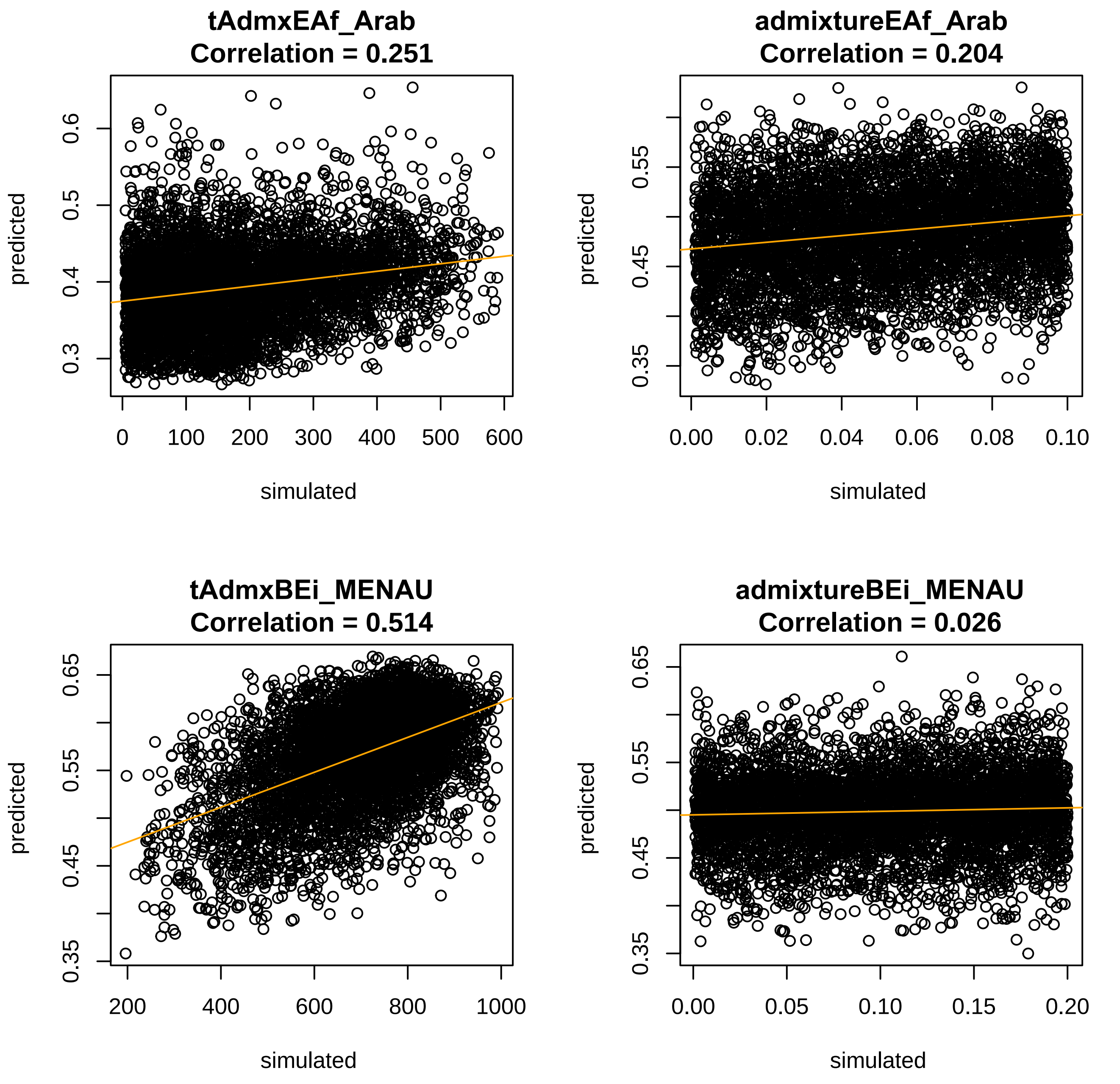
tAdmxEAf_Arab
Correlation = 0.251
predicted
simulated
0.3
0.4
0.5
0.6
0
100
200
300
400
500
600
admixtureEAf_Arab
Correlation = 0.204
predicted
simulated
0.35
0.45
0.55
0.00
0.02
0.04
0.06
0.08
0.10
tAdmxBEi_MENAU
Correlation = 0.514
predicted
simulated
0.35
0.45
0.55
0.65
200
400
600
800
1000
admixtureBEi_MENAU
Correlation = 0.026
predicted
simulated
0.35
0.45
0.55
0.65
0.00
0.05
0.10
0.15
0.20

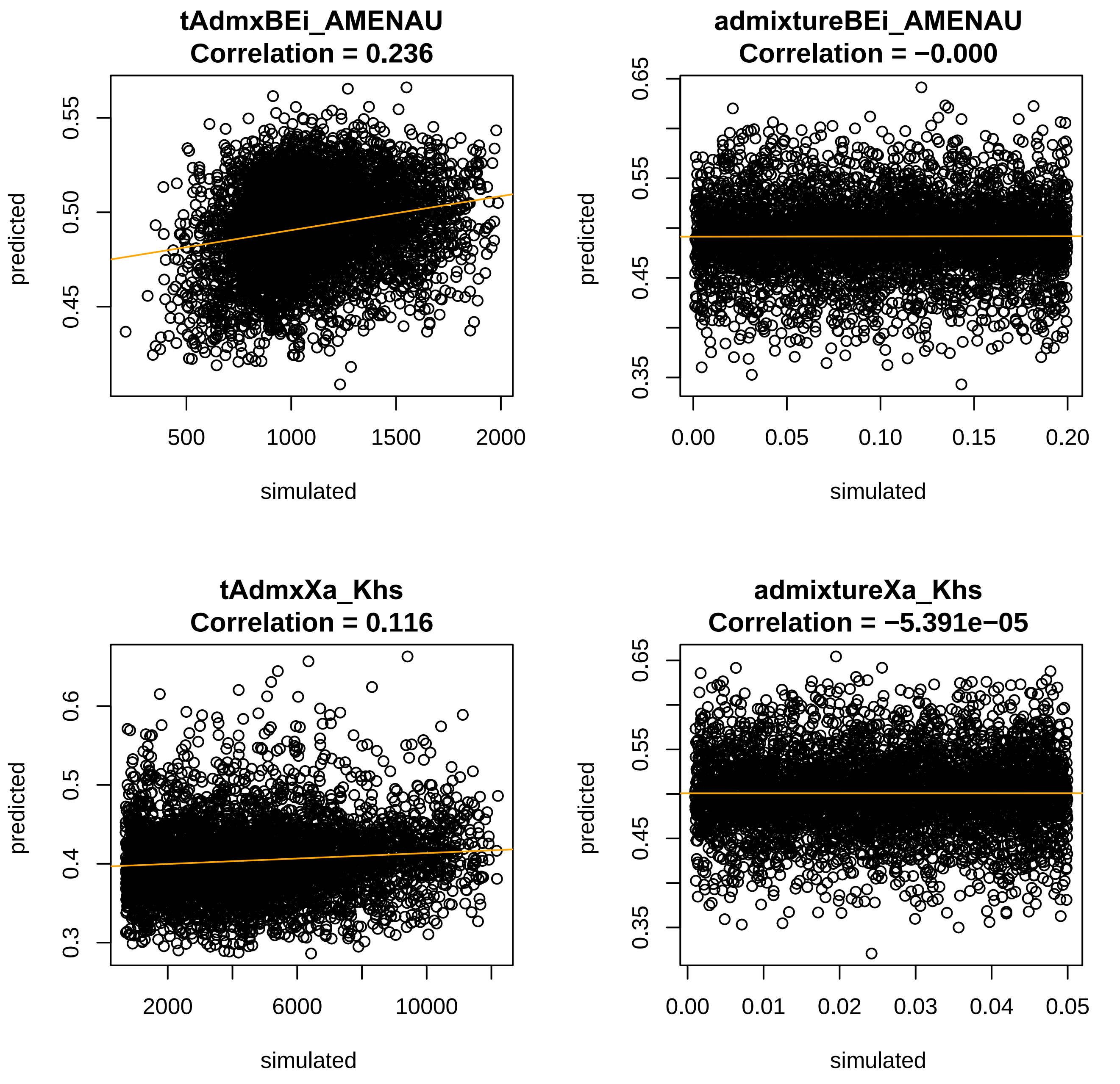
tAdmxBEi_AMENAU
Correlation = 0.236
predicted
0.45
0.50
0.55
500
1000
1500
2000
simulated
admixtureBEi_AMENAU
Correlation = −0.000
predicted
0.35
0.45
0.55
0.65
0.00
0.05
0.10
0.15
0.20
simulated
tAdmxXa_Khs
Correlation = 0.116
predicted
0.3
0.4
0.5
0.6
2000
6000
10000
simulated
admixtureXa_Khs
Correlation = −5.391e−05
predicted
0.35
0.45
0.55
0.65
0.00
0.01
0.02
0.03
0.04
0.05
simulated

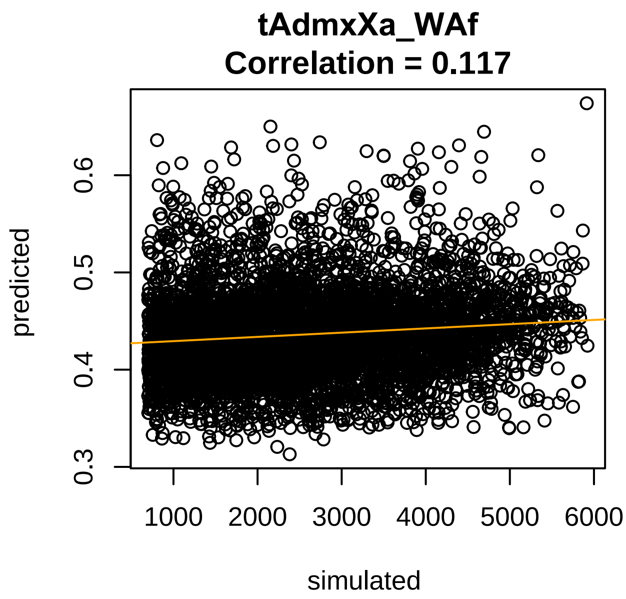
tAdmxXa_WAf
Correlation = 0.117
predicted
simulated
0.3
0.4
0.5
0.6
1000
2000
3000
4000
5000
6000

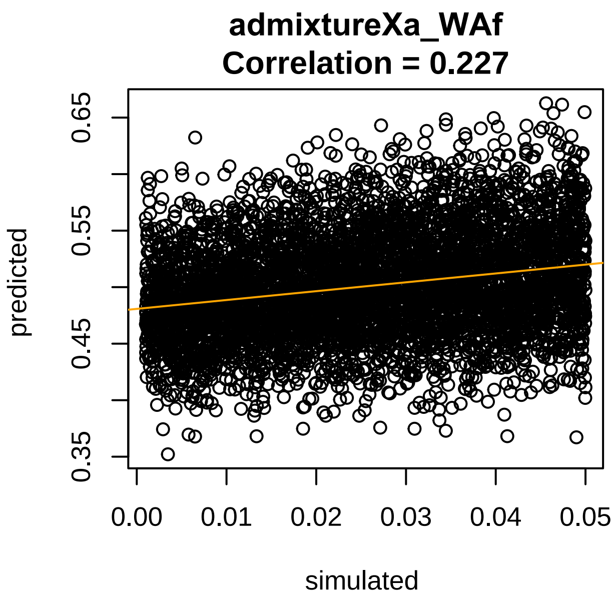
admixtureXa_WAf
Correlation = 0.227
predicted
simulated
0.35
0.45
0.55
0.65
0.00
0.01
0.02
0.03
0.04
0.05
