# Supplementary material for: Modelling the demographic history of human North African genomes points to a recent soft split divergence between populations
Source: Genome Biol. 2024 Jul 30;25:201. doi: 10.1186/s13059-024-03341-4 (PMC11290046; doi:10.1186/s13059-024-03341-4)
Supplement: Supplementary file 6 — Additional file 6. Peer review history. [file 13059_2024_3341_MOESM6_ESM.docx]

**Review history**

**First round of review**

**Reviewer 1**

In this study, the authors re-investigated the demographic history of North African populations using a new GP4PG algorithm to model the genetic substructure of these groups and reconstruct episodes of gene flow in these groups. The authors argue that this method provides a more resolved picture of the back-to-Africa origin or North African populations and their relationships with West Eurasian groups. In particular, they observed an older origin for Amazigh populations compared for Arab populations, clearly distinguishing them as the autochthonous groups of the region, whereas the Near Eastern genomic ancestry of Arab groups largely traces to the expansion of Arabic speaking groups in the 7th century CE. Unlike other models, that generated by the GP4PG model does not require multiple pulses of admixture from surrounding populations into North Africa, and instead emphasizes soft splits and population substructure as the drivers of population differentiation in North Africa.

The authors provide an extensive background for the comparative approach they take with GR4PG and ABC-DL models and also outline the methodological steps required for the use of each algorithm. They also do a nice job in describing the population of North Africa more generally.

It would be very interesting and useful to see this same approach used with genomic data sets from populations which also have complicated demographic histories, such as South Asians, or groups on island archipelagos that have experienced multiple colonization events.

While well written overall, the manuscript would benefit from some light editing.

**Reviewer 2**

In this manuscript, the authors describe a detailed analysis of human population genomics in North Africa. Their inferences support the "back-to-Africa" origin of the present inhabitants of North Africa, who presumably arrived in at least two migration pulses from the Middle East - potentially replacing earlier, autochthonous inhabitants of North Africa. The authors place a particular emphasis on the Amazigh populations (formerly "Berbers"), assigning to them a much earlier origin than what had been assumed before. This would now make the Amazigh populations as much "European" as they are "African".

Apart from these new insights into North African populations, the manuscript is mainly a methodological / technical contribution. The authors analyze the same genomic data using two distinct approaches: first, using an established Bayesian approach coupled to deep learning, and second, a novel approach using a biologically motivated class of algorithms from machine learning ("genetic programming"). They conclude that the latter approach fits the available data better and more robustly, and that the deep placement of the Amazigh population is supported by both approaches. Such a critical appraisal of competing methodologies is laudable, as is the emphasis on cross-checking and robustness testing. I find the manuscript interesting and topical, but I have a few major concerns that would have to be addressed first.

a) reproducibility
The software pipeline in which the authors have implemented their novel "GP4PG" genetic programming framework is not made available to the reader. I may have overlooked it, but I could not find a reference to github or another code repository. Since this manuscript, at its core, is a methodological contribution, I find this disappointing. As it stands, the reader cannot reproduce the work - allowing this to prevail would not be compatible with an open-science spirit.

b) missing details
Since the software was not provided, the reader has to rely on the description of the procedures in the methods section. However, there is insufficient detail to follow how exactly the GP4PG procedures are implemented. For example, the algorithm is described to follow an "isolation by distance" approach (page 27). In Figure S8, a simplified layout of the spacing of the eight populations seems to be provided. However, this Figure is never called out in the text, and it has no color-legend. Which population is which? What is the exact "migration decay function" - both over time as well as spatially ?
In a similar vein, the present manuscript describes the inclusion of 15 newly produced North African genomes (page 21, line 510). However, they are not referenced properly ("Ref EGAXXXX"), nor are they described in any detail. They represent about half of the "32 North African genomes", but apparently none of them ended up in the selection of 16 representative genomes chosen for the demographic inference. Why is that? From what lineages / proxy groups were these?

c) comparability between the models
On a more conceptual note, I am a bit concerned whether one can truly compare the ABC-DL and GP4PG models on an equal footing. For this, they would both have to be given the exact same starting data. However, the GP4PG model was provided with geographical distance information (however coarse), and the ABC-DL model was not. Conversely, the ABC-DL model was in later steps given the option to include specific migration pulses, presumably guided by previous knowledge, but the GP4PG model was not. Is this really comparable? Similarly, I find it concerning that the final, chosen "best" GP4PG model ended up being the one with inter-ecodeme migration disabled. Is this not against expections? We assume there have been at least some specific migration and introgression events, and I find it counter-intuitive that a model without any of these should be the winning model. In that sense, I find the ABC-DL outcome shown in Figure 2 more convincing and more easily interpretable. Perhaps there is an issue with a different number of parameters or "degrees of freedom" between the two models? ABC-DL would be more expressive and realistic, but had lower performance here solely because of lack of data ?

d) Amazigh focus
Given that the Amazigh populations are at the center of the new insights here, I was wondering how robust these are, given that only two Amazigh individuals were contributing (and both were sampled from the same locality). At least two more Amazigh individuals are apparently available … what would happen if these were chosen instead?

e) minor issues

- line 308: "searches" instead of "search"
- line 330: the callout should be to Figure S7, no ?
- line 372: "difficult" cannot be used as a verb

**Authors’ response to reviewers**

Reviewer 1:

It would be very interesting and useful to see this same approach used with genomic data sets from populations which also have complicated demographic histories, such as South Asians, or groups on island archipelagos that have experienced multiple colonization events.

Reply: We express our gratitude to the reviewer for their insightful comments. Furthermore, we acknowledge the potential applicability of our proposed approach in investigating complex demographic scenarios within various human populations or even in the context of species displaying intricate migration patterns. Although our current manuscript concentrates on modeling the intricate demographic dynamics of North Africa, we have taken the initiative to share both a comprehensive manual and the software necessary for conducting the GP4PG analysis with the public. This provision allows researchers with an interest in demographic inference for diverse groups to explore and implement our proposed methodology. By making these resources accessible, we aim to encourage broader applications of the GP4PG analysis across various populations, fostering a collaborative and inclusive approach to demographic modeling in human populations and potentially extending its utility to other species with complex migration patterns.

The manuscript would benefit from some light editing

Reply: We thank the reviewer for their commentaries and we revised the manuscript in order to perform the light editing suggested by the reviewer.
Reviewer 2:
The software pipeline in which the authors have implemented their novel "GP4PG" genetic programming framework is not made available to the reader. I may have overlooked it, but I could not find a reference to github or another code repository. Since this manuscript, at its core, is a methodological contribution, I find this disappointing. As it stands, the reader cannot reproduce the work - allowing this to prevail would not be compatible with an open-science spirit.

Reply: As the reviewer points out we also plead to the open-science spirit so we have made the software public in the following github https://github.com/oscarlao with a manual explaining how to reproduce the GP4PG.

Missing details

Isolation by distance" approach, migration decay function?

Reply: We thank the reviewer for pointing to this gap. We have introduced the formula in the manual of the software:


“When modeling a soft split between Ecodemes i and j, migration rates across generations decay exponentially over time according to the formula:

migration_(i,j) (t) = (〖migration〗_max-〖migration〗_min ) e^(t-T)+〖migration〗_min

Where T is the time in backward of the split between Ecodemes i and j, 〖migration〗_max is the migration at time T, and it is the maximum value of migration between the two Ecodemes, and 〖migration〗_min is the minimum migration that can be achieved between the two Ecodemes as the time of divergence between both goes to infinity.”

We also introduce it in the manuscript:

“Two Ecodemes perform a soft split if migration exponentially decays over time after the split according to the formula:

migration_(i,j) (t) = (〖migration〗_max-〖migration〗_min ) e^(t-T)+〖migration〗_min

In the context of backward time from the split between Ecodemes i and j, T represents the temporal parameter. 〖migration〗_max denotes the maximum migration occurring at time T, signifying the highest migration rate between the two Ecodemes. Conversely, 〖migration〗_min represents the minimum achievable migration as the divergence time between both Ecodemes extends to infinity. Due to the requirement of defining a new migration matrix in fastSimcoal2 each time migration is adjusted in fastSimcoal, in practice we update the migration matrix every 200 generations for efficient implementation.”

Figure S8, a simplified layout of the spacing of the eight populations seems to be provided. However, this Figure is never called out in the text, and it has no color-legend.

We have updated the figure S8 to include a color coded legend as well as calling out the figure in the text in line 704

“For the sake of simplicity, we considered that populations all occupy a same size space and that the distance between adjacent populations was zero, meaning that geographical barriers, such as the Sahara Desert or the Mediterranean Sea, were not considered to order the populations in the space (Additional file 1: Fig. S8) . “
15 newly produced North African genomes (page 21, line 510). However, they are not referenced properly ("Ref EGAXXXX")

As per told by the reviewer, we have added a brief description of the origin of the samples as well as a proper reference to this newly published genomes (REF: EGAxx). We are still waiting for the accession number, but as soon as EGA will provide it we will make it available.

none of them ended up in the selection of 16 representative genomes chosen for the demographic inference. Why is that?

We thank the reviewer for their suggestion but, although we consider those North African genomes to be included in the demographic analysis, we discarded that option since the Moroccan and Tunisian samples we collected from a set of volunteers of the Spanish Canary Island of Lanzarote with no clear identification of their cultural and geographical background in North Africa. And given that demographic inference analysis presents high sensitivity to location, without a proper identification of these parameters we could not use these new North African genomes in the analysis.

Comparability between the models

Concerned whether one can truly compare the ABC-DL and GP4PG models on an equal footing

Reply: Our contention lies in the observation that the methodology itself should yield consistent results. The discrepancy noted in ABC-DL stems from the requirement to a priori specify events within the demographic model, in contrast to GP4PG, where such events are learned through the Gaussian Process (GP) framework. In terms of parameterization, the augmenting nature of GP implies that demographic models formulated using this methodology may exhibit a higher number of parameters or events compared to an ABC-DL approach, where investigators typically aim to minimize such parameters. The elevated parameter count presents a potential susceptibility to model overfitting, limiting predictive capabilities to the data employed during training. To address this concern, we assessed the robustness of GP4PG models by subjecting them to new data, demonstrating their ability to accurately simulate the genetic variation in previously unseen datasets. Furthermore, both ABC-DL and GP4PG consistently yield identical population tree topologies.

The ABC-DL model was in later steps given the option to include specific migration pulses, presumably guided by previous knowledge, but the GP4PG model was not. Is this really comparable

Reply: The GP4PG tool incorporates the option to incorporate specific migration pulses into the analysis. While these pulses are integrated into the algorithm, they may not always be utilized in models with minimal error due to the optimization algorithm's discretion. However, it is important to note that migration pulses are examined in certain iterations, as they represent one of the evolutionary parameters that GP4PG can accommodate when constructing a model.

Similarly, I find it concerning that the final, chosen "best" GP4PG model ended up being the one with inter-ecodeme migration disabled. Is this not against expectations? We assume there have been at least some specific migration and introgression events, and I find it counter-intuitive that a model without any of these should be the winning model

Reply: We express our gratitude to the reviewer for highlighting this concern. Indeed, in the GP4PG, the "optimal" model incorporates migrations represented as soft split decays. These migration decays replicate the impact of distinct migrations and admixture pulses that were manually incorporated for each tested model in the context of ABC-DL. Therefore, GP4PG obviates the necessity for automatically introducing such events to model the diversity of North African populations. Regrettably, this clarification was not sufficiently emphasized in both the text and Figure 3. In the present version of the manuscript, we have rectified this omission by providing a comprehensive description:

“The absence of admixture events in the best model of the GP4PG algorithm can be attributed to the fact that our algorithm is weighted towards modelling population splits by a soft process in which migration decays forward in time between related populations. These migration decays replicate the impact of distinct migrations and admixture pulses that were manually incorporated for each tested model in the context of ABC-DL. Therefore, GP4PG obviates the necessity for automatically introducing such events to model the diversity of North African populations. Upon the split between two populations occurs, migration between the newly formed ecodemes continues for generations, decaying as the demes grow further apart in time following an exponential decline (Eq.1)”


Find the ABC-DL outcome shown in Figure 2 more convincing and more easily interpretable. ABC-DL would be more expressive and realistic, but had lower performance here solely because of lack of data?

Reply: The primary challenge we encountered with the ABC-DL approach lies in the fact that the best-trained demographic model fails to adequately explain the observed data. This issue cannot be solely attributed to a lack of data, as all models utilized in the ABC-DL approach handle the same volume of data. Rather, the fundamental concern arises from the absence of realistic demographic models employed during the training phase. This inherent limitation is a characteristic challenge of such approaches, as they are pre-defined by the investigator. While striking a balance between model complexity and interpretability is always sought after, the deficiency in replicability suggests that all models considered in ABC-DL are inherently inadequate representations of reality. Consequently, the historical interpretability of these models is compromised and, to a certain extent, rendered meaningless. This realization prompted our development of an alternative approach, GP4PG.

What would happen if other Amazigh were chosen instead?

Reply: We express gratitude to the reviewer for bringing this matter to our attention. It is noted that other Amazigh groups exhibit heightened levels of admixture in contrast to the Chenini Amazigh, necessitating a more complex demographic model to interpret the data comprehensively. Moreover, the North African population manifests considerable levels of admixture, thereby posing challenges in delineating between Amazigh and Arab communities. The rationale behind our selection of Chenini individuals lies in the distinctive isolating characteristics of their community, affording them a greater degree of genetic differentiation relative to other Amazigh populations, thereby rendering them the most suitable surrogate for representing the autochthonous North African demographic.
Moreover, it is imperative to acknowledge the substantial heterogeneity inherent within Amazigh populations. Utilizing individuals from diverse geographic locations may introduce complexities in demographic inference analyses, thereby jeopardizing the reproducibility of findings from the test dataset within the replication dataset. Consequently, efforts must be made to ensure the homogeneity of both samples in terms of origin or sequencing technology to bolster the robustness of the analyses conducted.

line 308: "searches" instead of "search" changed
line 330: the callout should be to Figure S7, no ? changed
line 372: "difficult" cannot be used as a verb changed

**Second round of review**

**Reviewer 2**

In their revised manuscript, the authors have addressed and discussed most of my concerns. They have added the corresponding clarifications and amendments to the manuscript, and to the supplementary material.

However, unfortunately, the GP4PG software is still not made available. The manuscript now provides a link to a github repository: "The JAVA-based pipeline to implement the Genetic Programming for Population Genetics (GP4PG) is available at https://github.com/oscarlao/GP4PG"
Unfortunately, that link provided above does not work: it yields a "404 page not found". My suspicion is that the repository exists, but has not yet been configured to be visible publicly. In my opinion, this would be a necessary prerequisite before the paper can be accepted.
